# High glucose induces phosphorylation and oxidation of mitochondrial proteins in renal tubular cells: A proteomics approach

**DOI:** 10.1038/s41598-020-62665-w

**Published:** 2020-04-03

**Authors:** Siripat Aluksanasuwan, Sirikanya Plumworasawat, Thanyalak Malaitad, Sakdithep Chaiyarit, Visith Thongboonkerd

**Affiliations:** Medical Proteomics Unit, Office for Research and Development, Faculty of Medicine Siriraj Hospital, Mahidol University, Bangkok, 10700 Thailand

**Keywords:** Mitochondrial proteins, Phosphoproteins, Phosphorylation, Proteomics, Diabetes complications

## Abstract

Mitochondrial dysfunction has been thought to play roles in the pathogenesis of diabetic nephropathy (DN). However, precise mechanisms underlying mitochondrial dysfunction in DN remained unclear. Herein, mitochondria were isolated from renal tubular cells after exposure to normal glucose (5.5 mM glucose), high glucose (25 mM glucose), or osmotic control (5.5 mM glucose + 19.5 mM mannitol) for 96 h. Comparative proteomic analysis revealed six differentially expressed proteins among groups that were subsequently identified by tandem mass spectrometry (nanoLC-ESI-ETD MS/MS) and confirmed by Western blotting. Several various types of post-translational modifications (PTMs) were identified in all of these identified proteins. Interestingly, phosphorylation and oxidation were most abundant in mitochondrial proteins whose levels were exclusively increased in high glucose condition. The high glucose-induced increases in phosphorylation and oxidation of mitochondrial proteins were successfully confirmed by various assays including MS/MS analyses. Moreover, high glucose also increased levels of phosphorylated ezrin, intracellular ATP and ROS, all of which could be abolished by a p38 MAPK inhibitor (SB239063), implicating a role of p38 MAPK-mediated phosphorylation in high glucose-induced mitochondrial dysfunction. These data indicate that phosphorylation and oxidation of mitochondrial proteins are, at least in part, involved in mitochondrial dysfunction in renal tubular cells during DN.

## Introduction

Mitochondria play important roles in many cellular processes, e.g., ATP production, calcium homeostasis, regulation of cellular metabolism, and apoptotic cell death^1^. Mitochondrial dysfunction frequently causes cellular injury and is involved in the pathogenesis of several diseases^2^. In diabetes, overproduction of reactive oxygen species (ROS) is a result of mitochondrial dysfunction and has been proposed as one of the key initiators contributing to development of diabetic nephropathy (DN)^3^. Moreover, high glucose-induced mitochondrial dysfunction results to a decline of ATP production and activation of apoptotic pathway, which ultimately leads to renal cell injury and death^4,5^. Several studies have suggested that DN is associated with alterations in mitochondrial dynamics, morphology, and autophagic removal of damaged mitochondria (mitophagy), which play important roles in regulation of mitochondrial function and homeostasis^6^. The enlargement of mitochondria in proximal renal tubular cells has been reported to correlate with microalbuminuria in diabetic rats^7^. Recent studies have demonstrated that hyperglycemia induces mitochondrial fission and fragmentation in renal cells that are associated with ROS overproduction, increased mitophagy and apoptosis^8,9^. These lines of evidence indicate that high glucose causes mitochondrial dysfunction leading to renal cell injury. Nevertheless, precise mechanisms underlying mitochondrial dysfunction induced by diabetes are not well understood and remain to be elucidated.

Our present study, therefore, investigated changes in mitochondrial proteome of renal tubular cells induced by high glucose by using a proteomics approach. Mitochondria were isolated and purified from the cells incubated under normal glucose, high glucose, or high osmolarity (osmotic control) condition. Differentially expressed proteins were then identified by tandem mass spectrometry (nanoLC-ESI-ETD MS/MS) and subjected to analysis for potential post-translational modifications (PTMs) followed by validation with various assays.

## Results

### Purity of mitochondrial isolation

**Figure 1:**
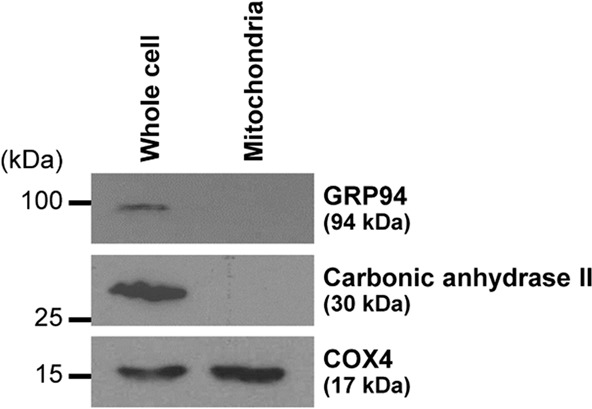
Purity of mitochondrial isolation. Immunoreactive bands of markers for mitochondria (COX4), cytoplasm (carbonic anhydrase II), and endoplasmic reticulum (GRP94) in whole cell lysate and mitochondrial fraction are shown. The full-membrane images of these blots are provided in Supplementary Fig. S1.

Mitochondria were isolated from Madin-Darby canine kidney (MDCK) renal tubular cells by sonication followed by differential centrifugation as detailed in “Materials and Methods”. The purity of the isolated mitochondria was evaluated by Western blotting. The data showed an enrichment of the mitochondrial marker (cytochrome c oxidase subunit 4) (COX4) in mitochondrial fraction, whereas contaminations from other cellular compartments were not detected in the mitochondrial samples (using carbonic anhydrase II and glucose regulated protein 94 kDa (GRP94) as the markers for cytoplasm and endoplasmic reticulum, respectively) (Fig. 1). These data indicated that mitochondria were successfully isolated and enriched with no contaminations from other cellular compartments.

### Altered mitochondrial proteome induced by high glucose

**Figure 2:**
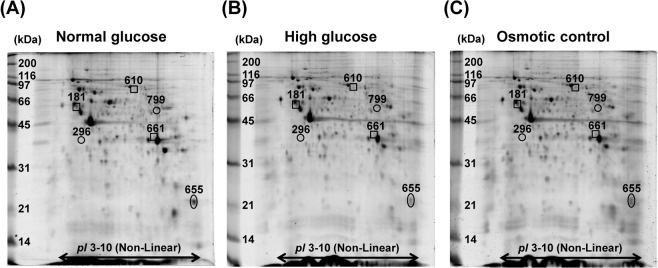
2-D proteome map of differentially expressed mitochondrial proteins. (**A**–**C**) Representative 2-D gels, stained with Deep Purple fluorescence dye, of mitochondrial proteins under normal glucose, high glucose, and osmotic control, respectively (n = 5 gels were derived from 5 individual culture flasks for each condition; a total of 15 gels were subjected to comparative analysis). Differentially expressed proteins are labelled with numbers, which correspond to those reported in Tables 1 and 2.

**Figure 3:**
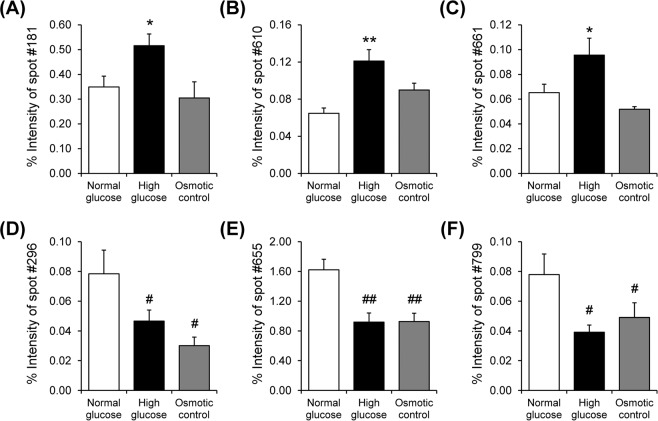
Intensity levels of differentially expressed protein spots. (**A**–**C**) Intensity levels of spots no. 181, 610, and 661, respectively, which were increased exclusively in high glucose condition. (**D**–**E**) Intensity levels of spots no. 296, 655, and 799, respectively, which were decreased in both high glucose and osmotic control conditions. Each bar represents mean ± SEM of intensity data obtained from five gels per group. *p < 0.05 vs. normal glucose and osmotic control; **p < 0.01 vs. normal glucose and osmotic control; #*p* < 0.05 vs. normal glucose; ## *p* < 0.01 vs. normal glucose.

After exposure to normal glucose (5.5 mM glucose), high glucose (25 mM glucose), or osmotic control (5.5 mM glucose + 19.5 mM mannitol) condition for 96 h, mitochondria were isolated and their proteome profiles were analyzed by 2-DE (n = 5 gels were derived from 5 individual culture flasks for each condition; a total of 15 gels were subjected to comparative analysis). Using Deep Purple fluorescence dye, spot matching and quantitative intensity analysis revealed six protein spots whose levels significantly differed among groups (Fig. 2). From these, three spots were exclusively increased by high glucose as compared to normal glucose and osmotic control conditions (Fig. 3A–C), whereas other three were decreased in both high glucose and osmotic control conditions (Fig. 3D–F). These differentially expressed mitochondrial proteins were subsequently identified by nanoLC-ESI-ETD MS/MS (Table 1). Their MS/MS fragmentation data are shown in Supplementary Table S1, whereas MS/MS spectra of all individual peptides assigned to each identified protein are illustrated in Supplementary Fig. S2. In addition, their molecular and biological functions are summarized in Table 2.

**Table 1:** Summary of differentially expressed mitochondrial proteins in MDCK cells after exposure to normal glucose (NG), high glucose (HG), or osmotic control (OS).

Spot no.	Protein name	NCBI ID	MS/MS score	%Cov	No. of distinct/total matched peptides	p*I*	MW (kDa)	Intensity (Mean ± SEM)			Ratio			ANOVA *p* value
								NG	HG	OS	HG/NG	HG/OS	OS/NG	
**HG-specific changes (increased)**														
181	Tubulin beta-2A chain	gi|4507729	879	35	14/28	4.78	50.27	0.3491 ± 0.0438	0.5158 ± 0.0472	0.3048 ± 0.0652	**1**.**48**	**1**.**69**	0.87	0.0365
610	Prelamin A/C	gi|560891942	1204	36	24/27	6.57	74.47	0.0647 ± 0.0058	0.1211 ± 0.0122	0.0898 ± 0.0074	**1**.**87**	**1**.**35**	1.39	0.0027
661	Annexin A2	gi|50950177	892	50	18/23	6.92	38.92	0.0653 ± 0.0068	0.0956 ± 0.0136	0.0519 ± 0.0021	**1**.**46**	**1**.**84**	0.79	0.0125
**Changes induced by both HG and OS (decreased)**														
296	Elongation factor 1-delta	gi|587020223	240	16	4/4	5.27	33.02	0.0784 ± 0.0159	0.0467 ± 0.0073	0.0301 ± 0.0058	**0**.**60**	1.55	**0**.**38**	0.0221
655	Peptidyl-prolyl cis-trans isomerase B isoform 2	gi|74000476	875	59	16/41	9.33	23.82	1.6213 ± 0.1433	0.9199 ± 0.1203	0.9275 ± 0.1105	**0**.**57**	0.99	**0**.**57**	0.0025
799	ATP synthase subunit beta, mitochondrial precursor	gi|31980648	630	26	12/15	5.19	56.27	0.0779 ± 0.0138	0.0392 ± 0.0048	0.0490 ± 0.0099	**0**.**50**	0.80	**0**.**63**	0.0493

NCBI = National Center for Biotechnology Information.

%Cov = %Sequence coverage = (number of the matched residues/total number of residues in the entire sequence) × 100%.

**Table 2:** Molecular and biological functions of the identified proteins^a^.

Spot no.	Protein name	Gene name	Molecular function	Biological function
181	Tubulin beta-2A chain	*TUBB2A*	GTPase activity, GTP binding	Microtubule-based process, organelle organization, regulation of mitochondrial respiration
296	Elongation factor 1-delta	*EEF1D*	Elongation factor	Protein biosynthesis, transcription, transcription regulation
610	Prelamin A/C	*LMNA*	Structural molecule activity	Regulation of protein localization to nucleus, nucleus organization
655	Peptidyl-prolyl cis-trans isomerase B isoform 2	*PPIB*	Isomerase, rotamase	Chaperone-mediated protein folding, protein peptidyl-prolyl isomerization
661	Annexin A2	*ANXA2*	Calcium ion binding	Membrane budding, membrane raft assembly, regulation of vesicle fusion
799	ATP synthase subunit beta, mitochondrial precursor	*ATP5B*	Hydrolase	ATP synthesis, mitochondrial organization

^a^Obtained from the UnitProt Protein Knowledgeable Database and literatures.

### Validation of the proteomic data by Western blotting

**Figure 4:**
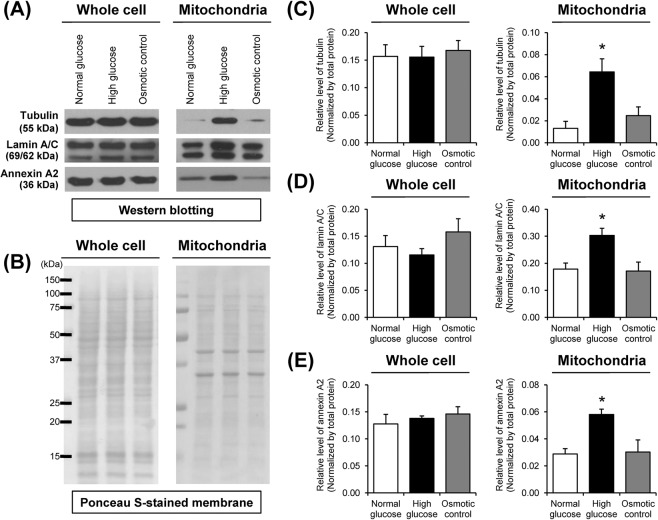
Validation of the proteomic data by Western blot analysis. (**A**) Immunoreactive bands of tubulin, lamin A/C and annexin A2 in whole cell and mitochondrial fraction. The full-membrane images of these blots are provided in Supplementary Fig. S3. (**B**) Ponceau S-stained membranes were used to verify equal protein loading in each sample. (**C**) Band intensity of tubulin normalized with total protein. (**D**) Band intensity of lamin A/C normalized with total protein. (**E**) Band intensity of annexin A2 normalized with total protein. Each bar represents mean ± SEM of intensity data obtained from three independent samples. *p < 0.05 vs. normal glucose and osmotic control.

Western blotting was performed to confirm high glucose-induced changes in mitochondrial protein levels as identified by proteomics approach. The data showed that the increases of tubulin, lamin A/C and annexin A2 exclusively in high glucose condition were nicely confirmed in mitochondrial fraction, whereas their levels in whole cell lysate were not changed significantly (Fig. 4).

### Identification of potential post-translational modification (PTMs)

**Figure 5:**
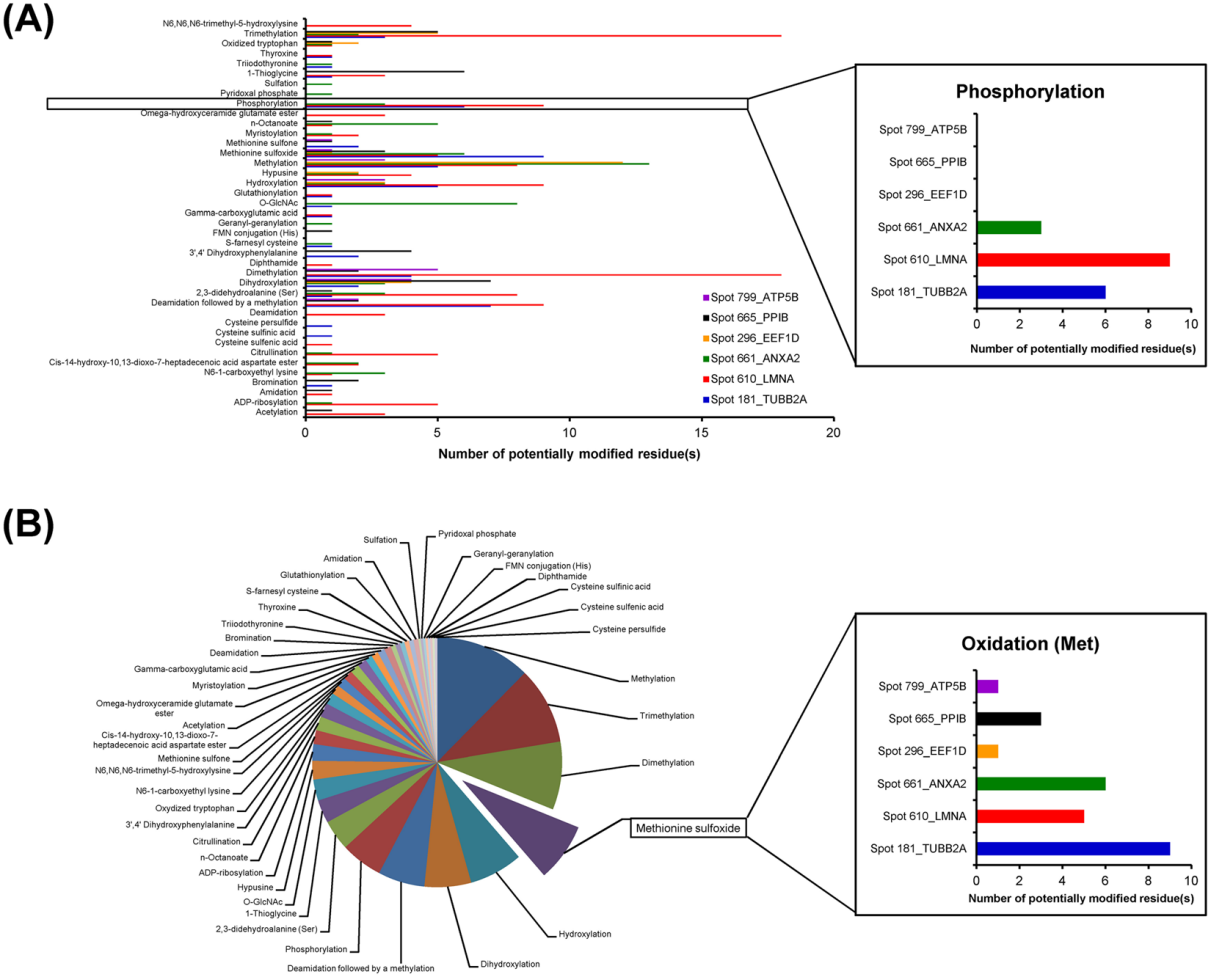
Potential PTMs identified from all differentially expressed mitochondrial proteins. (**A**) Number of potentially modified residues by individual PTMs in “each protein” identified by nanoLC-ESI-ETD MS/MS. (**B**) Summation of all the potentially modified residues by individual PTMs in “all proteins” identified. Insets demonstrate the zoom-in details of phosphorylation and oxidation, respectively. See more details in Supplementary Table S2.

From all differentially expressed mitochondrial proteins listed in Table 1, their peptides identified by nanoLC-ESI-ETD MS/MS were subjected to PTMs analysis, which identified several various types of PTMs in all of these identified proteins (Fig. 5). Types and other information of all these potential PTMs as well as details of the modified residues identified in each protein are summarized in Supplementary Table S2. Interestingly, phosphorylation and oxidation were most abundant in the mitochondrial proteins, whose levels were exclusively increased in high glucose condition (Fig. 5). Validation of their increases in mitochondrial proteome under the high glucose condition was done as follows.

### Increased levels of mitochondrial phosphoproteins by high glucose

**Figure 6:**
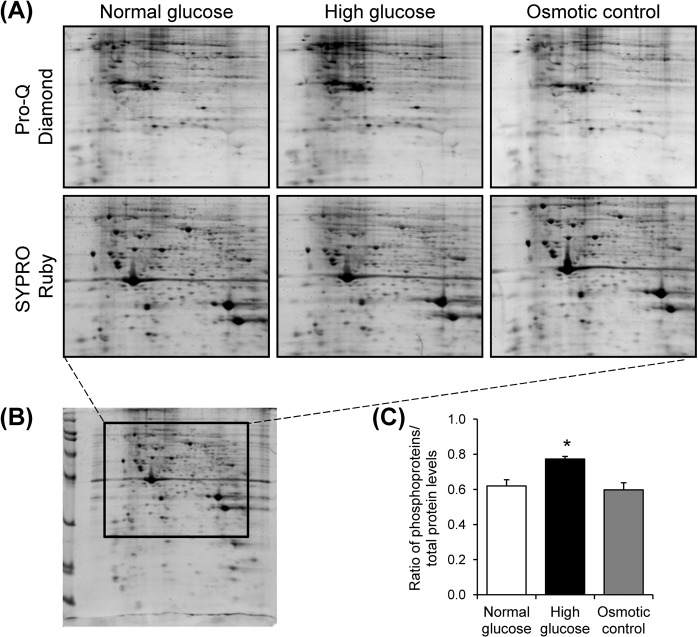
Detection and quantitative analysis of mitochondrial phosphoproteins. (**A**) Zoom-in images of representative 2-D gels, stained with Pro-Q Diamond phosphoprotein gel stain followed by SYPRO Ruby total protein gel stain, of mitochondrial proteins under normal glucose, high glucose, and osmotic control, respectively (n = 3 gels were derived from 3 individual culture flasks for each condition; a total of 9 gels were analyzed). The full-length gels of these zoom-in images are shown in Supplementary Fig. S4. (**B**) Demonstrates the whole 2-D gel, of which 2-D spot pattern was consistent with that of the Deep Purple stained gels used for initial comparative analysis (shown in Fig. 1). (**C**) Quantitative analysis of levels of phosphoproteins (Pro-Q Diamond) in relative to (normalized with) total protein (SYPRO Ruby) detected in each group. Each bar represents mean ± SEM of intensity data obtained from three individual gels per group. *p < 0.05 vs. normal glucose and osmotic control.

Mitochondrial proteins derived from each sample were resolved in 2-DE (n = 3 gels were derived from 3 individual culture flasks for each condition; a total of 9 gels were subjected to phosphoprotein analysis). The resolved proteins were then visualized by Pro-Q Diamond phosphoprotein gel stain followed by staining with SYPRO Ruby total protein gel stain (Fig. 6A,B). After normalization with total protein, the data demonstrated that levels of phosphoproteins were exclusively increased in high glucose condition as compared to the other two conditions (Fig. 6C), confirming the data obtained from MS/MS analysis.

### Increased levels of oxidatively modified proteins in mitochondria and intracellular ROS by high glucose

**Figure 7:**
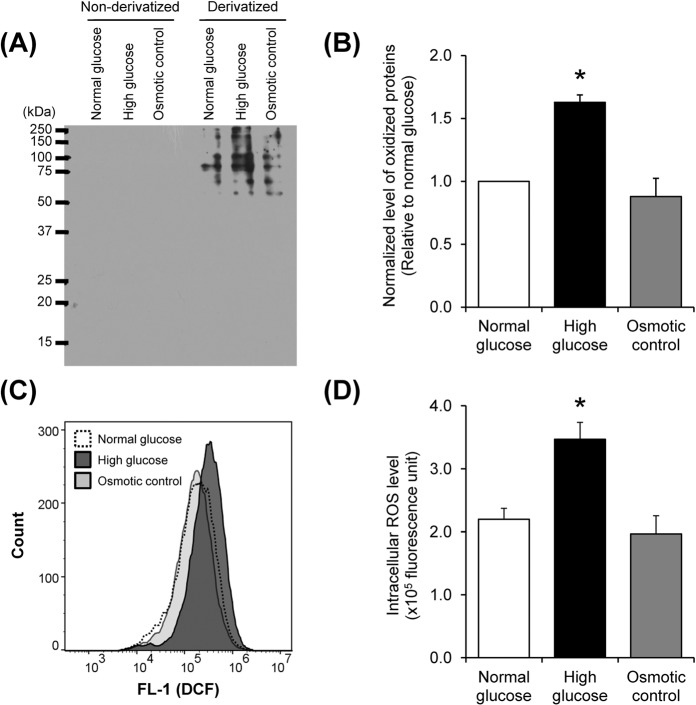
Oxidative stress assays. (**A**) Immunoreactive bands of oxidized mitochondrial proteins as detected by Oxyblot assay. (**B**) Quantitative analysis of oxidized mitochondrial proteins. (**C**) Histograms of DCF fluorescence intensity representing ROS level measured by flow cytometry. (**D**) Quantitative analysis of intracellular ROS level. Each bar represents mean ± SEM of intensity data obtained from three independent samples per group. *p < 0.05 vs. normal glucose and osmotic control.

Oxyblot assay was performed to verify that oxidation was enhanced in mitochondria under the high glucose condition. The result showed that levels of oxidatively modified proteins were exclusively increased in high glucose condition as compared to the other two conditions (Fig. 7A,B), confirming the data obtained from MS/MS analysis. In addition, DCFH-DA assay revealed the intracellular ROS overproduction under high glucose condition (Fig. 7C,D). These data indicated that high glucose induced mitochondrial ROS deregulation, resulting to oxidative damage of mitochondrial proteins.

### Mass spectrometric analyses of phosphorylated and oxidatively modified proteins

**Figure 8:**
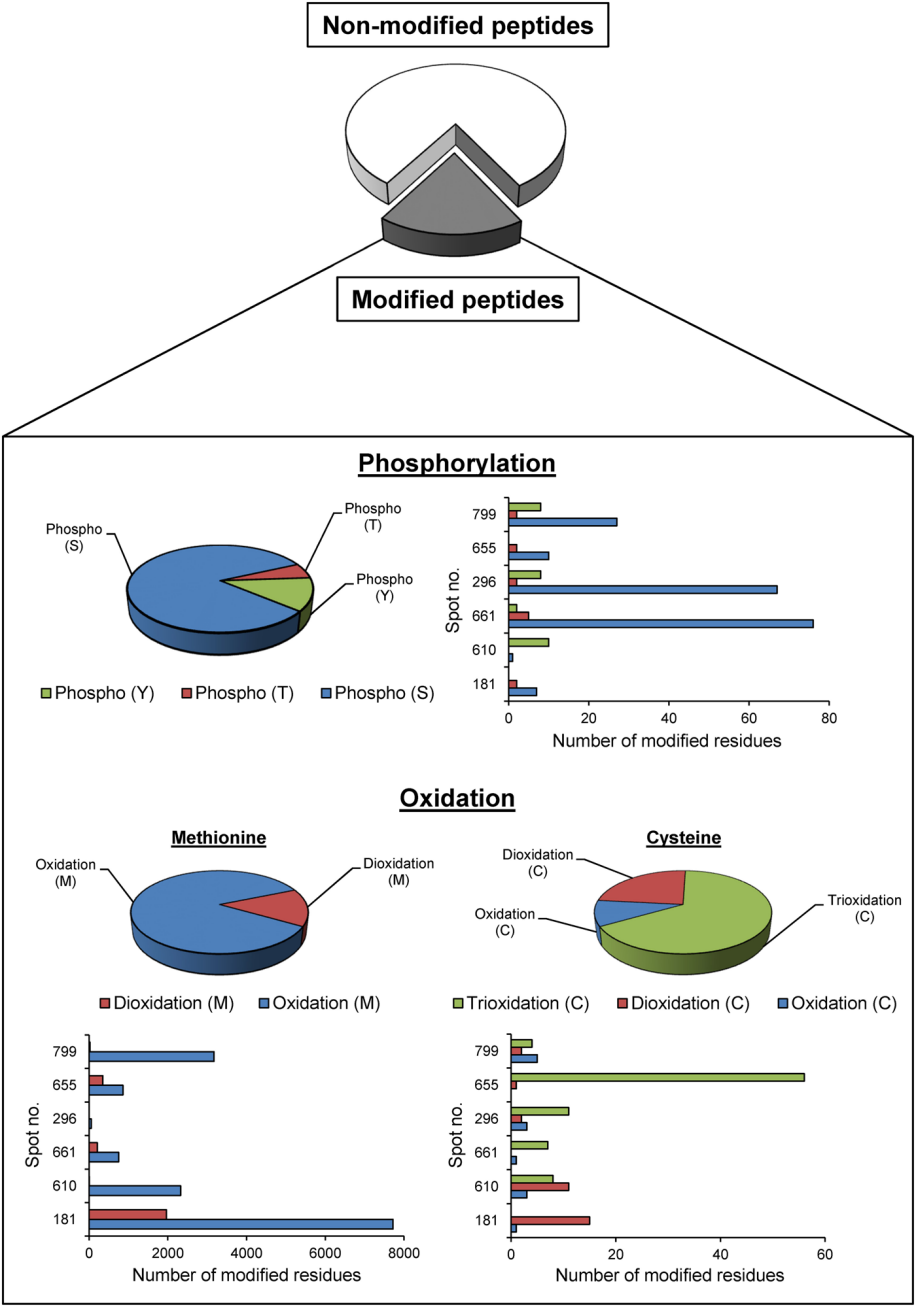
MS/MS analyses of phosphorylated and oxidatively modified proteins. Pie charts and bar graphs summarize all of the identified peptides from all differentially expressed mitochondrial proteins. The data include the proportion of non-modified and modified peptides, percentage and number of the modified residues, specifically with phosphorylation at serine (S), threonine (T) or tyrosine (Y), oxidation or dioxidation at methionine (M), and oxidation, dioxidation or trioxidation at cysteine (C). See illustrative MS/MS spectra with phosphorylated or oxidatively modified residue(s) in Supplementary Figs. S5 and S6.

MS/MS analyses of phosphorylated and oxidatively modified proteins were done to confirm the increases of protein phosphorylation and oxidation under the high glucose condition. The data showed that approximately 19% of total peptides identified in high glucose condition were either phosphorylated or oxidatively modified (Fig. 8), confirming the results obtained from phosphoprotein staining and Oxyblot assay. In addition, both types of PTMs were identified in all differentially expressed mitochondrial proteins (Fig. 8). The representative MS/MS spectra of phosphorylated and oxidatively modified peptides obtained from individual differentially expressed proteins are illustrated in Supplementary Figs. S5 and S6, respectively.

### Increased levels of phosphorylated ezrin (p-ezrin) and intracellular ATP by high glucose

**Figure 9:**
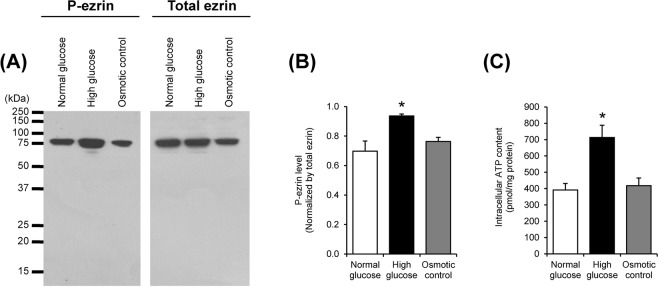
Levels of phosphorylated ezrin (p-ezrin) and intracellular ATP. (**A**) Immunoreactive bands of p-ezrin and total ezrin in the cells. (**B**) Band intensity of p-ezrin normalized with that of total ezrin. (**C**) Intracellular ATP level measured by luciferin-luciferase ATP assay. Each bar represents mean ± SEM of the data obtained from three independent samples. *p < 0.05 vs. normal glucose and osmotic control.

To assess the relevance of mitochondrial protein phosphorylation to mitochondrial dysfunction, levels of p-ezrin and intracellular ATP, were evaluated by Western blotting and luciferin-luciferase ATP assay, respectively. The results showed that level of the phosphorylated form of ezrin (p-ezrin) was significantly increased in high glucose condition, whereas its total level was not altered (Fig. 9A,B). In addition, an elevation of intracellular ATP was also found under high glucose condition (Fig. 9C). These data implicate the relevance of an increased phosphorylation of mitochondrial proteins in mitochondrial dysfunction induced by high glucose.

### Involvement of p38 MAPK-mediated phosphorylation in mitochondrial dysfunction induced by high glucose

**Figure 10:**
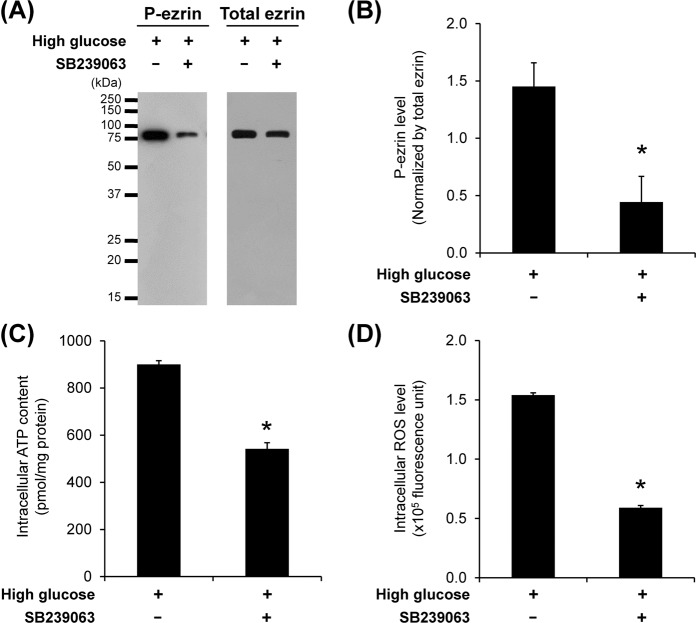
Effects of p38 MAPK inhibitor (SB239063) on levels of p-ezrin, intracellular ATP and ROS. (**A**) Immunoreactive bands of p-ezrin and total ezrin in high glucose-treated cells without or with SB239063 cotreatment. (**B**) Band intensity of p-ezrin normalized with total ezrin in high glucose-treated cells without or with SB239063 cotreatment. (**C**) Intracellular ATP level detected by luciferin-luciferase ATP assay. (**D**) Intracellular ROS level measured by DCFH-DA assay and flow cytometry. Each bar represents mean ± SEM of the data obtained from three independent samples. *p < 0.05 vs. high glucose.

Role of p38 MAPK (mitogen-activated protein kinase)-mediated phosphorylation in mitochondrial dysfunction induced by high glucose was assessed by using SB239063, a selective p38 MAPK inhibitor. The result from Western blotting showed that high glucose-induced increase of p-ezrin was significantly reduced in the cells cotreated with SB239063 (Fig. 10A,B). In addition, luciferin-luciferase ATP and DCFH-DA assays revealed that SB239063 could also abolish the induction effects of high glucose on levels of intracellular ATP (Fig. 10C) and intracellular ROS (Fig. 10D). These data suggest that p38 MAPK-mediated phosphorylation was, at least in part, involved in mitochondrial dysfunction in renal tubular cells induced by high glucose.

## Discussion

Hyperglycemia has been recognized as a key factor contributing to the pathogenesis of DN^10^. Previous studies have shown that hyperglycemia caused mitochondrial dysfunction, leading to oxidative stress, cellular injury, and cell death^4,5^. However, mechanisms by which high glucose induces mitochondrial dysfunction in renal cells remained unclear. Our present study therefore applied a proteomics approach to identify the altered mitochondrial proteins in MDCK renal tubular cells upon exposure to high glucose for 96 h. A previous cellular proteome study has shown that exposure of MDCK cells with high glucose (25 mM glucose) for 96 h caused significant changes in levels of many cellular proteins involving various functions, while severe cytotoxicity was not detected^11^. Therefore, this treatment condition was applied in the present mitochondrial proteome study.

In this study, comparative analysis revealed six differentially expressed mitochondrial proteins among normal glucose, high glucose, and osmotic control conditions (Fig. 2 and Table 1). These changes were induced either by high glucose specifically or by both high glucose and hyperosmolarity (Fig. 3 and Table 1). Among these, the altered mitochondrial proteins induced exclusively by high glucose seemed to be directly involved in the pathogenesis of DN, whereas those induced by both high glucose and hyperosmolarity were most likely the cellular response to hyperosmotic stress, which might not be specific to high glucose. The potential roles of altered proteins specifically induced by high glucose in mitochondrial dysfunction are discussed as follows.

Tubulin beta-2A chain is an isoform of β-tubulin that heterodimerizes with α-tubulin to form microtubules^12^. It has been recognized to play important roles in several cellular processes, e.g., maintaining cell shape, cell motility, cell division, and intracellular transport^13^. Additionally, several lines of evidence have shown its roles in regulation of mitochondrial function. Recent studies have shown that β-tubulin is also localized at mitochondrial membrane and regulates mitochondrial function via interacting with voltage-dependent anion channel (VDAC)^14,15^. Binding of tubulin to VDAC induces voltage-dependent channel closure, resulting to decreases in mitochondrial outer membrane permeability and respiration^16,17^. In our present study, proteomic analysis and Western blotting revealed the increased level of tubulin in mitochondria under the high glucose condition without change in its total level in whole cell (Figs. 3 and 4). These findings implicated that high glucose caused an increase in anchoring of tubulin to mitochondria rather than the increase in its expression level in the cells. Association of tubulin with mitochondria is required for maintaining mitochondrial function^18^. Interestingly, removal of β-tubulin from mitochondrial outer membrane by short proteolysis results in altered mitochondrial sensitivity for ADP and increased permeability of VDAC in cardiac cells^19^. Therefore, the increased tubulin in mitochondrial fraction observed in our present study might be related to its mitochondrial anchoring that the cells required for maintaining mitochondrial activity under the high glucose condition. However, precise mechanism underlying this phenomenon needs further elucidations.

Prelamin A/C is a precursor of lamin A/C that undergoes proteolytic cleavage of carboxyl terminus to form mature lamin A/C^20^. This protein is a major component of nuclear lamina^21^ and has been recognized to play essential roles in regulation of nuclear structure and functions involving DNA replication, transcription, and cell proliferation^22^. Although the role of lamin A/C in mitochondrial function is currently not well understood, a previous study has shown that defect (mutation) in *LMNA* gene caused mislocalization of mitochondria in nuclei of cardiomyocytes^23^. Our present study observed the increase of lamin A/C in mitochondria of the high glucose-treated cells, which was clearly confirmed by Western blot analysis (Figs. 3 and 4). Hence, its increase might reflect some mechanisms that were involved in regulation of mitochondrial morphology and localization upon high glucose exposure.

Annexin A2 is a calcium- and phospholipid-binding protein that plays crucial roles in intracellular calcium signaling, membrane organization and dynamics, and regulation of vesicular fusion^24,25^. Previous studies have shown that mitochondrial dysfunction induced by diabetes is associated with mitochondrial calcium overload and mitochondrial membrane dynamic change^8,26^. Triggering mitochondrial fission by hyperglycemia is mediated through Rho-associated coiled coil-containing protein kinase (ROCK) pathway^8^. Interestingly, annexin A2 has been reported to serve as an upstream regulator of the RhoA/ROCK pathway by regulating F-actin organization^27^. In the present study, we identified a significant increase of annexin A2 exclusively in mitochondria under high glucose condition (Figs. 3 and 4). It was thus plausible that the increase in mitochondrial annexin A2 might be involved in regulation of calcium homeostasis and mitochondrial dynamics during high glucose exposure.

Because PTMs are the crucial processes to regulate protein properties and functions^28^, we therefore evaluated potential PTMs in all of the identified proteins. A wide variety of PTMs were found in all of the differentially expressed mitochondrial proteins identified by nanoLC-ESI-ETD MS/MS (Supplementary Table S2). Most of these PTMs were randomly found in each protein without distinct pattern. Interestingly, phosphorylation and oxidation were more frequently found in mitochondrial proteins whose levels were significantly increased exclusively in the high glucose condition (Fig. 5). We thus confirmed this distinct finding by using Pro-Q Diamond phosphoprotein gel stain and Oxyblot assay. Both assays nicely confirmed the proteomic data demonstrating that levels of phosphorylated and oxidized mitochondrial proteins were significantly increased exclusively in the high glucose condition (Figs. 6 and 7). Moreover, the increased intracellular ROS level exclusively by high glucose strengthened the initial results. These data indicate that phosphorylation and oxidation are the important PTMs involving mitochondrial dysfunction induced by high glucose.

Several studies have demonstrated the role for protein phosphorylation in regulation of mitochondrial activities^29,30^. Many of mitochondrial phosphoproteins are involved in mitochondrial processes (e.g., oxidative phosphorylation and tricarboxylic acid cycle) and contain multiple phosphorylation sites that are potentially regulated by various kinases^31^. Additionally, phosphorylation of mitochondrial proteins can also cause various effects. For instance, protein kinase C-mediated serine phosphorylation of ATP synthase subunit β leads to a decrease in ATP synthase activity in renal proximal tubules^32^. In contrast, phosphorylation of COX4 (isoform 1) by protein kinase A results in enhancement of COX4 activity and respiration in mouse fibroblasts^33^. These lines of evidence underscore the significant role of phosphorylation of mitochondrial proteins in regulation of mitochondrial function.

MS/MS analyses of PTMs in differentially expressed mitochondrial proteins confirmed that both phosphorylation and oxidation were induced by high glucose. Among the three common sites for phosphorylation, serine was mostly affected by phosphorylated modification (Fig. 8) and thus might be related with mitochondrial dysfunction induced by high glucose. For protein oxidation, it has been evidenced that activation of mitochondrial protein kinase may promote catalytic metabolism and electron transport that subsequently induce ROS overproduction^34^. Furthermore, most of methionine residues were found oxidized in the differentially expressed proteins. In addition, dioxidation at methionine residues was found along with over-oxidation at cysteine residues indicating that degree of protein oxidation was amplified by high glucose. These data reflect the incapability of mitochondrial proteins to recover their functions and oxidative phosphorylation complex might be formed leading to more oxidative damage and ROS overproduction^35,36^. Furthermore, protein phosphatase that contains more cysteine residues can be inactivated by oxidation, thereby impairing dephosphorylation activity^37,38^. All of these sequelae can be accompanied with disruption of various mitochondrial functions, increase in phosphorylation and activation of apoptotic pathway.

In this study, ezrin was investigated and used as a model protein for evaluation of phosphorylation status of the cells under high glucose condition because it is a cytoskeletal linker that is known to be associated with mitochondria^39,40^. Additionally, ezrin has been reported to involve modulation of mitochondrial function^40^. Our data demonstrated the exclusive increase in level of p-ezrin by high glucose induction (Fig. 9A,B). Such increase was concomitantly found with an elevation of intracellular ATP in the high glucose-treated cells (Fig. 9C). These data implicate the relevance of mitochondrial protein phosphorylation in mitochondrial dysfunction in renal tubular cells induced by high glucose.

P38 MAPK is a kinase that plays important roles in the pathogenesis of DN. It has been shown that high glucose induces activation of p38 MAPK in renal tubular cells, leading to cellular injury and development of DN^41,42^. In addition, p38 MAPK has been also reported to localize in mitochondria^43,44^. Blockage of this pathway could attenuate mitochondrial dysfunction in various pathological conditions^45–47^. In the present study, we therefore assessed the role of p38 MAPK in mitochondrial dysfunction induced by high glucose. The data showed that inhibition of p38 MAPK using SB239063 could reduce the level of p-ezrin in the high glucose-treated cells (Fig. 10A,B). Moreover, the effect of high glucose on elevation of intracellular ATP and ROS levels could be ameliorated by cotreatment with SB239063 (Fig. 10C,D). These findings indicate that p38 MAPK-mediated phosphorylation plays a role in regulation of mitochondrial dysfunction in renal tubular cells under high glucose condition.

In summary, our present study has demonstrated alterations in mitochondrial proteome and protein phosphorylation in renal tubular cells induced by high glucose. In addition, high glucose also caused oxidative dysregulation in mitochondria, resulting to accumulation of intracellular ROS. These data indicate that phosphorylation and oxidation of mitochondrial proteins are, at least in part, involved in mitochondrial dysfunction in renal tubular cells during DN.

## Materials and Methods

### Cell culture

MDCK cells were maintained in growth medium containing Eagle’s minimal essential medium (MEM) (Gibco; Grand Island, NY) supplemented with 10% (v/v) heat-inactivated fetal bovine serum (FBS) (Gibco), 60 U/ml penicillin G, and 60 µg/ml streptomycin (Sigma; St. Louis, MO) as described previously^48,49^. The cells were then incubated in conditioned medium with normal glucose (5.5 mM glucose), high glucose (25 mM glucose), or high osmolarity (osmotic control) (5.5 mM glucose + 19.5 mM mannitol) for 96 h at 37 °C in a humidified incubator with 5% CO_2_. To examine the effects of SB239063 (a p38 MAPK inhibitor) on the high glucose induction, the cells were incubated in the medium containing high glucose (25 mM glucose) and 20 μM SB239063 for 96 h comparing to those exposed to high glucose without SB239063. The cells were then harvested and subjected to investigations as described below.

### Mitochondrial isolation

Mitochondria were isolated from the cells by sonication followed by differential centrifugation, as described previously^39,50^. Briefly, the cells were sonicated in an isolation buffer containing 0.25 M sucrose and 10 mM HEPES (pH 7.5) using a Bandelin Sonopuls HD 200 probe sonicator (Bandelin Electronic; Berlin, Germany) at MS 72/D (50 cycles) for 10 sec in an icebox. The samples were then centrifuged at 1,000 *g* for 10 min to remove intact cells and debris. The supernatants were collected and then centrifuged at 20,000 *g* for 25 min. The pellets were saved, washed once with fresh isolation buffer (0.25 M sucrose and 10 mM HEPES; pH 7.5), and then centrifuged again at 20,000 *g* for 25 min. The mitochondrial pellets were then saved for subsequent experiments. Their purity was examined by Janus green B staining, transmission electron microscopic (TEM) examination, and Western blot analyses of markers for individual subcellular organelles, as previously described^39,50^. Mitochondrial pellets were resuspended in a buffer containing 7 M urea, 2 M thiourea, 4% 3-[(3-cholamidopropyl) dimethyl-ammonio]-1-propanesulfonate (CHAPS), 120 mM dithiothreitol (DTT), 2% ampholytes (pH 3–10) and 40 mM Tris-HCl, and further incubated at 4 °C for 30 min. Protein concentrations in individual samples were measured by Bradford’s method using Bio-Rad protein assay (Bio-Rad Laboratories; Hercules, CA).

### Two-dimensional gel electrophoresis (2-DE)

For comparative analysis of mitochondrial proteome, proteins derived from individual mitochondrial samples were resolved in individual 2-D gels (n = 5 gels were derived from 5 individual culture flasks for each condition; a total of 15 gels were subjected to comparative analysis) as described previously^51,52^. Proteins with an equal amount (100 µg for each sample) were premixed with a rehydration buffer containing 7 M urea, 2 M thiourea, 2% CHAPS, 120 mM DTT, 40 mM Tris-base, 2% ampholytes (pH 3–10) and bromophenol blue to make the final volume of 150 μl/sample. The mixtures were rehydrated onto immobilized pH gradient (IPG) strips (p*I* 3–10, non-linear) (GE Healthcare; Uppsala, Sweden) at 25 °C for 16 h. The first dimensional separation or isoelectric focusing (IEF) was performed in Ettan IPGphor III IEF System (GE Healthcare) at 20 °C using a stepwise mode to reach 9,083 volt·hours. After completion of the IEF, the IPG strips were equilibrated in an equilibration buffer containing 6 M urea, 130 mM DTT, 112 mM Tris-base, 4% SDS, 30% glycerol and 0.002% bromophenol blue for 15 min. The strips were then equilibrated for additional 15 min in another equilibration buffer, in which DTT was replaced with 135 mM iodoacetamide. The second dimensional separation was performed in 12.5% polyacrylamide slab gel using SE260 Mini-Vertical Electrophoresis Unit (GE Healthcare) at 150 V for approximately 2 h. The gels were then stained with Deep Purple fluorescence dye (GE Healthcare). All the stained gels were imaged by Typhoon 9200 laser scanner (GE Healthcare).

### Matching and quantitative analysis of protein spots

Spot matching and quantitative intensity analysis were performed using Image Master 2D Platinum software (version 6.0) (GE Healthcare) as described previously^53,54^. Parameters used for spot detection were set as follows: minimal area = 10 pixels; smooth factor = 2.0; saliency = 2.0. A reference gel was created from an artificial gel combining all of the spots presenting in different gels into one image. The reference gel was then used for matching the corresponding protein spots among different gels. Background subtraction was performed and the intensity volume of each spot was normalized with total intensity volume (summation of the intensity volumes obtained from all spots within the same 2-DE gel).

### In-gel tryptic digestion

In-gel tryptic digestion was performed following protocol described previously^55,56^. Briefly, protein spots with significantly differential levels were excised from 2-D gels, washed with 1 ml deionized water, and then destained with 100 µl of 100 mM NH_4_HCO_3_ at 25 °C for 15 min. Thereafter, 100 µl acetonitrile (ACN) was added and incubated at 25 °C for 15 min. After removing the solvent, the gel pieces were dried in a SpeedVac concentrator (Savant; Holbrook, NY) and rehydrated with 50 µl of 10 mM DTT in 100 mM NH_4_HCO_3_ at 56 °C for 30 min using a heat box. After removing the reducing buffer, the gel pieces were incubated with 50 µl of 55 mM iodoacetamide in 100 mM NH_4_HCO_3_ at 25 °C for 20 min in the dark. The buffer was then removed, whereas the gel pieces were incubated with 100 µl of 50 mM NH_4_HCO_3_ at 25 °C for 15 min. Thereafter, 100 µl ACN was added and incubated at 25 °C for 15 min. After removing the solvent, the gel pieces were dried in a SpeedVac concentrator, and then incubated with a minimal volume (just to cover gel pieces) of 12 ng/µl sequencing grade modified trypsin (Promega; Madison, WI) in 50 mM NH_4_HCO_3_ in a ThermoMixer^®^ C (Eppendorf; Hauppauge, NY) at 37 °C for 16–18 h. The digestion reaction was stopped by incubation with 100 µl of 5% formic acid/ACN (1:2 vol/vol) at 37 °C for 15 min. The digested peptide mixtures were collected using a pipette with gel loader tip, transferred into a fresh tube, dried by a SpeedVac concentrator, and subjected to MS/MS analysis.

### Identification of proteins by nanoLC-ESI-ETD MS/MS (nanoscale liquid chromatography - electrospray ionization - electron transfer dissociation tandem mass spectrometry)

Separation of the digested peptides was performed using EASY-nLC II (Bruker Daltonics; Bremen, Germany) as previously described^57,58^. Peptides were loaded from a cooled (7 °C) autosampler into an in-house, 3-cm-long pre-column containing 5-µm C18 resin (Dr. Maisch GmbH; Ammerbuch, Germany) and then to an in-house, 10-cm-long analytical column packed with 3-µm C18 resin (Dr.Maisch GmbH) using mobile phase A (0.1% formic acid). The peptides were then separated by mobile phase B (ACN/0.1% formic acid) gradient elution with three steps as follows: 0–35% for 30 min, 35–80% for 10 min, and then 80% for 10 min at a flow rate of 300 nl/min. Peptide sequences were then analyzed by amaZon speed ETD (Bruker Daltonics) with ESI nanosprayer ion source (spray capillary: fused silica with outer diameter of 90 µm and inner diameter of 20 µm) controlled by HyStar version 3.2 and trapControl version 7.1. Mass spectrometric parameters were set as follows: electrospray voltage = 4,500 V, high-voltage end-plate offset = 500 V, nebulizer gas = 0.55 bar, dry gas = 5.0 l/min, and dry temperature = 150 °C. Precursors were scanned from 400 to 2,200 *m/z* range with enhanced resolution mode (speed = 8,100 *m/z*/s), ICC (Ion Charge Control) target = 200,000, maximal accumulation time = 50 ms. The three most intense signals in every MS scan were selected for MS/MS analysis, whereas singly charged ions were excluded. For MS/MS experiment, fragmented peptides from 150 to 3,000 *m/z* range were scanned with XtremeScan mode (speed = 52,000 *m/z*/sec), ICC target = 200,000, maximal accumulation time = 100 ms. Mass spectra were deconvoluted via DataAnalysis version 4.0 SP5 (BrukerDaltonics) to*.mgf* file. Mascot software version 2.4.0 (Matrix Science; London, UK) was used to search MS/MS spectra against NCBI database of mammalian with the following standard Mascot parameters for CID: Enzyme = trypsin, maximal number of missed cleavages = 1, peptide tolerance = ±1.2 Da, MS/MS tolerance = ±0.6 Da, fixed modification = carbamidomethyl (C), variable modification = oxidation (M), charge states = 2+ and 3+, and instrument type = ESI-Trap^59,60^.

### Analysis for protein post-translational modifications (PTMs)

All the experimental peptide masses of each identified protein obtained from MS/MS analysis as described above were subjected to analysis for potential PTMs using FindMod tool (http://web.expasy.org/findmod/). Types of PTMs and numbers of the modified amino acid residues found in each identified protein are then summarized.

### Mass spectrometric analyses of phosphorylated and oxidatively modified proteins

MS/MS spectra obtained from nanoLC-ESI-ETD MS/MS as described above were submitted to the Mascot search engine (Matrix Science) to query against the NCBI mammalian protein database using the following PTMs parameters: Enzyme = trypsin, maximal number of missed cleavages = 1, peptide tolerance = ±1.2 Da, MS/MS tolerance = ±0.6 Da, fixed modification = carbamidomethyl (C), variable modification = phosphorylation (S, Y and T) for phosphorylated peptides or oxidation (M and C), dioxidation (M and C) and trioxidation (C) for oxidatively modified peptides, charge states = 2+ and 3+, and instrument type = ESI-Trap. All of the identified peptides that contained at less 1 modified residue were counted and reported as the phosphorylated or oxidatively modified peptides.

### Western blotting

Whole cells and isolated mitochondria derived from each sample were lyzed with Laemmli’s buffer. Protein concentrations in individual samples were measured by Bradford’s method using Bio-Rad protein assay (Bio-Rad Laboratories). An equal amount of total protein (20 µg/each sample) was resolved in each lane of 12% SDS-PAGE using SE260 mini-Vertical Electrophoresis Unit (GE Healthcare) at 150 V for approximately 2 h. The resolved proteins were then transferred onto a nitrocellulose membrane using a semi-dry transfer apparatus (GE Healthcare) at 85 mA for 1.5 h. The membrane was subsequently stained with Ponceau S dye (Sigma) to verify equal protein loading and to serve as the loading control for subsequent quantitative intensity analysis. Thereafter, the membrane was destained with PBS and non-specific bindings were blocked with 5% skim milk in PBS at 25 °C for 1 h. The membrane was incubated with mouse monoclonal anti-COX4 (Santa Cruz Biotechnology; Santa Cruz, CA), rabbit polyclonal anti-carbonic anhydrase II (Chemicon; Temecula, CA), rabbit polyclonal anti-GRP94 (Santa Cruz Biotechnology), mouse monoclonal anti-tubulin (Santa Cruz Biotechnology), mouse monoclonal anti-lamin A/C (Santa Cruz Biotechnology), mouse monoclonal anti-annexin A2, mouse monoclonal anti-ezrin (Santa Cruz Biotechnology), or rabbit polyclonal anti-p-ezrin (Thr567) (Santa Cruz Biotechnology) antibody (all were diluted 1:1,000 in 1% skim milk/PBS) at 4 °C overnight. After washing with PBS three times, the membrane was incubated with corresponding secondary antibody conjugated with horseradish peroxidase (1:2,000 in 1% skim milk/PBS; DAKO Glostrup, Denmark) at 25 °C for 1 h. Immunoreactive bands were developed by SuperSignal West Pico chemiluminescence substrate (Pierce Biotechnology; Rockford, IL) and were then visualized by autoradiogram. Band intensity data was obtained using ImageQuant TL software (GE Healthcare).

### Detection and quantitative analysis of mitochondrial phosphoproteins

For detection of phosphoproteins, mitochondrial proteins were resolved by 2-DE using the identical protocol as for comparative 2-DE analysis described above. The gels were then subjected to dual stainings, i.e., first with Pro-Q Diamond phosphoprotein gel dye (Invitrogen – Molecular Probes; Eugene, OR) followed by SYPRO Ruby total protein gel stain (n = 3 gels were derived from 3 individual culture flasks for each condition; a total of 9 gels were subjected to phosphoprotein analysis). All the stained gels were imaged by Typhoon 9200 laser scanner (GE Healthcare). Quantitative analysis of phosphoproteins was performed using Image Master 2D Platinum software (version 6.0) (GE Healthcare).

### Oxyblot assay

To verify the mitochondrial ROS dysregulation, immunoblot detection of oxidatively modified proteins was performed using OxyBlot^TM^ Protein Oxidation Detection Kit (S7150) (Chemicon) as described previously^61,62^. Briefly, mitochondrial proteins were derivatized with or without 2, 4-dinitrophenylhydrazine (DNPH). The derivatized and non-derivatized mitochondrial proteins were resolved by SDS-PAGE and then transferred onto a nitrocellulose membrane. Thereafter, the membrane was incubated with primary antibody against DNP moiety of the oxidatively modified proteins at 25 °C for 1 h, and then with corresponding secondary antibody conjugated with HRP at 25 °C for 1 h. Finally, the oxidatively modified proteins were detected by SuperSignal^®^ West Pico chemiluminescence substrate (Pierce Biotechnology, Inc.). Band intensity data was obtained using ImageQuant TL software (GE Healthcare).

### Measurement of intracellular ROS level

Intracellular ROS level was measured by dichlorodihydrofluorescein diacetate (DCFH-DA) assay using flow cytometry as described previously^63,64^. Briefly, the cells were washed with PBS and detached by trypsinization. The cell suspension was incubated with 5 μM DCFH-DA at 37 °C for 20 min in the dark and then subjected to flow cytometry. At least 10,000 events for each sample were acquired on BD Accuri^TM^ C6 flow cytometer (Beckman Coulter; Fullerton, CA) and analyzed by FlowJo^TM^ software (version 10.5.3) (FlowJo, LLC; Ashland, OR).

### Measurement of intracellular ATP level

Intracellular ATP level was measured by luciferin-luciferase ATP assay as described previously^65,66^. Briefly, the cells were incubated with ATP extraction buffer (25 mM Tricine, 100 µM EDTA, 1 mM DTT, and 1% Triton X-100) at 4 °C for 5 min and cell debris was removed by centrifugation at 1,000 *g* and 4 °C for 5 min. The clarified supernatant was collected and then incubated with a reaction solution (25 mM Tricine, 0.5 mM D-luciferin, 1.25 µg/ml luciferase, 5 mM MgSO_4_, 100 µM EDTA, and 1 mM DTT). Bioluminescence was measured using a BioTek Synergy H1 Hybrid Multi-Mode microplate reader (BioTek Instruments; Winooski, VT). ATP concentration in the sample was calculated from a standard curve and normalized by protein concentration. The intracellular ATP content of each sample is reported as pmol/mg protein.

### Statistical analysis

All quantitative data are presented as mean ± SEM. Comparisons among groups were performed by one-way ANOVA using PASW Statistics 18 software (SPSS; Chicago, IL). *P* values less than 0.05 were considered statistically significant.

## Supplementary information

Supplementary information
