# Supplementary material for: High glucose induces phosphorylation and oxidation of mitochondrial proteins in renal tubular cells: A proteomics approach
Source: Sci Rep. 2020 Apr 3;10:5843. doi: 10.1038/s41598-020-62665-w (PMC7125224; doi:10.1038/s41598-020-62665-w)
Supplement: Supplementary file 1 — Supplementary information [file 41598_2020_62665_MOESM1_ESM.pdf]

SUPPORTING INFORMATION

High glucose induces phosphorylation and oxidation of mitochondrial proteins in renal tubular cells: A proteomics approach

*Siripat Aluksanasuwan, Sirikanya Plumworasawat, Thanyalak Malaitad, Sakdithep Chaiyarit, and Visith Thongboonkerd**
E-mail: thongboonkerd@dr.com (or) vthongbo@yahoo.com

Supplementary Figure S1: Full-length blots of the cropped images shown in **Figure 1**. The cropped areas are labeled with red-dotted boxes.

Supplementary Figure S2: MS/MS spectra of all individual peptides assigned to each identified protein.

Supplementary Figure S3: Full-length blots of the cropped images shown in **Figure 4A**. The cropped areas are labeled with red-dotted boxes.

Supplementary Figure S4: Whole-gel images of the cropped (zoom-in) areas shown in **Figure 6A**. The cropped areas are labeled with red-dotted boxes.

Supplementary Figure S5: Illustrative MS/MS spectra of phosphorylated peptide identified from each protein spot.

Supplementary Figure S6: Illustrative MS/MS spectra of oxidatively modified peptide identified from each protein spot.

Supplementary Table S1: Summary of all distinct peptides matched in each identified protein.

Supplementary Table S2: Summary of potential post-translational modifications (PTMs) of all the identified proteins.

Figure S1

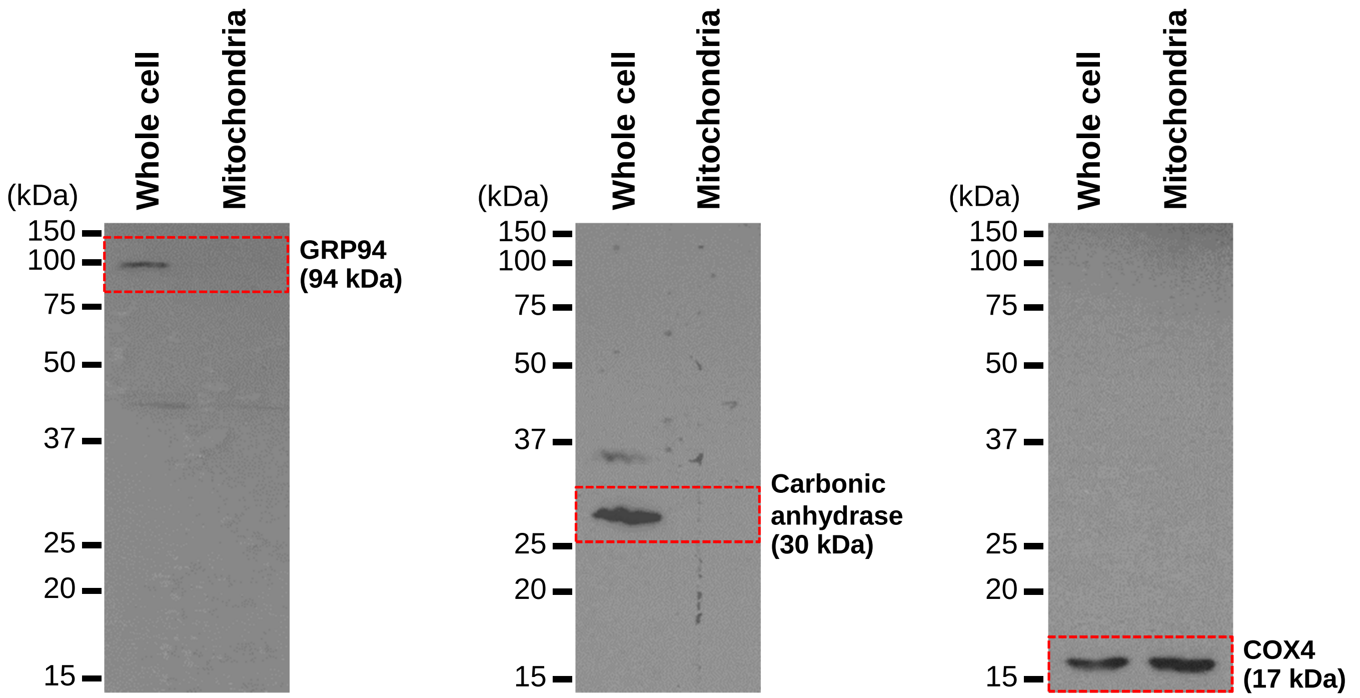

Supplementary Figure S1: Full-length blots of the cropped images shown in **Figure 1**. The cropped areas are labeled with red-dotted boxes.

Supplementary Figure S2: MS/MS spectra of all individual peptides assigned to each identified protein.

Spot no.181
Tubulin beta-2A chain
AILVDLEPGTMDSVR

#	b	b^{++}	b^0	b^{0++}	Seq.	y	y^{++}	y*	y^{*++}	y^0	y^{0++}	#
1	72.0444	36.5258			A							15
2	185.1285	93.0679			I	1560.7938	780.9005	1543.7672	772.3873	1542.7832	771.8952	14
3	**298.2125**	149.6099			L	1447.7097	724.3585	1430.6832	715.8452	1429.6992	715.3532	13
4	**397.2809**	199.1441			V	***1334.6257***	667.8165	1317.5991	659.3032	1316.6151	658.8112	12
5	**512.3079**	256.6576	494.2973	247.6523	D	***1235.5572***	618.2823	1218.5307	609.769	1217.5467	609.277	11
6	**625.3919**	313.1996	607.3814	304.1943	L	***1120.5303***	560.7688	1103.5038	552.2555	1102.5197	551.7635	10
7	**754.4345**	377.7209	736.424	368.7156	E	***1007.4462***	504.2268	990.4197	495.7135	989.4357	495.2215	9
8	851.4873	426.2473	833.4767	417.242	P	***878.4036***	439.7055	861.3771	431.1922	860.3931	430.7002	8
9	**908.5088**	454.758	890.4982	445.7527	G	***781.3509***	391.1791	764.3243	382.6658	763.3403	382.1738	7
10	1009.5564	505.2819	991.5459	496.2766	T	***724.3294***	362.6683	707.3029	354.1551	706.3189	353.6631	6
11	**1156.5918**	578.7996	1138.5813	569.7943	M	***623.2817***	312.1445	606.2552	303.6312	605.2712	303.1392	5
12	**1271.6188**	636.313	1253.6082	627.3077	D	***476.2463***	238.6268	459.2198	230.1135	458.2358	229.6215	4
13	1358.6508	679.829	1340.6402	670.8238	S	***361.2194***	181.1133	344.1928	172.6001	343.2088	172.1081	3
14	1457.7192	729.3633	1439.7087	720.358	V	274.1874	137.5973	257.1608	129.084			2
15					R	175.119	88.0631	158.0924	79.5498			1

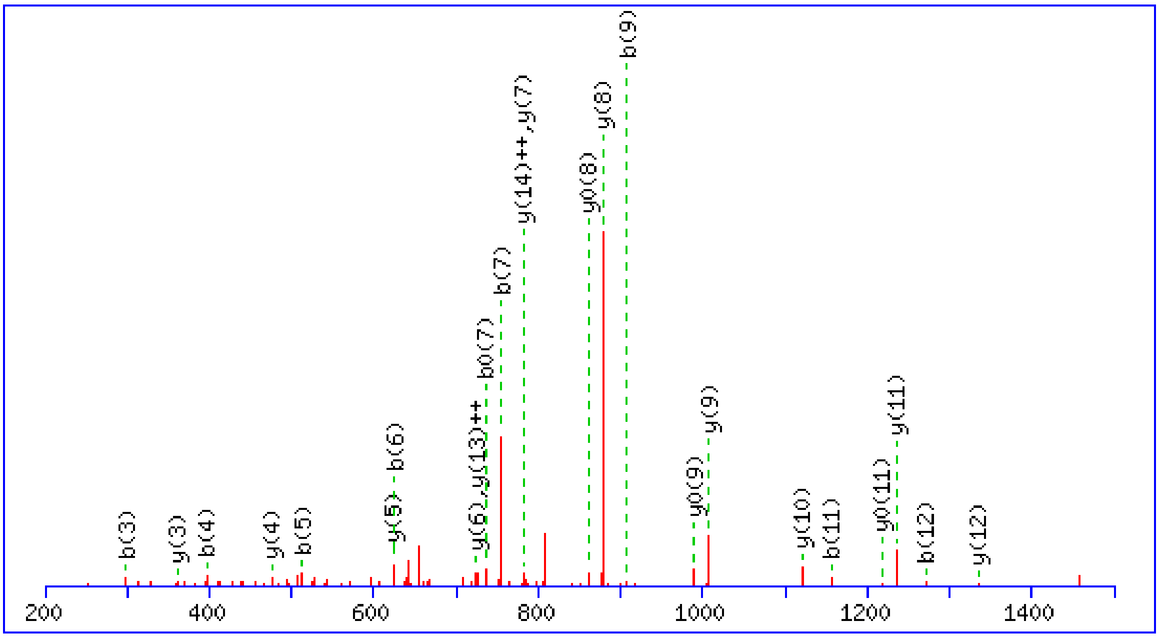

Spot no.181
Tubulin beta-2A chain
IREEYPDR

#	b	b++	b*	b*++	b0	b0++	Seq.	y	y++	y*	y*++	y0	y0++	#
1	114.0913	57.5493					I							8
2	*270.1925*	135.5999	253.1659	127.0866			R	964.4483	482.7278	947.4217	474.2145	946.4377	473.7225	7
3	*399.235*	200.1212	382.2085	191.6079	381.2245	191.1159	E	**808.3472**	404.6772	791.3206	396.164	790.3366	395.6719	6
4	*528.2776*	264.6425	511.2511	256.1292	510.2671	255.6372	E	679.3046	340.1559	662.278	331.6427	661.294	331.1506	5
5	*691.341*	346.1741	674.3144	337.6608	673.3304	337.1688	Y	550.262	275.6346	533.2354	267.1214	532.2514	266.6293	4
6	788.3937	394.7005	771.3672	386.1872	770.3832	385.6952	P	387.1987	194.103	370.1721	185.5897	369.1881	185.0977	3
7	*903.4207*	452.214	886.3941	443.7007	885.4101	443.2087	D	290.1459	145.5766	273.1193	137.0633	272.1353	136.5713	2
8							R	**175.119**	88.0631	158.0924	79.5498			1

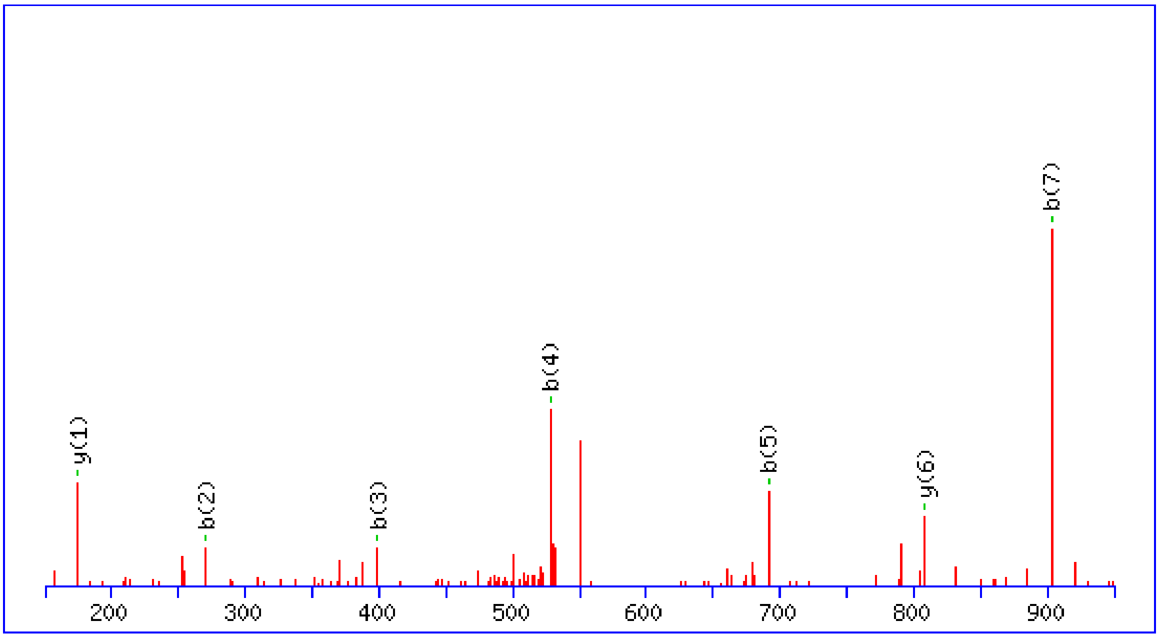

Spot no.181
Tubulin beta-2A chain
IMNTFSVMPSPK

#	b	b^{++}	b*	b^{*++}	b^{0}	b^{0++}	Seq.	y	y^{++}	y*	y^{*++}	y^{0}	y^{0++}	#
1	114.0913	57.5493					I							12
2	**261.1267**	131.067					M	1254.586	627.7965	1237.559	619.2832	1236.575	618.7912	11
3	375.1697	188.0885	358.1431	179.5752			N	***1107.55***	554.2788	1090.524	545.7655	1089.54	545.2735	10
4	**476.2173**	238.6123	459.1908	230.099	458.2068	229.607	T	***993.5074***	497.2573	976.4808	488.7441	975.4968	488.252	9
5	623.2858	312.1465	606.2592	303.6332	605.2752	303.1412	F	***892.4597***	446.7335	875.4332	438.2202	874.4491	437.7282	8
6	710.3178	355.6625	693.2912	347.1493	692.3072	346.6572	S	***745.3913***	373.1993	728.3647	364.686	727.3807	364.194	7
7	809.3862	405.1967	792.3597	396.6835	791.3756	396.1915	V	658.3593	329.6833	641.3327	321.17	640.3487	320.678	6
8	940.4267	470.717	923.4001	462.2037	922.4161	461.7117	M	***559.2908***	280.1491	542.2643	271.6358	541.2803	271.1438	5
9	1037.48	519.2434	1020.453	510.7301	1019.469	510.2381	P	***428.2504***	214.6288	411.2238	206.1155	410.2398	205.6235	4
10	1124.512	562.7594	1107.485	554.2461	1106.501	553.7541	S	331.1976	166.1024	314.171	157.5892	313.187	157.0972	3
11	1221.564	611.2858	1204.538	602.7725	1203.554	602.2805	P	***244.1656***	122.5864	227.139	114.0731			2
12							K	147.1128	74.06	130.0863	65.5468			1

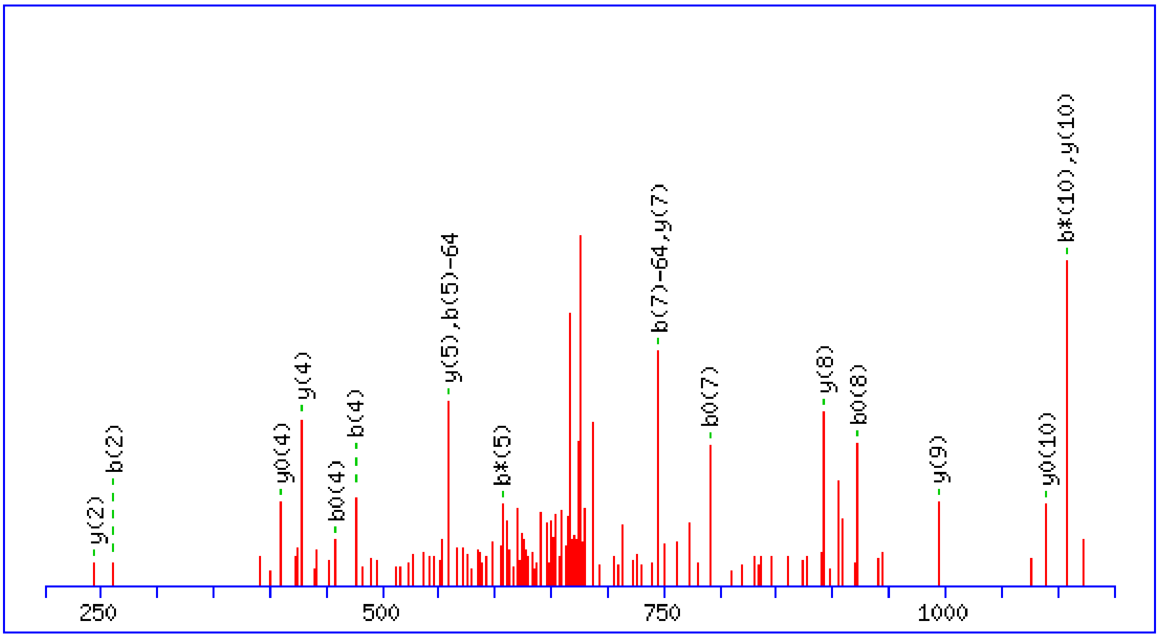

Spot no.181
Tubulin beta-2A chain
FPGQLNADLR

#	b	b^{++}	b*	b^{*++}	b^{0}	b^{0++}	Seq.	y	y^{++}	y*	y^{*++}	y^{0}	y^{0++}	#
1	148.0757	74.5415					F							10
2	**245.1285**	123.0679					P	***983.5269***	492.2671	966.5003	483.7538	965.5163	483.2618	9
3	302.1499	151.5786					G	***886.4741***	443.7407	869.4476	435.2274	868.4635	434.7354	8
4	**430.2085**	215.6079	413.1819	207.0946			Q	829.4526	415.23	812.4261	406.7167	811.4421	406.2247	7
5	**543.2926**	272.1499	526.266	263.6366			L	***701.3941***	351.2007	684.3675	342.6874	683.3835	342.1954	6
6	657.3355	329.1714	640.3089	320.6581			N	***588.31***	294.6586	571.2835	286.1454	570.2994	285.6534	5
7	728.3726	364.6899	711.3461	356.1767			A	***474.2671***	237.6372	457.2405	229.1239	456.2565	228.6319	4
8	**843.3995**	422.2034	826.373	413.6901	825.389	413.1981	D	***403.23***	202.1186	386.2034	193.6053	385.2194	193.1133	3
9	956.4836	478.7454	939.4571	470.2322	938.473	469.7402	L	***288.203***	144.6051	271.1765	136.0919			2
10							R	***175.119***	88.0631	158.0924	79.5498			1

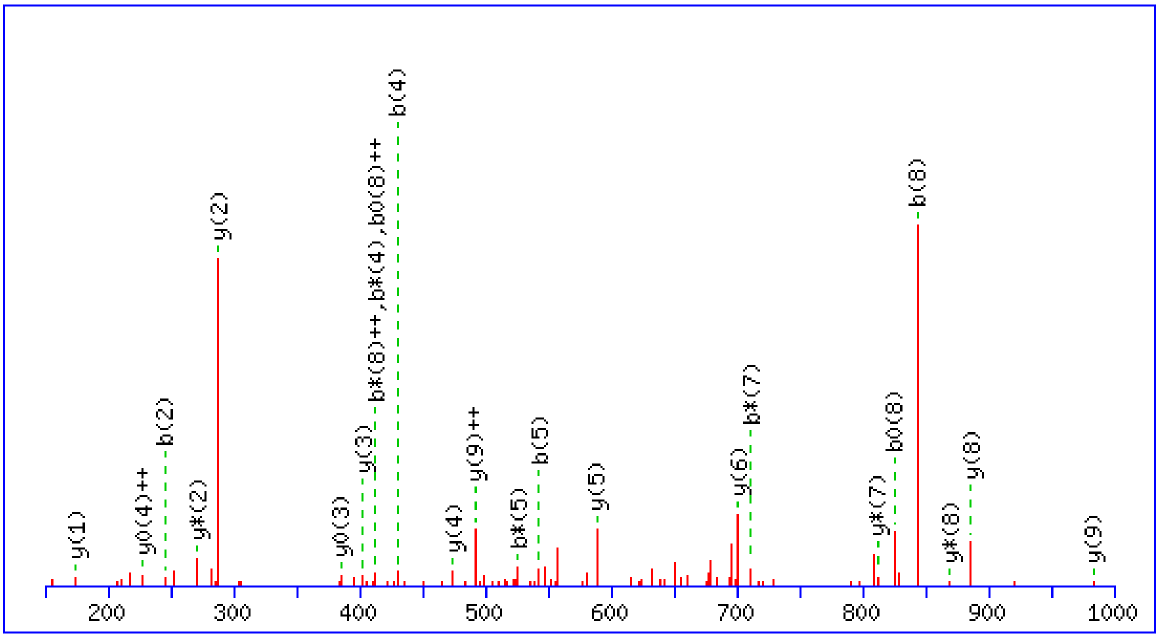

Spot no.181
Tubulin beta-2A chain
LAVNMVPFPR

#	b	b^{++}	b*	b^{*++}	Seq.	y	y^{++}	y*	y^{*++}	#
**1**	114.0913	57.5493			**L**					**10**
**2**	**185.1285**	93.0679			**A**	***1046.5452***	523.7762	1029.5186	515.2629	**9**
**3**	284.1969	142.6021			**V**	***975.508***	488.2577	958.4815	479.7444	**8**
**4**	398.2398	199.6235	381.2132	191.1103	**N**	***876.4396***	438.7235	859.4131	430.2102	**7**
**5**	545.2752	273.1412	528.2486	264.628	**M**	***762.3967***	381.702	745.3702	373.1887	**6**
**6**	**644.3436**	322.6754	627.3171	314.1622	**V**	***615.3613***	308.1843	598.3348	299.671	**5**
**7**	**741.3964**	371.2018	724.3698	362.6886	**P**	***516.2929***	258.6501	499.2663	250.1368	**4**
**8**	**888.4648**	444.736	871.4382	436.2228	**F**	***419.2401***	210.1237	402.2136	201.6104	**3**
**9**	985.5176	493.2624	968.491	484.7491	**P**	***272.1717***	136.5895	255.1452	128.0762	**2**
**10**					**R**	***175.119***	88.0631	158.0924	79.5498	**1**

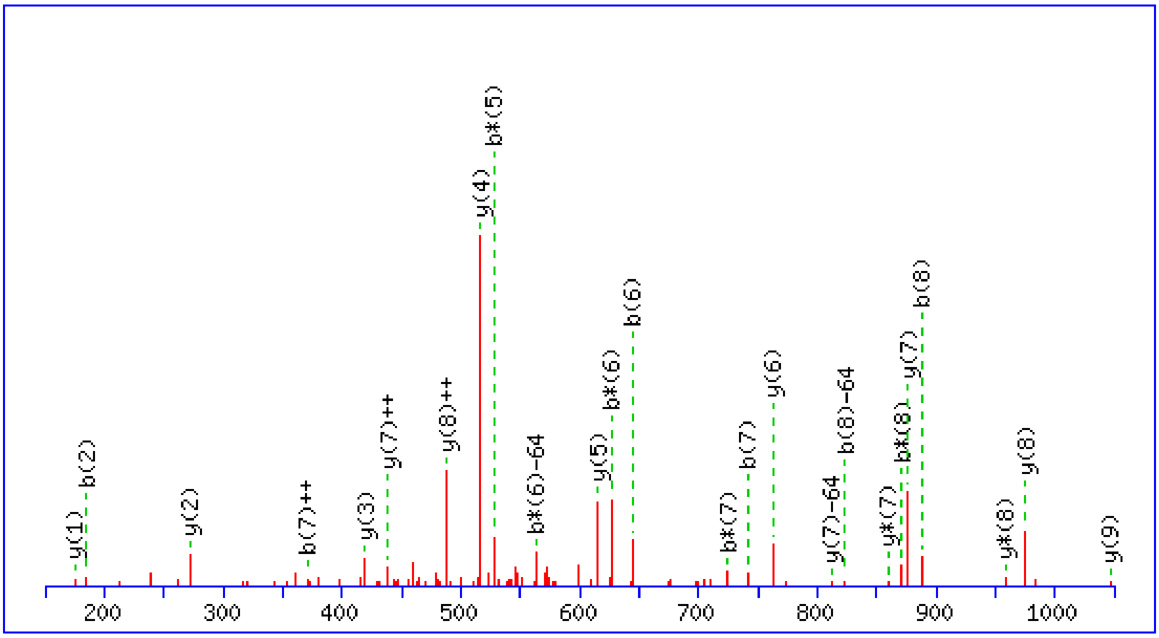

Spot no.181
Tubulin beta-2A chain
LHFFMPGFAPLTSR

#	b	b++	b0	b0++	Seq.	y	y++	y*	y*++	y0	y0++	#
1	114.0913	57.5493			L							14
2	***251.1503***	126.0788			H	1507.7515	754.3794	1490.7249	745.8661	1489.7409	745.3741	13
3	***398.2187***	199.613			F	***1370.6926***	685.8499	1353.666	677.3366	1352.682	676.8446	12
4	***545.2871***	273.1472			F	***1223.6241***	612.3157	1206.5976	603.8024	1205.6136	603.3104	11
5	***676.3276***	338.6674			M	***1076.5557***	538.7815	1059.5292	530.2682	1058.5452	529.7762	10
6	773.3803	387.1938			P	***945.5152***	473.2613	928.4887	464.748	927.5047	464.256	9
7	830.4018	415.7045			G	848.4625	424.7349	831.4359	416.2216	830.4519	415.7296	8
8	977.4702	489.2387			F	791.441	396.2241	774.4145	387.7109	773.4305	387.2189	7
9	***1048.5073***	524.7573			A	644.3726	322.6899	627.3461	314.1767	626.362	313.6847	6
10	1145.5601	573.2837			P	***573.3355***	287.1714	556.3089	278.6581	555.3249	278.1661	5
11	1258.6441	629.8257			L	476.2827	238.645	459.2562	230.1317	458.2722	229.6397	4
12	1359.6918	680.3496	1341.6813	671.3443	T	363.1987	182.103	346.1721	173.5897	345.1881	173.0977	3
13	1446.7239	723.8656	1428.7133	714.8603	S	262.151	131.5791	245.1244	123.0659	244.1404	122.5738	2
14					R	175.119	88.0631	158.0924	79.5498			1

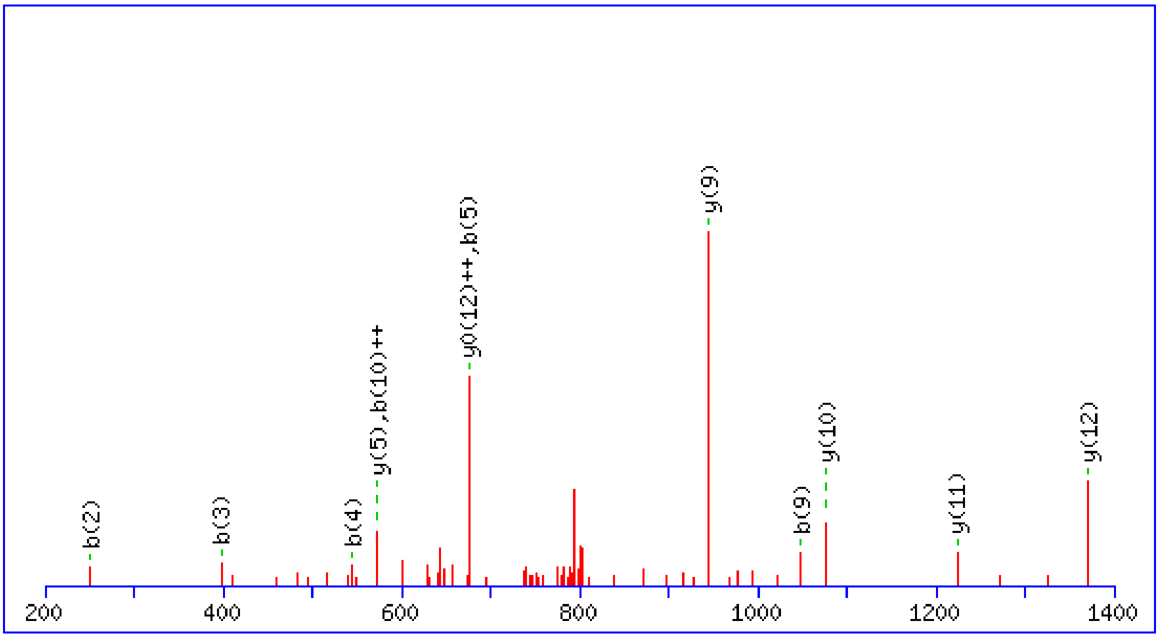

Spot no.181
Tubulin beta-2A chain
ALTVPELTQQMFDSK

#	b	b++	b*	b*++	b0	b0++	Seq.	y	y++	y*	y*++	y0	y0++	#
1	72.0444	36.5258					A							15
2	185.1285	93.0679					L	1652.82	826.9136	1635.7935	818.4004	1634.8094	817.9084	14
3	286.1761	143.5917			268.1656	134.5864	T	1539.7359	770.3716	1522.7094	761.8583	1521.7254	761.3663	13
4	**385.2445**	193.1259			367.234	184.1206	V	***1438.6883***	719.8478	1421.6617	711.3345	1420.6777	710.8425	12
5	482.2973	241.6523			464.2867	232.647	P	***1339.6198***	670.3136	1322.5933	661.8003	1321.6093	661.3083	11
6	611.3399	306.1736			593.3293	297.1683	E	***1242.5671***	621.7872	1225.5405	613.2739	1224.5565	612.7819	10
7	724.424	362.7156			706.4134	353.7103	L	***1113.5245***	557.2659	1096.4979	548.7526	1095.5139	548.2606	9
8	825.4716	413.2395			807.4611	404.2342	T	***1000.4404***	500.7239	983.4139	492.2106	982.4299	491.7186	8
9	953.5302	477.2688	936.5037	468.7555	935.5197	468.2635	Q	***899.3927***	450.2	882.3662	441.6867	881.3822	441.1947	7
10	**1081.5888**	541.298	1064.5623	532.7848	1063.5782	532.2928	Q	771.3342	386.1707	754.3076	377.6574	753.3236	377.1654	6
11	**1228.6242**	614.8157	1211.5977	606.3025	1210.6136	605.8105	M	***643.2756***	322.1414	626.249	313.6282	625.265	313.1362	5
12	**1375.6926**	688.3499	1358.6661	679.8367	1357.6821	679.3447	F	***496.2402***	248.6237	479.2136	240.1105	478.2296	239.6185	4
13	**1490.7196**	745.8634	1473.693	737.3501	1472.709	736.8581	D	***349.1718***	175.0895	332.1452	166.5763	331.1612	166.0842	3
14	1577.7516	789.3794	1560.725	780.8662	1559.741	780.3741	S	234.1448	117.5761	217.1183	109.0628	216.1343	108.5708	2
15							K	147.1128	74.06	130.0863	65.5468			1

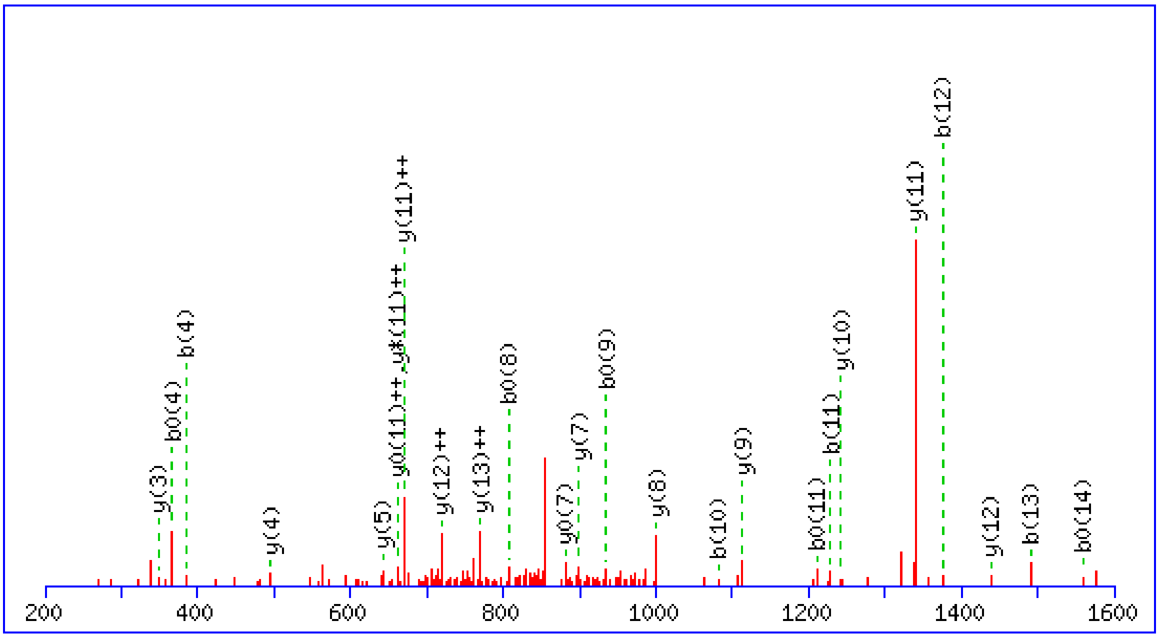

Spot no.181
Tubulin beta-2A chain
YLTVAAIFR

#	b	b++	b0	b0++	Seq.	y	y++	y*	y*++	y0	y0++	#
1	164.0706	82.5389			Y							9
2	**277.1547**	139.081			L	890.5458	445.7765	873.5193	437.2633	872.5352	436.7713	8
3	**378.2023**	189.6048	360.1918	180.5995	T	***777.4618***	389.2345	760.4352	380.7212	759.4512	380.2292	7
4	477.2708	239.139	459.2602	230.1337	V	***676.4141***	338.7107	659.3875	330.1974			6
5	548.3079	274.6576	530.2973	265.6523	A	***577.3457***	289.1765	560.3191	280.6632			5
6	**619.345**	310.1761	601.3344	301.1709	A	***506.3085***	253.6579	489.282	245.1446			4
7	**732.4291**	366.7182	714.4185	357.7129	I	***435.2714***	218.1394	418.2449	209.6261			3
8	879.4975	440.2524	861.4869	431.2471	F	***322.1874***	161.5973	305.1608	153.084			2
9					R	175.119	88.0631	158.0924	79.5498			1

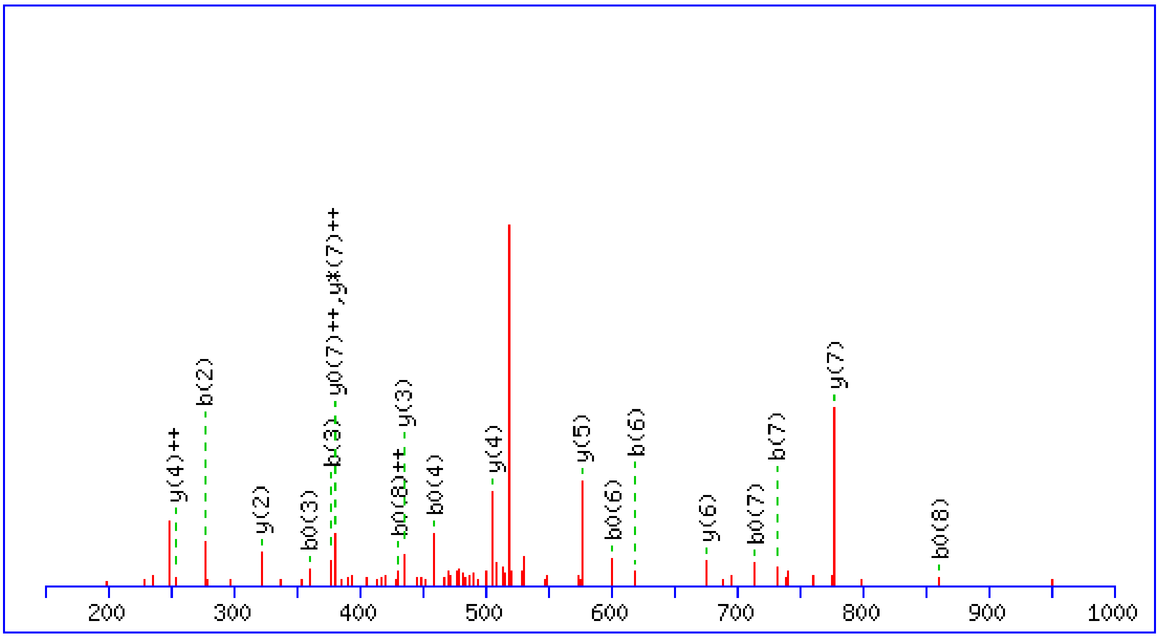

Spot no.181
Tubulin beta-2A chain
MSMKEVDEQMLNVQNK

#	b	b++	b*	b*++	b0	b0++	Seq.	y	y++	y*	y*++	y0	y0++	#
1	148.0427	74.525					M							16
2	235.0747	118.041			217.0641	109.0357	S	1824.8466	912.927	1807.8201	904.4137	1806.8361	903.9217	15
3	***382.1101***	191.5587			364.0995	182.5534	M	1737.8146	869.4109	1720.788	860.8977	1719.804	860.4057	14
4	***510.2051***	255.6062	493.1785	247.0929	492.1945	246.6009	K	1590.7792	795.8932	1573.7526	787.38	1572.7686	786.888	13
5	***639.2477***	320.1275	622.2211	311.6142	621.2371	311.1222	E	1462.6842	731.8458	1445.6577	723.3325	1444.6737	722.8405	12
6	***738.3161***	369.6617	721.2895	361.1484	720.3055	360.6564	V	1333.6416	667.3245	1316.6151	658.8112	1315.6311	658.3192	11
7	***853.343***	427.1751	836.3165	418.6619	835.3325	418.1699	D	1234.5732	617.7903	1217.5467	609.277	1216.5627	608.785	10
8	***982.3856***	***491.6964***	965.3591	483.1832	964.375	482.6912	E	1119.5463	560.2768	1102.5197	551.7635	1101.5357	551.2715	9
9	1110.4442	555.7257	1093.4176	547.2125	1092.4336	546.7204	Q	990.5037	495.7555	973.4771	487.2422			8
10	1257.4796	***629.2434***	1240.453	620.7302	1239.469	620.2381	M	**862.4451**	431.7262	845.4186	423.2129			7
11	1370.5637	***685.7855***	1353.5371	677.2722	1352.5531	676.7802	L	**715.4097**	358.2085	698.3832	349.6952			6
12	1484.6066	***742.8069***	1467.58	734.2937	1466.596	733.8016	N	**602.3257**	301.6665	585.2991	293.1532			5
13	1583.675	***792.3411***	1566.6484	783.8279	1565.6644	783.3359	V	**488.2827**	244.645	471.2562	236.1317			4
14	1711.7336	***856.3704***	1694.707	847.8571	1693.723	847.3651	Q	**389.2143**	195.1108	372.1878	186.5975			3
15	1825.7765	***913.3919***	1808.7499	904.8786	1807.7659	904.3866	N	**261.1557**	131.0815	244.1292	122.5682			2
16							K	147.1128	74.06	130.0863	65.5468			1

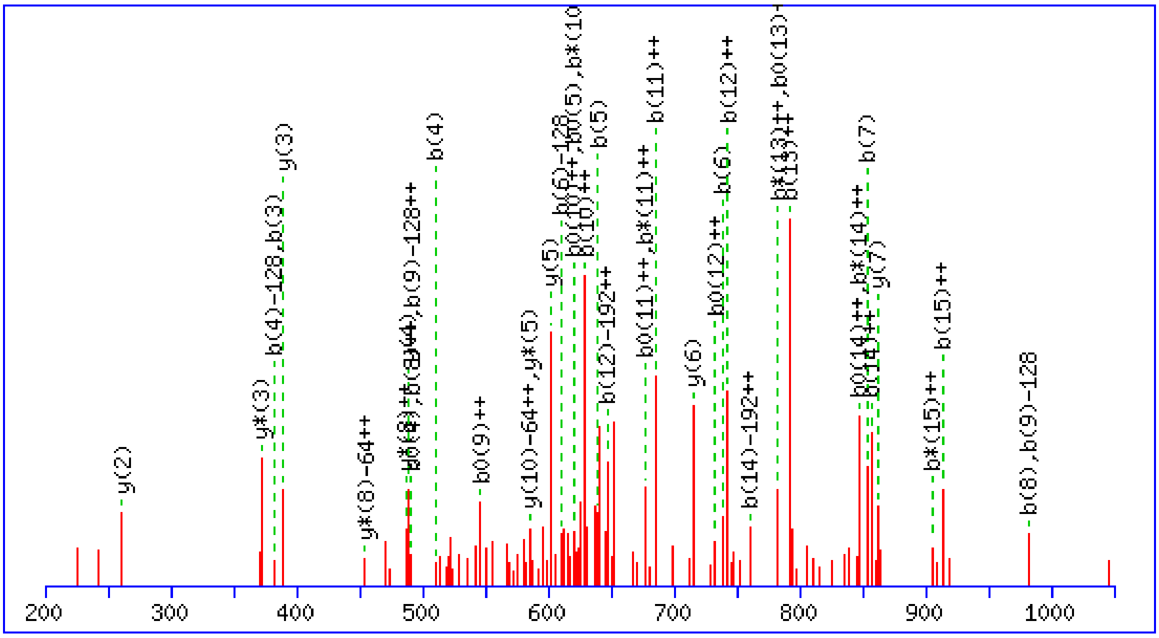

Spot no.181
Tubulin beta-2A chain
EVDEQMLNVQNK

#	b	b++	b*	b*++	b0	b0++	Seq.	y	y++	y*	y*++	y0	y0++	#
1	130.0499	65.5286			112.0393	56.5233	E							12
2	229.1183	115.0628			211.1077	106.0575	V	1333.6416	667.3245	1316.6151	658.8112	1315.6311	658.3192	11
3	344.1452	172.5763			326.1347	163.571	D	***1234.5732***	617.7903	1217.5467	609.277	1216.5627	608.785	10
4	473.1878	237.0975			455.1773	228.0923	E	***1119.5463***	560.2768	1102.5197	551.7635	1101.5357	551.2715	9
5	601.2464	301.1268	584.2198	292.6136	583.2358	292.1216	Q	***990.5037***	495.7555	973.4771	487.2422			8
6	748.2818	374.6445	731.2552	366.1313	730.2712	365.6393	M	862.4451	431.7262	845.4186	423.2129			7
7	**861.3659**	431.1866	844.3393	422.6733	843.3553	422.1813	L	***715.4097***	358.2085	698.3832	349.6952			6
8	975.4088	**488.208**	958.3822	479.6948	957.3982	479.2027	N	***602.3256***	301.6665	585.2991	293.1532			5
9	**1074.4772**	537.7422	1057.4507	529.229	1056.4666	528.737	V	***488.2827***	244.645	471.2562	236.1317			4
10	**1202.5358**	**601.7715**	1185.5092	593.2583	1184.5252	592.7662	Q	***389.2143***	195.1108	372.1878	186.5975			3
11	**1316.5787**	658.793	1299.5522	650.2797	1298.5681	649.7877	N	***261.1557***	131.0815	244.1292	122.5682			2
12							K	147.1128	74.06	130.0863	65.5468			1

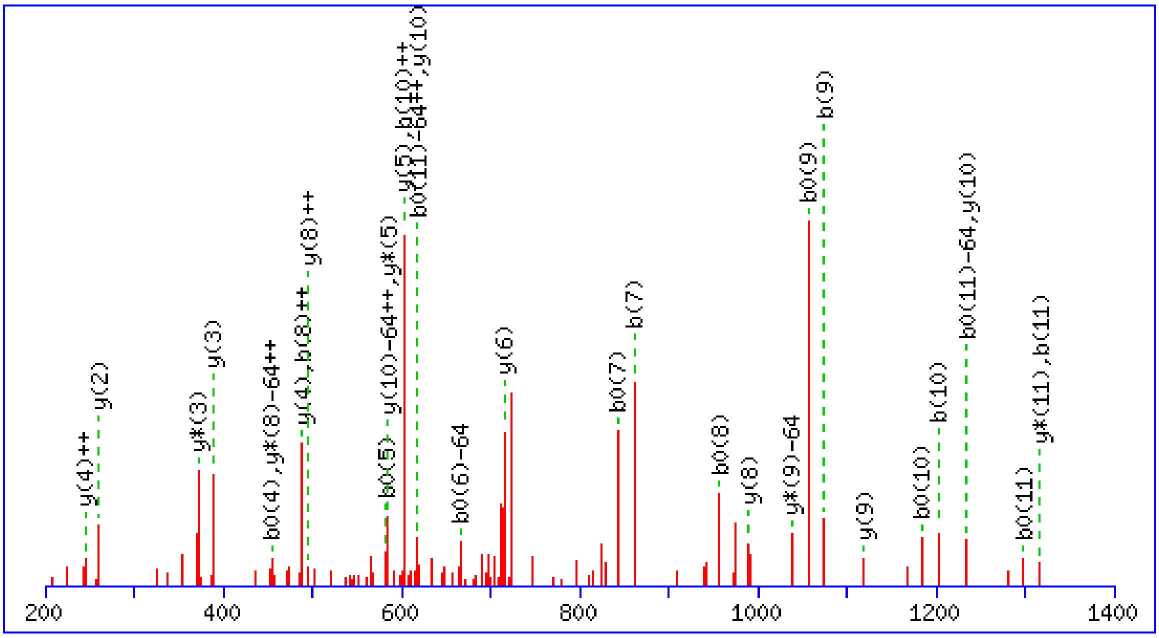

Spot no.181
Tubulin beta-2A chain
NSSYFVEWIPNNVK

#	b	b^{++}	b*	b^{*++}	b^{0}	b^{0++}	Seq.	y	y^{++}	y*	y^{*++}	y^{0}	y^{0++}	#
1	115.0502	58.0287	98.0237	49.5155			N							14
2	202.0822	101.5448	185.0557	93.0315	184.0717	92.5395	S	1582.79	791.8986	1565.7635	783.3854	1564.7795	782.8934	13
3	289.1143	145.0608	272.0877	136.5475	271.1037	136.0555	S	**1495.758**	748.3826	1478.7314	739.8694	1477.7474	739.3774	12
4	452.1776	226.5924	435.151	218.0792	434.167	217.5871	Y	**1408.726**	704.8666	1391.6994	696.3533	1390.7154	695.8613	11
5	599.246	300.1266	582.2195	291.6134	581.2354	291.1214	F	**1245.6626**	623.335	1228.6361	614.8217	1227.6521	614.3297	10
6	**698.3144**	349.6608	681.2879	341.1476	680.3039	340.6556	V	1098.5942	549.8007	1081.5677	541.2875	1080.5837	540.7955	9
7	827.357	414.1821	810.3305	405.6689	809.3464	405.1769	E	**999.5258**	500.2665	982.4993	491.7533	981.5152	491.2613	8
8	1013.4363	507.2218	996.4098	498.7085	995.4258	498.2165	W	**870.4832**	435.7452	853.4567	427.232			7
9	**1126.5204**	563.7638	1109.4938	555.2506	1108.5098	554.7585	I	**684.4039**	342.7056	667.3774	334.1923			6
10	**1223.5731**	612.2902	1206.5466	603.7769	1205.5626	603.2849	P	**571.3198**	286.1636	554.2933	277.6503			5
11	1337.6161	669.3117	1320.5895	660.7984	1319.6055	660.3064	N	474.2671	237.6372	457.2405	229.1239			4
12	**1451.659**	726.3331	1434.6325	717.8199	1433.6484	717.3279	N	**360.2241**	180.6157	343.1976	172.1024			3
13	**1550.7274**	775.8673	1533.7009	767.3541	1532.7169	766.8621	V	246.1812	123.5942	229.1547	115.081			2
14							K	147.1128	74.06	130.0863	65.5468			1

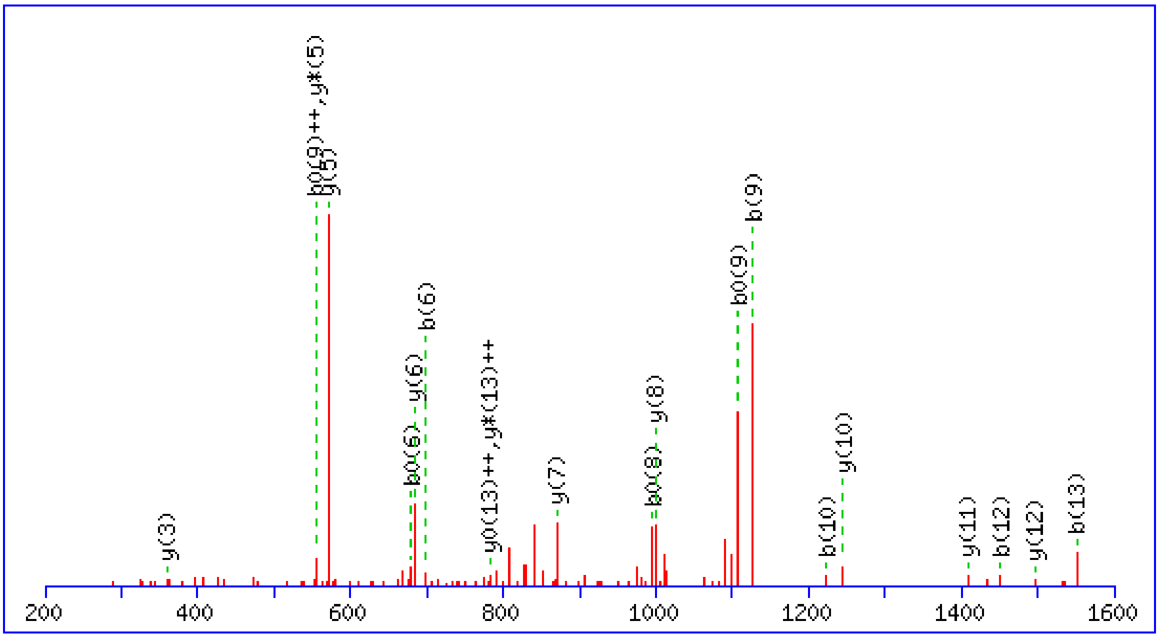

Spot no.181
Tubulin beta-2A chain
TAVCDIPPR

#	b	b++	b0	b0++	Seq.	y	y++	y*	y*++	y0	y0++	#
1	102.055	51.5311	84.0444	42.5258	T							9
2	173.0921	87.0497	155.0815	78.0444	A	927.4717	464.2395	910.4451	455.7262	909.4611	455.2342	8
3	272.1605	136.5839	254.1499	127.5786	V	856.4346	428.7209	839.408	420.2076	838.424	419.7156	7
4	432.1911	216.5992	414.1806	207.5939	C	757.3661	379.1867	740.3396	370.6734	739.3556	370.1814	6
5	547.2181	274.1127	529.2075	265.1074	D	597.3355	299.1714	580.3089	290.6581	579.3249	290.1661	5
6	660.3021	330.6547	642.2916	321.6494	I	482.3085	241.6579	465.282	233.1446			4
7	757.3549	379.1811	739.3443	370.1758	P	369.2245	185.1159	352.1979	176.6026			3
8	854.4077	427.7075	836.3971	418.7022	P	272.1717	136.5895	255.1452	128.0762			2
9					R	175.119	88.0631	158.0924	79.5498			1

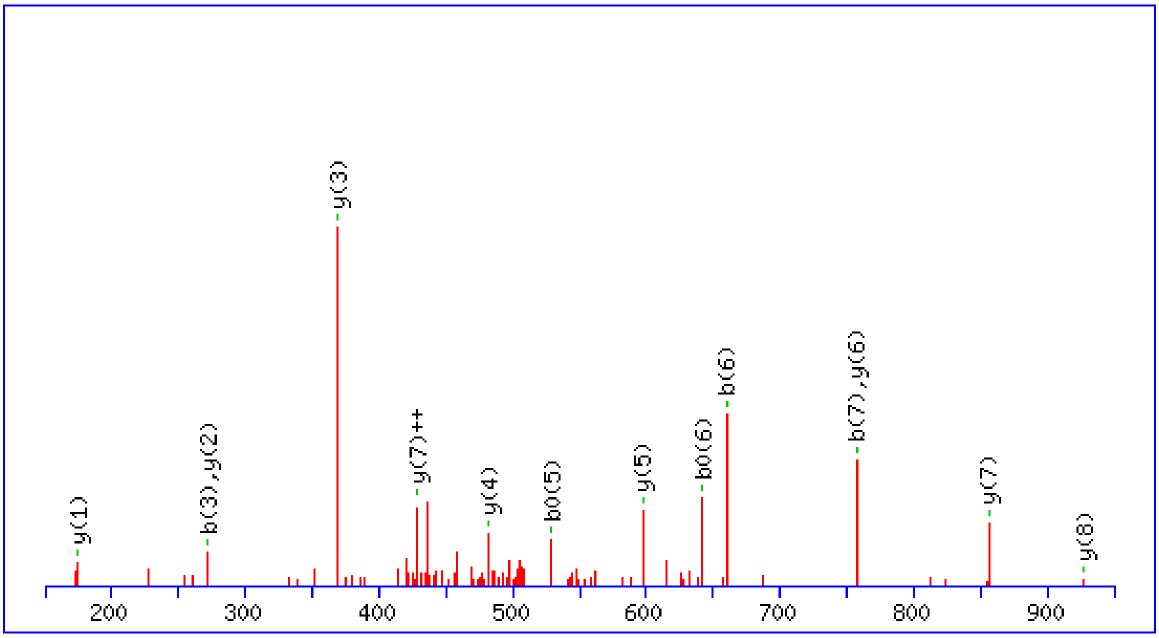

Spot no.181
Tubulin beta-2A chain
MSATFIGNSTAIQELFK

#	b	b++	b*	b*++	b0	b0++	Seq.	y	y++	y*	y*++	y0	y0++	#
1	148.0427	74.525					M							17
2	235.0747	118.041			217.0641	109.0357	S	1726.901	863.9542	1709.8745	855.4409	1708.8905	854.9489	16
3	306.1118	153.5595			288.1013	144.5543	A	***1639.869***	820.4381	1622.8425	811.9249	1621.8584	811.4329	15
4	**407.1595**	204.0834			389.1489	195.0781	T	***1568.8319***	784.9196	1551.8053	776.4063	1550.8213	775.9143	14
5	554.2279	277.6176			536.2173	268.6123	F	1467.7842	734.3957	1450.7577	725.8825	1449.7736	725.3905	13
6	667.312	334.1596			649.3014	325.1543	I	***1320.7158***	660.8615	1303.6892	652.3483	1302.7052	651.8563	12
7	**724.3334**	362.6704			706.3229	353.6651	G	***1207.6317***	604.3195	1190.6052	595.8062	1189.6212	595.3142	11
8	838.3764	419.6918	821.3498	411.1785	820.3658	410.6865	N	***1150.6103***	575.8088	1133.5837	567.2955	1132.5997	566.8035	10
9	925.4084	463.2078	908.3818	454.6946	907.3978	454.2026	S	***1036.5673***	518.7873	1019.5408	510.274	1018.5568	509.782	9
10	1026.4561	513.7317	1009.4295	505.2184	1008.4455	504.7264	T	***949.5353***	475.2713	932.5088	466.758	931.5247	466.266	8
11	1097.4932	549.2502	1080.4666	540.737	1079.4826	540.2449	A	***848.4876***	424.7475	831.4611	416.2342	830.4771	415.7422	7
12	1210.5773	605.7923	1193.5507	597.279	1192.5667	596.787	I	***777.4505***	389.2289	760.424	380.7156	759.44	380.2236	6
13	1338.6358	669.8216	1321.6093	661.3083	1320.6253	660.8163	Q	***664.3665***	332.6869	647.3399	324.1736	646.3559	323.6816	5
14	1467.6784	734.3428	1450.6519	725.8296	1449.6679	725.3376	E	***536.3079***	268.6576	519.2813	260.1443	518.2973	259.6523	4
15	**1580.7625**	790.8849	1563.7359	782.3716	1562.7519	781.8796	L	***407.2653***	204.1363	390.2387	195.623			3
16	**1727.8309**	864.4191	1710.8044	855.9058	1709.8203	855.4138	F	294.1812	147.5942	277.1547	139.081			2
17							K	147.1128	74.06	130.0863	65.5468			1

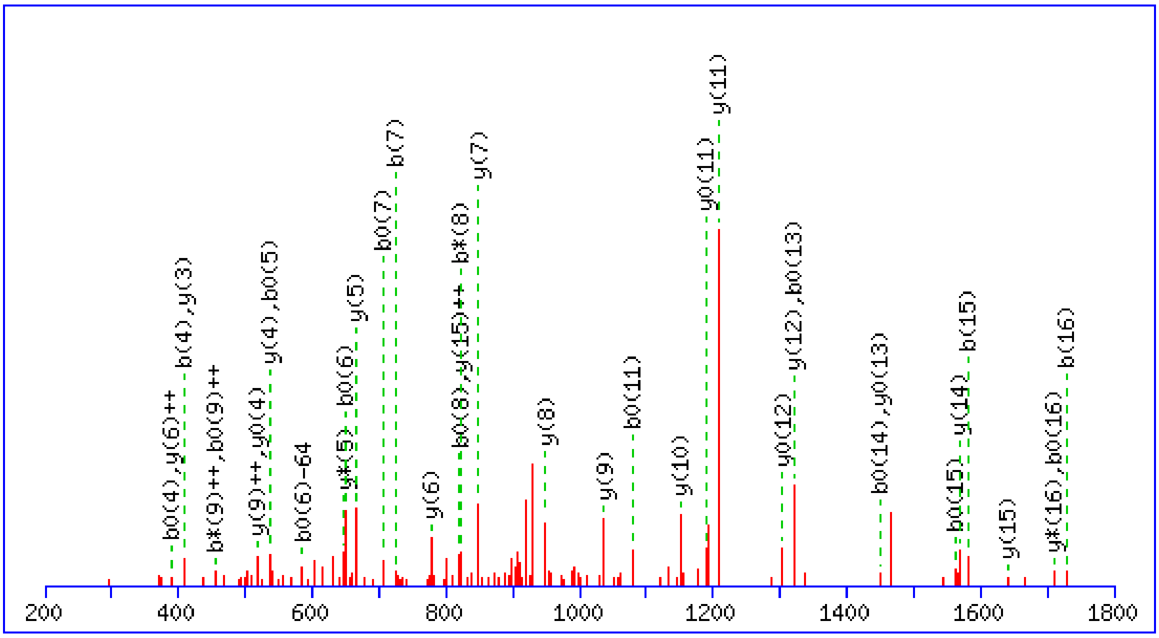

Spot no.181
Tubulin beta-2A chain
ISEQFTAMFR

#	b	b^{++}	b*	b^{*++}	b^{0}	b^{0++}	Seq.	y	y^{++}	y*	y^{*++}	y^{0}	y^{0++}	#
1	114.0913	57.5493					I							10
2	201.1234	101.0653			183.1128	92.06	S	***1132.5092***	566.7582	1115.4826	558.2449	1114.4986	557.7529	9
3	330.166	165.5866			312.1554	156.5813	E	***1045.4771***	523.2422	1028.4506	514.7289	1027.4666	514.2369	8
4	458.2245	229.6159	441.198	221.1026	440.214	220.6106	Q	***916.4346***	458.7209	899.408	450.2076	898.424	449.7156	7
5	**605.293**	303.1501	588.2664	294.6368	587.2824	294.1448	F	***788.376***	394.6916	771.3494	386.1783	770.3654	385.6863	6
6	**706.3406**	353.674	689.3141	345.1607	688.3301	344.6687	T	***641.3076***	321.1574	624.281	312.6441	623.297	312.1521	5
7	**777.3777**	389.1925	760.3512	380.6792	759.3672	380.1872	A	***540.2599***	270.6336	523.2333	262.1203			4
8	**924.4131**	462.7102	907.3866	454.1969	906.4026	453.7049	M	***469.2228***	235.115	452.1962	226.6017			3
9	**1071.4816**	536.2444	1054.455	527.7311	1053.471	527.2391	F	***322.1874***	161.5973	305.1608	153.084			2
10							R	***175.119***	88.0631	158.0924	79.5498			1

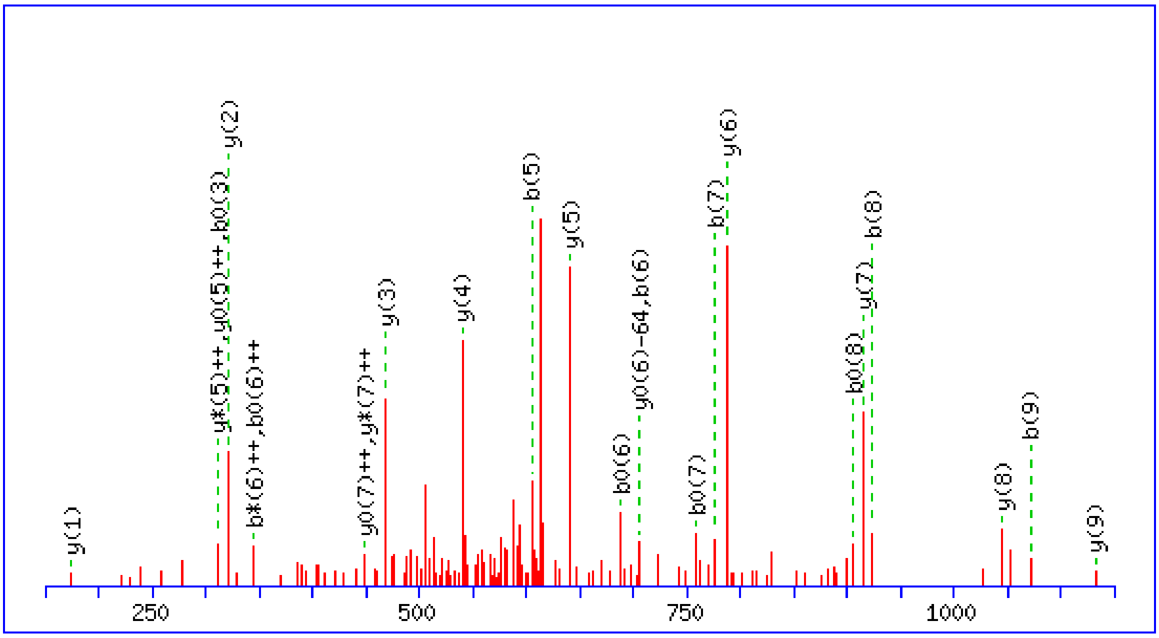

Spot no.610
Prelamin A/C
SGAQASSTPLSPTR

#	b	b++	b*	b*++	b0	b0++	Seq.	y	y++	y*	y*++	y0	y0++	#
1	88.0393	44.5233			70.0287	35.518	S							14
2	145.0608	73.034			127.0502	64.0287	G	1272.6543	636.8308	1255.6277	628.3175	1254.6437	627.8255	13
3	216.0979	108.5526			198.0873	99.5473	A	1215.6328	608.32	1198.6062	599.8068	1197.6222	599.3148	12
4	344.1565	172.5819	327.1299	164.0686	326.1459	163.5766	Q	1144.5957	572.8015	1127.5691	564.2882	1126.5851	563.7962	11
5	415.1936	208.1004	398.167	199.5872	397.183	199.0951	A	1016.5371	508.7722	999.5106	500.2589	998.5265	499.7669	10
6	502.2256	251.6164	485.1991	243.1032	484.215	242.6112	S	***945.5***	473.2536	928.4734	464.7404	927.4894	464.2483	9
7	589.2576	295.1325	572.2311	286.6192	571.2471	286.1272	S	***858.468***	429.7376	841.4414	421.2243	840.4574	420.7323	8
8	690.3053	345.6563	673.2788	337.143	672.2947	336.651	T	***771.4359***	386.2216	754.4094	377.7083	753.4254	377.2163	7
9	787.3581	394.1827	770.3315	385.6694	769.3475	385.1774	P	***670.3883***	335.6978	653.3617	327.1845	652.3777	326.6925	6
10	900.4421	450.7247	883.4156	442.2114	882.4316	441.7194	L	573.3355	287.1714	556.3089	278.6581	555.3249	278.1661	5
11	987.4742	494.2407	970.4476	485.7274	969.4636	485.2354	S	***460.2514***	230.6293	443.2249	222.1161	442.2409	221.6241	4
12	1084.5269	542.7671	1067.5004	534.2538	1066.5164	533.7618	P	***373.2194***	187.1133	356.1928	178.6001	355.2088	178.1081	3
13	1185.5746	593.2909	1168.5481	584.7777	1167.564	584.2857	T	276.1666	138.587	259.1401	130.0737	258.1561	129.5817	2
14							R	175.119	88.0631	158.0924	79.5498			1

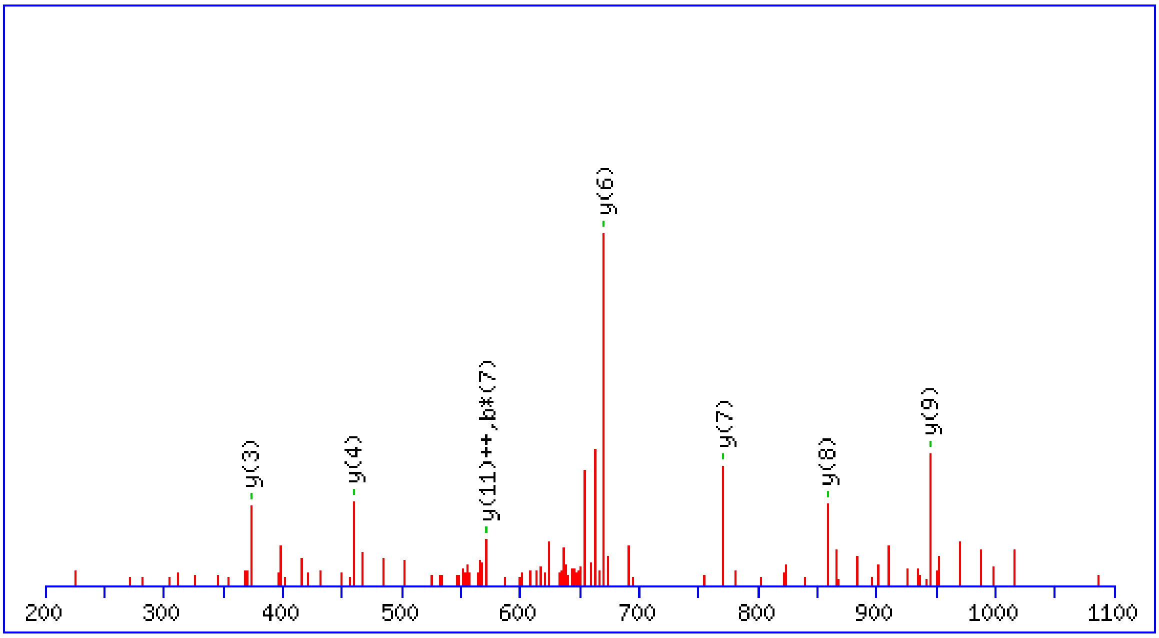

Spot no.610
Prelamin A/C
LQEKEDLQELNDR

#	b	b++	b*	b*++	b0	b0++	Seq.	y	y++	y*	y*++	y0	y0++	#
1	114.0913	57.5493					L							13
2	242.1499	121.5786	225.1234	113.0653			Q	1516.7238	758.8655	1499.6972	750.3523	1498.7132	749.8603	12
3	***371.1925***	186.0999	354.166	177.5866	353.1819	177.0946	E	***1388.6652***	694.8362	1371.6387	686.323	1370.6546	685.831	11
4	***499.2875***	250.1474	482.2609	241.6341	481.2769	241.1421	K	1259.6226	630.3149	1242.5961	621.8017	1241.6121	621.3097	10
5	***628.3301***	314.6687	611.3035	306.1554	610.3195	305.6634	E	***1131.5277***	566.2675	1114.5011	557.7542	1113.5171	557.2622	9
6	743.357	372.1821	726.3305	363.6689	725.3464	363.1769	D	***1002.4851***	501.7462	985.4585	493.2329	984.4745	492.7409	8
7	856.4411	428.7242	839.4145	420.2109	838.4305	419.7189	L	***887.4581***	444.2327	870.4316	435.7194	869.4476	435.2274	7
8	***984.4997***	492.7535	967.4731	484.2402	966.4891	483.7482	Q	***774.3741***	387.6907	757.3475	379.1774	756.3635	378.6854	6
9	***1113.5422***	557.2748	1096.5157	548.7615	1095.5317	548.2695	E	***646.3155***	323.6614	629.2889	315.1481	628.3049	314.6561	5
10	1226.6263	613.8168	1209.5998	605.3035	1208.6157	604.8115	L	***517.2729***	259.1401	500.2463	250.6268	499.2623	250.1348	4
11	***1340.6692***	670.8383	1323.6427	662.325	1322.6587	661.833	N	***404.1888***	202.598	387.1623	194.0848	386.1783	193.5928	3
12	***1455.6962***	728.3517	1438.6696	719.8385	1437.6856	719.3464	D	290.1459	145.5766	273.1193	137.0633	272.1353	136.5713	2
13							R	175.119	88.0631	158.0924	79.5498			1

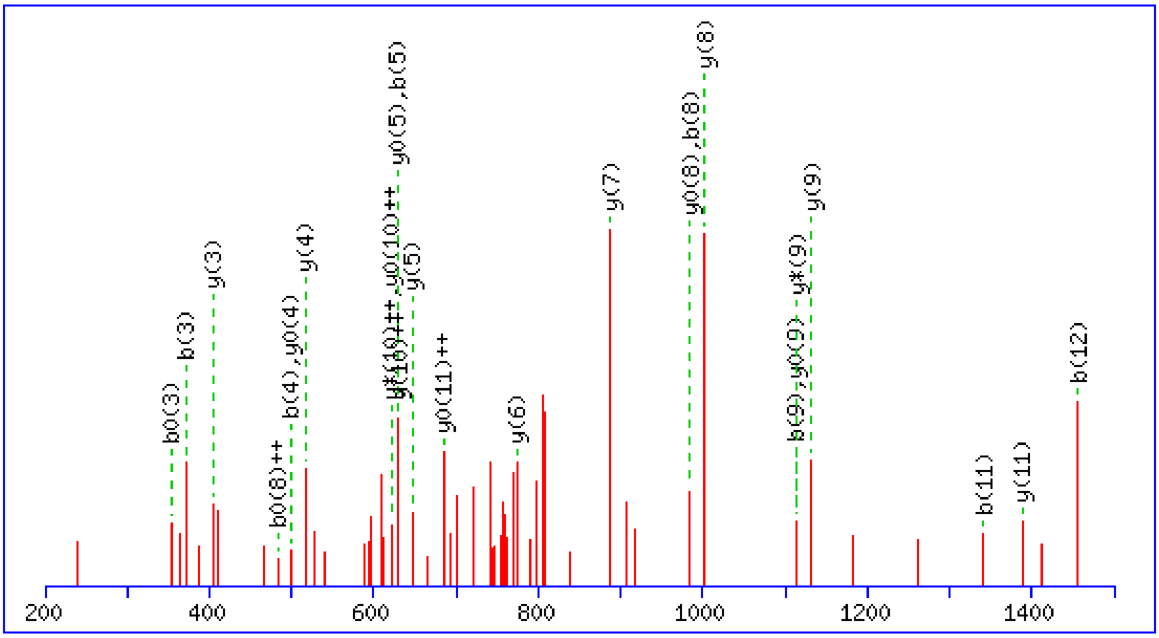

Spot no.610
Prelamin A/C
EDLQELNDR

#	b	b++	b*	b*++	b0	b0++	Seq.	y	y++	y*	y*++	y0	y0++	#
1	130.0499	65.5286			112.0393	56.5233	E							9
2	245.0768	123.042			227.0662	114.0368	D	1002.4851	501.7462	985.4585	493.2329	984.4745	492.7409	8
3	358.1609	179.5841			340.1503	170.5788	L	***887.4581***	444.2327	870.4316	435.7194	869.4476	435.2274	7
4	486.2195	243.6134	469.1929	235.1001	468.2089	234.6081	Q	***774.3741***	387.6907	757.3475	379.1774	756.3635	378.6854	6
5	615.262	308.1347	598.2355	299.6214	597.2515	299.1294	E	***646.3155***	323.6614	629.2889	315.1481	628.3049	314.6561	5
6	**728.3461**	364.6767	711.3196	356.1634	710.3355	355.6714	L	***517.2729***	259.1401	500.2463	250.6268	499.2623	250.1348	4
7	842.389	421.6982	825.3625	413.1849	824.3785	412.6929	N	***404.1888***	202.598	387.1623	194.0848	386.1783	193.5928	3
8	**957.416**	479.2116	940.3894	470.6984	939.4054	470.2063	D	***290.1459***	145.5766	273.1193	137.0633	272.1353	136.5713	2
9							R	175.119	88.0631	158.0924	79.5498			1

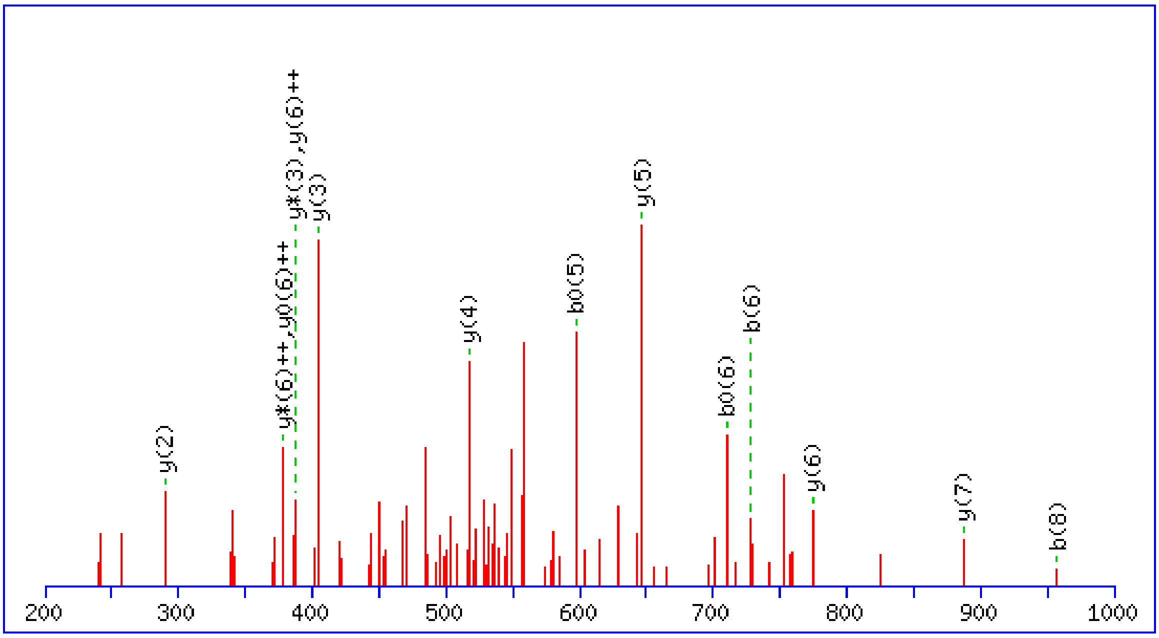

Spot no.610
Prelamin A/C
LAVYIDR

#	b	b^{++}	b^0	b^{0++}	Seq.	y	y^{++}	y*	y^{*++}	y^0	y^{0++}	#
1	114.0913	57.5493			L							7
2	185.1285	93.0679			A	736.3988	368.703	719.3723	360.1898	718.3883	359.6978	6
3	284.1969	142.6021			V	665.3617	333.1845	648.3352	324.6712	647.3511	324.1792	5
4	447.2602	224.1337			Y	566.2933	283.6503	549.2667	275.137	548.2827	274.645	4
5	560.3443	280.6758			I	403.23	202.1186	386.2034	193.6053	385.2194	193.1133	3
6	675.3712	338.1892	657.3606	329.184	D	290.1459	145.5766	273.1193	137.0633	272.1353	136.5713	2
7					R	175.119	88.0631	158.0924	79.5498			1

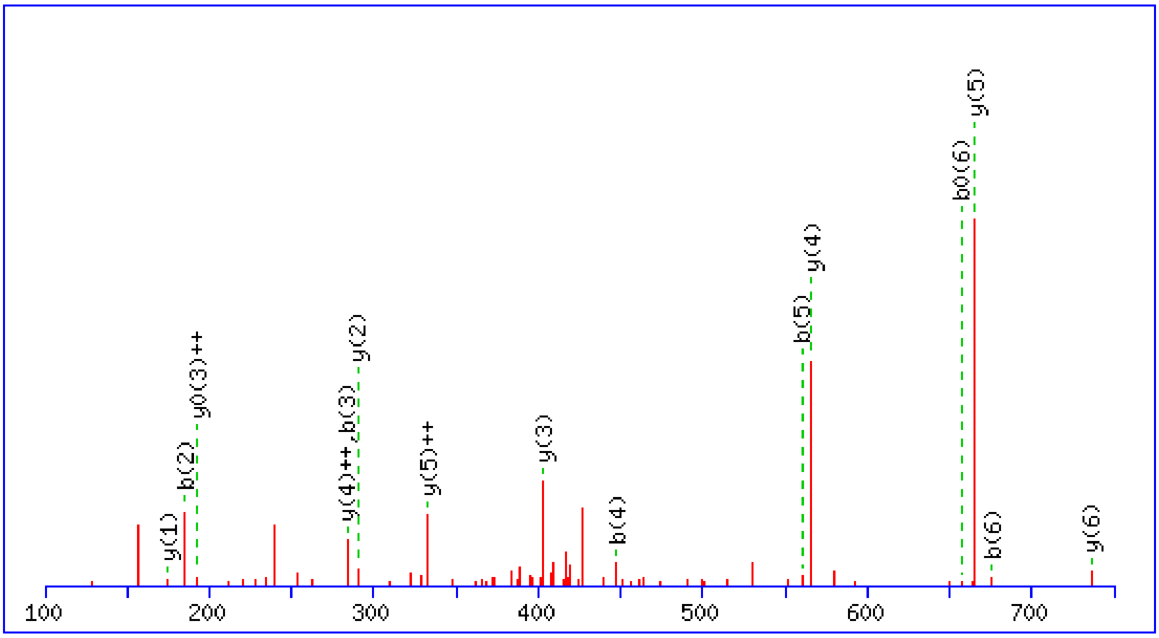

Spot no.610
Prelamin A/C
SLETENAGLR

#	b	b++	b*	b*++	b0	b0++	Seq.	y	y++	y*	y*++	y0	y0++	#
1	88.0393	44.5233			70.0287	35.518	S							10
2	**201.1234**	101.0653			183.1128	92.06	L	1002.5215	501.7644	985.4949	493.2511	984.5109	492.7591	9
3	**330.166**	165.5866			312.1554	156.5813	E	***889.4374***	445.2223	872.4108	436.7091	871.4268	436.217	8
4	431.2136	216.1105			413.2031	207.1052	T	***760.3948***	380.701	743.3682	372.1878	742.3842	371.6958	7
5	560.2562	280.6318			542.2457	271.6265	E	***659.3471***	330.1772	642.3206	321.6639	641.3365	321.1719	6
6	674.2992	337.6532	657.2726	329.1399	656.2886	328.6479	N	530.3045	265.6559	513.278	257.1426			5
7	**745.3363**	373.1718	728.3097	364.6585	727.3257	364.1665	A	***416.2616***	208.6344	399.235	200.1212			4
8	802.3577	401.6825	785.3312	393.1692	784.3472	392.6772	G	***345.2245***	173.1159	328.1979	164.6026			3
9	**915.4418**	458.2245	898.4153	449.7113	897.4312	449.2193	L	***288.203***	144.6051	271.1765	136.0919			2
10							R	***175.119***	88.0631	158.0924	79.5498			1

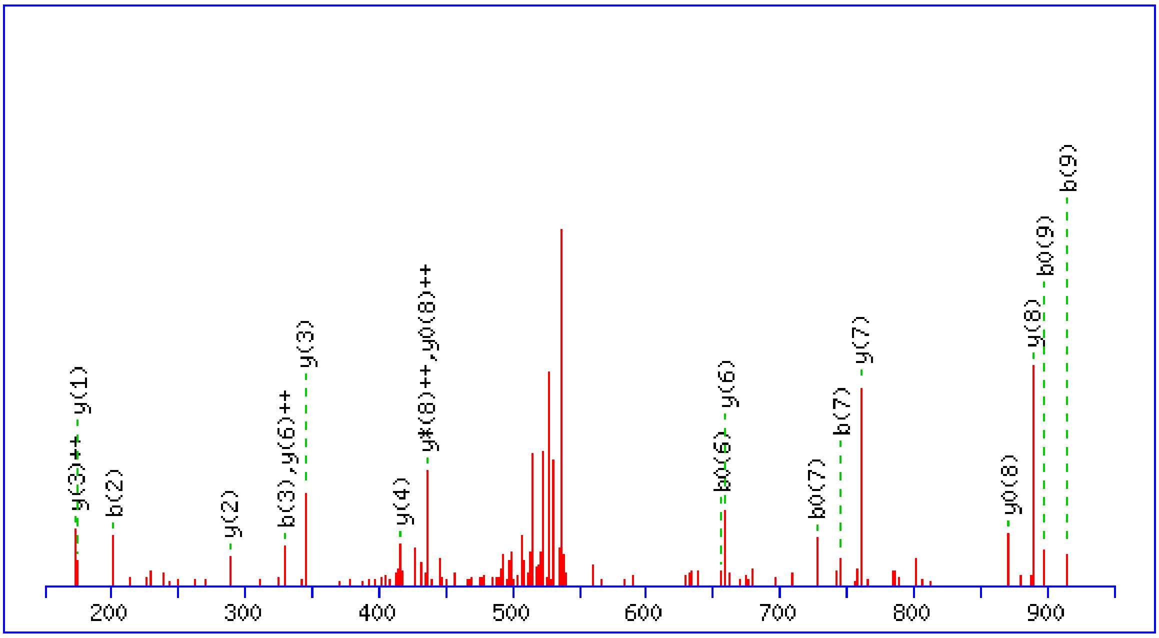

Spot no.610
Prelamin A/C
ITESEEVVSR

#	b	b++	b0	b0++	Seq.	y	y++	y*	y*++	y0	y0++	#
1	114.0913	57.5493			I							10
2	215.139	108.0731	197.1285	99.0679	T	1035.4953	518.2513	1018.4687	509.738	1017.4847	509.246	9
3	344.1816	172.5944	326.171	163.5892	E	934.4476	467.7274	917.4211	459.2142	916.437	458.7222	8
4	431.2136	216.1105	413.2031	207.1052	S	805.405	403.2061	788.3785	394.6929	787.3945	394.2009	7
5	560.2562	280.6318	542.2457	271.6265	E	718.373	359.6901	701.3464	351.1769	700.3624	350.6849	6
6	689.2988	345.1531	671.2883	336.1478	E	589.3304	295.1688	572.3039	286.6556	571.3198	286.1636	5
7	788.3672	394.6873	770.3567	385.682	V	460.2878	230.6475	443.2613	222.1343	442.2772	221.6423	4
8	887.4357	444.2215	869.4251	435.2162	V	361.2194	181.1133	344.1928	172.6001	343.2088	172.1081	3
9	974.4677	487.7375	956.4571	478.7322	S	262.151	131.5791	245.1244	123.0659	244.1404	122.5738	2
10					R	175.119	88.0631	158.0924	79.5498			1

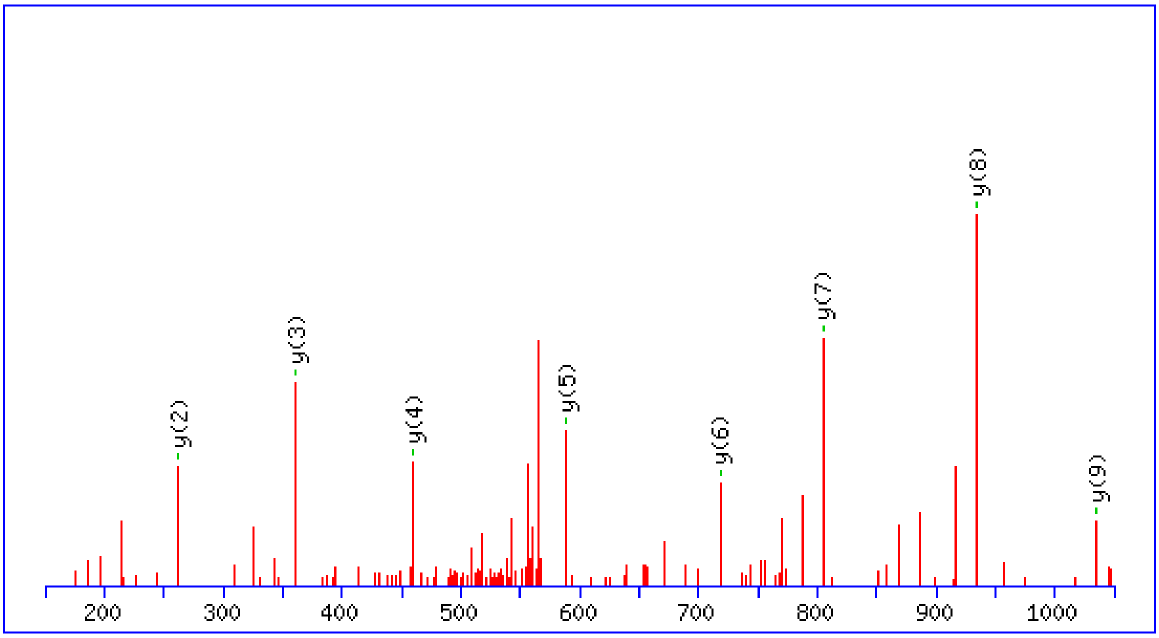

Spot no.610
Prelamin A/C
AAYEAELGDAR

#	b	b++	b0	b0++	Seq.	y	y++	y*	y*++	y0	y0++	#
1	72.0444	36.5258			A							11
2	143.0815	72.0444			A	1094.5113	547.7593	1077.4847	539.246	1076.5007	538.754	10
3	306.1448	153.5761			Y	1023.4742	512.2407	1006.4476	503.7274	1005.4636	503.2354	9
4	435.1874	218.0974	417.1769	209.0921	E	860.4108	430.7091	843.3843	422.1958	842.4003	421.7038	8
5	506.2245	253.6159	488.214	244.6106	A	731.3682	366.1878	714.3417	357.6745	713.3577	357.1825	7
6	635.2671	318.1372	617.2566	309.1319	E	660.3311	330.6692	643.3046	322.1559	642.3206	321.6639	6
7	748.3512	374.6792	730.3406	365.674	L	531.2885	266.1479	514.262	257.6346	513.278	257.1426	5
8	805.3727	403.19	787.3621	394.1847	G	418.2045	209.6059	401.1779	201.0926	400.1939	200.6006	4
9	920.3996	460.7034	902.389	451.6982	D	361.183	181.0951	344.1565	172.5819	343.1724	172.0899	3
10	991.4367	496.222	973.4262	487.2167	A	246.1561	123.5817	229.1295	115.0684			2
11					R	175.119	88.0631	158.0924	79.5498			1

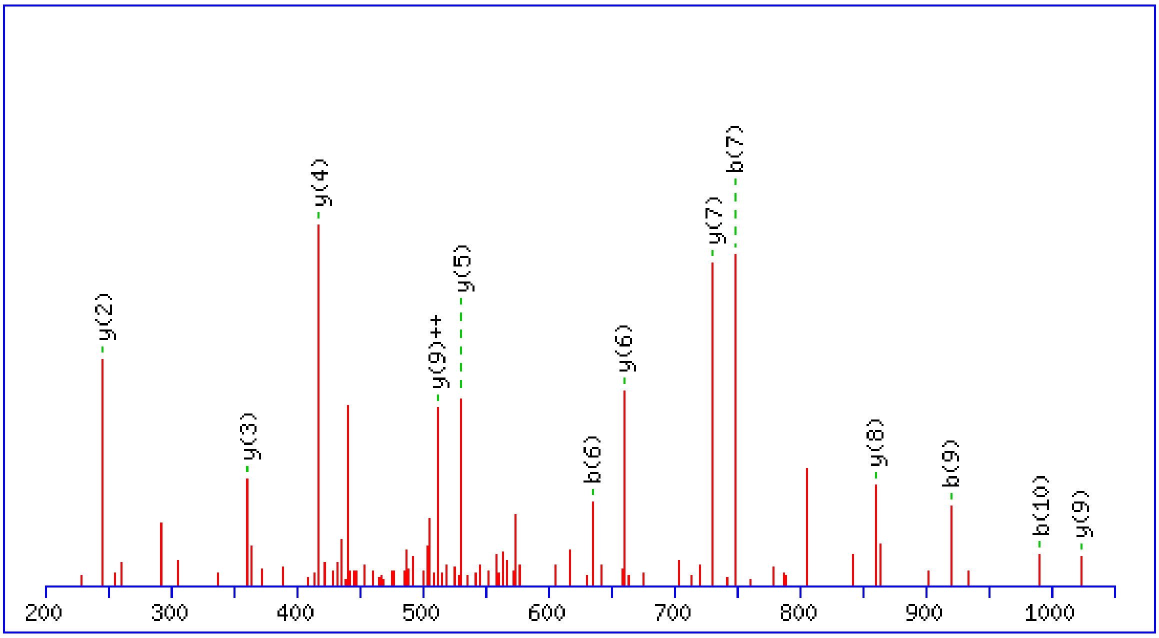

Spot no.610
Prelamin A/C
KTLDSVAK

#	b	b++	b*	b*++	b0	b0++	Seq.	y	y++	y*	y*++	y0	y0++	#
1	***129.1022***	65.0548	112.0757	56.5415			K							8
2	230.1499	115.5786	213.1234	107.0653	212.1394	106.5733	T	***733.409***	367.2082	716.3825	358.6949	715.3985	358.2029	7
3	343.234	172.1206	326.2074	163.6074	325.2234	163.1153	L	***632.3614***	316.6843	615.3348	308.171	614.3508	307.679	6
4	458.2609	229.6341	441.2344	221.1208	440.2504	220.6288	D	519.2773	260.1423	502.2508	251.629	501.2667	251.137	5
5	***545.293***	273.1501	528.2664	264.6368	527.2824	264.1448	S	***404.2504***	202.6288	387.2238	194.1155	386.2398	193.6235	4
6	644.3614	322.6843	627.3348	314.171	626.3508	313.679	V	***317.2183***	159.1128	300.1918	150.5995			3
7	***715.3985***	358.2029	698.3719	349.6896	697.3879	349.1976	A	218.1499	109.5786	201.1234	101.0653			2
8							K	147.1128	74.06	130.0863	65.5468			1

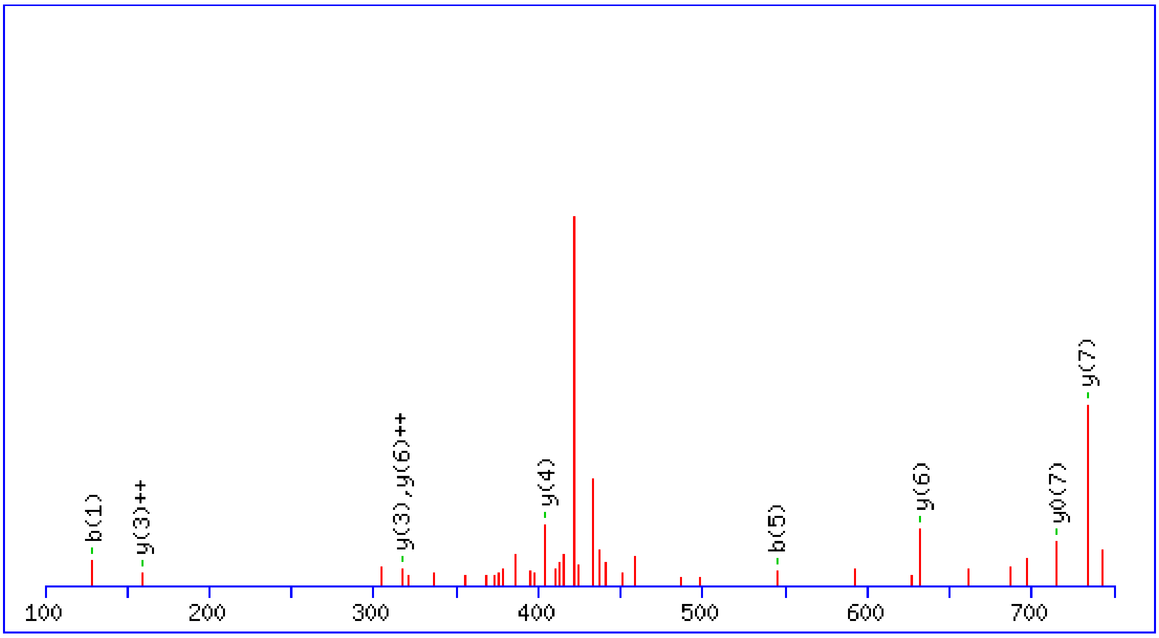

Spot no.610
Prelamin A/C
EGDLMAAQAR

#	b	b^{++}	b*	b*$^{++}$	b^{0}	b^{0++}	Seq.	y	y^{++}	y*	y*$^{++}$	y^{0}	y^{0++}	#
1	130.0499	65.5286			112.0393	56.5233	E							10
2	187.0713	94.0393			169.0608	85.034	G	948.4567	474.732	931.4302	466.2187	930.4462	465.7267	9
3	302.0983	151.5528			284.0877	142.5475	D	***891.4353***	446.2213	874.4087	437.708	873.4247	437.216	8
4	415.1823	208.0948			397.1718	199.0895	L	***776.4083***	388.7078	759.3818	380.1945			7
5	562.2177	281.6125			544.2072	272.6072	M	***663.3243***	332.1658	646.2977	323.6525			6
6	**633.2549**	317.1311			615.2443	308.1258	A	***516.2889***	258.6481	499.2623	250.1348			5
7	**704.292**	352.6496			686.2814	343.6443	A	***445.2518***	223.1295	428.2252	214.6162			4
8	**832.3505**	416.6789	815.324	408.1656	814.34	407.6736	Q	***374.2146***	187.611	357.1881	179.0977			3
9	**903.3877**	452.1975	886.3611	443.6842	885.3771	443.1922	A	***246.1561***	123.5817	229.1295	115.0684			2
10							R	***175.119***	88.0631	158.0924	79.5498			1

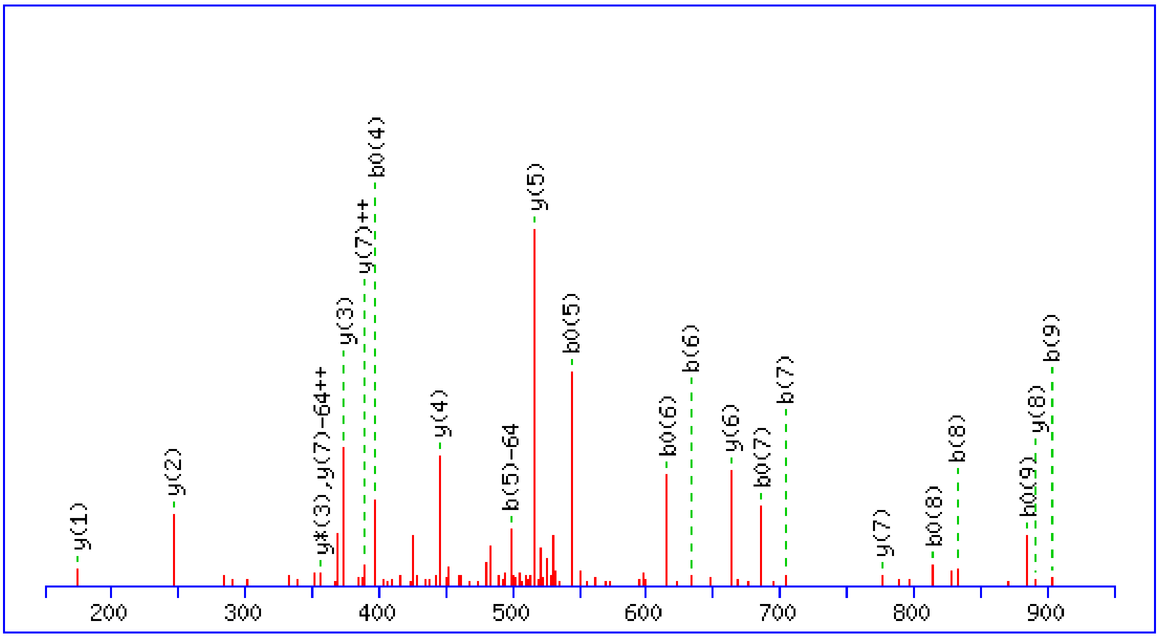

Spot no.610
Prelamin A/C
EAALSTALSEK

#	b	b++	b0	b0++	Seq.	y	y++	y*	y*++	y0	y0++	#
1	130.0499	65.5286	112.0393	56.5233	E							11
2	201.087	101.0471	183.0764	92.0418	A	990.5466	495.7769	973.5201	487.2637	972.536	486.7717	10
3	272.1241	136.5657	254.1135	127.5604	A	***919.5095***	460.2584	902.4829	451.7451	901.4989	451.2531	9
4	385.2082	193.1077	367.1976	184.1024	L	***848.4724***	424.7398	831.4458	416.2266	830.4618	415.7345	8
5	472.2402	236.6237	454.2296	227.6185	S	***735.3883***	368.1978	718.3618	359.6845	717.3777	359.1925	7
6	573.2879	287.1476	555.2773	278.1423	T	***648.3563***	324.6818	631.3297	316.1685	630.3457	315.6765	6
7	644.325	322.6661	626.3144	313.6608	A	547.3086	274.1579	530.2821	265.6447	529.298	265.1527	5
8	757.409	379.2082	739.3985	370.2029	L	***476.2715***	238.6394	459.2449	230.1261	458.2609	229.6341	4
9	844.4411	422.7242	826.4305	413.7189	S	***363.1874***	182.0974	346.1609	173.5841	345.1769	173.0921	3
10	973.4837	487.2455	955.4731	478.2402	E	276.1554	138.5813	259.1288	130.0681	258.1448	129.5761	2
11					K	147.1128	74.06	130.0863	65.5468			1

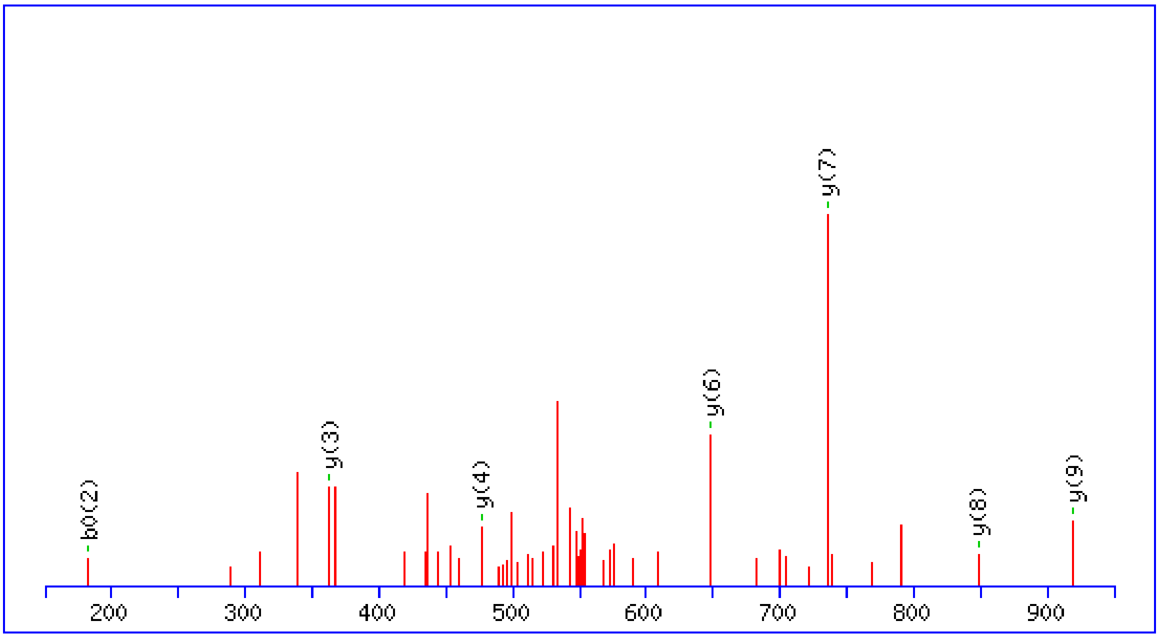

Spot no.610
Prelamin A/C
TLEGELHDLR

#	b	b++	b0	b0++	Seq.	y	y++	y*	y*++	y0	y0++	#
1	102.055	51.5311	84.0444	42.5258	T							10
2	215.139	108.0731	197.1285	99.0679	L	1081.5636	541.2855	1064.5371	532.7722	1063.5531	532.2802	9
3	344.1816	172.5944	326.171	163.5892	E	***968.4796***	484.7434	951.453	476.2302	950.469	475.7381	8
4	401.2031	201.1052	383.1925	192.0999	G	***839.437***	420.2221	822.4104	411.7089	821.4264	411.2169	7
5	530.2457	265.6265	512.2351	256.6212	E	782.4155	391.7114	765.389	383.1981	764.405	382.7061	6
6	643.3297	322.1685	625.3192	313.1632	L	***653.3729***	327.1901	636.3464	318.6768	635.3624	318.1848	5
7	**780.3886**	390.698	762.3781	381.6927	H	***540.2889***	270.6481	523.2623	262.1348	522.2783	261.6428	4
8	**895.4156**	448.2114	877.405	439.2061	D	***403.23***	202.1186	386.2034	193.6053	385.2194	193.1133	3
9	**1008.4997**	504.7535	990.4891	495.7482	L	***288.203***	144.6051	271.1765	136.0919			2
10					R	175.119	88.0631	158.0924	79.5498			1

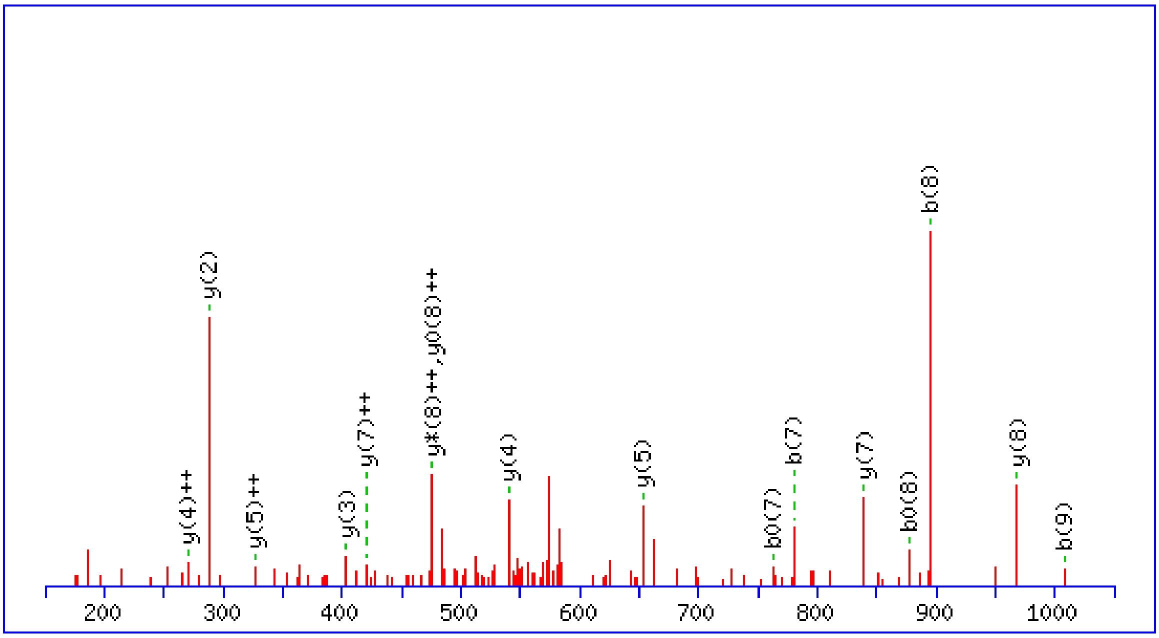

Spot no.610
Prelamin A/C
LVEIDNGK

#	b	b++	b*	b*++	b0	b0++	Seq.	y	y++	y*	y*++	y0	y0++	#
1	114.0913	57.5493					L							8
2	**213.1598**	107.0835					V	***774.3992***	387.7032	757.3727	379.19	756.3886	378.698	7
3	**342.2023**	171.6048			324.1918	162.5995	E	***675.3308***	338.169	658.3042	329.6558	657.3202	329.1638	6
4	455.2864	228.1468			437.2758	219.1416	I	***546.2882***	273.6477	529.2617	265.1345	528.2776	264.6425	5
5	570.3134	285.6603			552.3028	276.655	D	***433.2041***	217.1057	416.1776	208.5924	415.1936	208.1004	4
6	684.3563	342.6818	667.3297	334.1685	666.3457	333.6765	N	***318.1772***	159.5922	301.1506	151.079			3
7	741.3777	371.1925	724.3512	362.6792	723.3672	362.1872	G	204.1343	102.5708	187.1077	94.0575			2
8							K	147.1128	74.06	130.0863	65.5468			1

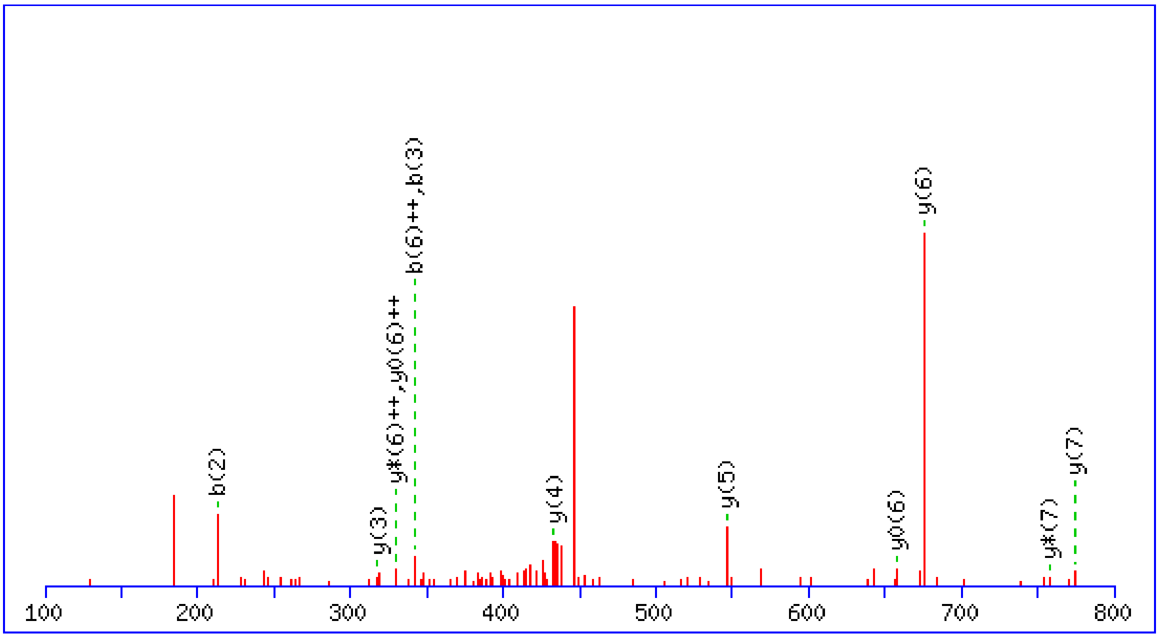

Spot no.610
Prelamin A/C
LADALQELR

#	b	b++	b*	b*++	b0	b0++	Seq.	y	y++	y*	y*++	y0	y0++	#
1	114.0913	57.5493					L							9
2	**185.1285**	93.0679					A	***915.4894***	458.2483	898.4629	449.7351	897.4789	449.2431	8
3	**300.1554**	150.5813			282.1448	141.5761	D	***844.4523***	422.7298	827.4258	414.2165	826.4417	413.7245	7
4	**371.1925**	186.0999			353.1819	177.0946	A	***729.4254***	365.2163	712.3988	356.703	711.4148	356.211	6
5	484.2766	242.6419			466.266	233.6366	L	***658.3883***	329.6978	641.3617	321.1845	640.3777	320.6925	5
6	**612.3352**	306.6712	595.3086	298.1579	594.3246	297.6659	Q	***545.3042***	273.1557	528.2776	264.6425	527.2936	264.1504	4
7	741.3777	371.1925	724.3512	362.6792	723.3672	362.1872	E	***417.2456***	209.1264	400.2191	200.6132	399.235	200.1212	3
8	**854.4618**	427.7345	837.4353	419.2213	836.4512	418.7293	L	***288.203***	144.6051	271.1765	136.0919			2
9							R	***175.119***	88.0631	158.0924	79.5498			1

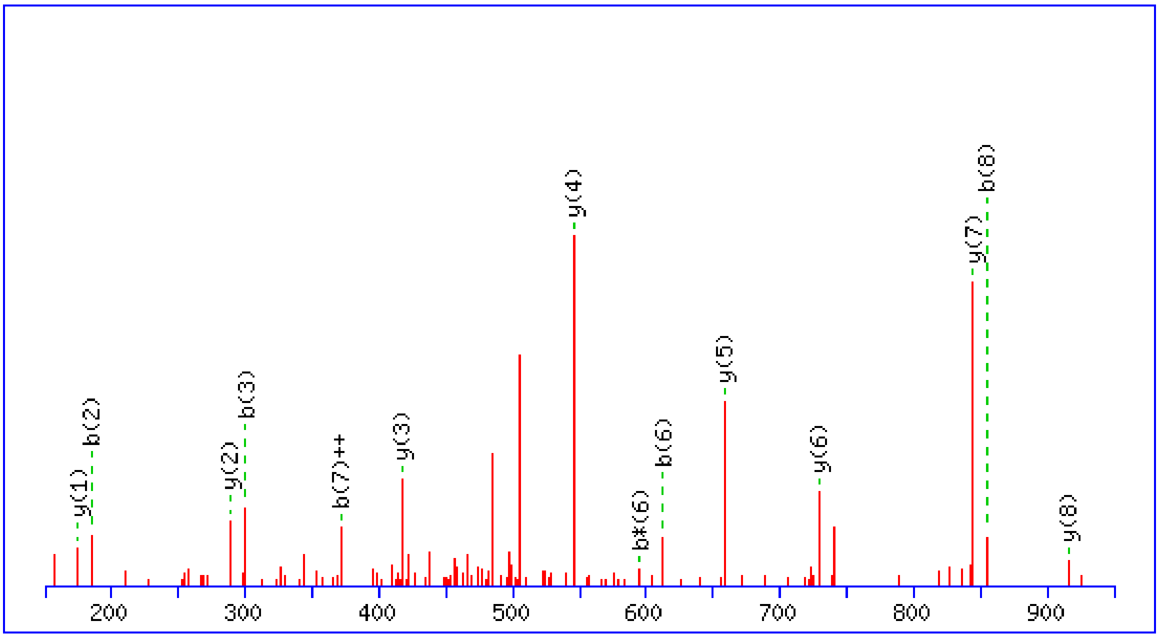

Spot no.610
Prelamin A/C
AQHEDQVEQYKK

#	b	b^{++}	b*	b^{*++}	b^{0}	b^{0++}	Seq.	y	y^{++}	y*	y^{*++}	y^{0}	y^{0++}	#
1	72.0444	36.5258					A							12
2	200.103	100.5551	183.0764	92.0418			Q	1431.6863	716.3468	1414.6597	707.8335	1413.6757	707.3415	11
3	337.1619	169.0846	320.1353	160.5713			H	1303.6277	652.3175	1286.6012	643.8042	1285.6171	643.3122	10
4	466.2045	233.6059	449.1779	225.0926	448.1939	224.6006	E	**1166.5688**	583.788	1149.5422	575.2748	1148.5582	574.7828	9
5	***581.2314***	291.1193	564.2049	282.6061	563.2209	282.1141	D	**1037.5262**	519.2667	1020.4997	510.7535	1019.5156	510.2615	8
6	709.29	355.1486	692.2634	346.6354	691.2794	346.1434	Q	922.4993	461.7533	905.4727	453.24	904.4887	452.748	7
7	***808.3584***	404.6828	791.3319	396.1696	790.3478	395.6776	V	794.4407	397.724	777.4141	389.2107	776.4301	388.7187	6
8	***937.401***	469.2041	920.3745	460.6909	919.3904	460.1989	E	695.3723	348.1898	678.3457	339.6765	677.3617	339.1845	5
9	***1065.4596***	533.2334	1048.433	524.7202	1047.449	524.2281	Q	566.3297	283.6685	549.3031	275.1552			4
10	***1228.5229***	614.7651	1211.4964	606.2518	1210.5123	605.7598	Y	438.2711	219.6392	421.2445	211.1259			3
11	***1356.6179***	678.8126	1339.5913	670.2993	1338.6073	669.8073	K	275.2078	138.1075	258.1812	129.5942			2
12							K	147.1128	74.06	130.0863	65.5468			1

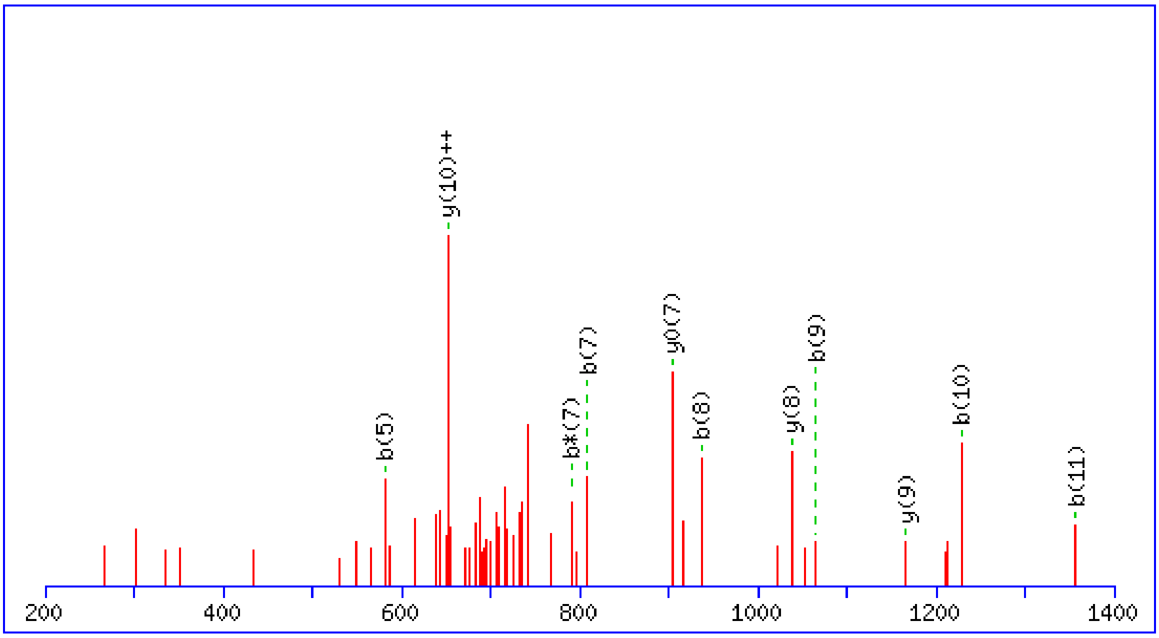

Spot no.610
Prelamin A/C
NSNLVGAAHEELQQSR

#	b	b^{++}	b*	b^{*++}	b^{0}	b^{0++}	Seq.	y	y^{++}	y*	y^{*++}	y^{0}	y^{0++}	#
1	115.0502	58.0287	98.0237	49.5155			N							16
2	202.0822	101.5448	185.0557	93.0315	184.0717	92.5395	S	1638.8194	819.9133	1621.7929	811.4001	1620.8089	810.9081	15
3	**316.1252**	158.5662	299.0986	150.0529	298.1146	149.5609	N	1551.7874	776.3973	1534.7608	767.8841	1533.7768	767.3921	14
4	**429.2092**	215.1082	412.1827	206.595	411.1987	206.103	L	1437.7445	719.3759	1420.7179	710.8626	1419.7339	710.3706	13
5	528.2776	264.6425	511.2511	256.1292	510.2671	255.6372	V	***1324.6604***	662.8338	1307.6339	654.3206	1306.6498	653.8286	12
6	585.2991	293.1532	568.2726	284.6399	567.2885	284.1479	G	***1225.592***	613.2996	1208.5654	604.7864	1207.5814	604.2944	11
7	656.3362	328.6717	639.3097	320.1585	638.3256	319.6665	A	***1168.5705***	584.7889	1151.544	576.2756	1150.56	575.7836	10
8	727.3733	364.1903	710.3468	355.677	709.3628	355.185	A	***1097.5334***	549.2703	1080.5069	540.7571	1079.5228	540.2651	9
9	864.4322	432.7198	847.4057	424.2065	846.4217	423.7145	H	***1026.4963***	513.7518	1009.4697	505.2385	1008.4857	504.7465	8
10	**993.4748**	497.2411	976.4483	488.7278	975.4643	488.2358	E	***889.4374***	445.2223	872.4108	436.7091	871.4268	436.217	7
11	1122.5174	561.7624	1105.4909	553.2491	1104.5069	552.7571	E	***760.3948***	380.701	743.3682	372.1878	742.3842	371.6958	6
12	**1235.6015**	618.3044	1218.5749	609.7911	1217.5909	609.2991	L	***631.3522***	316.1797	614.3257	307.6665	613.3416	307.1745	5
13	1363.6601	682.3337	1346.6335	673.8204	1345.6495	673.3284	Q	***518.2681***	259.6377	501.2416	251.1244	500.2576	250.6324	4
14	**1491.7186**	746.363	1474.6921	737.8497	1473.7081	737.3577	Q	390.2096	195.6084	373.183	187.0951	372.199	186.6031	3
15	1578.7507	789.879	1561.7241	781.3657	1560.7401	780.8737	S	262.151	131.5791	245.1244	123.0659	244.1404	122.5738	2
16							R	175.119	88.0631	158.0924	79.5498			1

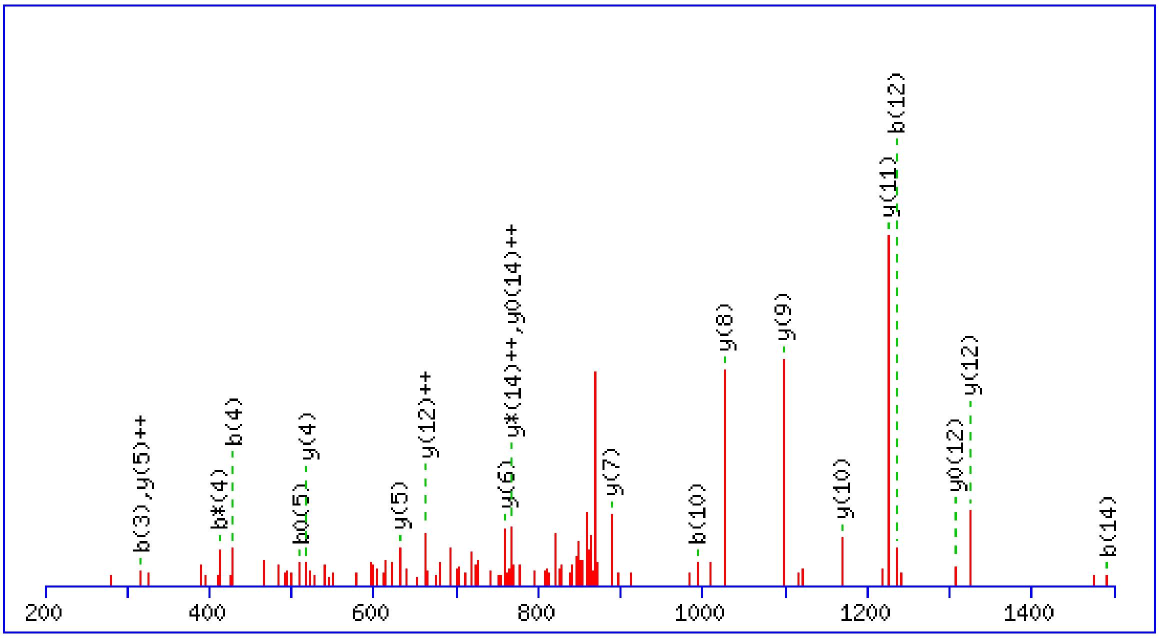

Spot no.610
Prelamin A/C
IDSLSAQLSQLQK

#	b	b++	b*	b*++	b0	b0++	Seq.	y	y++	y*	y*++	y0	y0++	#
1	114.0913	57.5493					I							13
2	229.1183	115.0628			211.1077	106.0575	D	1317.7009	659.3541	1300.6743	650.8408	1299.6903	650.3488	12
3	316.1503	158.5788			298.1397	149.5735	S	***1202.6739***	601.8406	1185.6474	593.3273	1184.6634	592.8353	11
4	429.2344	215.1208			411.2238	206.1155	L	***1115.6419***	558.3246	1098.6154	549.8113	1097.6313	549.3193	10
5	***516.2664***	258.6368			498.2558	249.6316	S	***1002.5578***	501.7826	985.5313	493.2693	984.5473	492.7773	9
6	587.3035	294.1554			569.293	285.1501	A	***915.5258***	458.2665	898.4993	449.7533	897.5152	449.2613	8
7	715.3621	358.1847	698.3355	349.6714	697.3515	349.1794	Q	***844.4887***	422.748	827.4621	414.2347	826.4781	413.7427	7
8	***828.4462***	414.7267	811.4196	406.2134	810.4356	405.7214	L	716.4301	358.7187	699.4036	350.2054	698.4196	349.7134	6
9	***915.4782***	458.2427	898.4516	449.7295	897.4676	449.2374	S	603.3461	302.1767	586.3195	293.6634	585.3355	293.1714	5
10	***1043.5368***	522.272	1026.5102	513.7587	1025.5262	513.2667	Q	***516.314***	258.6606	499.2875	250.1474			4
11	***1156.6208***	578.8141	1139.5943	570.3008	1138.6103	569.8088	L	***388.2554***	194.6314	371.2289	186.1181			3
12	***1284.6794***	642.8433	1267.6529	634.3301	1266.6688	633.8381	Q	***275.1714***	138.0893	258.1448	129.5761			2
13							K	147.1128	74.06	130.0863	65.5468			1

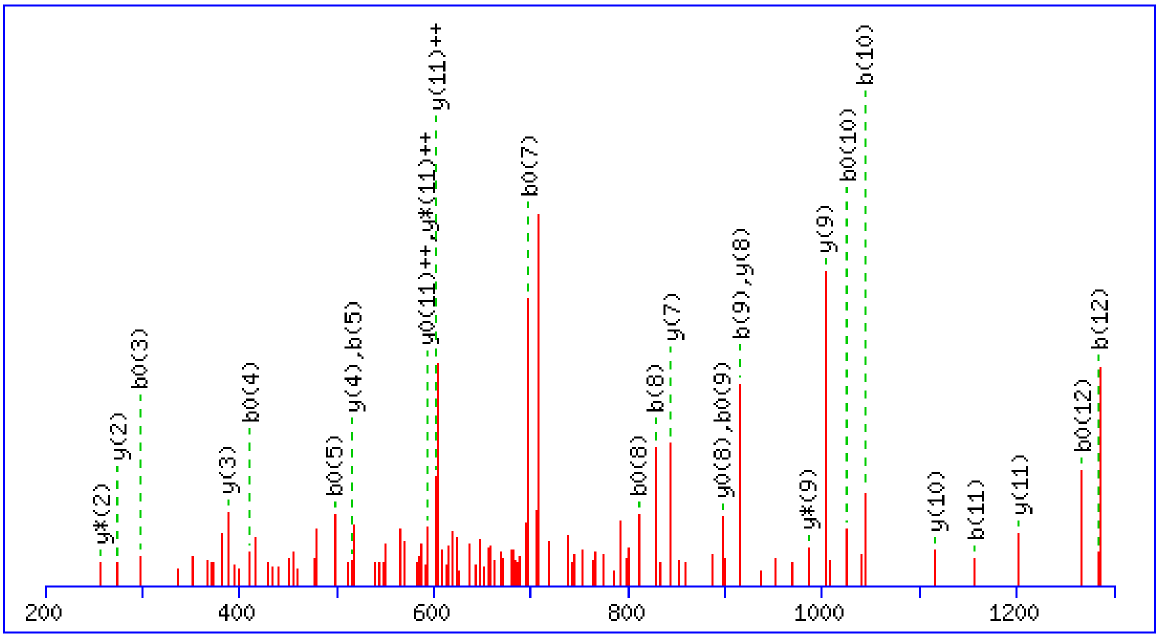

Spot no.610
Prelamin A/C
LRDLEDSLAR

#	b	b^{++}	b*	b^{*++}	b^{0}	b^{0++}	Seq.	y	y^{++}	y*	y^{*++}	y^{0}	y^{0++}	#
1	114.0913	57.5493					L							10
2	***270.1925***	135.5999	253.1659	127.0866			R	1074.5538	537.7805	1057.5273	529.2673	1056.5432	528.7753	9
3	***385.2194***	193.1133	368.1928	184.6001	367.2088	184.1081	D	**918.4527**	459.73	901.4262	451.2167	900.4421	450.7247	8
4	***498.3035***	249.6554	481.2769	241.1421	480.2929	240.6501	L	**803.4258**	402.2165	786.3992	393.7032	785.4152	393.2112	7
5	***627.3461***	314.1767	610.3195	305.6634	609.3355	305.1714	E	690.3417	345.6745	673.3151	337.1612	672.3311	336.6692	6
6	***742.373***	371.6901	725.3464	363.1769	724.3624	362.6849	D	**561.2991**	281.1532	544.2726	272.6399	543.2885	272.1479	5
7	***829.405***	415.2061	812.3785	406.6929	811.3945	406.2009	S	**446.2722**	223.6397	429.2456	215.1264	428.2616	214.6344	4
8	942.4891	471.7482	925.4625	463.2349	924.4785	462.7429	L	359.2401	180.1237	342.2136	171.6104			3
9	***1013.5262***	507.2667	996.4997	498.7535	995.5156	498.2615	A	246.1561	123.5817	229.1295	115.0684			2
10							R	**175.119**	88.0631	158.0924	79.5498			1

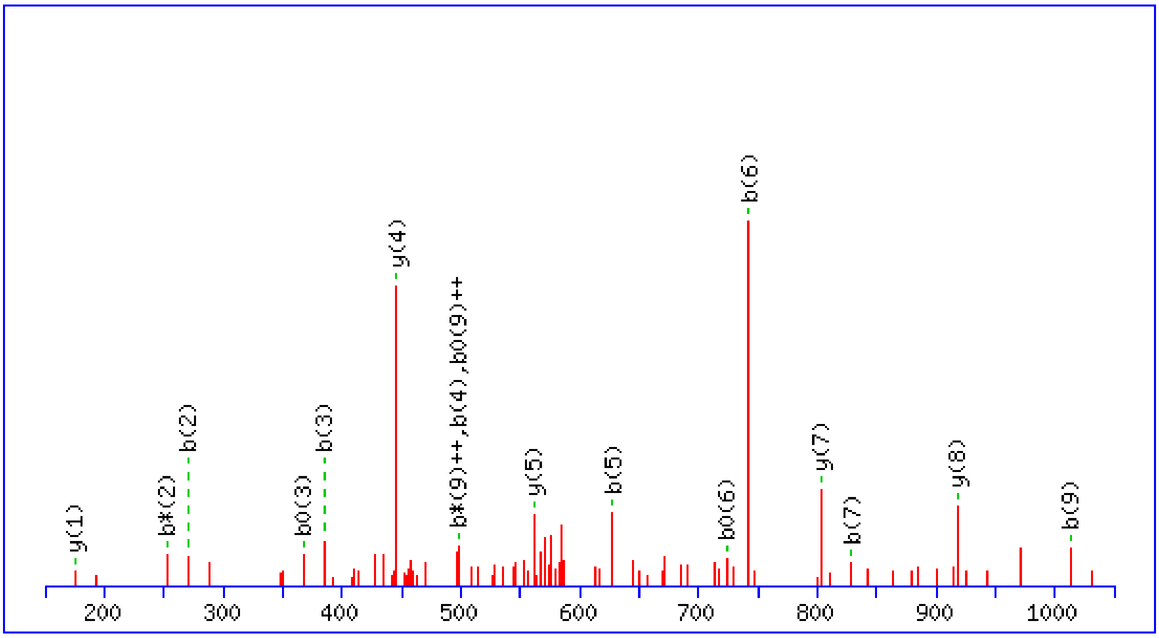

Spot no.610
Prelamin A/C
DLEDSLAR

#	b	b++	b0	b0++	Seq.	y	y++	y*	y*++	y0	y0++	#
1	116.0342	58.5207	98.0237	49.5155	D							8
2	**229.1183**	115.0628	211.1077	106.0575	L	***803.4258***	402.2165	786.3992	393.7032	785.4152	393.2112	7
3	**358.1609**	179.5841	340.1503	170.5788	E	***690.3417***	345.6745	673.3151	337.1612	672.3311	336.6692	6
4	473.1878	237.0975	455.1773	228.0923	D	***561.2991***	281.1532	544.2726	272.6399	543.2885	272.1479	5
5	**560.2198**	280.6136	542.2093	271.6083	S	***446.2722***	223.6397	429.2456	215.1264	428.2616	214.6344	4
6	673.3039	337.1556	655.2933	328.1503	L	359.2401	180.1237	342.2136	171.6104			3
7	**744.341**	372.6742	726.3305	363.6689	A	***246.1561***	123.5817	229.1295	115.0684			2
8					R	***175.119***	88.0631	158.0924	79.5498			1

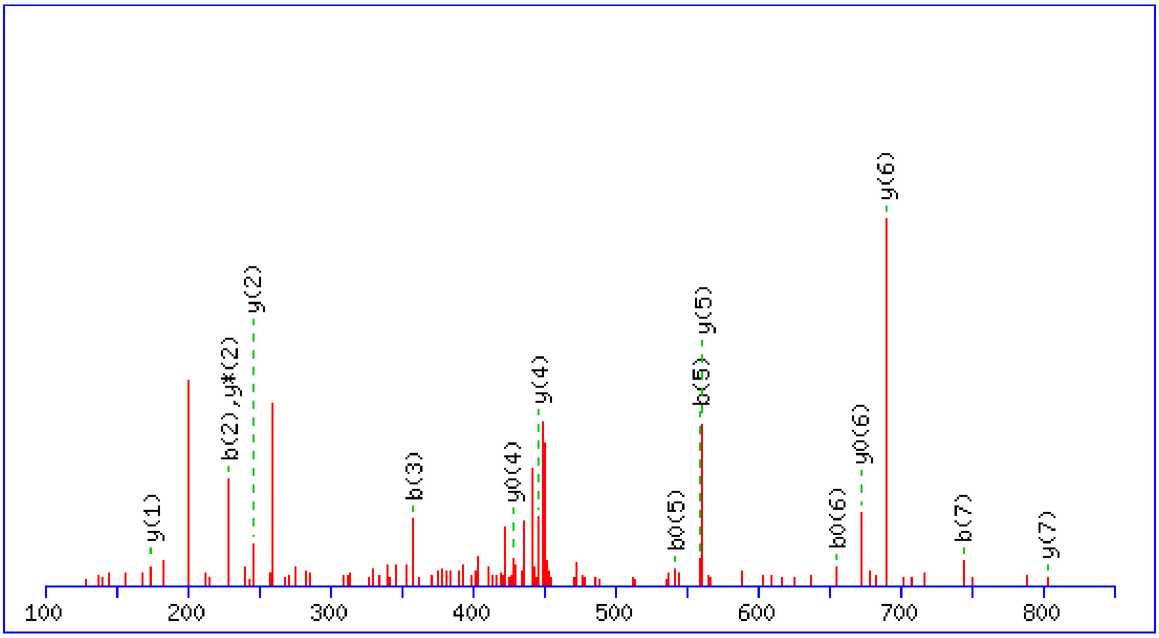

Spot no.610
Prelamin A/C
LLADKER

#	b	b++	b*	b*++	b0	b0++	Seq.	y	y++	y*	y*++	y0	y0++	#
1	114.0913	57.5493					L							7
2	**227.1754**	114.0913					L	***731.4046***	366.206	714.3781	357.6927	713.3941	357.2007	6
3	**298.2125**	149.6099					A	***618.3206***	309.6639	601.294	301.1506	600.31	300.6586	5
4	**413.2395**	207.1234			395.2289	198.1181	D	***547.2835***	274.1454	530.2569	265.6321	529.2729	265.1401	4
5	541.3344	271.1709	524.3079	262.6576	523.3239	262.1656	K	***432.2565***	216.6319	415.23	208.1186	414.2459	207.6266	3
6	**670.377**	335.6921	653.3505	327.1789	652.3665	326.6869	E	***304.1615***	152.5844	287.135	144.0711	286.151	143.5791	2
7							R	***175.119***	88.0631	158.0924	79.5498			1

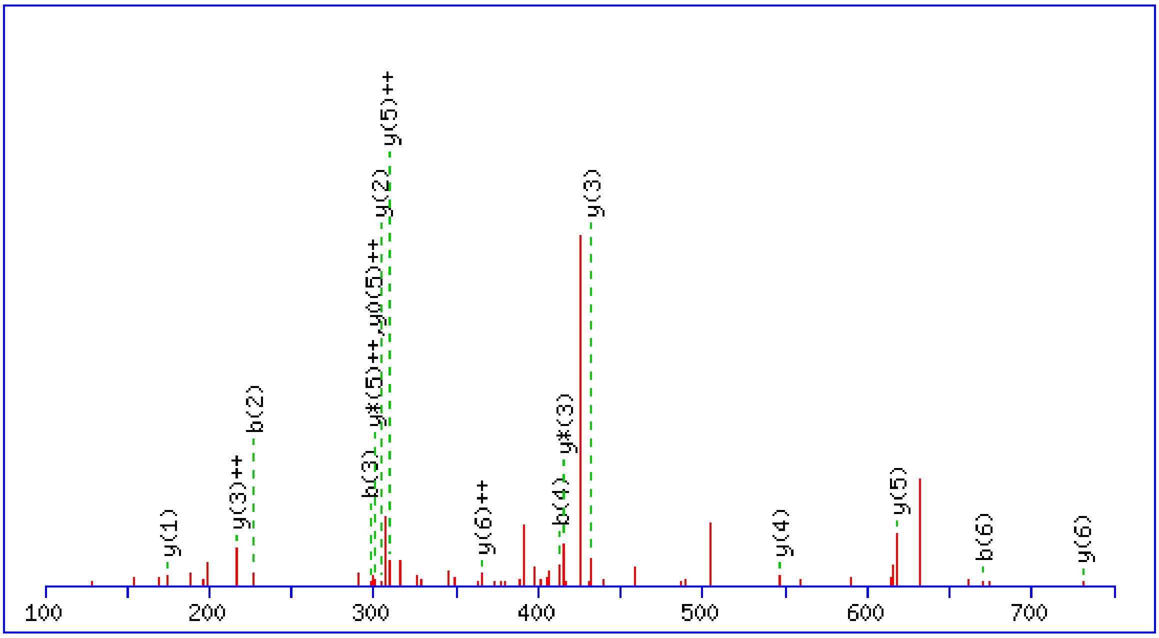

Spot no.610
Prelamin A/C
MQQQLDEYQELLDIK

#	b	b^{++}	b*	b^{*++}	b^{0}	b^{0++}	Seq.	y	y^{++}	y*	y^{*++}	y^{0}	y^{0++}	#
1	148.0427	74.525					M							15
2	276.1013	138.5543	259.0747	130.041			Q	1762.8858	881.9465	1745.8592	873.4333	1744.8752	872.9412	14
3	**404.1598**	202.5836	387.1333	194.0703			Q	1634.8272	817.9172	1617.8006	809.404	1616.8166	808.912	13
4	**532.2184**	266.6128	515.1919	258.0996			Q	***1506.7686***	753.8879	1489.7421	745.3747	1488.7581	744.8827	12
5	645.3025	323.1549	628.2759	314.6416			L	***1378.71***	689.8587	1361.6835	681.3454	1360.6995	680.8534	11
6	**760.3294**	380.6683	743.3029	372.1551	742.3189	371.6631	D	***1265.626***	633.3166	1248.5994	624.8034	1247.6154	624.3113	10
7	889.372	445.1896	872.3455	436.6764	871.3614	436.1844	E	***1150.599***	575.8032	1133.5725	567.2899	1132.5885	566.7979	9
8	1052.4353	526.7213	1035.4088	518.208	1034.4248	517.716	Y	***1021.5564***	511.2819	1004.5299	502.7686	1003.5459	502.2766	8
9	1180.4939	590.7506	1163.4674	582.2373	1162.4834	581.7453	Q	858.4931	429.7502	841.4666	421.2369	840.4825	420.7449	7
10	1309.5365	655.2719	1292.51	646.7586	1291.5259	646.2666	E	730.4345	365.7209	713.408	357.2076	712.424	356.7156	6
11	1422.6206	711.8139	1405.594	703.3007	1404.61	702.8086	L	***601.3919***	301.1996	584.3654	292.6863	583.3814	292.1943	5
12	**1535.7046**	768.356	1518.6781	759.8427	1517.6941	759.3507	L	488.3079	244.6576	471.2813	236.1443	470.2973	235.6523	4
13	**1650.7316**	825.8694	1633.705	817.3562	1632.721	816.8641	D	***375.2238***	188.1155	358.1973	179.6023	357.2132	179.1103	3
14	1763.8156	882.4115	1746.7891	873.8982	1745.8051	873.4062	I	260.1969	130.6021	243.1703	122.0888			2
15							K	147.1128	74.06	130.0863	65.5468			1

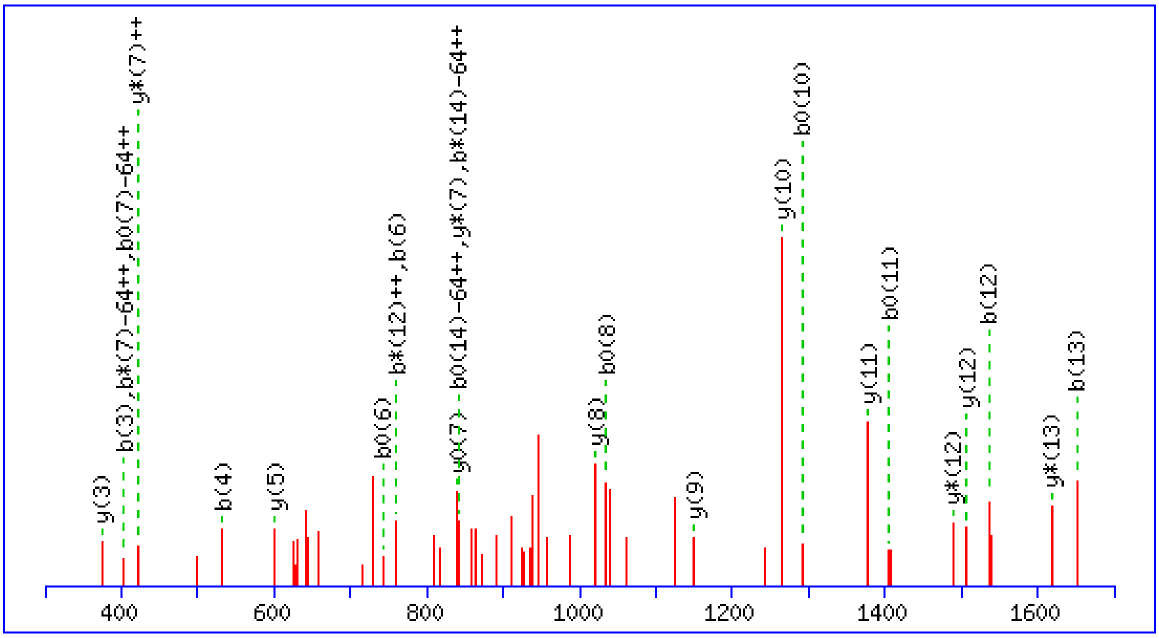

Spot no.610
Prelamin A/C
LLEGEEER

#	b	b++	b0	b0++	Seq.	y	y++	y*	y*++	y0	y0++	#
1	114.0913	57.5493			L							8
2	227.1754	114.0913			L	861.3949	431.2011	844.3683	422.6878	843.3843	422.1958	7
3	356.218	178.6126	338.2074	169.6074	E	748.3108	374.659	731.2842	366.1458	730.3002	365.6537	6
4	413.2395	207.1234	395.2289	198.1181	G	619.2682	310.1377	602.2416	301.6245	601.2576	301.1325	5
5	542.2821	271.6447	524.2715	262.6394	E	562.2467	281.627	545.2202	273.1137	544.2362	272.6217	4
6	671.3246	336.166	653.3141	327.1607	E	433.2041	217.1057	416.1776	208.5924	415.1936	208.1004	3
7	800.3672	400.6873	782.3567	391.682	E	304.1615	152.5844	287.135	144.0711	286.151	143.5791	2
8					R	175.119	88.0631	158.0924	79.5498			1

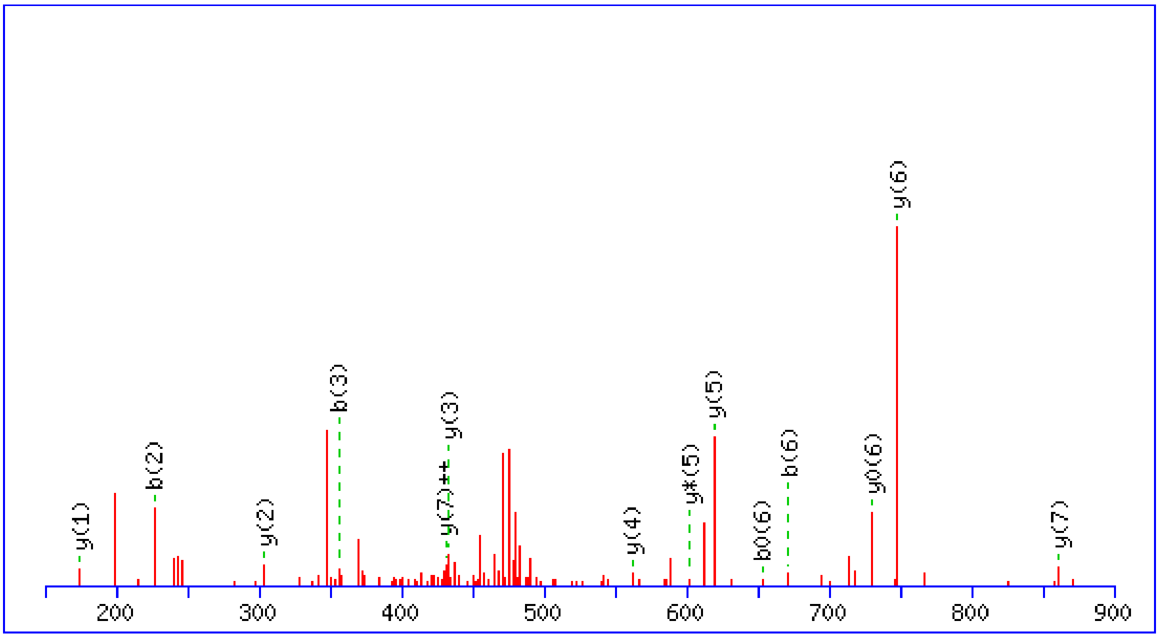

Spot no.610
Prelamin A/C
ASSHSSQTQGTGSITK

#	b	b++	b*	b*++	b0	b0++	Seq.	y	y++	y*	y*++	y0	y0++	#
1	72.0444	36.5258					A							16
2	159.0764	80.0418			141.0659	71.0366	S	1505.719	753.3632	1488.6925	744.8499	1487.7085	744.3579	15
3	246.1084	123.5579			228.0979	114.5526	S	1418.687	709.8471	1401.6605	701.3339	1400.6764	700.8419	14
4	383.1674	192.0873			365.1568	183.082	H	1331.655	666.3311	1314.6284	657.8179	1313.6444	657.3258	13
5	***470.1994***	235.6033			452.1888	226.598	S	***1194.5961***	597.8017	1177.5695	589.2884	1176.5855	588.7964	12
6	557.2314	279.1193			539.2208	270.1141	S	***1107.564***	554.2857	1090.5375	545.7724	1089.5535	545.2804	11
7	685.29	343.1486	668.2634	334.6354	667.2794	334.1434	Q	1020.532	510.7696	1003.5055	502.2564	1002.5215	501.7644	10
8	786.3377	393.6725	769.3111	385.1592	768.3271	384.6672	T	***892.4734***	446.7404	875.4469	438.2271	874.4629	437.7351	9
9	914.3962	457.7018	897.3697	449.1885	896.3857	448.6965	Q	791.4258	396.2165	774.3992	387.7032	773.4152	387.2112	8
10	971.4177	486.2125	954.3912	477.6992	953.4071	477.2072	G	663.3672	332.1872	646.3406	323.674	645.3566	323.1819	7
11	1072.4654	536.7363	1055.4388	528.2231	1054.4548	527.7311	T	606.3457	303.6765	589.3192	295.1632	588.3352	294.6712	6
12	1129.4869	565.2471	1112.4603	556.7338	1111.4763	556.2418	G	505.298	253.1527	488.2715	244.6394	487.2875	244.1474	5
13	1216.5189	608.7631	1199.4923	600.2498	1198.5083	599.7578	S	448.2766	224.6419	431.25	216.1287	430.266	215.6366	4
14	***1329.6029***	665.3051	1312.5764	656.7918	1311.5924	656.2998	I	361.2445	181.1259	344.218	172.6126	343.234	172.1206	3
15	1430.6506	715.829	1413.6241	707.3157	1412.6401	706.8237	T	248.1605	124.5839	231.1339	116.0706	230.1499	115.5786	2
16							K	147.1128	74.06	130.0863	65.5468			1

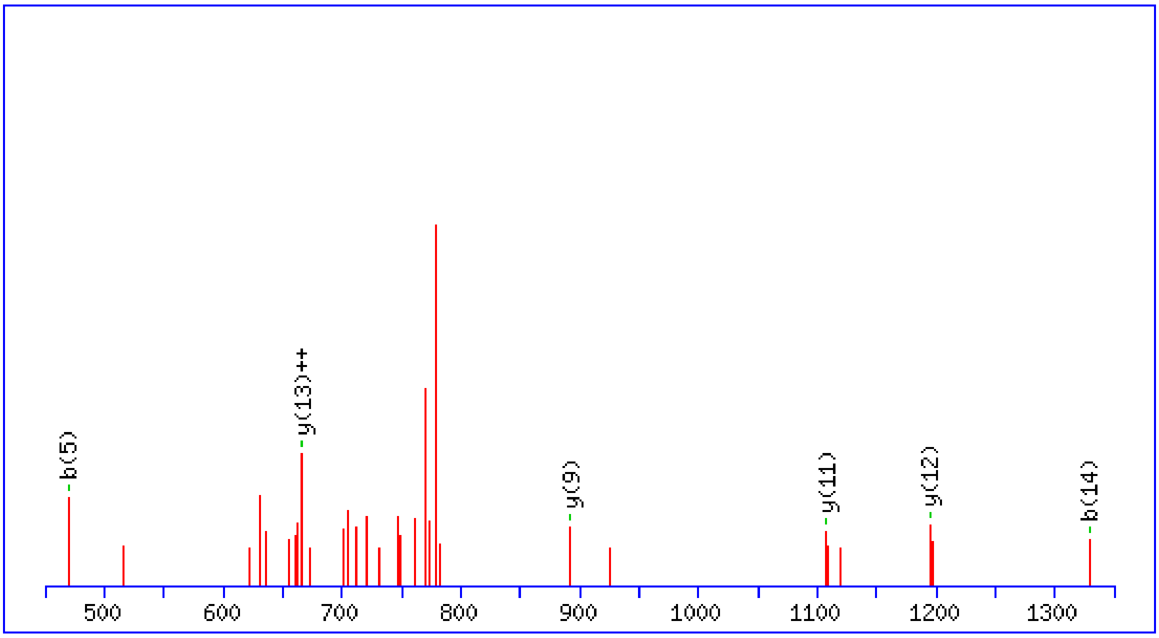

Spot no.610
Prelamin A/C
VAVEEVDEEGK

#	b	b++	b0	b0++	Seq.	y	y++	y*	y*++	y0	y0++	#
1	100.0757	50.5415			V							11
2	171.1128	86.06			A	1104.5055	552.7564	1087.479	544.2431	1086.495	543.7511	10
3	270.1812	135.5942			V	1033.4684	517.2378	1016.4419	508.7246	1015.4578	508.2326	9
4	399.2238	200.1155	381.2132	191.1103	E	934.4	467.7036	917.3734	459.1904	916.3894	458.6984	8
5	528.2664	264.6368	510.2558	255.6316	E	805.3574	403.1823	788.3309	394.6691	787.3468	394.1771	7
6	627.3348	314.171	609.3243	305.1658	V	676.3148	338.661	659.2883	330.1478	658.3042	329.6558	6
7	742.3618	371.6845	724.3512	362.6792	D	577.2464	289.1268	560.2198	280.6136	559.2358	280.1216	5
8	871.4044	436.2058	853.3938	427.2005	E	462.2195	231.6134	445.1929	223.1001	444.2089	222.6081	4
9	1000.4469	500.7271	982.4364	491.7218	E	333.1769	167.0921	316.1503	158.5788	315.1663	158.0868	3
10	1057.4684	529.2378	1039.4578	520.2326	G	204.1343	102.5708	187.1077	94.0575			2
11					K	147.1128	74.06	130.0863	65.5468			1

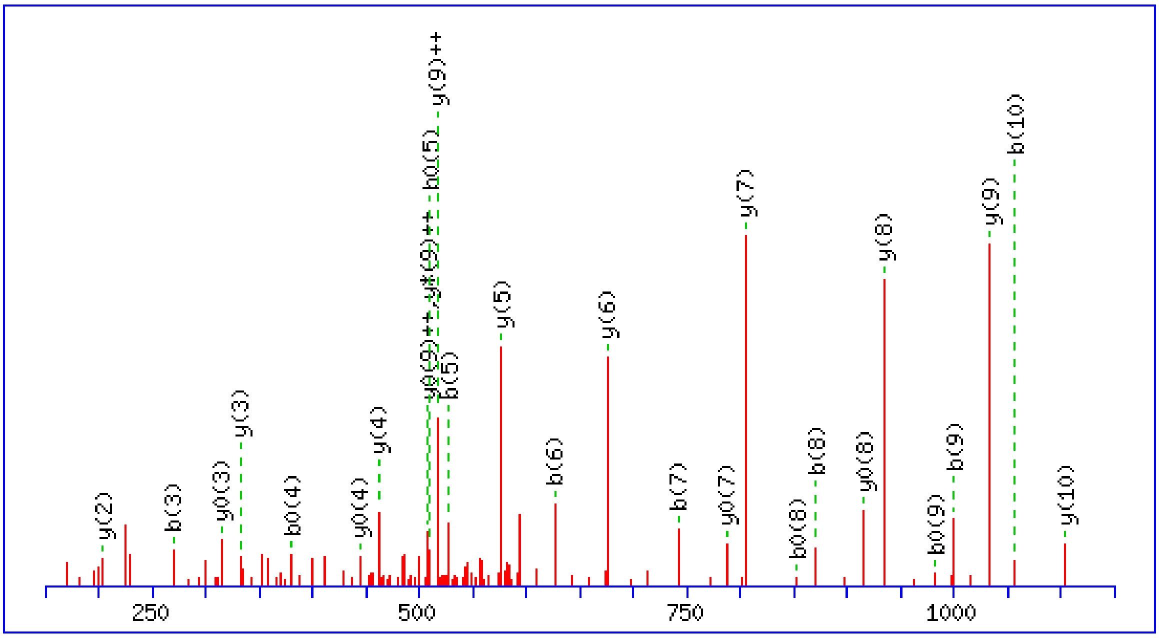

Spot no.610
Prelamin A/C
TALINSTGEEVAMR

#	b	b^{++}	b*	b*$^{++}$	b^{0}	b^{0++}	Seq.	y	y^{++}	y*	y*$^{++}$	y^{0}	y^{0++}	#
**1**	102.055	51.5311			84.0444	42.5258	**T**							**14**
**2**	173.0921	87.0497			155.0815	78.0444	**A**	1406.6944	703.8508	1389.6679	695.3376	1388.6838	694.8456	**13**
**3**	286.1761	143.5917			268.1656	134.5864	**L**	1335.6573	668.3323	1318.6307	659.819	1317.6467	659.327	**12**
**4**	399.2602	200.1337			381.2496	191.1285	**I**	***1222.5732***	611.7903	1205.5467	603.277	1204.5627	602.785	**11**
**5**	513.3031	257.1552	496.2766	248.6419	495.2926	248.1499	**N**	***1109.4892***	555.2482	1092.4626	546.7349	1091.4786	546.2429	**10**
**6**	600.3352	300.6712	583.3086	292.1579	582.3246	291.6659	**S**	***995.4462***	498.2268	978.4197	489.7135	977.4357	489.2215	**9**
**7**	701.3828	351.1951	684.3563	342.6818	683.3723	342.1898	**T**	***908.4142***	454.7107	891.3877	446.1975	890.4036	445.7055	**8**
**8**	758.4043	379.7058	741.3777	371.1925	740.3937	370.7005	**G**	***807.3665***	404.1869	790.34	395.6736	789.356	395.1816	**7**
**9**	887.4469	444.2271	870.4203	435.7138	869.4363	435.2218	**E**	750.3451	375.6762	733.3185	367.1629	732.3345	366.6709	**6**
**10**	1016.4895	508.7484	999.4629	500.2351	998.4789	499.7431	**E**	***621.3025***	311.1549	604.2759	302.6416	603.2919	302.1496	**5**
**11**	1115.5579	558.2826	1098.5313	549.7693	1097.5473	549.2773	**V**	***492.2599***	246.6336	475.2333	238.1203			**4**
**12**	1186.595	593.8011	1169.5685	585.2879	1168.5844	584.7959	**A**	***393.1915***	197.0994	376.1649	188.5861			**3**
**13**	1333.6304	667.3188	1316.6039	658.8056	1315.6198	658.3136	**M**	***322.1544***	161.5808	305.1278	153.0675			**2**
**14**							**R**	175.119	88.0631	158.0924	79.5498			**1**

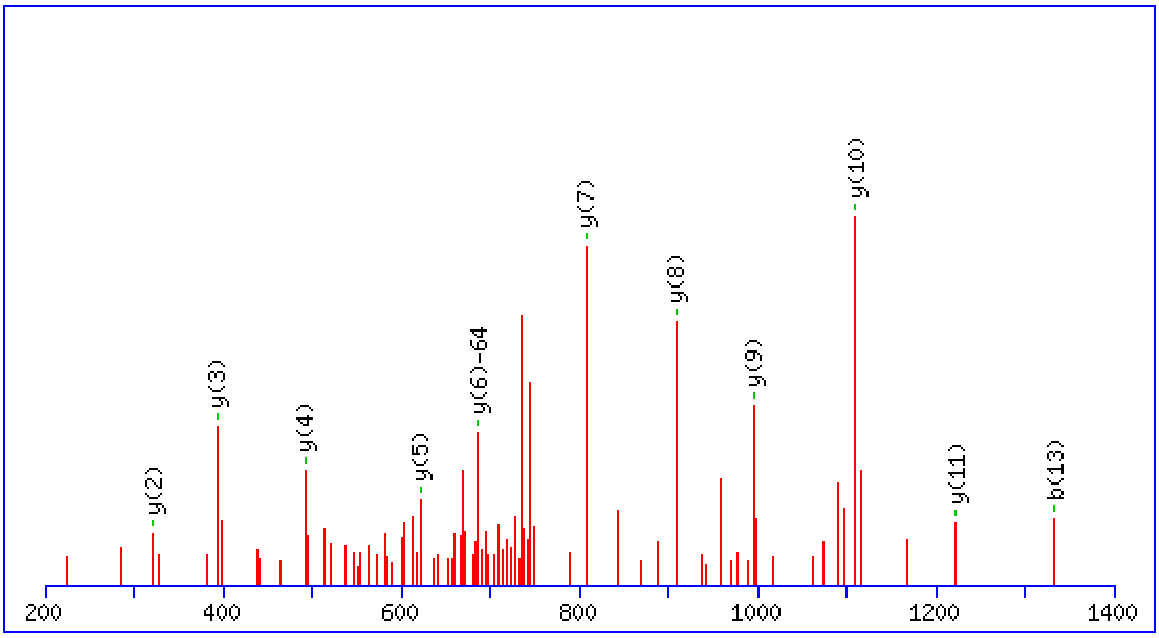

Spot no.661
Annexin A2
LSLEGDHSTPPSAYGSVK

#	b	b^{++}	b^{0}	b^{0++}	Seq.	y	y^{++}	y*	y^{*++}	y^{0}	y^{0++}	#
1	114.0913	57.5493			L							18
2	**201.1234**	101.0653	183.1128	92.06	S	1731.8184	866.4128	1714.7919	857.8996	1713.8079	857.4076	17
3	314.2074	157.6074	296.1969	148.6021	L	1644.7864	822.8968	1627.7598	814.3836	1626.7758	813.8916	16
4	443.25	222.1287	425.2395	213.1234	E	1531.7023	766.3548	1514.6758	757.8415	1513.6918	757.3495	15
5	500.2715	250.6394	482.2609	241.6341	G	1402.6597	701.8335	1385.6332	693.3202	1384.6492	692.8282	14
6	615.2984	308.1529	597.2879	299.1476	D	1345.6383	673.3228	1328.6117	664.8095	1327.6277	664.3175	13
7	752.3573	376.6823	734.3468	367.677	H	1230.6113	615.8093	1213.5848	607.296	1212.6008	606.804	12
8	839.3894	420.1983	821.3788	411.193	S	1093.5524	547.2798	1076.5259	538.7666	1075.5419	538.2746	11
9	**940.4371**	470.7222	922.4265	461.7169	T	1006.5204	503.7638	989.4938	495.2506	988.5098	494.7585	10
10	1037.4898	519.2485	1019.4792	510.2433	P	**905.4727**	453.24	888.4462	444.7267	887.4621	444.2347	9
11	1134.5426	567.7749	1116.532	558.7696	P	**808.4199**	404.7136	791.3934	396.2003	790.4094	395.7083	8
12	1221.5746	611.2909	1203.564	602.2857	S	**711.3672**	356.1872	694.3406	347.674	693.3566	347.1819	7
13	1292.6117	646.8095	1274.6012	637.8042	A	624.3352	312.6712	607.3086	304.1579	606.3246	303.6659	6
14	1455.6751	728.3412	1437.6645	719.3359	Y	553.298	277.1527	536.2715	268.6394	535.2875	268.1474	5
15	1512.6965	756.8519	1494.6859	747.8466	G	390.2347	195.621	373.2082	187.1077	372.2241	186.6157	4
16	1599.7285	800.3679	1581.718	791.3626	S	333.2132	167.1103	316.1867	158.597	315.2027	158.105	3
17	1698.797	849.9021	1680.7864	840.8968	V	246.1812	123.5942	229.1547	115.081			2
18					K	147.1128	74.06	130.0863	65.5468			1

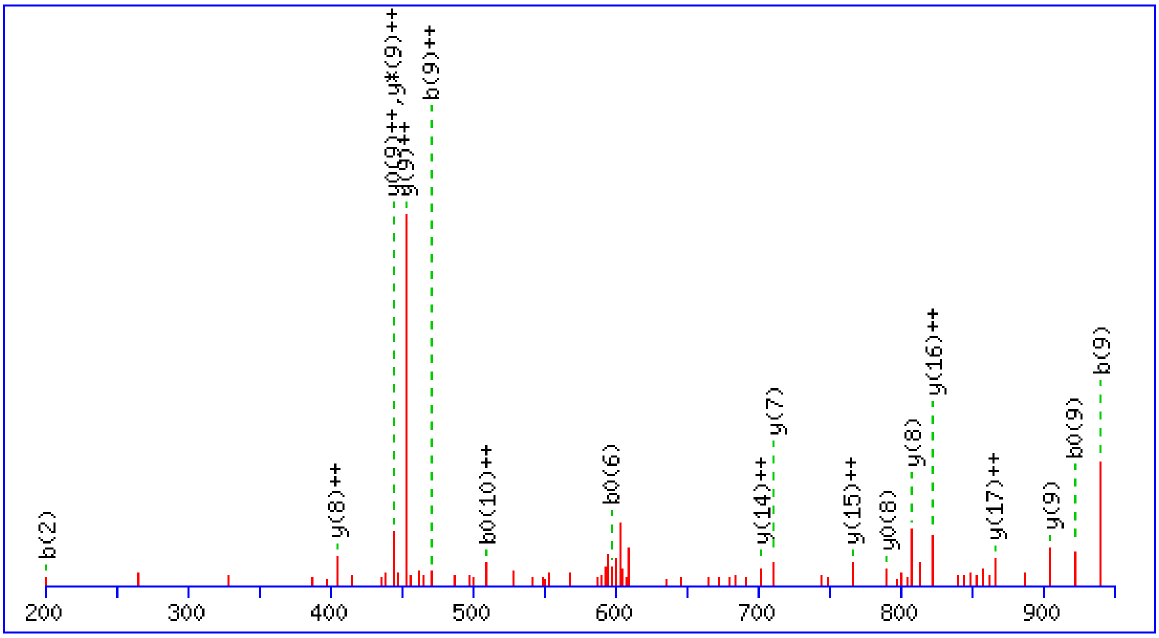

Spot no.661
Annexin A2
AYTNFDAER

#	b	b++	b*	b*++	b0	b0++	Seq.	y	y++	y*	y*++	y0	y0++	#
1	72.0444	36.5258					A							9
2	**235.1077**	118.0575					Y	1015.448	508.2276	998.4214	499.7143	997.4374	499.2223	8
3	**336.1554**	168.5813			318.1448	159.5761	T	***852.3846***	426.6959	835.3581	418.1827	834.3741	417.6907	7
4	450.1983	225.6028	433.1718	217.0895	432.1878	216.5975	N	***751.3369***	376.1721	734.3104	367.6588	733.3264	367.1668	6
5	597.2667	299.137	580.2402	290.6237	579.2562	290.1317	F	***637.294***	319.1506	620.2675	310.6374	619.2835	310.1454	5
6	712.2937	356.6505	695.2671	348.1372	694.2831	347.6452	D	***490.2256***	245.6164	473.1991	237.1032	472.215	236.6112	4
7	783.3308	392.169	766.3042	383.6558	765.3202	383.1638	A	***375.1987***	188.103	358.1721	179.5897	357.1881	179.0977	3
8	**912.3734**	456.6903	895.3468	448.1771	894.3628	447.685	E	***304.1615***	152.5844	287.135	144.0711	286.151	143.5791	2
9							R	175.119	88.0631	158.0924	79.5498			1

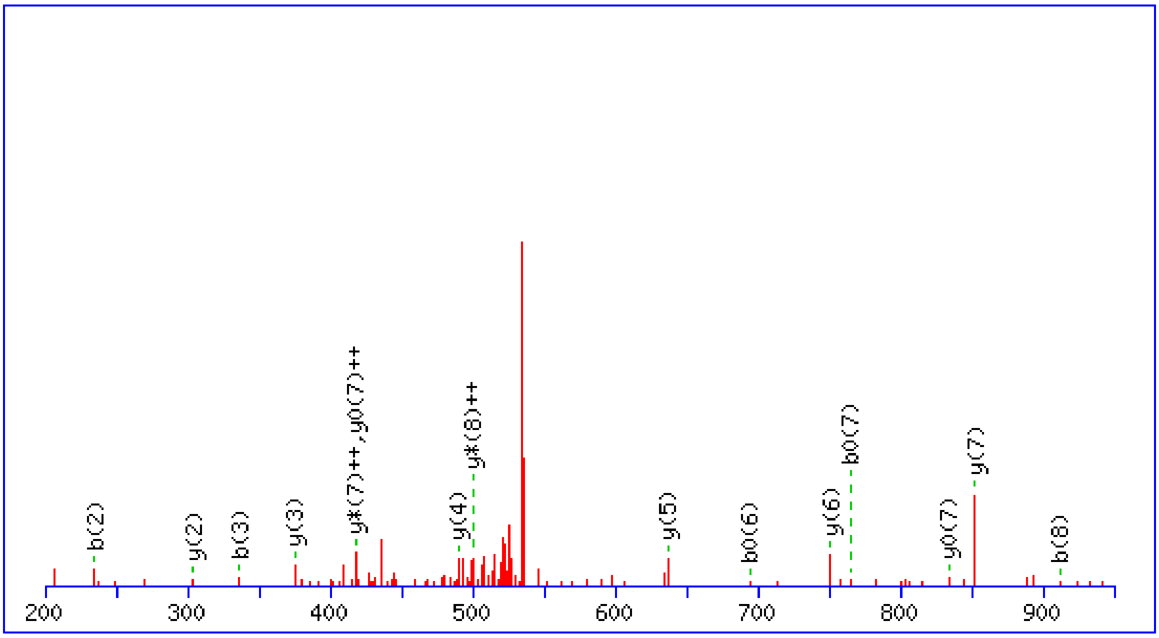

Spot no.661
Annexin A2
DALNIETAIK

#	b	b^{++}	b*	b^{*++}	b^{0}	b^{0++}	Seq.	y	y^{++}	y*	y^{*++}	y^{0}	y^{0++}	#
1	116.0342	58.5207			98.0237	49.5155	D							10
2	***187.0713***	94.0393			169.0608	85.034	A	972.5724	486.7898	955.5459	478.2766	954.5619	477.7846	9
3	***300.1554***	150.5813			282.1448	141.5761	L	**901.5353**	451.2713	884.5088	442.758	883.5247	442.266	8
4	***414.1983***	207.6028	397.1718	199.0895	396.1878	198.5975	N	**788.4512**	394.7293	771.4247	386.216	770.4407	385.724	7
5	***527.2824***	264.1448	510.2558	255.6316	509.2718	255.1395	I	**674.4083**	337.7078	657.3818	329.1945	656.3978	328.7025	6
6	***656.325***	328.6661	639.2984	320.1529	638.3144	319.6608	E	**561.3243**	281.1658	544.2977	272.6525	543.3137	272.1605	5
7	***757.3727***	379.19	740.3461	370.6767	739.3621	370.1847	T	**432.2817**	216.6445	415.2551	208.1312	414.2711	207.6392	4
8	***828.4098***	414.7085	811.3832	406.1953	810.3992	405.7032	A	**331.234**	166.1206	314.2074	157.6074			3
9	***941.4938***	471.2506	924.4673	462.7373	923.4833	462.2453	I	**260.1969**	130.6021	243.1703	122.0888			2
10							K	147.1128	74.06	130.0863	65.5468			1

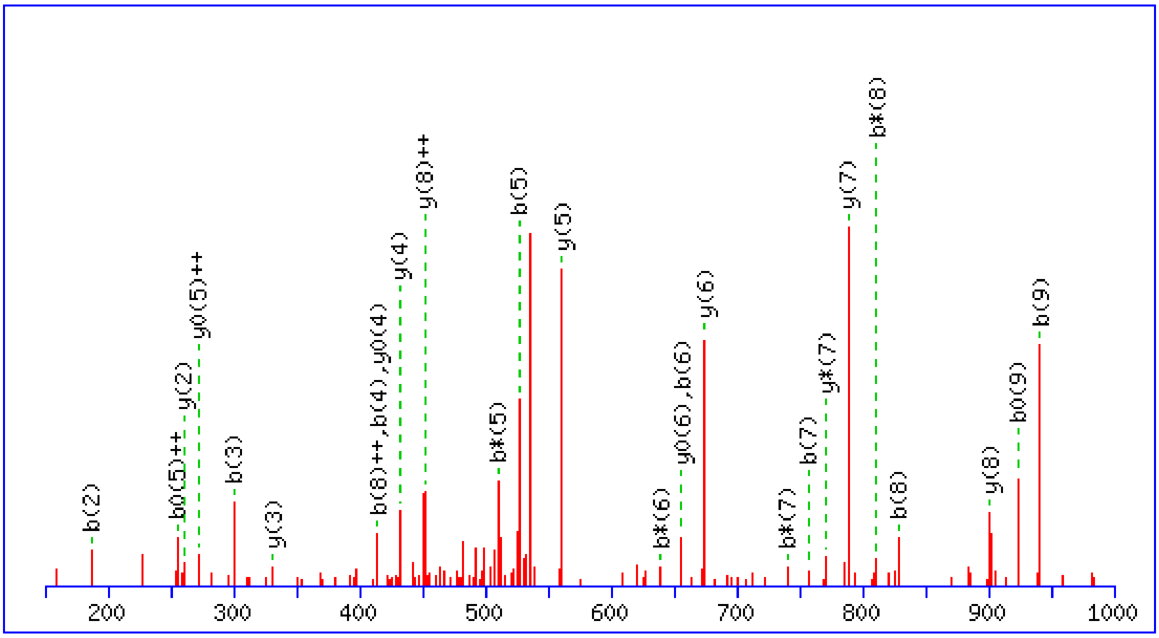

Spot no.661
Annexin A2
GVDEVTIVNILTNR

#	b	b⁺⁺	b*	b*⁺⁺	b⁰	b⁰⁺⁺	Seq.	y	y⁺⁺	y*	y*⁺⁺	y⁰	y⁰⁺⁺	#
1	58.0287	29.518					G							14
2	157.0972	79.0522					V	1485.8271	743.4172	1468.8006	734.9039	1467.8166	734.4119	13
3	272.1241	136.5657			254.1135	127.5604	D	1386.7587	693.883	1369.7322	685.3697	1368.7482	684.8777	12
4	401.1667	201.087			383.1561	192.0817	E	1271.7318	636.3695	1254.7052	627.8563	1253.7212	627.3642	11
5	500.2351	250.6212			482.2245	241.6159	V	1142.6892	571.8482	1125.6626	563.335	1124.6786	562.8429	10
6	601.2828	301.145			583.2722	292.1397	T	1043.6208	522.314	1026.5942	513.8007	1025.6102	513.3087	9
7	714.3668	357.6871			696.3563	348.6818	I	942.5731	471.7902	925.5465	463.2769	924.5625	462.7849	8
8	813.4353	407.2213			795.4247	398.216	V	829.489	415.2482	812.4625	406.7349	811.4785	406.2429	7
9	927.4782	464.2427	910.4516	455.7295	909.4676	455.2374	N	730.4206	365.7139	713.3941	357.2007	712.41	356.7087	6
10	1040.5623	520.7848	1023.5357	512.2715	1022.5517	511.7795	I	616.3777	308.6925	599.3511	300.1792	598.3671	299.6872	5
11	1153.6463	577.3268	1136.6198	568.8135	1135.6358	568.3215	L	503.2936	252.1504	486.2671	243.6372	485.2831	243.1452	4
12	1254.694	627.8506	1237.6674	619.3374	1236.6834	618.8454	T	390.2096	195.6084	373.183	187.0951	372.199	186.6031	3
13	1368.7369	684.8721	1351.7104	676.3588	1350.7264	675.8668	N	289.1619	145.0846	272.1353	136.5713			2
14							R	175.119	88.0631	158.0924	79.5498			1

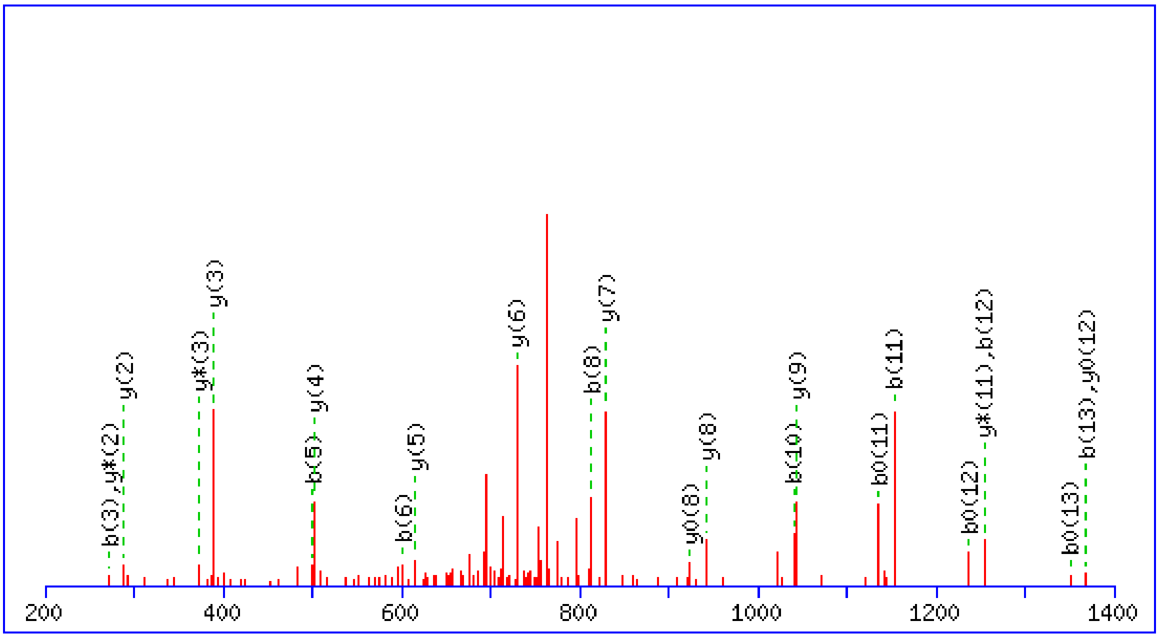

Spot no.661
Annexin A2
QDIAFAYQR

#	b	b++	b*	b*++	b0	b0++	Seq.	y	y++	y*	y*++	y0	y0++	#
1	129.0659	65.0366	112.0393	56.5233			Q							9
2	**244.0928**	122.55	227.0662	114.0368	226.0822	113.5448	D	983.4945	492.2509	966.468	483.7376	965.4839	483.2456	8
3	**357.1769**	179.0921	340.1503	170.5788	339.1663	170.0868	I	***868.4676***	434.7374	851.441	426.2241			7
4	428.214	214.6106	411.1874	206.0974	410.2034	205.6053	A	***755.3835***	378.1954	738.357	369.6821			6
5	**575.2824**	288.1448	558.2558	279.6316	557.2718	279.1396	F	***684.3464***	342.6768	667.3198	334.1636			5
6	646.3195	323.6634	629.293	315.1501	628.3089	314.6581	A	***537.278***	269.1426	520.2514	260.6294			4
7	**809.3828**	405.1951	792.3563	396.6818	791.3723	396.1898	Y	***466.2409***	233.6241	449.2143	225.1108			3
8	937.4414	469.2243	920.4149	460.7111	919.4308	460.2191	Q	***303.1775***	152.0924	286.151	143.5791			2
9							R	***175.119***	88.0631	158.0924	79.5498			1

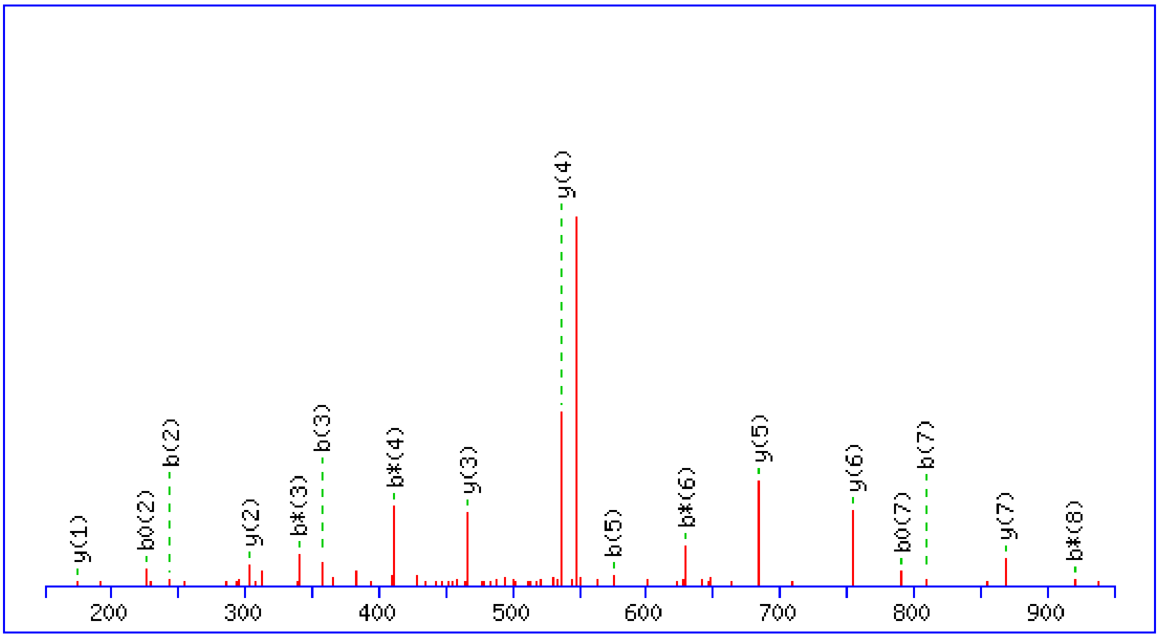

Spot no.661
Annexin A2
TPAQYDASELK

#	b	b++	b*	b*++	b0	b0++	Seq.	y	y++	y*	y*++	y0	y0++	#
1	102.055	51.5311			84.0444	42.5258	T							11
2	199.1077	100.0575			181.0972	91.0522	P	***1121.5473***	561.2773	1104.5208	552.764	1103.5368	552.272	10
3	**270.1448**	135.5761			252.1343	126.5708	A	***1024.4946***	512.7509	1007.468	504.2376	1006.484	503.7456	9
4	**398.2034**	199.6053	381.1769	191.0921	380.1928	190.6001	Q	***953.4575***	477.2324	936.4309	468.7191	935.4469	468.2271	8
5	**561.2667**	281.137	544.2402	272.6237	543.2562	272.1317	Y	***825.3989***	413.2031	808.3723	404.6898	807.3883	404.1978	7
6	**676.2937**	338.6505	659.2671	330.1372	658.2831	329.6452	D	***662.3355***	331.6714	645.309	323.1581	644.325	322.6661	6
7	747.3308	374.169	730.3042	365.6558	729.3202	365.1638	A	***547.3086***	274.1579	530.2821	265.6447	529.298	265.1527	5
8	834.3628	417.6851	817.3363	409.1718	816.3523	408.6798	S	***476.2715***	238.6394	459.2449	230.1261	458.2609	229.6341	4
9	963.4054	482.2063	946.3789	473.6931	945.3949	473.2011	E	389.2395	195.1234	372.2129	186.6101	371.2289	186.1181	3
10	1076.4895	538.7484	1059.4629	530.2351	1058.4789	529.7431	L	***260.1969***	130.6021	243.1703	122.0888			2
11							K	147.1128	74.06	130.0863	65.5468			1

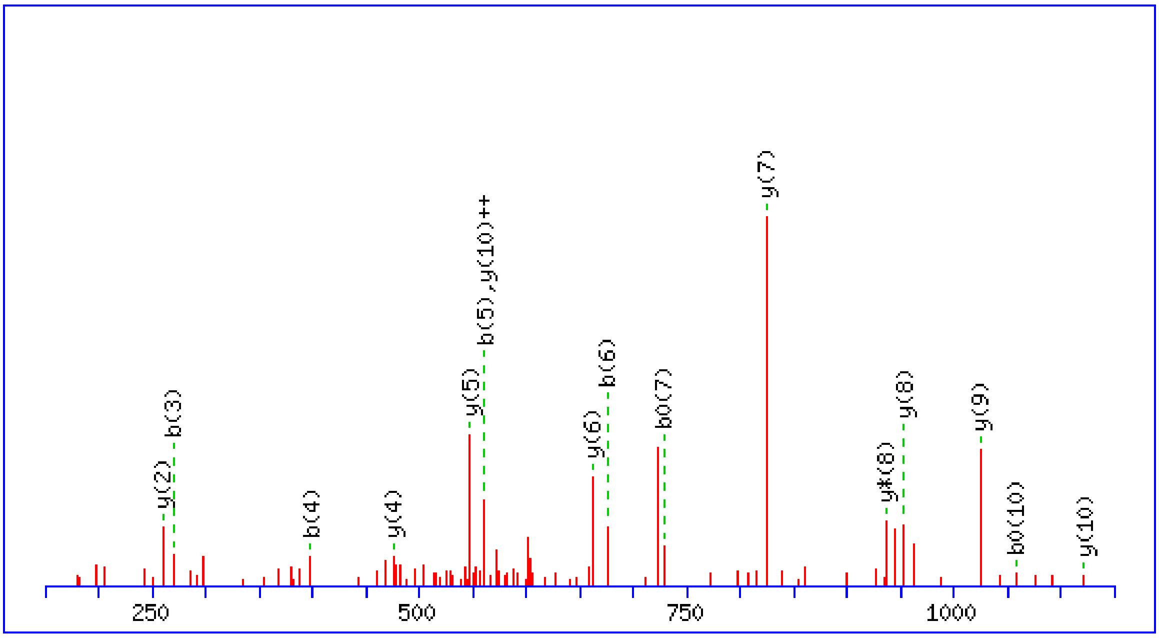

Spot no.661
Annexin A2
GLGTDEDSLIEIICSR

#	b	b^{++}	b^{0}	b^{0++}	Seq.	y	y^{++}	y*	y^{*++}	y^{0}	y^{0++}	#
**1**	58.0287	29.518			**G**							**16**
**2**	171.1128	86.06			**L**	1720.8422	860.9247	1703.8156	852.4115	1702.8316	851.9195	**15**
**3**	228.1343	114.5708			**G**	1607.7581	804.3827	1590.7316	795.8694	1589.7476	795.3774	**14**
**4**	329.1819	165.0946	311.1714	156.0893	**T**	1550.7367	775.872	1533.7101	767.3587	1532.7261	766.8667	**13**
**5**	444.2089	222.6081	426.1983	213.6028	**D**	1449.689	725.3481	1432.6624	716.8349	1431.6784	716.3428	**12**
**6**	573.2515	287.1294	555.2409	278.1241	**E**	1334.662	667.8347	1317.6355	659.3214	1316.6515	658.8294	**11**
**7**	688.2784	344.6429	670.2679	335.6376	**D**	***1205.6194***	603.3134	1188.5929	594.8001	1187.6089	594.3081	**10**
**8**	775.3105	388.1589	757.2999	379.1536	**S**	1090.5925	545.7999	1073.566	537.2866	1072.5819	536.7946	**9**
**9**	888.3945	444.7009	870.384	435.6956	**L**	1003.5605	502.2839	986.5339	493.7706	985.5499	493.2786	**8**
**10**	1001.4786	501.2429	983.468	492.2376	**I**	890.4764	445.7418	873.4499	437.2286	872.4658	436.7366	**7**
**11**	1130.5212	565.7642	1112.5106	556.7589	**E**	777.3924	389.1998	760.3658	380.6865	759.3818	380.1945	**6**
**12**	1243.6052	622.3063	1225.5947	613.301	**I**	***648.3498***	324.6785	631.3232	316.1652	630.3392	315.6732	**5**
**13**	1356.6893	678.8483	1338.6787	669.843	**I**	***535.2657***	268.1365	518.2391	259.6232	517.2551	259.1312	**4**
**14**	1516.72	758.8636	1498.7094	749.8583	**C**	***422.1816***	211.5945	405.1551	203.0812	404.1711	202.5892	**3**
**15**	1603.752	802.3796	1585.7414	793.3743	**S**	262.151	131.5791	245.1244	123.0659	244.1404	122.5738	**2**
**16**					**R**	175.119	88.0631	158.0924	79.5498			**1**

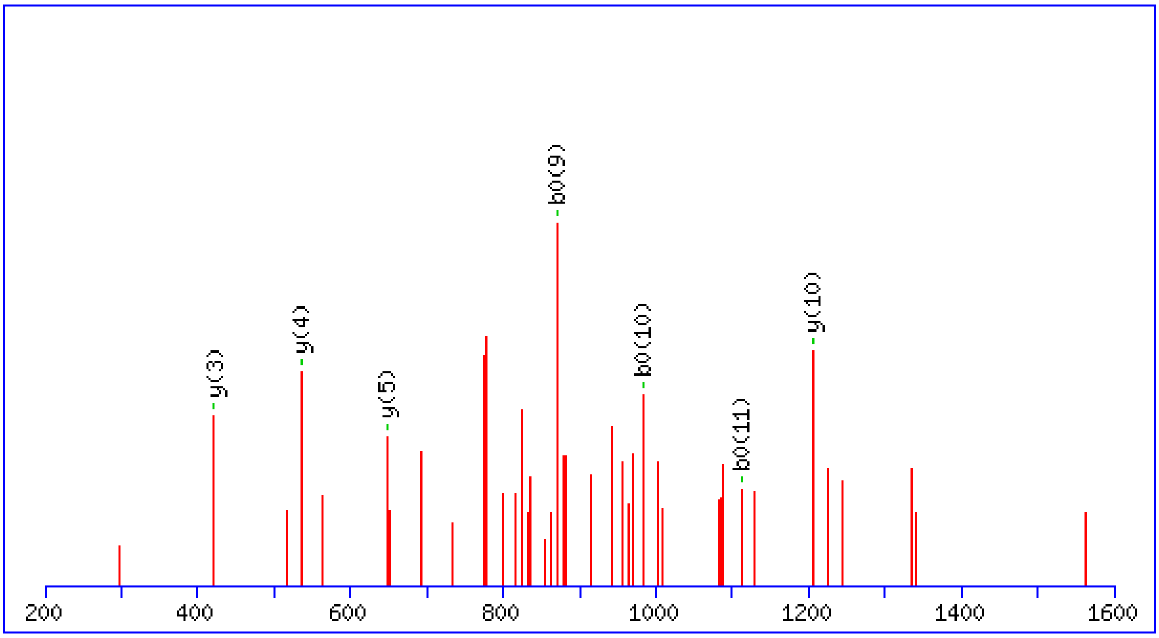

Spot no.661
Annexin A2
TNQELQEINR

#	b	b^{++}	b*	b^{*++}	b^0	b^{0++}	Seq.	y	y^{++}	y*	y^{*++}	y^0	y^{0++}	#
1	102.055	51.5311			84.0444	42.5258	T							10
2	216.0979	108.5526	199.0713	100.0393	198.0873	99.5473	N	1143.5753	572.2913	1126.5487	563.778	1125.5647	563.286	9
3	344.1565	172.5819	327.1299	164.0686	326.1459	163.5766	Q	***1029.5323***	515.2698	1012.5058	506.7565	1011.5218	506.2645	8
4	**473.1991**	237.1032	456.1725	228.5899	455.1885	228.0979	E	***901.4738***	451.2405	884.4472	442.7272	883.4632	442.2352	7
5	586.2831	293.6452	569.2566	285.1319	568.2726	284.6399	L	***772.4312***	386.7192	755.4046	378.206	754.4206	377.7139	6
6	**714.3417**	357.6745	697.3151	349.1612	696.3311	348.6692	Q	***659.3471***	330.1772	642.3206	321.6639	641.3365	321.1719	5
7	**843.3843**	422.1958	826.3577	413.6825	825.3737	413.1905	E	***531.2885***	266.1479	514.262	257.6346	513.278	257.1426	4
8	956.4684	478.7378	939.4418	470.2245	938.4578	469.7325	I	***402.2459***	201.6266	385.2194	193.1133			3
9	1070.5113	535.7593	1053.4847	527.246	1052.5007	526.754	N	***289.1619***	145.0846	272.1353	136.5713			2
10							R	175.119	88.0631	158.0924	79.5498			1

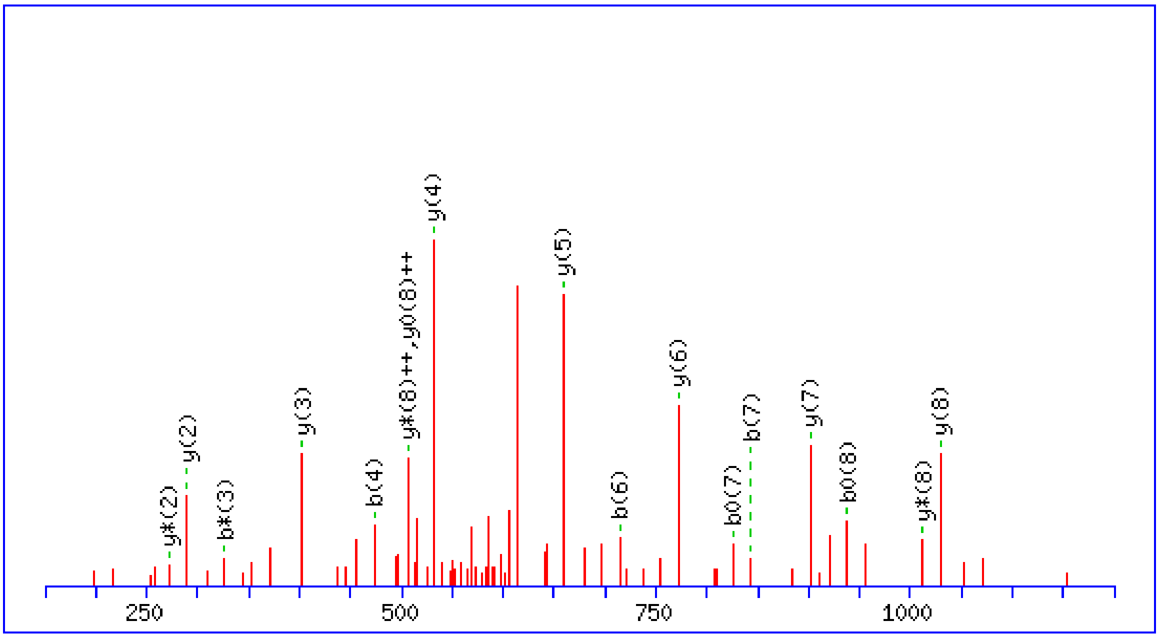

Spot no.661
Annexin A2
VYKEMYK

#	b	b++	b*	b*++	b0	b0++	Seq.	y	y++	y*	y*++	y0	y0++	#
1	100.0757	50.5415					V							7
2	263.139	132.0731					Y	***877.4124***	439.2098	860.3859	430.6966	859.4019	430.2046	6
3	391.234	196.1206	374.2074	187.6074			K	***714.3491***	357.6782	697.3225	349.1649	696.3385	348.6729	5
4	520.2766	260.6419	503.25	252.1287	502.266	251.6366	E	***586.2541***	293.6307	569.2276	285.1174	568.2436	284.6254	4
5	667.312	334.1596	650.2854	325.6464	649.3014	325.1543	M	***457.2115***	229.1094	440.185	220.5961			3
6	830.3753	415.6913	813.3488	407.178	812.3647	406.686	Y	310.1761	155.5917	293.1496	147.0784			2
7							K	***147.1128***	74.06	130.0863	65.5468			1

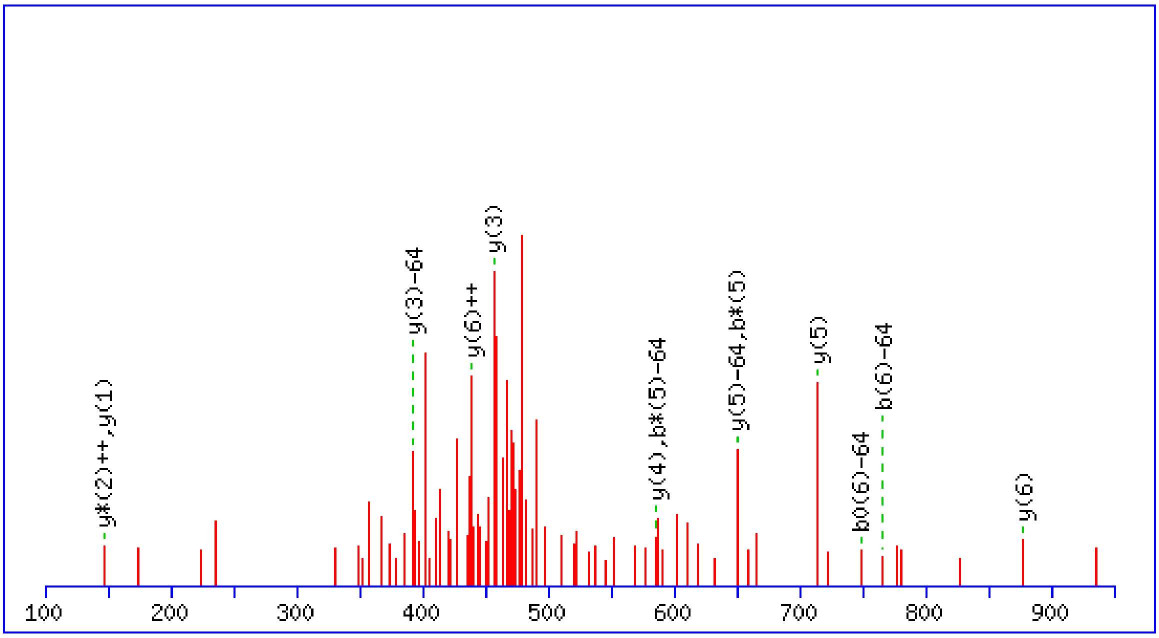

Spot no.661
Annexin A2
DIISDTSGDFR

#	b	b^{++}	b^0	b^{0++}	Seq.	y	y^{++}	y*	y^{*++}	y^0	y^{0++}	#
1	116.0342	58.5207	98.0237	49.5155	D							11
2	***229.1183***	115.0628	211.1077	106.0575	I	1110.5426	555.7749	1093.516	547.2617	1092.532	546.7696	10
3	***342.2023***	171.6048	324.1918	162.5995	I	***997.4585***	499.2329	980.432	490.7196	979.4479	490.2276	9
4	429.2344	215.1208	411.2238	206.1155	S	***884.3745***	442.6909	867.3479	434.1776	866.3639	433.6856	8
5	544.2613	272.6343	526.2508	263.629	D	***797.3424***	399.1748	780.3159	390.6616	779.3319	390.1696	7
6	645.309	323.1581	627.2984	314.1529	T	***682.3155***	341.6614	665.2889	333.1481	664.3049	332.6561	6
7	***732.341***	366.6742	714.3305	357.6689	S	***581.2678***	291.1375	564.2413	282.6243	563.2572	282.1323	5
8	789.3625	395.1849	771.3519	386.1796	G	***494.2358***	247.6215	477.2092	239.1083	476.2252	238.6162	4
9	***904.3894***	452.6984	886.3789	443.6931	D	437.2143	219.1108	420.1878	210.5975	419.2037	210.1055	3
10	***1051.4578***	526.2326	1033.4473	517.2273	F	***322.1874***	161.5973	305.1608	153.084			2
11					R	175.119	88.0631	158.0924	79.5498			1

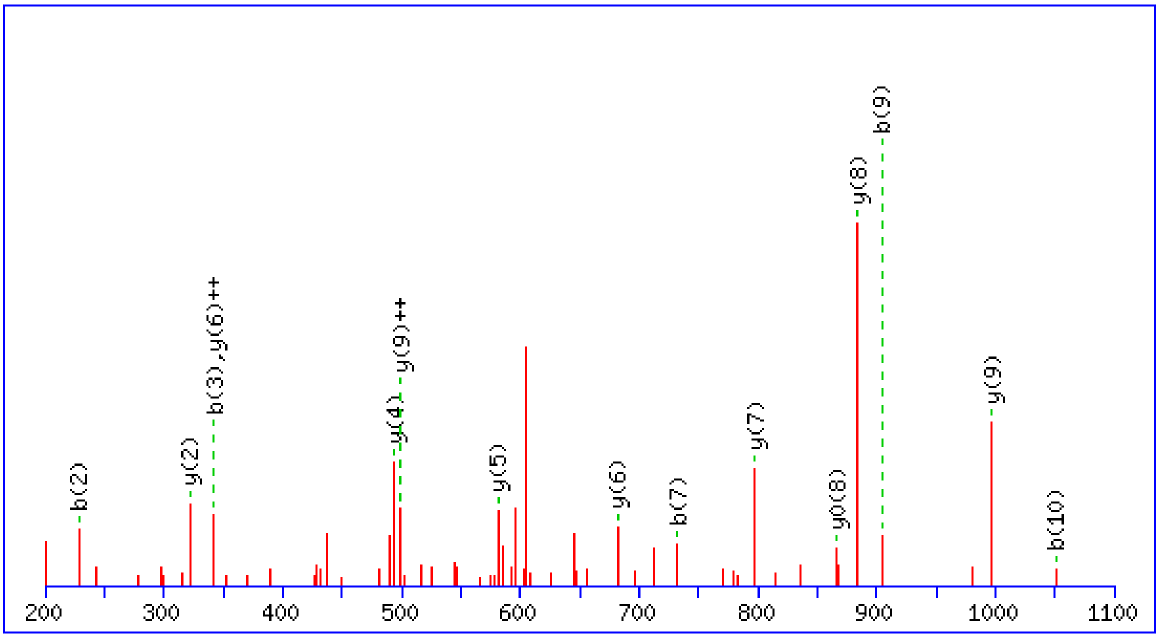

Spot no.661
Annexin A2
DIISDTSGDFRK

#	b	b^{++}	b*	b^{*++}	b^{0}	b^{0++}	Seq.	y	y^{++}	y*	y^{*++}	y^{0}	y^{0++}	#
**1**	116.0342	58.5207			98.0237	49.5155	**D**							**12**
**2**	229.1183	115.0628			211.1077	106.0575	**I**	1238.6375	619.8224	1221.611	611.3091	1220.627	610.8171	**11**
**3**	**342.2023**	171.6048			324.1918	162.5995	**I**	1125.5535	563.2804	1108.5269	554.7671	1107.5429	554.2751	**10**
**4**	429.2344	215.1208			411.2238	206.1155	**S**	***1012.4694***	506.7383	995.4429	498.2251	994.4588	497.7331	**9**
**5**	544.2613	272.6343			526.2508	263.629	**D**	***925.4374***	463.2223	908.4108	454.7091	907.4268	454.217	**8**
**6**	**645.309**	323.1581			627.2984	314.1529	**T**	***810.4104***	405.7089	793.3839	397.1956	792.3999	396.7036	**7**
**7**	732.341	366.6742			714.3305	357.6689	**S**	***709.3628***	355.185	692.3362	346.6717	691.3522	346.1797	**6**
**8**	789.3625	395.1849			771.3519	386.1796	**G**	622.3307	311.669	605.3042	303.1557	604.3202	302.6637	**5**
**9**	**904.3894**	452.6984			886.3789	443.6931	**D**	***565.3093***	283.1583	548.2827	274.645	547.2987	274.153	**4**
**10**	1051.4578	526.2326			1033.4473	517.2273	**F**	***450.2823***	225.6448	433.2558	217.1315			**3**
**11**	**1207.559**	604.2831	1190.5324	595.7698	1189.5484	595.2778	**R**	303.2139	152.1106	286.1874	143.5973			**2**
**12**							**K**	147.1128	74.06	130.0863	65.5468			**1**

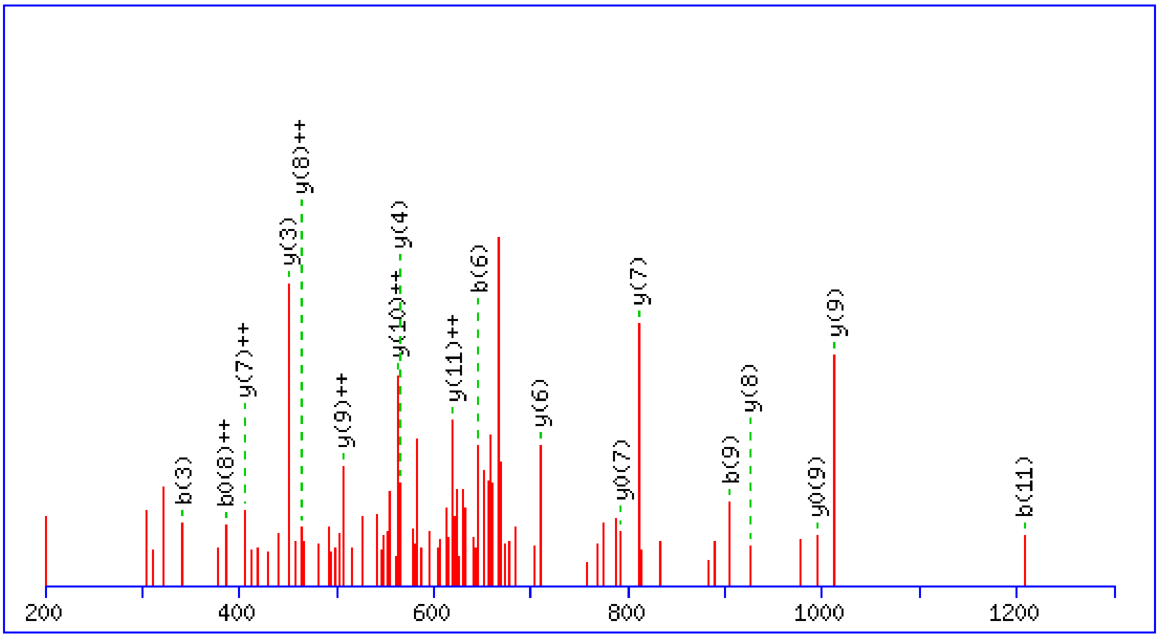

Spot no.661
Annexin A2
RAEDGSVIDYELIDQDAR

#	b	b++	b*	b*++	b0	b0++	Seq.	y	y++	y*	y*++	y0	y0++	#
1	157.1084	79.0578	140.0818	70.5446			R							18
2	228.1455	114.5764	211.119	106.0631			A	1908.8821	954.9447	1891.8556	946.4314	1890.8716	945.9394	17
3	357.1881	179.0977	340.1615	170.5844	339.1775	170.0924	E	1837.845	919.4262	1820.8185	910.9129	1819.8345	910.4209	16
4	**472.215**	236.6112	455.1885	228.0979	454.2045	227.6059	D	1708.8024	854.9049	1691.7759	846.3916	1690.7919	845.8996	15
5	529.2365	265.1219	512.21	256.6086	511.2259	256.1166	G	1593.7755	797.3914	1576.7489	788.8781	1575.7649	788.3861	14
6	616.2685	308.6379	599.242	300.1246	598.258	299.6326	S	1536.754	768.8807	1519.7275	760.3674	1518.7435	759.8754	13
7	715.3369	358.1721	698.3104	349.6588	697.3264	349.1668	V	1449.722	725.3646	1432.6955	716.8514	1431.7114	716.3594	12
8	**828.421**	414.7141	811.3945	406.2009	810.4104	405.7089	I	1350.6536	675.8304	1333.627	667.3172	1332.643	666.8251	11
9	**943.4479**	***472.2276***	926.4214	463.7143	925.4374	463.2223	D	1237.5695	619.2884	1220.543	610.7751	1219.559	610.2831	10
10	1106.5113	***553.7593***	1089.4847	545.246	1088.5007	544.754	Y	1122.5426	561.7749	1105.516	553.2617	1104.532	552.7696	9
11	**1235.5539**	***618.2806***	1218.5273	609.7673	1217.5433	609.2753	E	**959.4793**	480.2433	942.4527	471.73	941.4687	471.238	8
12	**1348.6379**	***674.8226***	1331.6114	666.3093	1330.6274	665.8173	L	830.4367	415.722	813.4101	407.2087	812.4261	406.7167	7
13	1461.722	***731.3646***	1444.6955	722.8514	1443.7114	722.3594	I	**717.3526**	359.1799	700.326	350.6667	699.342	350.1747	6
14	1576.7489	***788.8781***	1559.7224	780.3648	1558.7384	779.8728	D	**604.2685**	302.6379	587.242	294.1246	586.258	293.6326	5
15	1704.8075	***852.9074***	1687.781	844.3941	1686.797	843.9021	Q	**489.2416**	245.1244	472.215	236.6112	471.231	236.1191	4
16	1819.8345	***910.4209***	1802.8079	901.9076	1801.8239	901.4156	D	**361.183**	181.0951	344.1565	172.5819	343.1724	172.0899	3
17	1890.8716	***945.9394***	1873.845	937.4262	1872.861	936.9341	A	**246.1561**	123.5817	229.1295	115.0684			2
18							R	175.119	88.0631	158.0924	79.5498			1

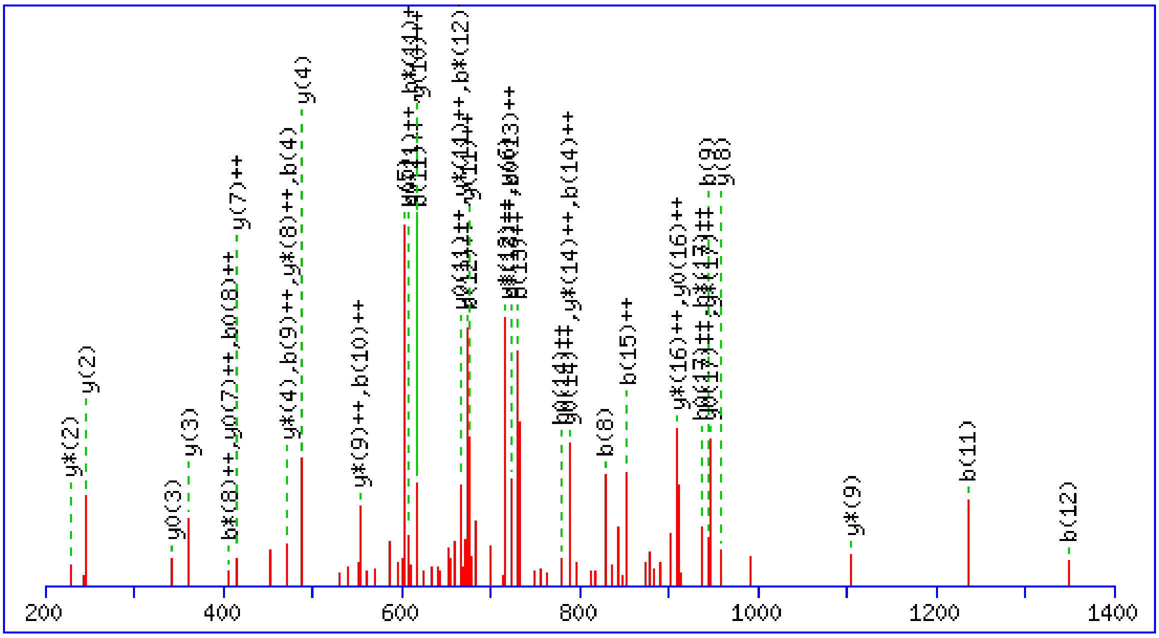

Spot no.661
Annexin A2
AEDGSVIDYELIDQDAR

#	b	b++	b*	b*++	b0	b0++	Seq.	y	y++	y*	y*++	y0	y0++	#
1	72.0444	36.5258					A							17
2	201.087	101.0471			183.0764	92.0418	E	1837.845	919.4262	1820.8185	910.9129	1819.8345	910.4209	16
3	316.1139	158.5606			298.1034	149.5553	D	1708.8024	854.9049	1691.7759	846.3916	1690.7919	845.8996	15
4	373.1354	187.0713			355.1248	178.0661	G	1593.7755	797.3914	1576.7489	788.8781	1575.7649	788.3861	14
5	460.1674	230.5873			442.1569	221.5821	S	1536.754	768.8807	1519.7275	760.3674	1518.7435	759.8754	13
6	559.2358	280.1216			541.2253	271.1163	V	***1449.722***	725.3646	1432.6955	716.8514	1431.7114	716.3594	12
7	672.3199	336.6636			654.3093	327.6583	I	***1350.6536***	675.8304	1333.627	667.3172	1332.643	666.8251	11
8	787.3468	394.1771			769.3363	385.1718	D	***1237.5695***	619.2884	1220.543	610.7751	1219.559	610.2831	10
9	950.4102	475.7087			932.3996	466.7034	Y	***1122.5426***	561.7749	1105.516	553.2617	1104.532	552.7696	9
10	1079.4528	540.23			1061.4422	531.2247	E	959.4793	480.2433	942.4527	471.73	941.4687	471.238	8
11	1192.5368	596.7721			1174.5263	587.7668	L	***830.4367***	415.722	813.4101	407.2087	812.4261	406.7167	7
12	1305.6209	653.3141			1287.6103	644.3088	I	***717.3526***	359.1799	700.326	350.6667	699.342	350.1747	6
13	1420.6478	710.8276			1402.6373	701.8223	D	***604.2685***	302.6379	587.242	294.1246	586.258	293.6326	5
14	**1548.7064**	774.8568	1531.6799	766.3436	1530.6958	765.8516	Q	***489.2416***	245.1244	472.215	236.6112	471.231	236.1191	4
15	**1663.7334**	832.3703	1646.7068	823.857	1645.7228	823.365	D	361.183	181.0951	344.1565	172.5819	343.1724	172.0899	3
16	1734.7705	867.8889	1717.7439	859.3756	1716.7599	858.8836	A	246.1561	123.5817	229.1295	115.0684			2
17							R	175.119	88.0631	158.0924	79.5498			1

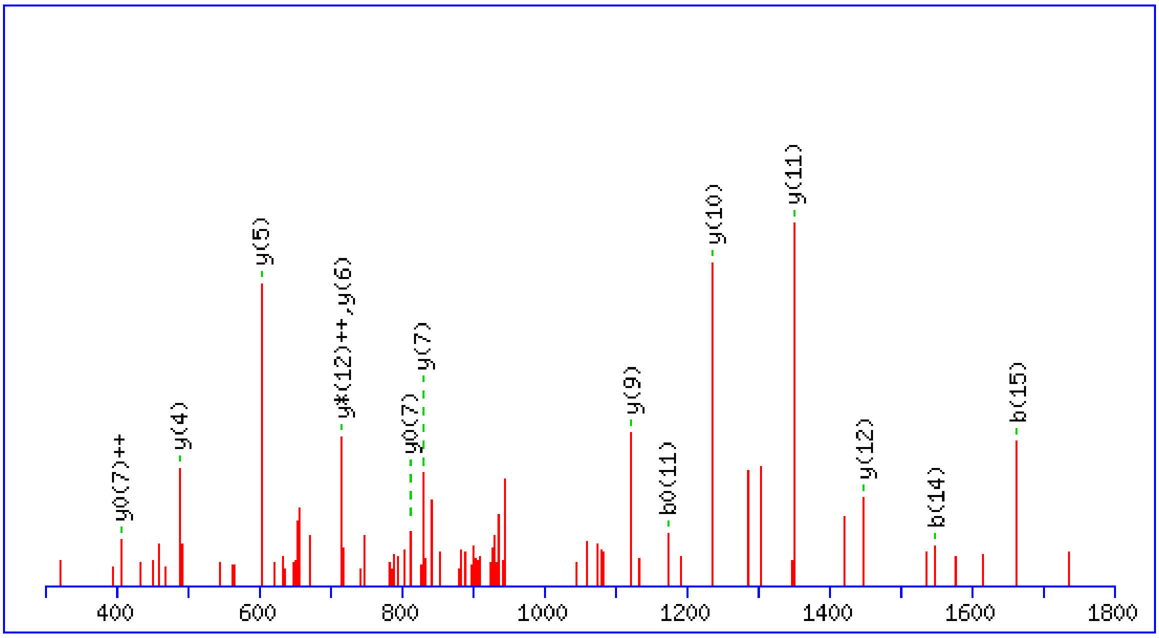

Spot no.661
Annexin A2
SVCHLQK

#	b	b^{++}	b*	b^{*++}	b^{0}	b^{0++}	Seq.	y	y^{++}	y*	y^{*++}	#
1	88.0393	44.5233			70.0287	35.518	S					7
2	***187.1077***	94.0575			169.0972	85.0522	V	784.4134	392.7103	767.3869	384.1971	6
3	***347.1384***	174.0728			329.1278	165.0675	C	**685.345**	343.1761	668.3185	334.6629	5
4	***484.1973***	242.6023			466.1867	233.597	H	**525.3144**	263.1608	508.2878	254.6475	4
5	***597.2813***	299.1443			579.2708	290.139	L	**388.2554**	194.6314	371.2289	186.1181	3
6	***725.3399***	363.1736	708.3134	354.6603	707.3294	354.1683	Q	275.1714	138.0893	258.1448	129.5761	2
7							K	147.1128	74.06	130.0863	65.5468	1

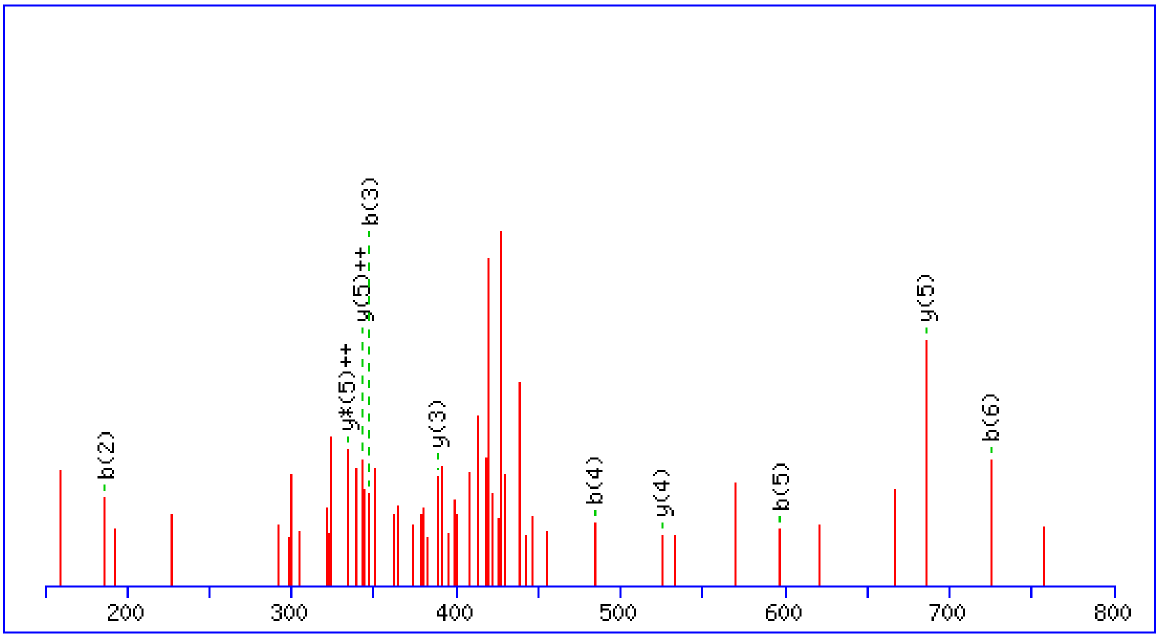

Spot no.661
Annexin A2
SYSPYDMLESIK

#	b	b^{++}	b^{0}	b^{0++}	Seq.	y	y^{++}	y*	y^{*++}	y^{0}	y^{0++}	#
1	88.0393	44.5233	70.0287	35.518	S							12
2	***251.1026***	126.055	233.0921	117.0497	Y	1361.6293	681.3183	1344.6028	672.805	1343.6188	672.313	11
3	***338.1347***	169.571	320.1241	160.5657	S	***1198.566***	599.7866	1181.5395	591.2734	1180.5555	590.7814	10
4	435.1874	218.0974	417.1769	209.0921	P	***1111.534***	556.2706	1094.5074	547.7574	1093.5234	547.2654	9
5	598.2508	299.629	580.2402	290.6237	Y	***1014.4812***	507.7443	997.4547	499.231	996.4707	498.739	8
6	713.2777	357.1425	695.2671	348.1372	D	***851.4179***	426.2126	834.3913	417.6993	833.4073	417.2073	7
7	***860.3131***	430.6602	842.3025	421.6549	M	***736.391***	368.6991	719.3644	360.1858	718.3804	359.6938	6
8	***973.3972***	487.2022	955.3866	478.1969	L	589.3556	295.1814	572.329	286.6681	571.345	286.1761	5
9	***1102.4398***	551.7235	1084.4292	542.7182	E	***476.2715***	238.6394	459.2449	230.1261	458.2609	229.6341	4
10	1189.4718	595.2395	1171.4612	586.2342	S	***347.2289***	174.1181	330.2023	165.6048	329.2183	165.1128	3
11	***1302.5558***	651.7816	1284.5453	642.7763	I	260.1969	130.6021	243.1703	122.0888			2
12					K	147.1128	74.06	130.0863	65.5468			1

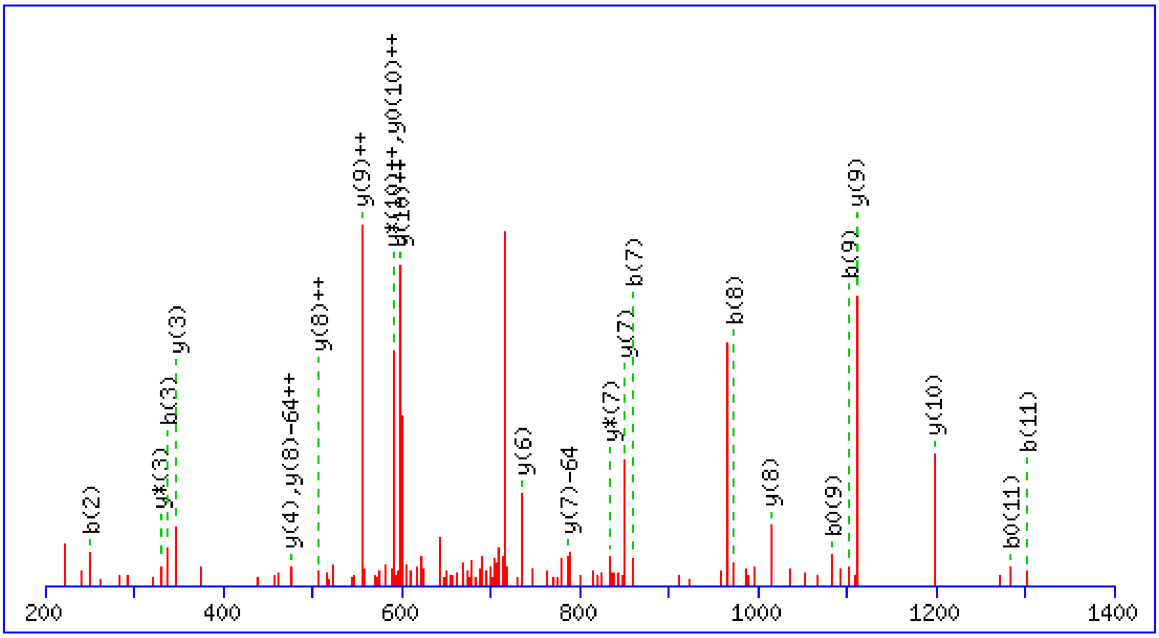

Spot no.661
Annexin A2
SYSPYDMLESIKK

#	b	b^{++}	b*	b^{*++}	b^{0}	b^{0++}	Seq.	y	y^{++}	y*	y^{*++}	y^{0}	y^{0++}	#
1	88.0393	44.5233			70.0287	35.518	S							13
2	251.1026	126.055			233.0921	117.0497	Y	1489.7243	745.3658	1472.6978	736.8525	1471.7137	736.3605	12
3	338.1347	169.571			320.1241	160.5657	S	1326.661	663.8341	1309.6344	655.3209	1308.6504	654.8288	11
4	435.1874	218.0974			417.1769	209.0921	P	1239.629	620.3181	1222.6024	611.8048	1221.6184	611.3128	10
5	598.2508	299.629			580.2402	290.6237	Y	1142.5762	571.7917	1125.5496	563.2785	1124.5656	562.7864	9
6	713.2777	357.1425			695.2671	348.1372	D	979.5129	490.2601	962.4863	481.7468	961.5023	481.2548	8
7	860.3131	430.6602			842.3025	421.6549	M	864.4859	432.7466	847.4594	424.2333	846.4754	423.7413	7
8	973.3972	487.2022			955.3866	478.1969	L	717.4505	359.2289	700.424	350.7156	699.44	350.2236	6
9	1102.4398	551.7235			1084.4292	542.7182	E	604.3665	302.6869	587.3399	294.1736	586.3559	293.6816	5
10	1189.4718	595.2395			1171.4612	586.2342	S	475.3239	238.1656	458.2973	229.6523	457.3133	229.1603	4
11	1302.5558	651.7816			1284.5453	642.7763	I	388.2918	194.6496	371.2653	186.1363			3
12	1430.6508	715.829	1413.6243	707.3158	1412.6402	706.8238	K	275.2078	138.1075	258.1812	129.5942			2
13							K	147.1128	74.06	130.0863	65.5468			1

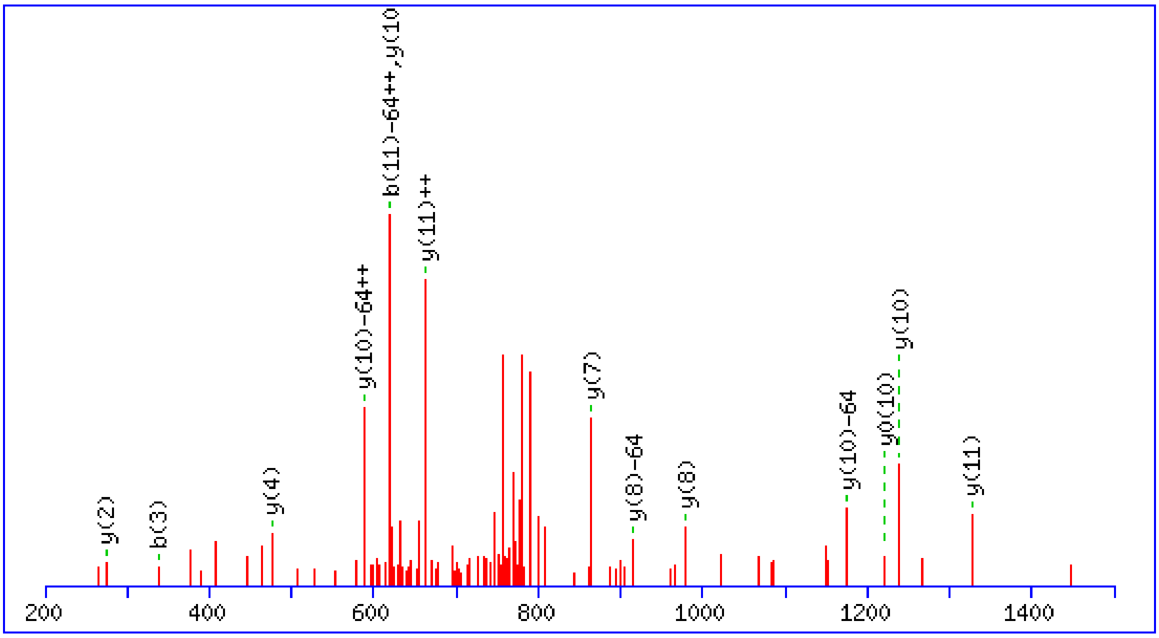

Spot no.661
Annexin A2
SEVDMLK

#	b	b^{++}	b^{0}	b^{0++}	Seq.	y	y^{++}	y*	y^{*++}	y^{0}	y^{0++}	#
1	88.0393	44.5233	70.0287	35.518	S							7
2	***217.0819***	109.0446	199.0713	100.0393	E	***750.3702***	375.6887	733.3437	367.1755	732.3597	366.6835	6
3	***316.1503***	158.5788	298.1397	149.5735	V	***621.3276***	311.1675	604.3011	302.6542	603.3171	302.1622	5
4	***431.1773***	216.0923	413.1667	207.087	D	***522.2592***	261.6332	505.2327	253.12	504.2486	252.628	4
5	***578.2127***	289.61	560.2021	280.6047	M	***407.2323***	204.1198	390.2057	195.6065			3
6	***691.2967***	346.152	673.2862	337.1467	L	***260.1969***	130.6021	243.1703	122.0888			2
7					K	147.1128	74.06	130.0863	65.5468			1

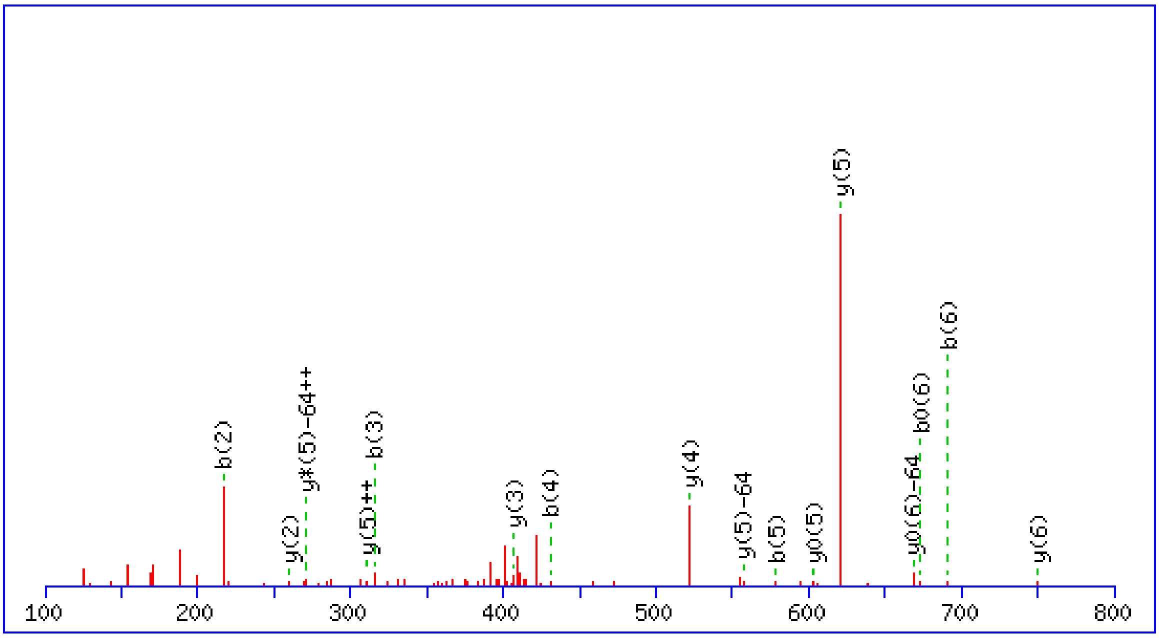

Spot no.661
Annexin A2
SLYYYIQQDTK

#	b	b^{++}	b*	b^{*++}	b^{0}	b^{0++}	Seq.	y	y^{++}	y*	y^{*++}	y^{0}	y^{0++}	#
1	88.0393	44.5233			70.0287	35.518	S							11
2	201.1234	101.0653			183.1128	92.06	L	1334.6627	667.835	1317.6361	659.3217	1316.6521	658.8297	10
3	**364.1867**	182.597			346.1761	173.5917	Y	***1221.5786***	611.293	1204.5521	602.7797	1203.5681	602.2877	9
4	527.25	264.1287			509.2395	255.1234	Y	***1058.5153***	529.7613	1041.4888	521.248	1040.5047	520.756	8
5	690.3134	345.6603			672.3028	336.655	Y	***895.452***	448.2296	878.4254	439.7164	877.4414	439.2243	7
6	**803.3974**	402.2023			785.3869	393.1971	I	***732.3886***	366.698	715.3621	358.1847	714.3781	357.6927	6
7	**931.456**	466.2316	914.4294	457.7184	913.4454	457.2264	Q	***619.3046***	310.1559	602.278	301.6427	601.294	301.1506	5
8	1059.5146	530.2609	1042.488	521.7477	1041.504	521.2556	Q	***491.246***	246.1266	474.2195	237.6134	473.2354	237.1214	4
9	**1174.5415**	587.7744	1157.515	579.2611	1156.531	578.7691	D	***363.1874***	182.0974	346.1609	173.5841	345.1769	173.0921	3
10	**1275.5892**	638.2982	1258.5626	629.785	1257.5786	629.293	T	***248.1605***	124.5839	231.1339	116.0706	230.1499	115.5786	2
11							K	147.1128	74.06	130.0863	65.5468			1

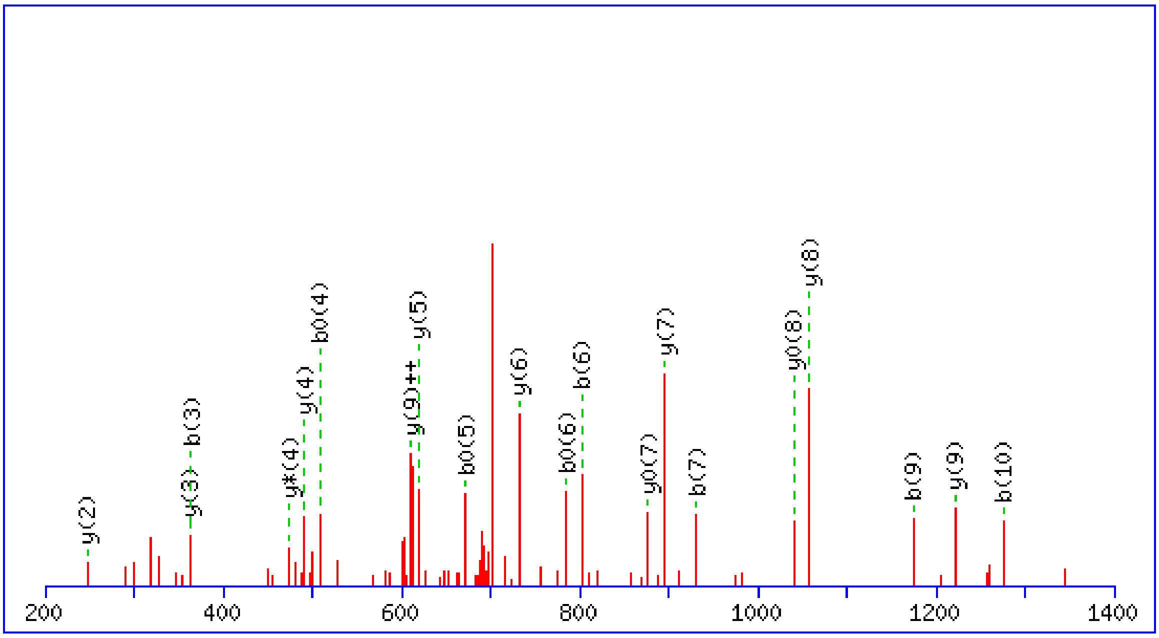

Spot no.296
Elongation factor 1-delta
QENGASVILR

#	b	b^{++}	b*	b*$^{++}$	b^{0}	b^{0++}	Seq.	y	y^{++}	y*	y*$^{++}$	y^{0}	y^{0++}	#
1	129.0659	65.0366	112.0393	56.5233			Q							10
2	258.1084	129.5579	241.0819	121.0446	240.0979	120.5526	E	958.5316	479.7694	941.5051	471.2562	940.5211	470.7642	9
3	372.1514	186.5793	355.1248	178.0661	354.1408	177.574	N	829.489	415.2482	812.4625	406.7349	811.4785	406.2429	8
4	429.1728	215.0901	412.1463	206.5768	411.1623	206.0848	G	715.4461	358.2267	698.4196	349.7134	697.4355	349.2214	7
5	**500.21**	250.6086	483.1834	242.0953	482.1994	241.6033	A	***658.4246***	329.716	641.3981	321.2027	640.4141	320.7107	6
6	**587.242**	294.1246	570.2154	285.6114	569.2314	285.1193	S	***587.3875***	294.1974	570.361	285.6841	569.377	285.1921	5
7	686.3104	343.6588	669.2838	335.1456	668.2998	334.6536	V	***500.3555***	250.6814	483.3289	242.1681			4
8	799.3945	400.2009	782.3679	391.6876	781.3839	391.1956	I	***401.2871***	201.1472	384.2605	192.6339			3
9	912.4785	456.7429	895.452	448.2296	894.468	447.7376	L	***288.203***	144.6051	271.1765	136.0919			2
10							R	***175.119***	88.0631	158.0924	79.5498			1

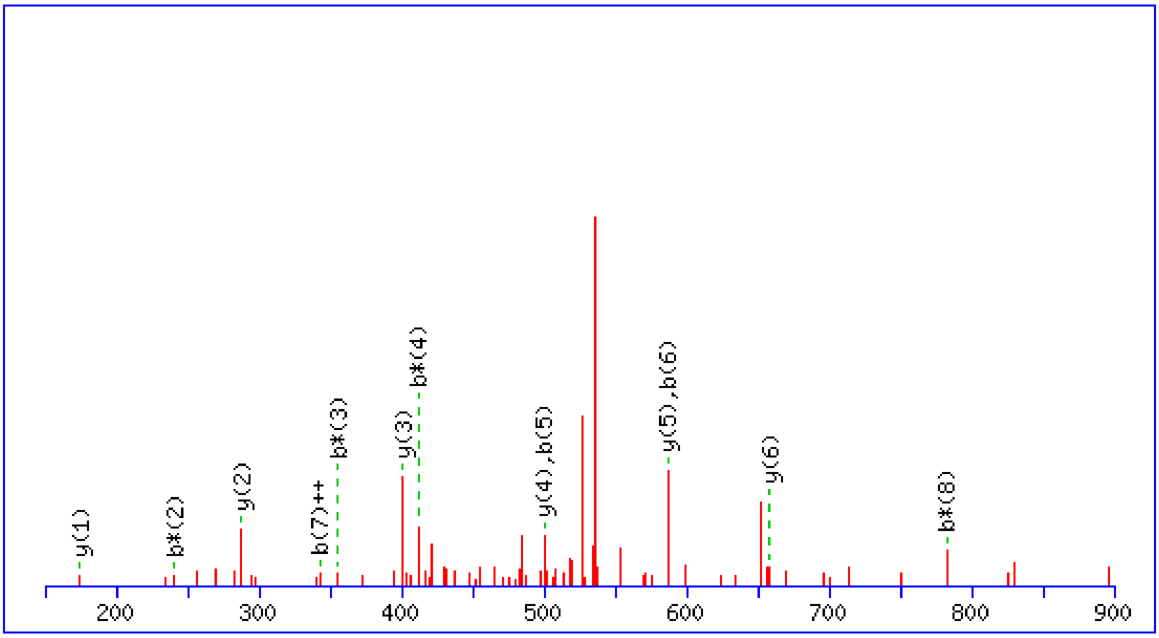

Spot no.296
Elongation factor 1-delta
IASLEVENQSLR

#	b	b++	b*	b*++	b0	b0++	Seq.	y	y++	y*	y*++	y0	y0++	#
1	114.0913	57.5493					I							12
2	185.1285	93.0679					A	1245.6434	623.3253	1228.6168	614.812	1227.6328	614.32	11
3	**272.1605**	136.5839			254.1499	127.5786	S	***1174.6062***	587.8068	1157.5797	579.2935	1156.5957	578.8015	10
4	385.2445	193.1259			367.234	184.1206	L	***1087.5742***	544.2907	1070.5477	535.7775	1069.5636	535.2855	9
5	514.2871	257.6472			496.2766	248.6419	E	***974.4901***	487.7487	957.4636	479.2354	956.4796	478.7434	8
6	**613.3556**	307.1814			595.345	298.1761	V	***845.4476***	423.2274	828.421	414.7141	827.437	414.2221	7
7	**742.3981**	371.7027			724.3876	362.6974	E	***746.3791***	373.6932	729.3526	365.1799	728.3686	364.6879	6
8	**856.4411**	428.7242	839.4145	420.2109	838.4305	419.7189	N	***617.3365***	309.1719	600.31	300.6586	599.326	300.1666	5
9	984.4997	492.7535	967.4731	484.2402	966.4891	483.7482	Q	***503.2936***	252.1504	486.2671	243.6372	485.2831	243.1452	4
10	1071.5317	536.2695	1054.5051	527.7562	1053.5211	527.2642	S	***375.235***	188.1212	358.2085	179.6079	357.2245	179.1159	3
11	**1184.6157**	592.8115	1167.5892	584.2982	1166.6052	583.8062	L	***288.203***	144.6051	271.1765	136.0919			2
12							R	175.119	88.0631	158.0924	79.5498			1

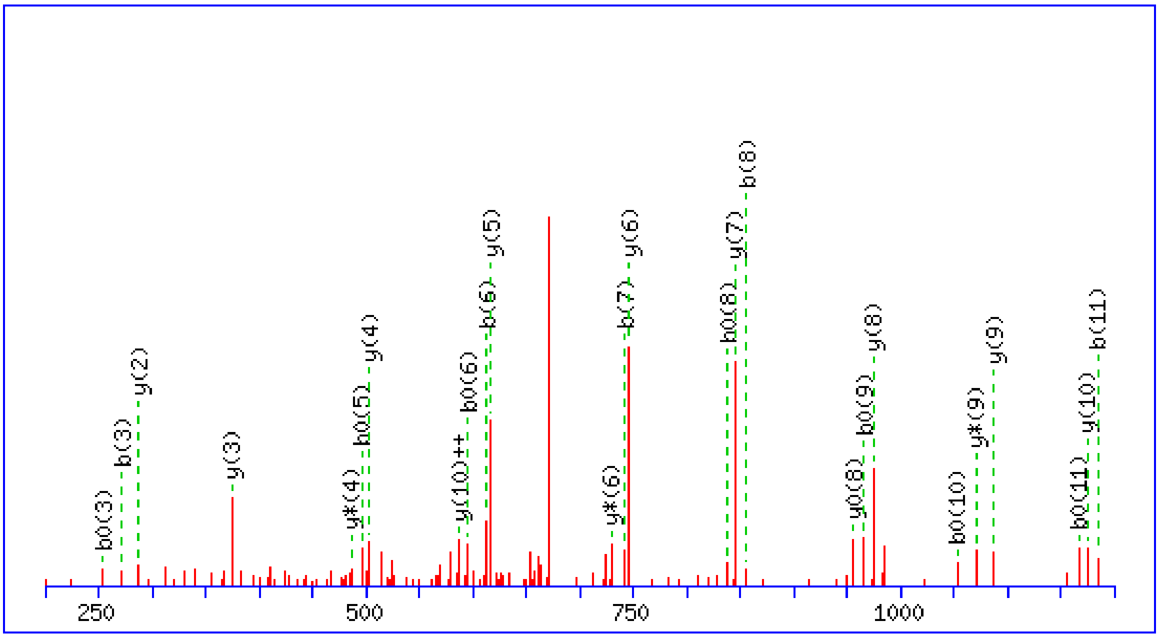

Spot no.296
Elongation factor 1-delta
AAAPQTQHVSPMR

#	b	b^{++}	b*	b*$^{++}$	b^{0}	b^{0++}	Seq.	y	y^{++}	y*	y*$^{++}$	y^{0}	y^{0++}	#
1	72.0444	36.5258					A							13
2	143.0815	72.0444					A	1338.6583	669.8328	1321.6317	661.3195	1320.6477	660.8275	12
3	214.1186	107.5629					A	1267.6212	634.3142	1250.5946	625.801	1249.6106	625.3089	11
4	311.1714	156.0893					P	***1196.5841***	598.7957	1179.5575	590.2824	1178.5735	589.7904	10
5	439.23	220.1186	422.2034	211.6053			Q	1099.5313	550.2693	1082.5048	541.756	1081.5207	541.264	9
6	540.2776	270.6425	523.2511	262.1292	522.2671	261.6372	T	***971.4727***	486.24	954.4462	477.7267	953.4622	477.2347	8
7	668.3362	334.6717	651.3097	326.1585	650.3257	325.6665	Q	***870.425***	435.7162	853.3985	427.2029	852.4145	426.7109	7
8	**805.3951**	403.2012	788.3686	394.6879	787.3846	394.1959	H	***742.3665***	371.6869	725.3399	363.1736	724.3559	362.6816	6
9	904.4635	452.7354	887.437	444.2221	886.453	443.7301	V	***605.3076***	303.1574	588.281	294.6441	587.297	294.1521	5
10	**991.4956**	496.2514	974.469	487.7381	973.485	487.2461	S	***506.2391***	253.6232	489.2126	245.1099	488.2286	244.6179	4
11	1088.5483	544.7778	1071.5218	536.2645	1070.5378	535.7725	P	***419.2071***	210.1072	402.1806	201.5939			3
12	1235.5837	618.2955	1218.5572	609.7822	1217.5732	609.2902	M	322.1544	161.5808	305.1278	153.0675			2
13							R	175.119	88.0631	158.0924	79.5498			1

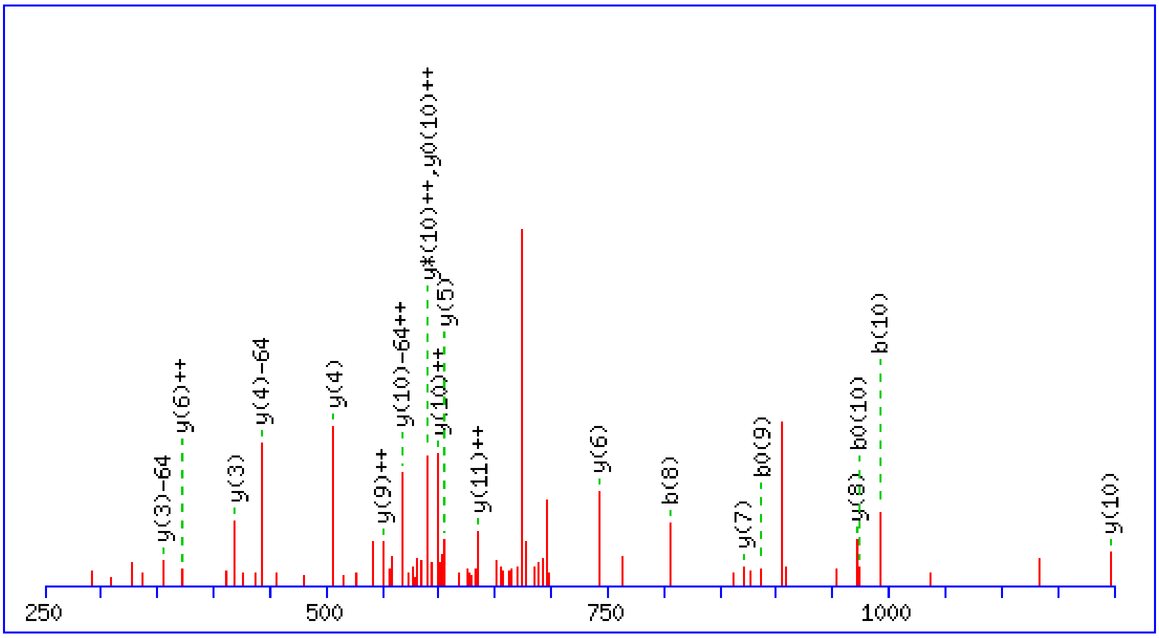

Spot no.296
Elongation factor 1-delta
SIQLDGLTWGGSK

#	b	b++	b*	b*++	b0	b0++	Seq.	y	y++	y*	y*++	y0	y0++	#
1	88.0393	44.5233			70.0287	35.518	S							13
2	201.1234	101.0653			183.1128	92.06	I	1274.6739	637.8406	1257.6474	629.3273	1256.6634	628.8353	12
3	**329.1819**	165.0946	312.1554	156.5813	311.1714	156.0893	Q	***1161.5899***	581.2986	1144.5633	572.7853	1143.5793	572.2933	11
4	**442.266**	221.6366	425.2395	213.1234	424.2554	212.6314	L	***1033.5313***	517.2693	1016.5047	508.756	1015.5207	508.264	10
5	557.293	279.1501	540.2664	270.6368	539.2824	270.1448	D	***920.4472***	460.7272	903.4207	452.214	902.4367	451.722	9
6	614.3144	307.6608	597.2879	299.1476	596.3039	298.6556	G	***805.4203***	403.2138	788.3937	394.7005	787.4097	394.2085	8
7	**727.3985**	364.2029	710.3719	355.6896	709.3879	355.1976	L	***748.3988***	374.703	731.3723	366.1898	730.3883	365.6978	7
8	**828.4462**	414.7267	811.4196	406.2134	810.4356	405.7214	T	***635.3148***	318.161	618.2882	309.6477	617.3042	309.1557	6
9	**1014.5255**	507.7664	997.4989	499.2531	996.5149	498.7611	W	***534.2671***	267.6372	517.2405	259.1239	516.2565	258.6319	5
10	**1071.5469**	536.2771	1054.5204	527.7638	1053.5364	527.2718	G	***348.1878***	174.5975	331.1612	166.0842	330.1772	165.5922	4
11	**1128.5684**	564.7878	1111.5419	556.2746	1110.5578	555.7826	G	***291.1663***	146.0868	274.1397	137.5735	273.1557	137.0815	3
12	**1215.6004**	608.3039	1198.5739	599.7906	1197.5899	599.2986	S	***234.1448***	117.5761	217.1183	109.0628	216.1343	108.5708	2
13							K	147.1128	74.06	130.0863	65.5468			1

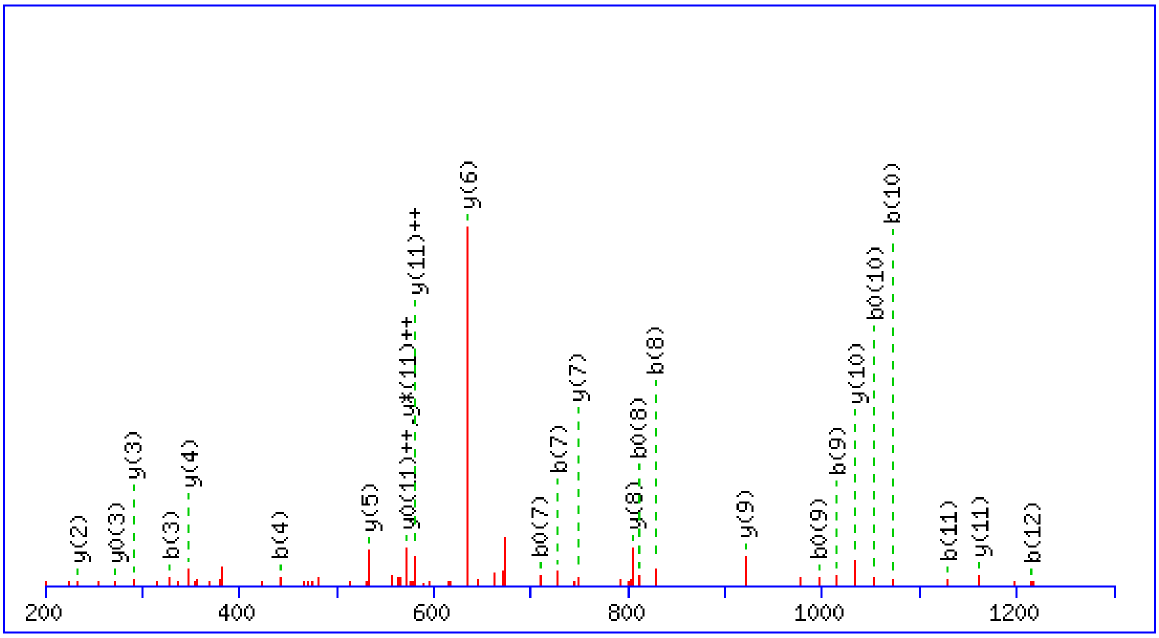

Spot no.655
Peptidyl prolyl cis-trans isomerase B isoform 2
VYFDLR

#	b	b++	b0	b0++	Seq.	y	y++	y*	y*++	y0	y0++	#
1	100.0757	50.5415			V							6
2	**263.139**	132.0731			Y	***713.3617***	357.1845	696.3352	348.6712	695.3511	348.1792	5
3	410.2074	205.6074			F	***550.2984***	275.6528	533.2718	267.1395	532.2878	266.6475	4
4	**525.2344**	263.1208	507.2238	254.1155	D	***403.23***	202.1186	386.2034	193.6053	385.2194	193.1133	3
5	638.3184	319.6629	620.3079	310.6576	L	***288.203***	144.6051	271.1765	136.0919			2
6					R	***175.119***	88.0631	158.0924	79.5498			1

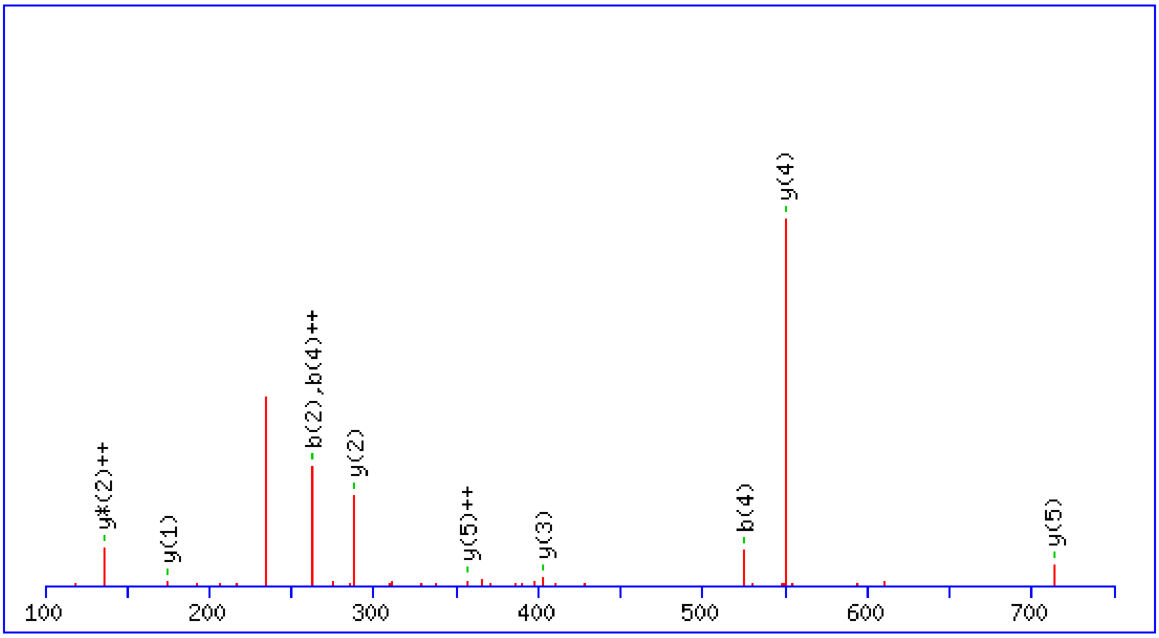

Spot no.655
Peptidyl prolyl cis-trans isomerase B isoform 2
IGDEDIGR

#	b	b++	b0	b0++	Seq.	y	y++	y*	y*++	y0	y0++	#
1	114.0913	57.5493			I							8
2	171.1128	86.06			G	761.3424	381.1748	744.3159	372.6616	743.3319	372.1696	7
3	286.1397	143.5735	268.1292	134.5682	D	704.321	352.6641	687.2944	344.1508	686.3104	343.6588	6
4	415.1823	208.0948	397.1718	199.0895	E	589.294	295.1506	572.2675	286.6374	571.2835	286.1454	5
5	530.2093	265.6083	512.1987	256.603	D	460.2514	230.6293	443.2249	222.1161	442.2409	221.6241	4
6	643.2933	322.1503	625.2828	313.145	I	345.2245	173.1159	328.1979	164.6026			3
7	700.3148	350.661	682.3042	341.6558	G	232.1404	116.5738	215.1139	108.0606			2
8					R	175.119	88.0631	158.0924	79.5498			1

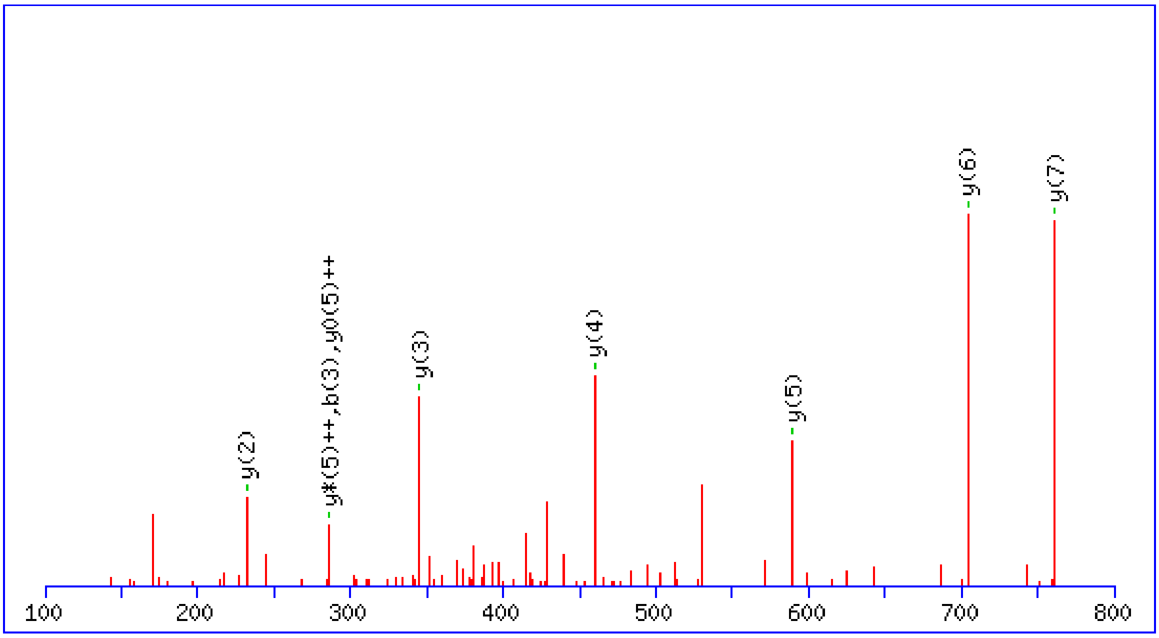

Spot no.655
Peptidyl prolyl cis-trans isomerase B isoform 2
VVIGLFGK

#	b	b++	Seq.	y	y++	y*	y*++	#
1	100.0757	50.5415	V					8
2	***199.1441***	100.0757	V	***733.4607***	367.234	716.4341	358.7207	7
3	***312.2282***	156.6177	I	***634.3923***	317.6998	617.3657	309.1865	6
4	***369.2496***	185.1285	G	***521.3082***	261.1577	504.2817	252.6445	5
5	***482.3337***	241.6705	L	***464.2867***	232.647	447.2602	224.1337	4
6	629.4021	315.2047	F	***351.2027***	176.105	334.1761	167.5917	3
7	***686.4236***	343.7154	G	***204.1343***	102.5708	187.1077	94.0575	2
8			K	147.1128	74.06	130.0863	65.5468	1

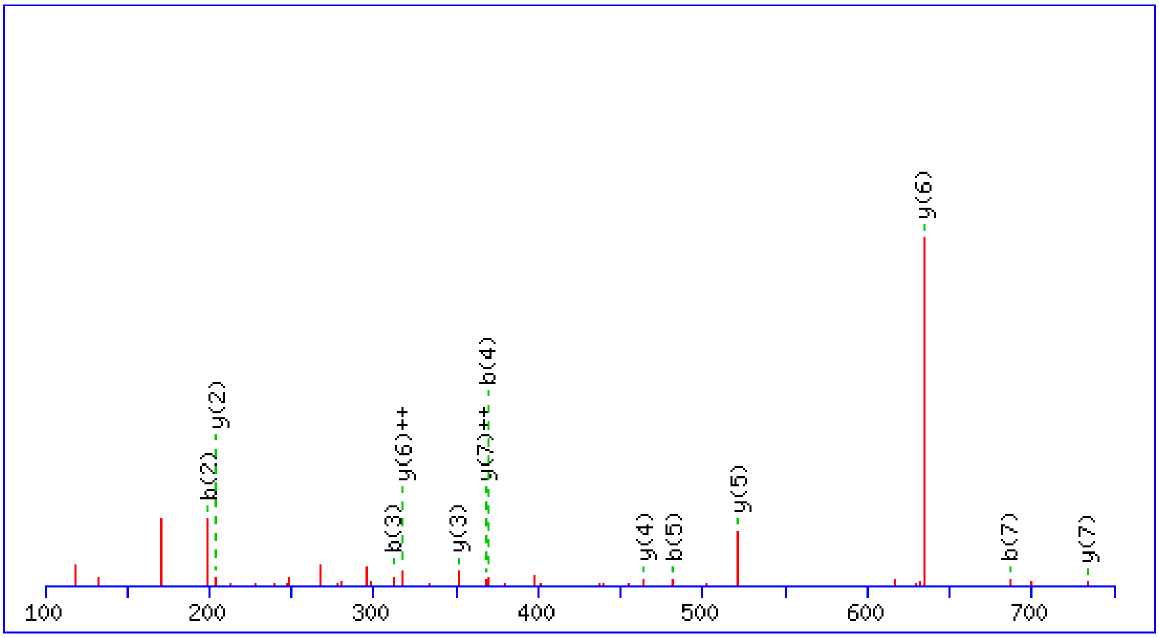

Spot no.655
Peptidyl prolyl cis-trans isomerase B isoform 2
TVDNFVALATGEK

#	b	b^{++}	b*	b^{*++}	b^0	b^{0++}	Seq.	y	y^{++}	y*	y^{*++}	y^0	y^{0++}	#
1	102.055	51.5311			84.0444	42.5258	T							13
2	**201.1234**	101.0653			183.1128	92.06	V	1263.6579	632.3326	1246.6314	623.8193	1245.6474	623.3273	12
3	316.1503	158.5788			298.1397	149.5735	D	***1164.5895***	582.7984	1147.563	574.2851	1146.579	573.7931	11
4	430.1932	215.6003	413.1667	207.087	412.1827	206.595	N	***1049.5626***	525.2849	1032.536	516.7717	1031.552	516.2796	10
5	**577.2617**	289.1345	560.2351	280.6212	559.2511	280.1292	F	***935.5197***	468.2635	918.4931	459.7502	917.5091	459.2582	9
6	676.3301	338.6687	659.3035	330.1554	658.3195	329.6634	V	***788.4512***	394.7293	771.4247	386.216	770.4407	385.724	8
7	747.3672	374.1872	730.3406	365.674	729.3566	365.1819	A	***689.3828***	345.1951	672.3563	336.6818	671.3723	336.1898	7
8	**860.4512**	430.7293	843.4247	422.216	842.4407	421.724	L	***618.3457***	309.6765	601.3192	301.1632	600.3352	300.6712	6
9	931.4884	466.2478	914.4618	457.7345	913.4778	457.2425	A	***505.2617***	253.1345	488.2351	244.6212	487.2511	244.1292	5
10	1032.536	516.7717	1015.5095	508.2584	1014.5255	507.7664	T	***434.2245***	217.6159	417.198	209.1026	416.214	208.6106	4
11	1089.5575	545.2824	1072.531	536.7691	1071.5469	536.2771	G	***333.1769***	167.0921	316.1503	158.5788	315.1663	158.0868	3
12	**1218.6001**	609.8037	1201.5735	601.2904	1200.5895	600.7984	E	276.1554	138.5813	259.1288	130.0681	258.1448	129.5761	2
13							K	147.1128	74.06	130.0863	65.5468			1

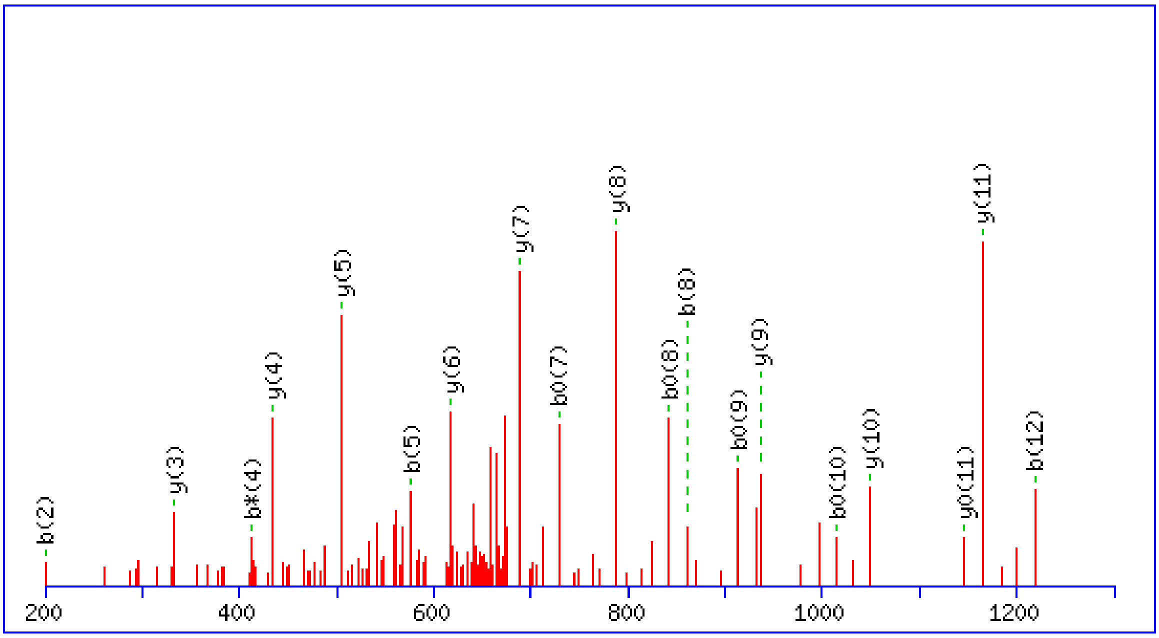

Spot no.655
Peptidyl prolyl cis-trans isomerase B isoform 2
GFGYKDSK

#	b	b++	b*	b*++	b0	b0++	Seq.	y	y++	y*	y*++	y0	y0++	#
1	58.0287	29.518					G							8
2	205.0972	103.0522					F	844.4199	422.7136	827.3934	414.2003	826.4094	413.7083	7
3	***262.1186***	131.5629					G	***697.3515***	349.1794	680.325	340.6661	679.341	340.1741	6
4	***425.1819***	213.0946					Y	***640.3301***	320.6687	623.3035	312.1554	622.3195	311.6634	5
5	***553.2769***	277.1421	536.2504	268.6288			K	477.2667	239.137	460.2402	230.6237	459.2562	230.1317	4
6	***668.3039***	334.6556	651.2773	326.1423	650.2933	325.6503	D	***349.1718***	175.0895	332.1452	166.5763	331.1612	166.0842	3
7	755.3359	378.1716	738.3093	369.6583	737.3253	369.1663	S	***234.1448***	117.5761	217.1183	109.0628	216.1343	108.5708	2
8							K	***147.1128***	74.06	130.0863	65.5468			1

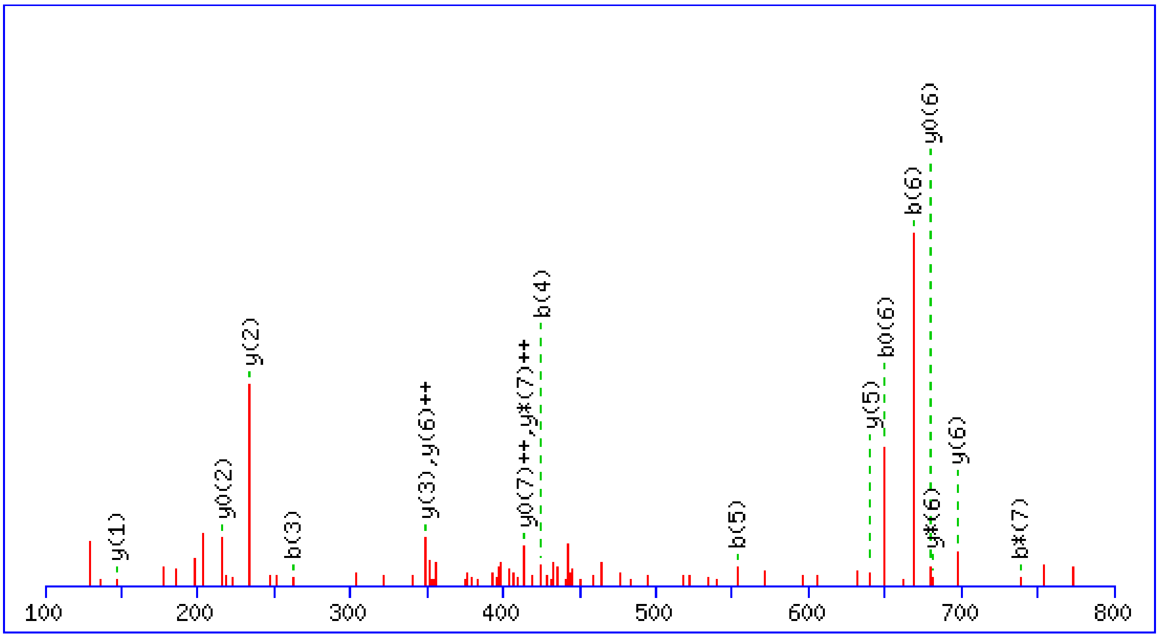

Spot no.655
Peptidyl prolyl cis-trans isomerase B isoform 2
VIKDFMIQGGDFTR

#	b	b++	b*	b*++	b0	b0++	Seq.	y	y++	y*	y*++	y0	y0++	#
1	100.0757	50.5415					V							14
2	213.1598	107.0835					I	1543.7573	772.3823	1526.7308	763.869	1525.7468	763.377	13
3	341.2547	171.131	324.2282	162.6177			K	***1430.6733***	715.8403	1413.6467	707.327	1412.6627	706.835	12
4	**456.2817**	228.6445	439.2551	220.1312	438.2711	219.6392	D	***1302.5783***	651.7928	1285.5518	643.2795	1284.5678	642.7875	11
5	**603.3501**	302.1787	586.3235	293.6654	585.3395	293.1734	F	***1187.5514***	594.2793	1170.5248	585.766	1169.5408	585.274	10
6	750.3855	375.6964	733.3589	367.1831	732.3749	366.6911	M	***1040.483***	520.7451	1023.4564	512.2318	1022.4724	511.7398	9
7	**863.4695**	432.2384	846.443	423.7251	845.459	423.2331	I	***893.4476***	447.2274	876.421	438.7141	875.437	438.2221	8
8	**991.5281**	496.2677	974.5016	487.7544	973.5176	487.2624	Q	***780.3635***	390.6854	763.3369	382.1721	762.3529	381.6801	7
9	1048.5496	524.7784	1031.523	516.2652	1030.539	515.7731	G	***652.3049***	326.6561	635.2784	318.1428	634.2944	317.6508	6
10	**1105.571**	553.2892	1088.5445	544.7759	1087.5605	544.2839	G	***595.2835***	298.1454	578.2569	289.6321	577.2729	289.1401	5
11	**1220.598**	610.8026	1203.5714	602.2894	1202.5874	601.7973	D	***538.262***	269.6346	521.2354	261.1214	520.2514	260.6293	4
12	1367.6664	684.3368	1350.6399	675.8236	1349.6558	675.3316	F	***423.235***	212.1212	406.2085	203.6079	405.2245	203.1159	3
13	1468.7141	734.8607	1451.6875	726.3474	1450.7035	725.8554	T	***276.1666***	138.587	259.1401	130.0737	258.1561	129.5817	2
14							R	175.119	88.0631	158.0924	79.5498			1

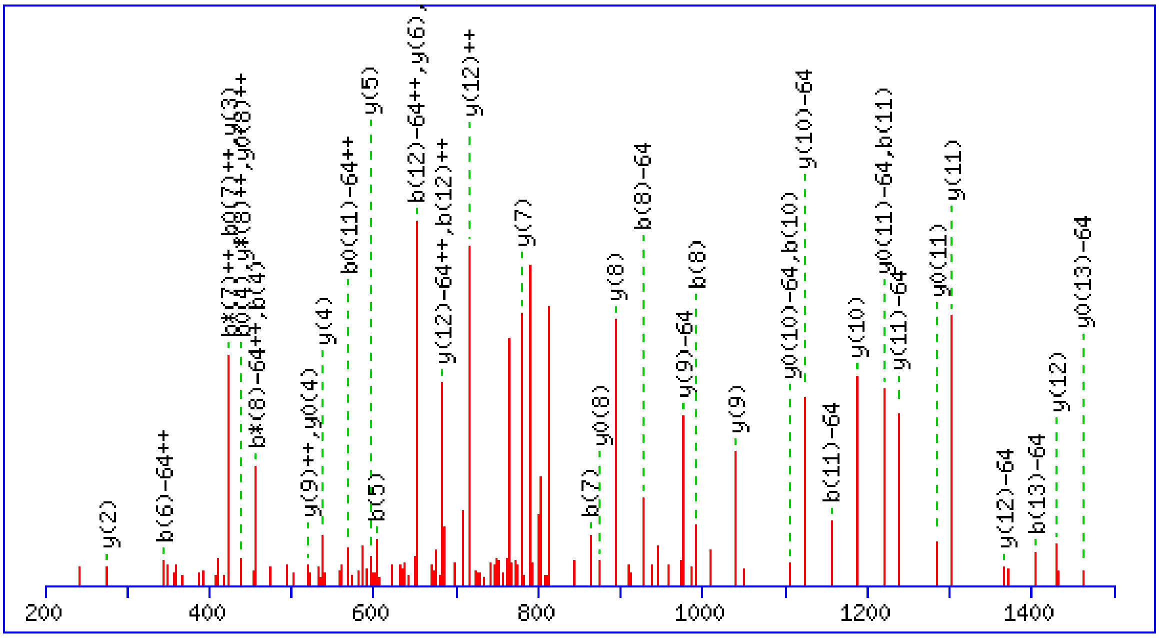

Spot no.655
Peptidyl prolyl cis-trans isomerase B isoform 2
DFMIQGGDFTR

#	b	b^{++}	b^{*}	b^{*++}	b^{0}	b^{0++}	Seq.	y	y^{++}	y^{*}	y^{*++}	y^{0}	y^{0++}	#
1	116.0342	58.5207			98.0237	49.5155	D							11
2	**263.1026**	132.055			245.0921	123.0497	F	1171.5565	586.2819	1154.5299	577.7686	1153.5459	577.2766	10
3	**394.1431**	197.5752			376.1326	188.5699	M	***1024.488***	512.7477	1007.4615	504.2344	1006.4775	503.7424	9
4	**507.2272**	254.1172			489.2166	245.1119	I	***893.4476***	447.2274	876.421	438.7141	875.437	438.2221	8
5	**635.2858**	318.1465	618.2592	309.6332	617.2752	309.1412	Q	***780.3635***	390.6854	763.3369	382.1721	762.3529	381.6801	7
6	692.3072	346.6573	675.2807	338.144	674.2967	337.652	G	***652.3049***	326.6561	635.2784	318.1428	634.2944	317.6508	6
7	749.3287	375.168	732.3021	366.6547	731.3181	366.1627	G	***595.2835***	298.1454	578.2569	289.6321	577.2729	289.1401	5
8	**864.3556**	432.6815	847.3291	424.1682	846.3451	423.6762	D	***538.262***	269.6346	521.2354	261.1214	520.2514	260.6293	4
9	**1011.424**	506.2157	994.3975	497.7024	993.4135	497.2104	F	***423.235***	212.1212	406.2085	203.6079	405.2245	203.1159	3
10	1112.4717	556.7395	1095.4452	548.2262	1094.4612	547.7342	T	***276.1666***	138.587	259.1401	130.0737	258.1561	129.5817	2
11							R	175.119	88.0631	158.0924	79.5498			1

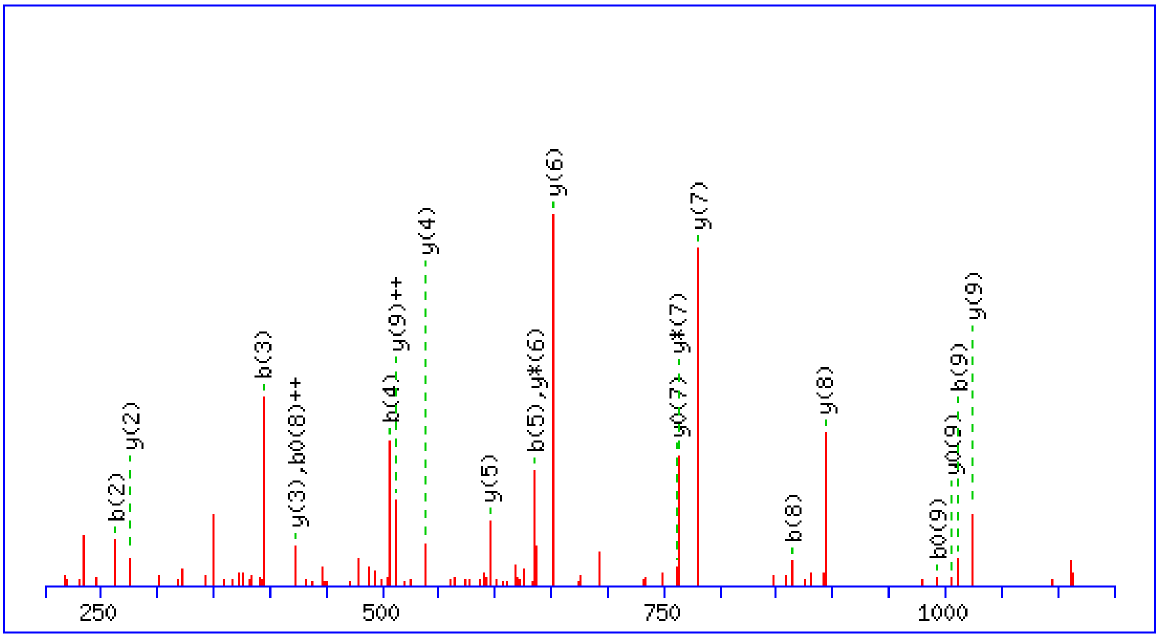

Spot no.655
Peptidyl prolyl cis-trans isomerase B isoform 2
FPDENFK

#	b	b++	b*	b*++	b0	b0++	Seq.	y	y++	y*	y*++	y0	y0++	#
1	148.0757	74.5415					F							7
2	245.1285	123.0679					P	***749.3464***	375.1769	732.3199	366.6636	731.3359	366.1716	6
3	360.1554	180.5813			342.1448	171.5761	D	***652.2937***	326.6505	635.2671	318.1372	634.2831	317.6452	5
4	489.198	**245.1026**			471.1874	236.0974	E	***537.2667***	269.137	520.2402	260.6237	519.2562	260.1317	4
5	603.2409	302.1241	586.2144	293.6108	585.2304	293.1188	N	***408.2241***	204.6157	391.1976	196.1024			3
6	750.3093	**375.6583**	733.2828	367.145	732.2988	366.653	F	***294.1812***	147.5942	277.1547	139.081			2
7							K	147.1128	74.06	130.0863	65.5468			1

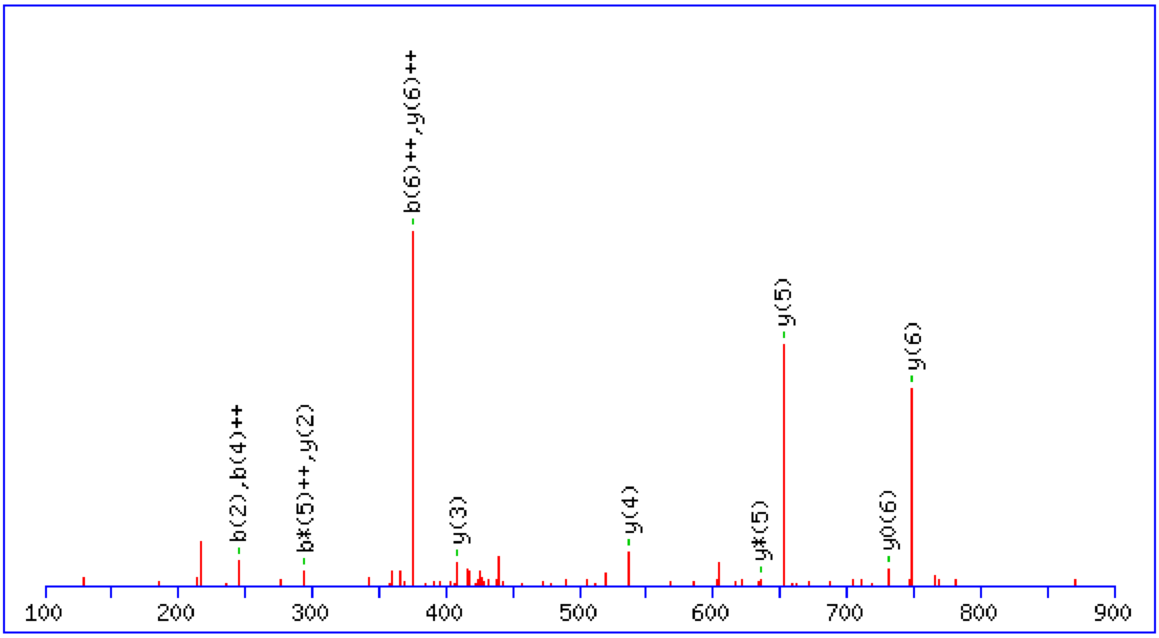

Spot no.655
Peptidyl prolyl cis-trans isomerase B isoform 2
LKHYGPGWVSMANAGK

#	b	b++	b*	b*++	b0	b0++	Seq.	y	y++	y*	y*++	y0	y0++	#
1	114.0913	57.5493					L							16
2	242.1863	121.5968	225.1598	113.0835			K	1618.7795	809.8934	1601.7529	801.3801	1600.7689	800.8881	15
3	379.2452	190.1262	362.2187	181.613			H	1490.6845	745.8459	1473.658	737.3326	1472.6739	736.8406	14
4	542.3085	271.6579	525.282	263.1446			Y	1353.6256	677.3164	1336.5991	668.8032	1335.615	668.3112	13
5	599.33	300.1686	582.3035	291.6554			G	1190.5623	595.7848	1173.5357	587.2715	1172.5517	586.7795	12
6	**696.3828**	348.695	679.3562	340.1817			P	1133.5408	567.274	1116.5143	558.7608	1115.5302	558.2688	11
7	753.4042	377.2058	736.3777	368.6925			G	1036.488	518.7477	1019.4615	510.2344	1018.4775	509.7424	10
8	939.4835	**470.2454**	922.457	461.7321			W	979.4666	490.2369	962.44	481.7237	961.456	481.2316	9
9	**1038.552**	**519.7796**	1021.5254	511.2663			V	***793.3873***	397.1973	776.3607	388.684	775.3767	388.192	8
10	1125.584	563.2956	1108.5574	554.7824	1107.5734	554.2904	S	***694.3189***	347.6631	677.2923	339.1498	676.3083	338.6578	7
11	1272.6194	636.8133	1255.5928	628.3001	1254.6088	627.8081	M	607.2868	304.147	590.2603	295.6338			6
12	1343.6565	672.3319	1326.63	663.8186	1325.6459	663.3266	A	460.2514	230.6293	443.2249	222.1161			5
13	1457.6994	729.3534	1440.6729	720.8401	1439.6889	720.3481	N	***389.2143***	195.1108	372.1878	186.5975			4
14	1528.7365	764.8719	1511.71	756.3586	1510.726	755.8666	A	***275.1714***	138.0893	258.1448	129.5761			3
15	1585.758	**793.3826**	1568.7315	784.8694	1567.7474	784.3774	G	204.1343	102.5708	187.1077	94.0575			2
16							K	147.1128	74.06	130.0863	65.5468			1

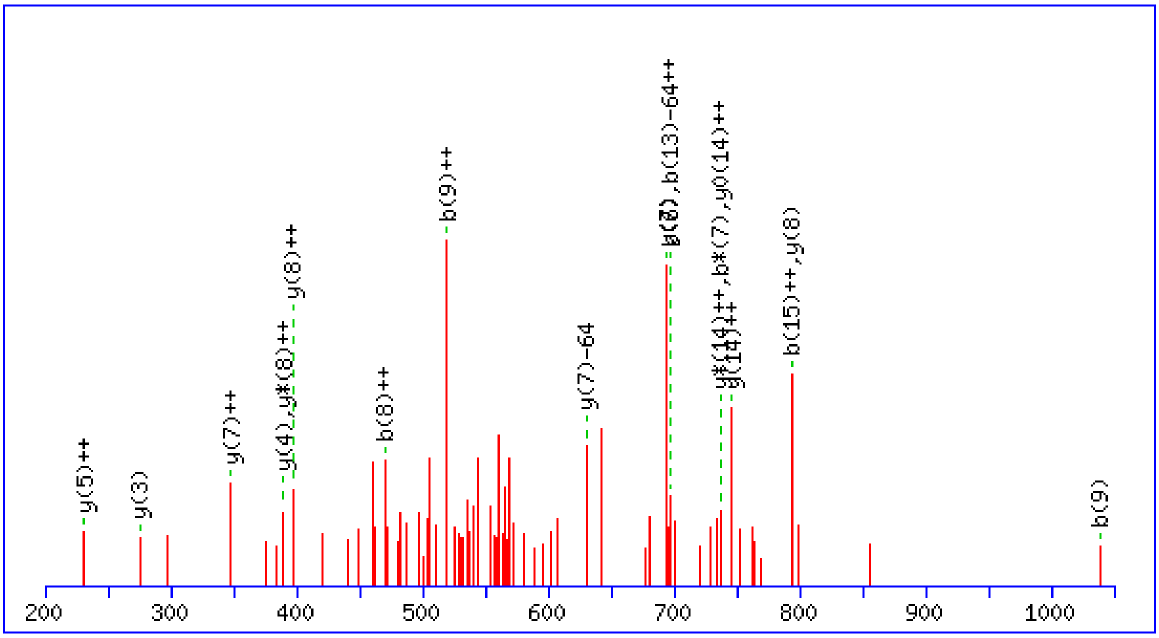

Spot no.655
Peptidyl prolyl cis-trans isomerase B isoform 2
HYGPGWVSMANAGK

#	b	b^{++}	b*	b^{*++}	b^{0}	b^{0++}	Seq.	y	y^{++}	y*	y^{*++}	y^{0}	y^{0++}	#
1	138.0662	69.5367					H							14
2	***301.1295***	151.0684					Y	***1353.6256***	677.3164	1336.5991	668.8032	1335.615	668.3112	13
3	***358.151***	179.5791					G	***1190.5623***	595.7848	1173.5357	587.2715	1172.5517	586.7795	12
4	455.2037	228.1055					P	***1133.5408***	567.274	1116.5143	558.7608	1115.5302	558.2688	11
5	***512.2252***	256.6162					G	***1036.488***	518.7477	1019.4615	510.2344	1018.4775	509.7424	10
6	698.3045	349.6559					W	***979.4666***	490.2369	962.44	481.7237	961.456	481.2316	9
7	***797.3729***	399.1901					V	***793.3873***	397.1973	776.3607	388.684	775.3767	388.192	8
8	***884.405***	442.7061			866.3944	433.7008	S	***694.3189***	347.6631	677.2923	339.1498	676.3083	338.6578	7
9	1031.4404	516.2238			1013.4298	507.2185	M	607.2868	304.147	590.2603	295.6338			6
10	1102.4775	551.7424			1084.4669	542.7371	A	***460.2514***	230.6293	443.2249	222.1161			5
11	***1216.5204***	608.7638	1199.4939	600.2506	1198.5098	599.7586	N	***389.2143***	195.1108	372.1878	186.5975			4
12	***1287.5575***	644.2824	1270.531	635.7691	1269.547	635.2771	A	275.1714	138.0893	258.1448	129.5761			3
13	***1344.579***	672.7931	1327.5524	664.2799	1326.5684	663.7878	G	204.1343	102.5708	187.1077	94.0575			2
14							K	147.1128	74.06	130.0863	65.5468			1

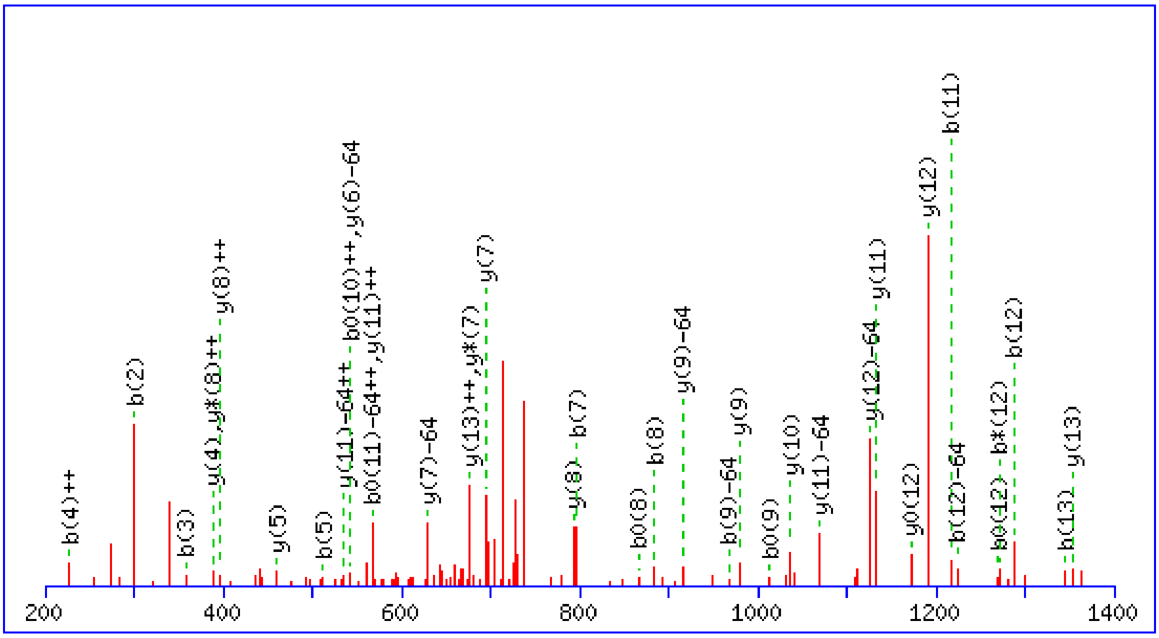

Spot no.655
Peptidyl prolyl cis-trans isomerase B isoform 2
DTNGSQFFITTVK

#	b	b^{++}	b*	b^{*++}	b^{0}	b^{0++}	Seq.	y	y^{++}	y*	y^{*++}	y^{0}	y^{0++}	#
1	116.0342	58.5207			98.0237	49.5155	D							13
2	217.0819	109.0446			199.0713	100.0393	T	1342.7001	671.8537	1325.6736	663.3404	1324.6896	662.8484	12
3	331.1248	166.0661	314.0983	157.5528	313.1143	157.0608	N	1241.6525	621.3299	1224.6259	612.8166	1223.6419	612.3246	11
4	388.1463	194.5768	371.1197	186.0635	370.1357	185.5715	G	***1127.6095***	564.3084	1110.583	555.7951	1109.599	555.3031	10
5	475.1783	238.0928	458.1518	229.5795	457.1678	229.0875	S	***1070.5881***	535.7977	1053.5615	527.2844	1052.5775	526.7924	9
6	603.2369	302.1221	586.2103	293.6088	585.2263	293.1168	Q	***983.556***	492.2817	966.5295	483.7684	965.5455	483.2764	8
7	750.3053	375.6563	733.2788	367.143	732.2947	366.651	F	***855.4975***	428.2524	838.4709	419.7391	837.4869	419.2471	7
8	**897.3737**	449.1905	880.3472	440.6772	879.3632	440.1852	F	***708.4291***	354.7182	691.4025	346.2049	690.4185	345.7129	6
9	**1010.4578**	505.7325	993.4312	497.2193	992.4472	496.7272	I	***561.3606***	281.184	544.3341	272.6707	543.3501	272.1787	5
10	**1111.5055**	556.2564	1094.4789	547.7431	1093.4949	547.2511	T	***448.2766***	224.6419	431.25	216.1287	430.266	215.6366	4
11	1212.5531	606.7802	1195.5266	598.2669	1194.5426	597.7749	T	***347.2289***	174.1181	330.2023	165.6048	329.2183	165.1128	3
12	**1311.6216**	656.3144	1294.595	647.8011	1293.611	647.3091	V	246.1812	123.5942	229.1547	115.081			2
13							K	147.1128	74.06	130.0863	65.5468			1

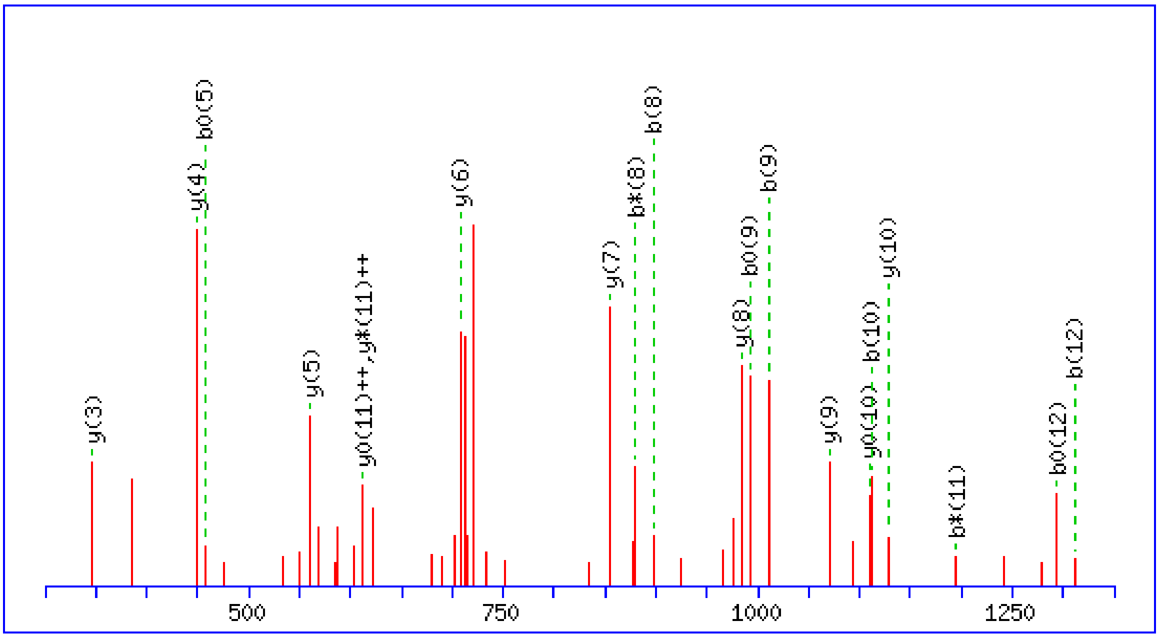

Spot no.655
Peptidyl prolyl cis-trans isomerase B isoform 2
VLEGMEVVR

#	b	b^{++}	b^{0}	b^{0++}	Seq.	y	y^{++}	y*	y^{*++}	y^{0}	y^{0++}	#
1	100.0757	50.5415			V							9
2	**213.1598**	107.0835			L	932.487	466.7471	915.4604	458.2339	914.4764	457.7418	8
3	**342.2023**	171.6048	324.1918	162.5995	E	***819.4029***	410.2051	802.3764	401.6918	801.3924	401.1998	7
4	399.2238	200.1155	381.2132	191.1103	G	***690.3603***	345.6838	673.3338	337.1705	672.3498	336.6785	6
5	530.2643	265.6358	512.2537	256.6305	M	***633.3389***	317.1731	616.3123	308.6598	615.3283	308.1678	5
6	659.3069	330.1571	641.2963	321.1518	E	***502.2984***	251.6528	485.2718	243.1395	484.2878	242.6475	4
7	758.3753	379.6913	740.3647	370.686	V	***373.2558***	187.1315	356.2292	178.6183			3
8	**857.4437**	429.2255	839.4332	420.2202	V	***274.1874***	137.5973	257.1608	129.084			2
9					R	175.119	88.0631	158.0924	79.5498			

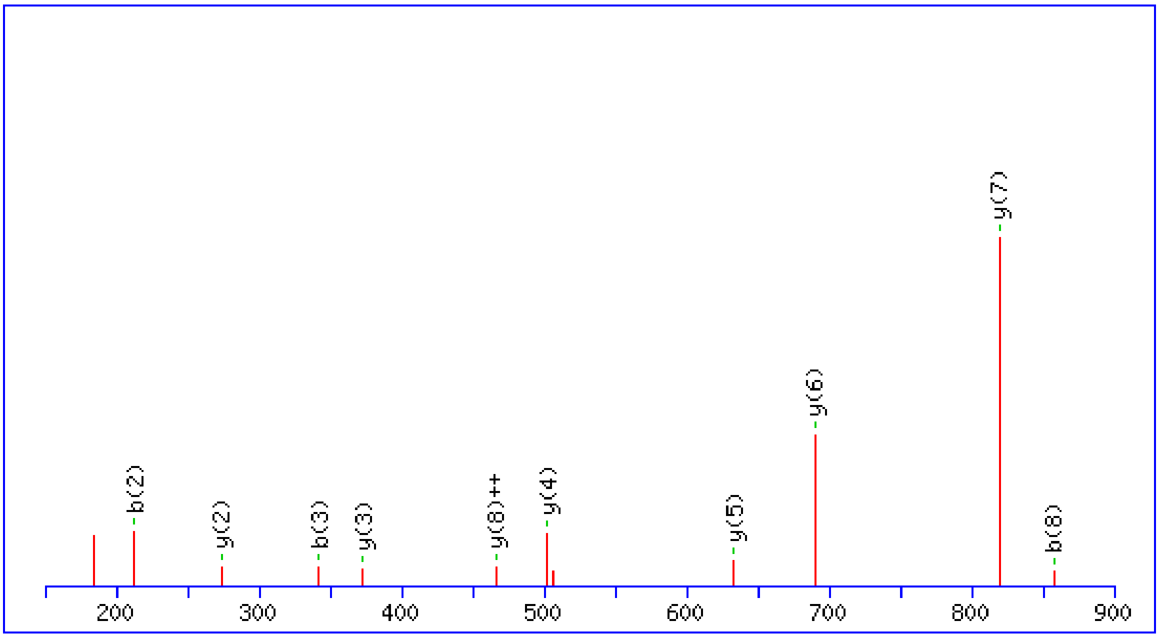

Spot no.655
Peptidyl prolyl cis-trans isomerase B isoform 2
DKPLKDVTIADCGK

#	b	b^{++}	b*	b^{*++}	b^{0}	b^{0++}	Seq.	y	y^{++}	y*	y^{*++}	y^{0}	y^{0++}	#
1	116.0342	58.5207			98.0237	49.5155	D							14
2	244.1292	122.5682	227.1026	114.055	226.1186	113.5629	K	1444.7828	722.8951	1427.7563	714.3818	1426.7723	713.8898	13
3	341.1819	171.0946	324.1554	162.5813	323.1714	162.0893	P	***1316.6879***	658.8476	1299.6613	650.3343	1298.6773	649.8423	12
4	454.266	227.6366	437.2395	219.1234	436.2554	218.6314	L	***1219.6351***	610.3212	1202.6086	601.8079	1201.6245	601.3159	11
5	**582.361**	291.6841	565.3344	283.1709	564.3504	282.6788	K	***1106.551***	553.7792	1089.5245	545.2659	1088.5405	544.7739	10
6	697.3879	349.1976	680.3614	340.6843	679.3774	340.1923	D	***978.4561***	489.7317	961.4295	481.2184	960.4455	480.7264	9
7	796.4563	398.7318	779.4298	390.2185	778.4458	389.7265	V	***863.4291***	432.2182	846.4026	423.7049	845.4186	423.2129	8
8	897.504	449.2556	880.4775	440.7424	879.4934	440.2504	T	764.3607	382.684	747.3342	374.1707	746.3502	373.6787	7
9	**1010.5881**	505.7977	993.5615	497.2844	992.5775	496.7924	I	663.313	332.1602	646.2865	323.6469	645.3025	323.1549	6
10	**1081.6252**	541.3162	1064.5986	532.803	1063.6146	532.3109	A	***550.229***	275.6181	533.2024	267.1049	532.2184	266.6128	5
11	**1196.6521**	598.8297	1179.6256	590.3164	1178.6416	589.8244	D	***479.1919***	240.0996	462.1653	231.5863	461.1813	231.0943	4
12	1356.6828	678.845	1339.6562	670.3318	1338.6722	669.8397	C	***364.1649***	182.5861	347.1384	174.0728			3
13	1413.7042	707.3558	1396.6777	698.8425	1395.6937	698.3505	G	204.1343	102.5708	187.1077	94.0575			2
14							K	147.1128	74.06	130.0863	65.5468			1

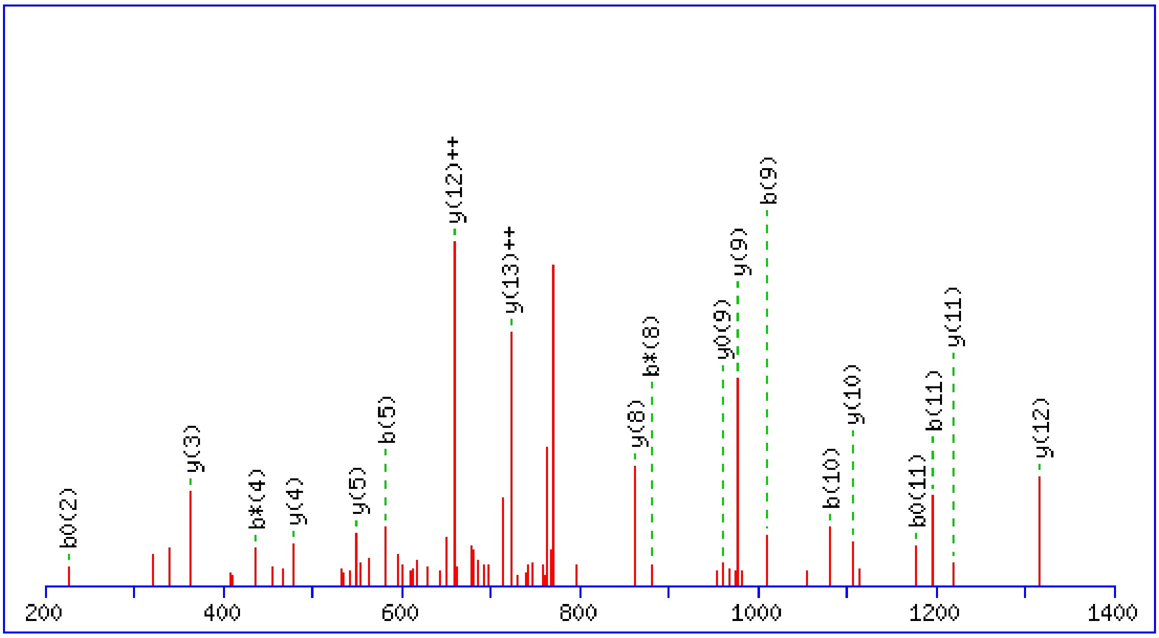

Spot no.655
Peptidyl prolyl cis-trans isomerase B isoform 2
DVTIADCGK

#	b	b^{++}	b^{0}	b^{0++}	Seq.	y	y^{++}	y*	y^{*++}	y^{0}	y^{0++}	#
1	116.0342	58.5207	98.0237	49.5155	D							9
2	***215.1026***	108.055	197.0921	99.0497	V	863.4291	432.2182	846.4026	423.7049	845.4186	423.2129	8
3	***316.1503***	158.5788	298.1397	149.5735	T	***764.3607***	382.684	747.3342	374.1707	746.3502	373.6787	7
4	***429.2344***	215.1208	411.2238	206.1155	I	***663.313***	332.1602	646.2865	323.6469	645.3025	323.1549	6
5	500.2715	250.6394	482.2609	241.6341	A	***550.229***	275.6181	533.2024	267.1049	532.2184	266.6128	5
6	***615.2984***	308.1529	597.2879	299.1476	D	***479.1919***	240.0996	462.1653	231.5863	461.1813	231.0943	4
7	775.3291	388.1682	757.3185	379.1629	C	364.1649	182.5861	347.1384	174.0728			3
8	***832.3505***	416.6789	814.34	407.6736	G	204.1343	102.5708	187.1077	94.0575			2
9					K	147.1128	74.06	130.0863	65.5468			1

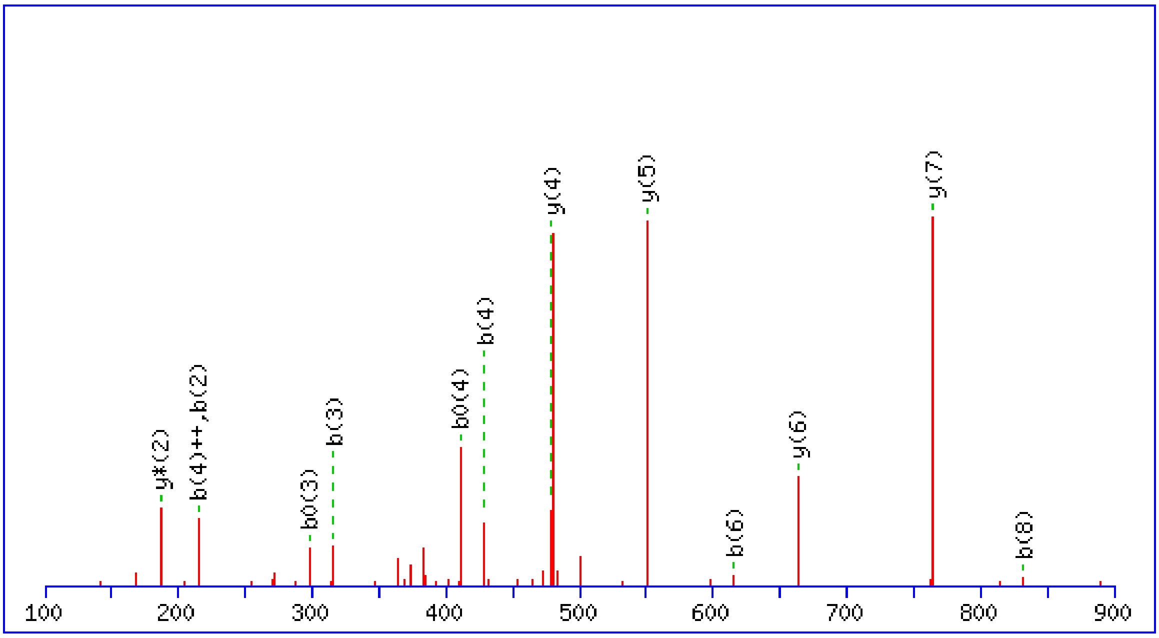

Spot no.655
Peptidyl prolyl cis-trans isomerase B isoform 2
IEVEKPFAIAK

#	b	b^{++}	b*	b^{*++}	b^{0}	b^{0++}	Seq.	y	y^{++}	y*	y^{*++}	y^{0}	y^{0++}	#
1	114.0913	57.5493					I							11
2	***243.1339***	122.0706			225.1234	113.0653	E	1131.6408	566.3241	1114.6143	557.8108	1113.6303	557.3188	10
3	***342.2023***	171.6048			324.1918	162.5995	V	**1002.5982**	501.8028	985.5717	493.2895	984.5877	492.7975	9
4	***471.2449***	236.1261			453.2344	227.1208	E	**903.5298**	452.2686	886.5033	443.7553	885.5193	443.2633	8
5	599.3399	300.1736	582.3134	291.6603	581.3293	291.1683	K	**774.4872**	387.7473	757.4607	379.234			7
6	696.3927	348.7	679.3661	340.1867	678.3821	339.6947	P	**646.3923**	323.6998	629.3657	315.1865			6
7	843.4611	422.2342	826.4345	413.7209	825.4505	413.2289	F	549.3395	275.1734	532.313	266.6601			5
8	***914.4982***	457.7527	897.4716	449.2395	896.4876	448.7475	A	402.2711	201.6392	385.2445	193.1259			4
9	***1027.5823***	514.2948	1010.5557	505.7815	1009.5717	505.2895	I	331.234	166.1206	314.2074	157.6074			3
10	***1098.6194***	549.8133	1081.5928	541.3001	1080.6088	540.808	A	218.1499	109.5786	201.1234	101.0653			2
11							K	147.1128	74.06	130.0863	65.5468			1

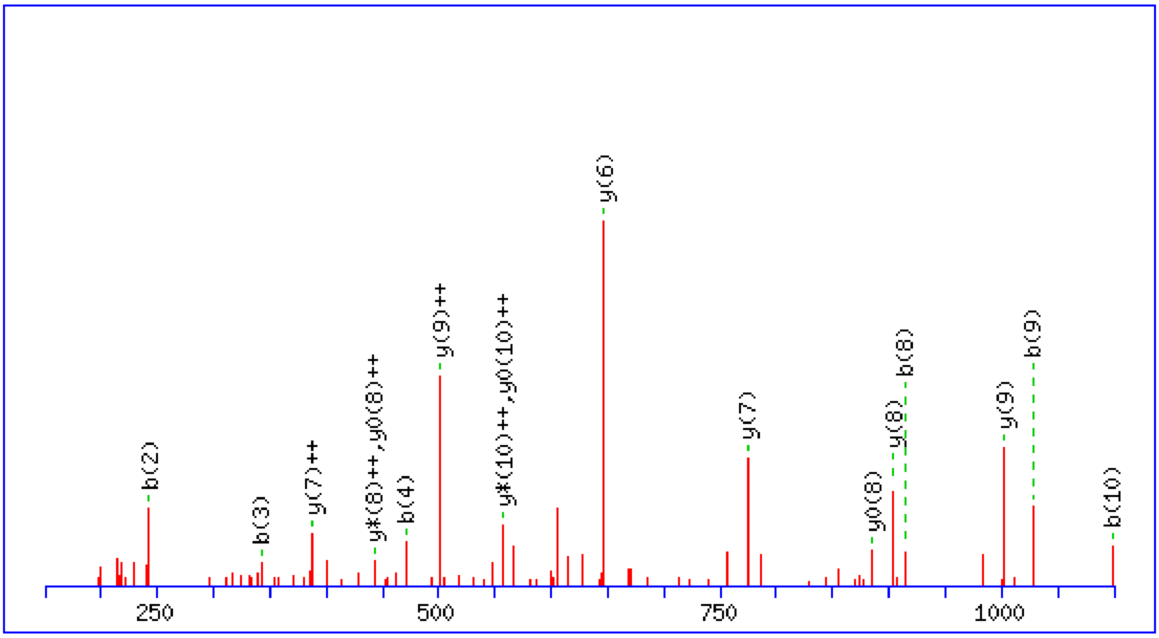

Spot no.655
Peptidyl prolyl cis-trans isomerase B isoform 2
IEVEKPFAIAKE

#	b	b++	b*	b*++	b0	b0++	Seq.	y	y++	y*	y*++	y0	y0++	#
1	114.0913	57.5493					I							12
2	**243.1339**	122.0706			225.1234	113.0653	E	1260.6834	630.8454	1243.6569	622.3321	1242.6729	621.8401	11
3	**342.2023**	171.6048			324.1918	162.5995	V	***1131.6408***	566.3241	1114.6143	557.8108	1113.6303	557.3188	10
4	**471.2449**	236.1261			453.2344	227.1208	E	***1032.5724***	516.7898	1015.5459	508.2766	1014.5619	507.7846	9
5	**599.3399**	300.1736	582.3134	291.6603	581.3293	291.1683	K	***903.5298***	452.2686	886.5033	443.7553	885.5193	443.2633	8
6	696.3927	348.7	679.3661	340.1867	678.3821	339.6947	P	***775.4349***	388.2211	758.4083	379.7078	757.4243	379.2158	7
7	843.4611	422.2342	826.4345	413.7209	825.4505	413.2289	F	***678.3821***	339.6947	661.3556	331.1814	660.3715	330.6894	6
8	**914.4982**	457.7527	897.4716	449.2395	896.4876	448.7475	A	531.3137	266.1605	514.2871	257.6472	513.3031	257.1552	5
9	**1027.5823**	514.2948	1010.5557	505.7815	1009.5717	505.2895	I	***460.2766***	230.6419	443.25	222.1287	442.266	221.6366	4
10	**1098.6194**	549.8133	1081.5928	541.3001	1080.6088	540.808	A	***347.1925***	174.0999	330.166	165.5866	329.1819	165.0946	3
11	1226.7143	613.8608	1209.6878	605.3475	1208.7038	604.8555	K	***276.1554***	138.5813	259.1288	130.0681	258.1448	129.5761	2
12							E	148.0604	74.5339			130.0499	65.5286	1

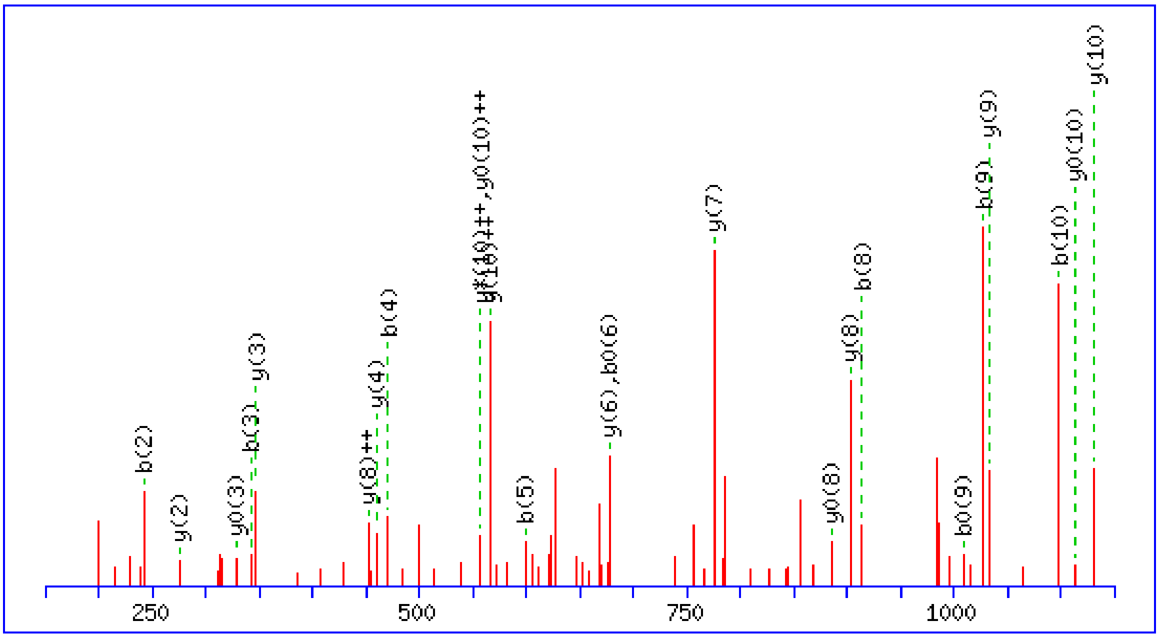

Spot no.799
ATP synthase subunit beta, mitochondrial precursor
LVLEVAQHLGESTVR

#	b	b++	b*	b*++	b0	b0++	Seq.	y	y++	y*	y*++	y0	y0++	#
1	114.0913	57.5493					L							15
2	213.1598	107.0835					V	1537.8333	769.4203	1520.8067	760.907	1519.8227	760.415	14
3	326.2438	163.6255					L	1438.7649	719.8861	1421.7383	711.3728	1420.7543	710.8808	13
4	455.2864	228.1468			437.2758	219.1416	E	1325.6808	663.344	1308.6543	654.8308	1307.6702	654.3388	12
5	554.3548	277.6811			536.3443	268.6758	V	***1196.6382***	598.8227	1179.6117	590.3095	1178.6276	589.8175	11
6	625.3919	313.1996			607.3814	304.1943	A	***1097.5698***	549.2885	1080.5432	540.7753	1079.5592	540.2833	10
7	753.4505	377.2289	736.424	368.7156	735.44	368.2236	Q	***1026.5327***	513.77	1009.5061	505.2567	1008.5221	504.7647	9
8	**890.5094**	445.7584	873.4829	437.2451	872.4989	436.7531	H	***898.4741***	449.7407	881.4476	441.2274	880.4635	440.7354	8
9	**1003.5935**	502.3004	986.5669	493.7871	985.5829	493.2951	L	***761.4152***	381.2112	744.3886	372.698	743.4046	372.206	7
10	1060.615	530.8111	1043.5884	522.2978	1042.6044	521.8058	G	***648.3311***	324.6692	631.3046	316.1559	630.3206	315.6639	6
11	**1189.6576**	595.3324	1172.631	586.8191	1171.647	586.3271	E	591.3097	296.1585	574.2831	287.6452	573.2991	287.1532	5
12	1276.6896	638.8484	1259.663	630.3352	1258.679	629.8431	S	***462.2671***	231.6372	445.2405	223.1239	444.2565	222.6319	4
13	1377.7373	689.3723	1360.7107	680.859	1359.7267	680.367	T	375.235	188.1212	358.2085	179.6079	357.2245	179.1159	3
14	1476.8057	738.9065	1459.7791	730.3932	1458.7951	729.9012	V	274.1874	137.5973	257.1608	129.084			2
15							R	175.119	88.0631	158.0924	79.5498			1

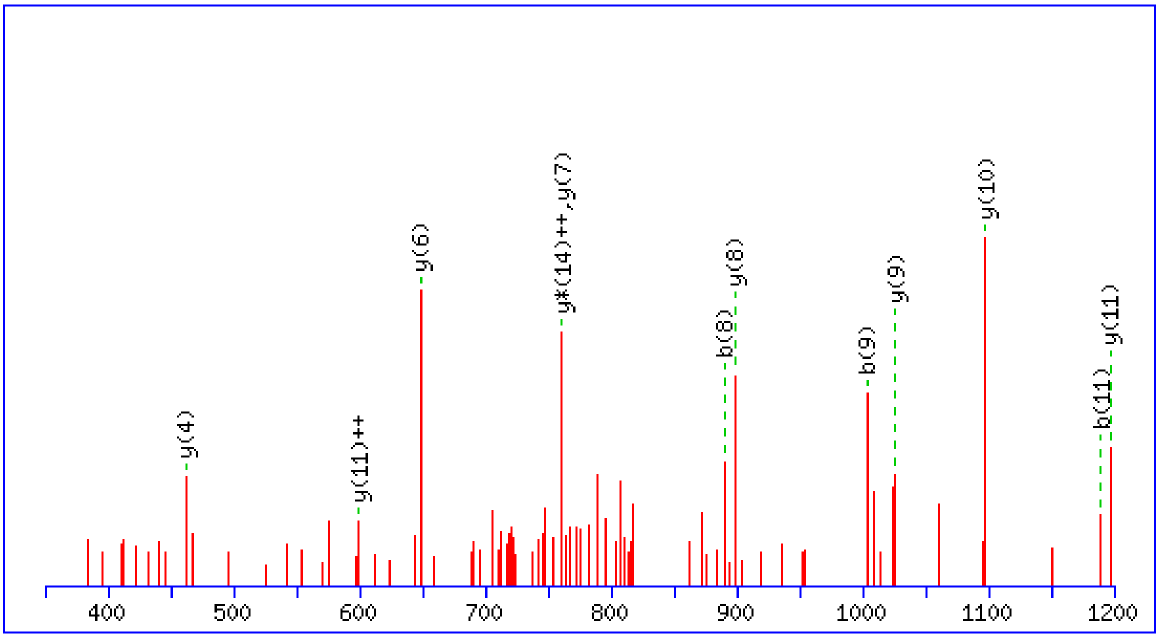

Spot no.799
ATP synthase subunit beta, mitochondrial precursor
TIAMDGTEGLVR

#	b	b^{++}	b^{0}	b^{0++}	Seq.	y	y^{++}	y*	y^{*++}	y^{0}	y^{0++}	#
1	102.055	51.5311	84.0444	42.5258	T							12
2	**215.139**	108.0731	197.1285	99.0679	I	1161.5932	581.3003	1144.5667	572.787	1143.5827	572.295	11
3	**286.1761**	143.5917	268.1656	134.5864	A	***1048.5092***	524.7582	1031.4826	516.2449	1030.4986	515.7529	10
4	417.2166	209.1119	399.2061	200.1067	M	***977.4721***	489.2397	960.4455	480.7264	959.4615	480.2344	9
5	**532.2436**	266.6254	514.233	257.6201	D	***846.4316***	423.7194	829.405	415.2062	828.421	414.7141	8
6	589.265	295.1362	571.2545	286.1309	G	***731.4046***	366.206	714.3781	357.6927	713.3941	357.2007	7
7	690.3127	345.66	672.3021	336.6547	T	***674.3832***	337.6952	657.3566	329.1819	656.3726	328.6899	6
8	**819.3553**	410.1813	801.3447	401.176	E	***573.3355***	287.1714	556.3089	278.6581	555.3249	278.1661	5
9	876.3768	438.692	858.3662	429.6867	G	***444.2929***	222.6501	427.2663	214.1368			4
10	989.4608	495.2341	971.4503	486.2288	L	***387.2714***	194.1394	370.2449	185.6261			3
11	**1088.5292**	544.7683	1070.5187	535.763	V	***274.1874***	137.5973	257.1608	129.084			2
12					R	175.119	88.0631	158.0924	79.5498			1

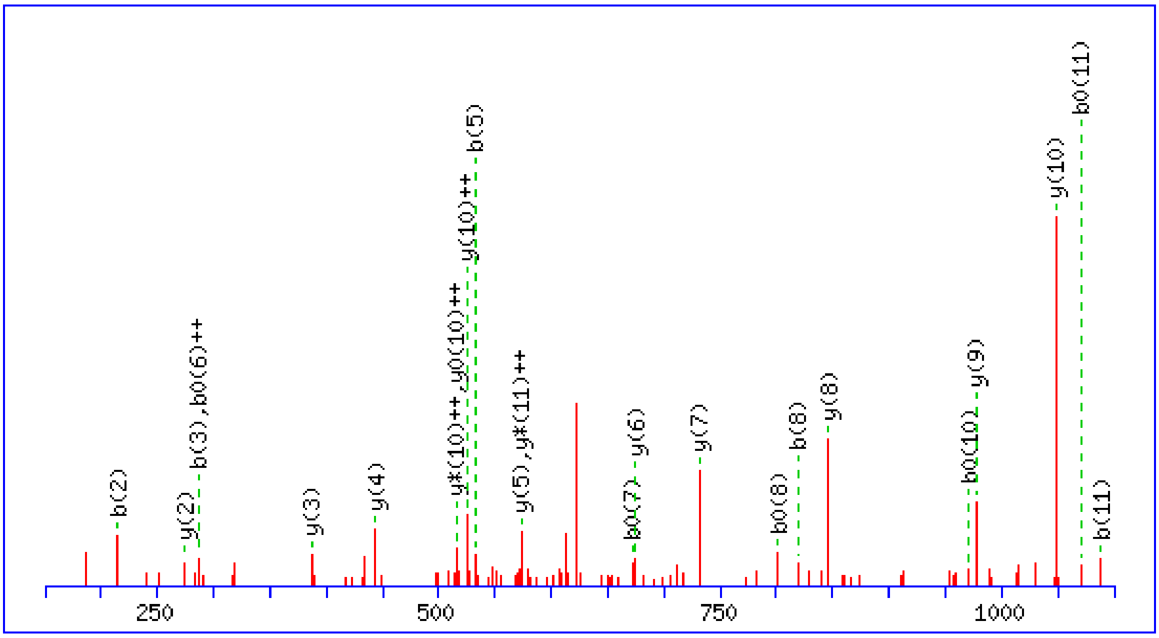

Spot no.799
ATP synthase subunit beta, mitochondrial precursor
VLDSGAPIK

#	b	b++	b0	b0++	Seq.	y	y++	y*	y*++	y0	y0++	#
1	100.0757	50.5415			V							9
2	***213.1598***	107.0835			L	***800.4512***	400.7293	783.4247	392.216	782.4407	391.724	8
3	***328.1867***	164.597	310.1761	155.5917	D	***687.3672***	344.1872	670.3406	335.674	669.3566	335.1819	7
4	415.2187	208.113	397.2082	199.1077	S	***572.3402***	286.6738	555.3137	278.1605	554.3297	277.6685	6
5	472.2402	236.6237	454.2296	227.6185	G	***485.3082***	243.1577	468.2817	234.6445			5
6	***543.2773***	272.1423	525.2667	263.137	A	428.2867	214.647	411.2602	206.1337			4
7	640.3301	320.6687	622.3195	311.6634	P	***357.2496***	179.1285	340.2231	170.6152			3
8	***753.4141***	377.2107	735.4036	368.2054	I	260.1969	130.6021	243.1703	122.0888			2
9					K	147.1128	74.06	130.0863	65.5468			1

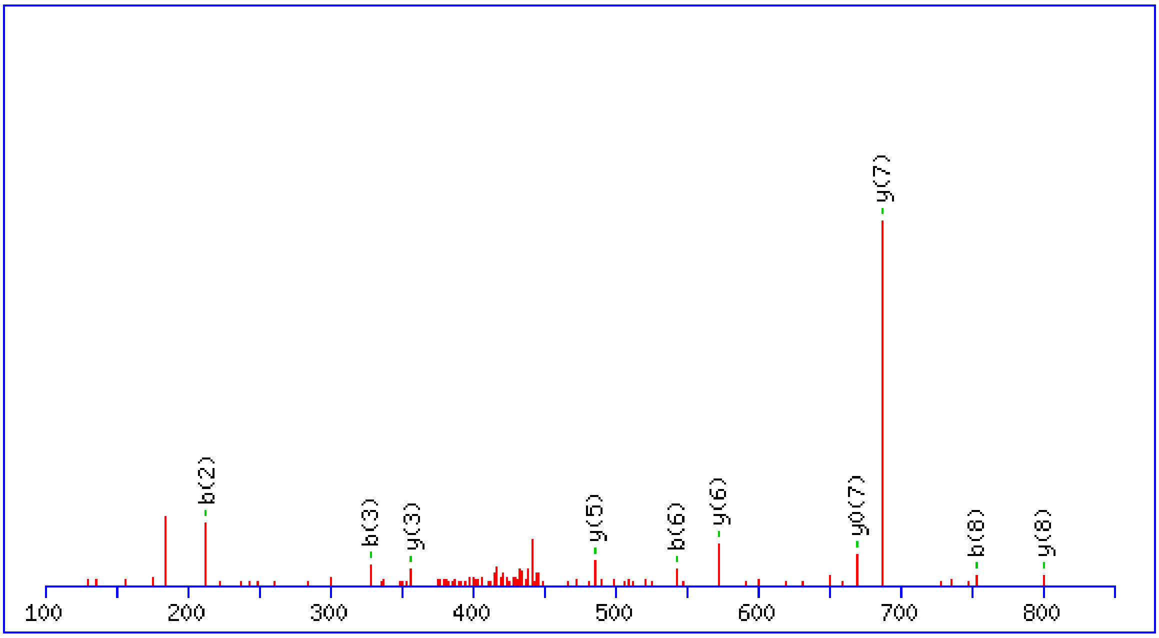

Spot no.799
ATP synthase subunit beta, mitochondrial precursor
VLDSGAPIKIPVGPETLGR

#	b	b^{++}	b*	b^{*++}	b^{0}	b^{0++}	Seq.	y	y^{++}	y*	y^{*++}	y^{0}	y^{0++}	#
1	100.0757	50.5415					V							19
2	213.1598	107.0835					L	1820.0276	910.5175	1803.0011	902.0042	1802.0171	901.5122	18
3	328.1867	164.597			310.1761	155.5917	D	1706.9436	853.9754	1689.917	845.4621	1688.933	844.9701	17
4	415.2187	208.113			397.2082	199.1077	S	1591.9166	796.4619	1574.8901	787.9487	1573.9061	787.4567	16
5	472.2402	236.6237			454.2296	227.6185	G	1504.8846	752.9459	1487.858	744.4327	1486.874	743.9407	15
6	**543.2773**	272.1423			525.2667	263.137	A	1447.8631	724.4352	1430.8366	715.9219	1429.8526	715.4299	14
7	640.3301	320.6687			622.3195	311.6634	P	1376.826	688.9166	1359.7995	680.4034	1358.8154	679.9114	13
8	**753.4141**	377.2107			735.4036	368.2054	I	1279.7732	640.3903	1262.7467	631.877	1261.7627	631.385	12
9	881.5091	441.2582	864.4825	432.7449	863.4985	432.2529	K	1166.6892	583.8482	1149.6626	575.335	1148.6786	574.8429	11
10	**994.5932**	497.8002	977.5666	489.2869	976.5826	488.7949	I	1038.5942	519.8007	1021.5677	511.2875	1020.5837	510.7955	10
11	1091.6459	546.3266	1074.6194	537.8133	1073.6354	537.3213	P	**925.5102**	463.2587	908.4836	454.7454	907.4996	454.2534	9
12	1190.7143	595.8608	1173.6878	587.3475	1172.7038	586.8555	V	828.4574	414.7323	811.4308	406.2191	810.4468	405.7271	8
13	1247.7358	624.3715	1230.7093	615.8583	1229.7252	615.3663	G	**729.389**	365.1981	712.3624	356.6849	711.3784	356.1928	7
14	1344.7886	672.8979	1327.762	664.3846	1326.778	663.8926	P	**672.3675**	336.6874	655.341	328.1741	654.357	327.6821	6
15	1473.8312	737.4192	1456.8046	728.9059	1455.8206	728.4139	E	575.3148	288.161	558.2882	279.6477	557.3042	279.1557	5
16	1574.8788	787.9431	1557.8523	779.4298	1556.8683	778.9378	T	446.2722	223.6397	429.2456	215.1264	428.2616	214.6344	4
17	1687.9629	844.4851	1670.9364	835.9718	1669.9523	835.4798	L	345.2245	173.1159	328.1979	164.6026			3
18	1744.9844	872.9958	1727.9578	864.4825	1726.9738	863.9905	G	232.1404	116.5738	215.1139	108.0606			2
19							R	175.119	88.0631	158.0924	79.5498			1

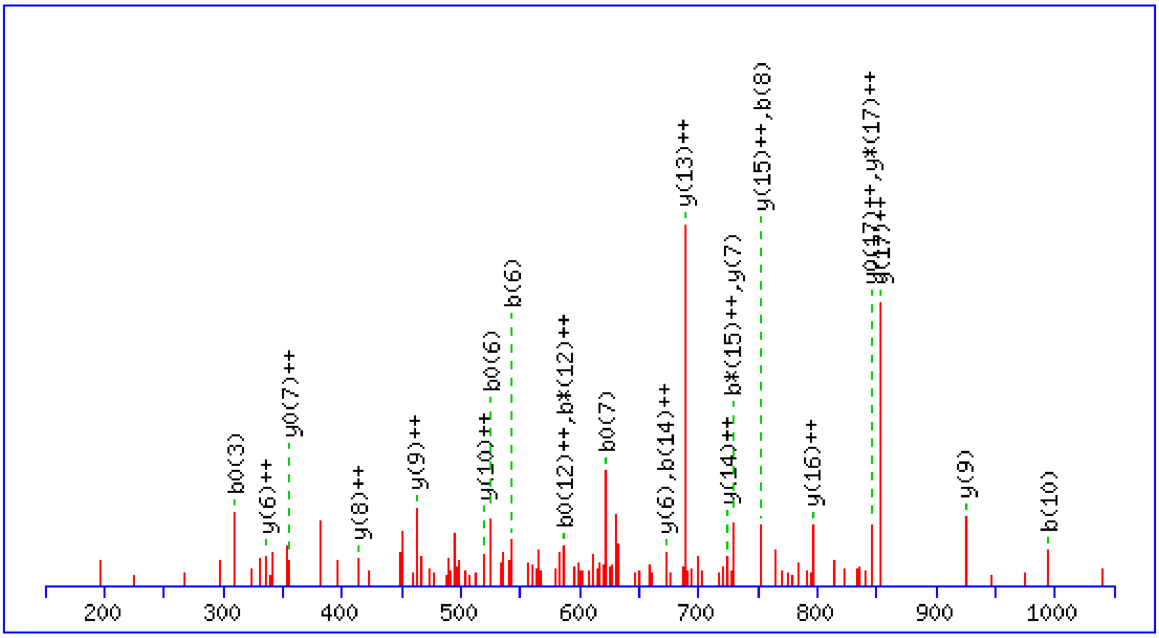

Spot no.799
ATP synthase subunit beta, mitochondrial precursor
IPVGPETLGR

#	b	b++	b0	b0++	Seq.	y	y++	y*	y*++	y0	y0++	#
1	114.0913	57.5493			I							10
2	211.1441	106.0757			P	925.5102	463.2587	908.4836	454.7454	907.4996	454.2534	9
3	310.2125	155.6099			V	828.4574	414.7323	811.4308	406.2191	810.4468	405.7271	8
4	367.234	184.1206			G	729.389	365.1981	712.3624	356.6849	711.3784	356.1928	7
5	464.2867	232.647			P	672.3675	336.6874	655.341	328.1741	654.357	327.6821	6
6	593.3293	297.1683	575.3188	288.163	E	575.3148	288.161	558.2882	279.6477	557.3042	279.1557	5
7	694.377	347.6921	676.3665	338.6869	T	446.2722	223.6397	429.2456	215.1264	428.2616	214.6344	4
8	807.4611	404.2342	789.4505	395.2289	L	345.2245	173.1159	328.1979	164.6026			3
9	864.4825	432.7449	846.472	423.7396	G	232.1404	116.5738	215.1139	108.0606			2
10					R	175.119	88.0631	158.0924	79.5498			1

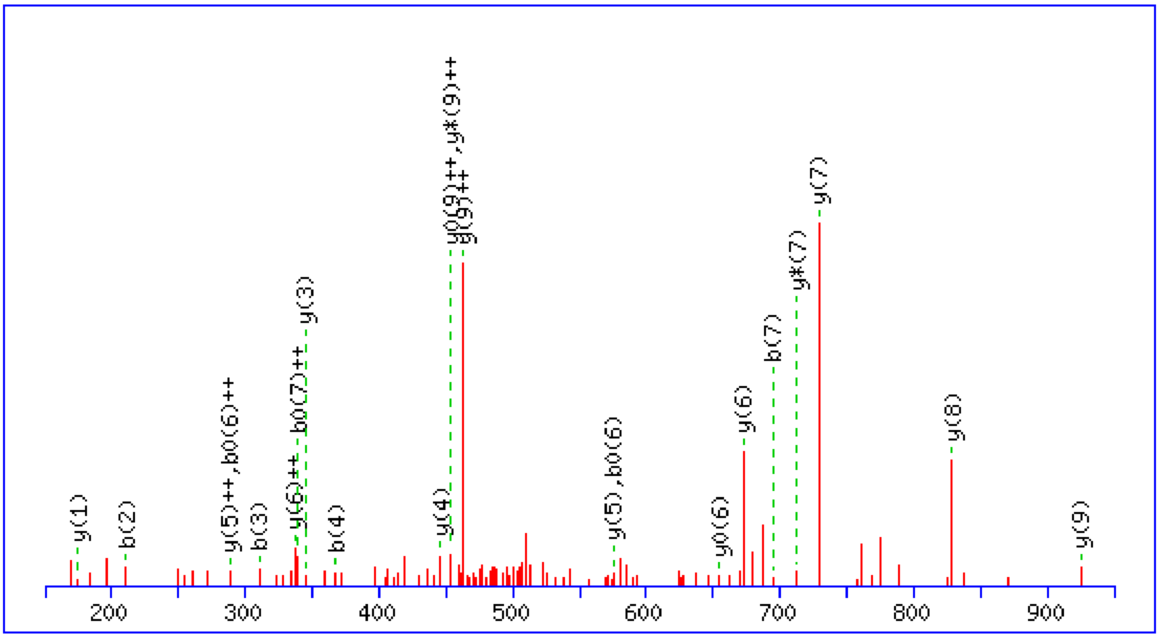

Spot no.799
ATP synthase subunit beta, mitochondrial precursor
VVDLLAPYAK

#	b	b++	b0	b0++	Seq.	y	y++	y*	y*++	y0	y0++	#
1	100.0757	50.5415			V							10
2	**199.1441**	100.0757			V	989.5666	495.2869	972.5401	486.7737	971.556	486.2817	9
3	**314.171**	157.5892	296.1605	148.5839	D	***890.4982***	445.7527	873.4716	437.2395	872.4876	436.7475	8
4	**427.2551**	214.1312	409.2445	205.1259	L	***775.4713***	388.2393	758.4447	379.726			7
5	540.3392	270.6732	522.3286	261.6679	L	***662.3872***	331.6972	645.3606	323.184			6
6	**611.3763**	306.1918	593.3657	297.1865	A	***549.3031***	275.1552	532.2766	266.6419			5
7	708.4291	354.7182	690.4185	345.7129	P	***478.266***	239.6366	461.2395	231.1234			4
8	871.4924	436.2498	853.4818	427.2445	Y	381.2132	191.1103	364.1867	182.597			3
9	942.5295	471.7684	924.5189	462.7631	A	218.1499	109.5786	201.1234	101.0653			2
10					K	147.1128	74.06	130.0863	65.5468			1

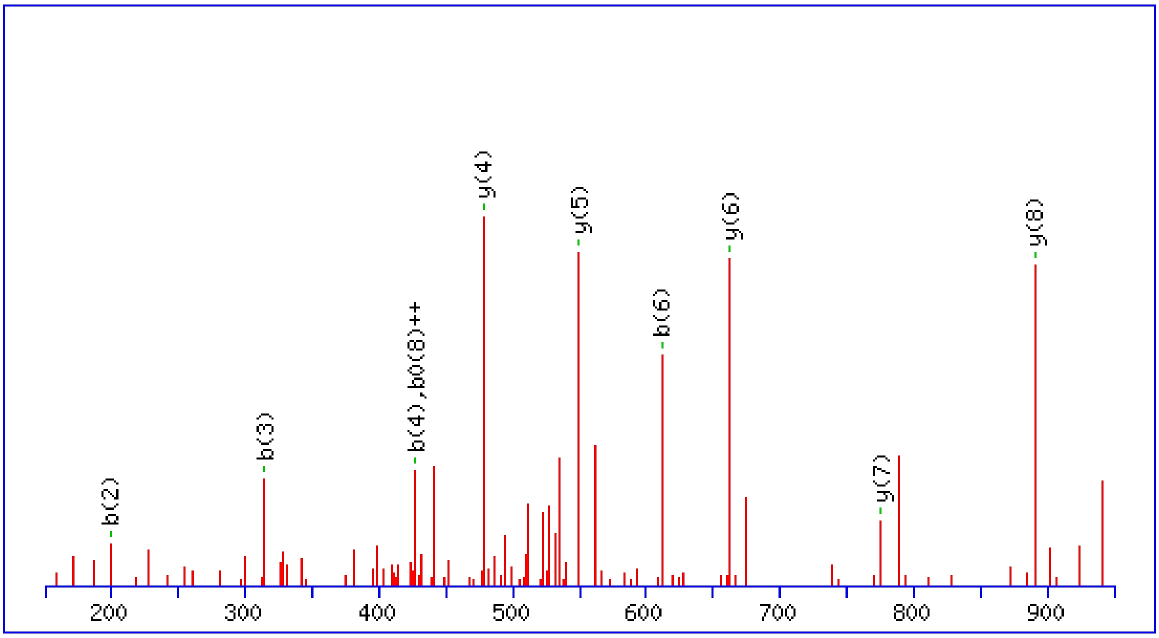

Spot no.799
ATP synthase subunit beta, mitochondrial precursor
IGLFGGAGVGK

#	b	b++	Seq.	y	y++	y*	y*++	#
1	114.0913	57.5493	I					11
2	***171.1128***	86.06	G	***862.4781***	431.7427	845.4516	423.2294	10
3	***284.1969***	142.6021	L	***805.4567***	403.232	788.4301	394.7187	9
4	***431.2653***	216.1363	F	***692.3726***	346.6899	675.3461	338.1767	8
5	488.2867	244.647	G	***545.3042***	273.1557	528.2776	264.6425	7
6	***545.3082***	273.1577	G	488.2827	244.645	471.2562	236.1317	6
7	***616.3453***	308.6763	A	***431.2613***	216.1343	414.2347	207.621	5
8	673.3668	337.187	G	***360.2241***	180.6157	343.1976	172.1024	4
9	***772.4352***	386.7212	V	***303.2027***	152.105	286.1761	143.5917	3
10	***829.4567***	415.232	G	***204.1343***	102.5708	187.1077	94.0575	2
11			K	147.1128	74.06	130.0863	65.5468	1

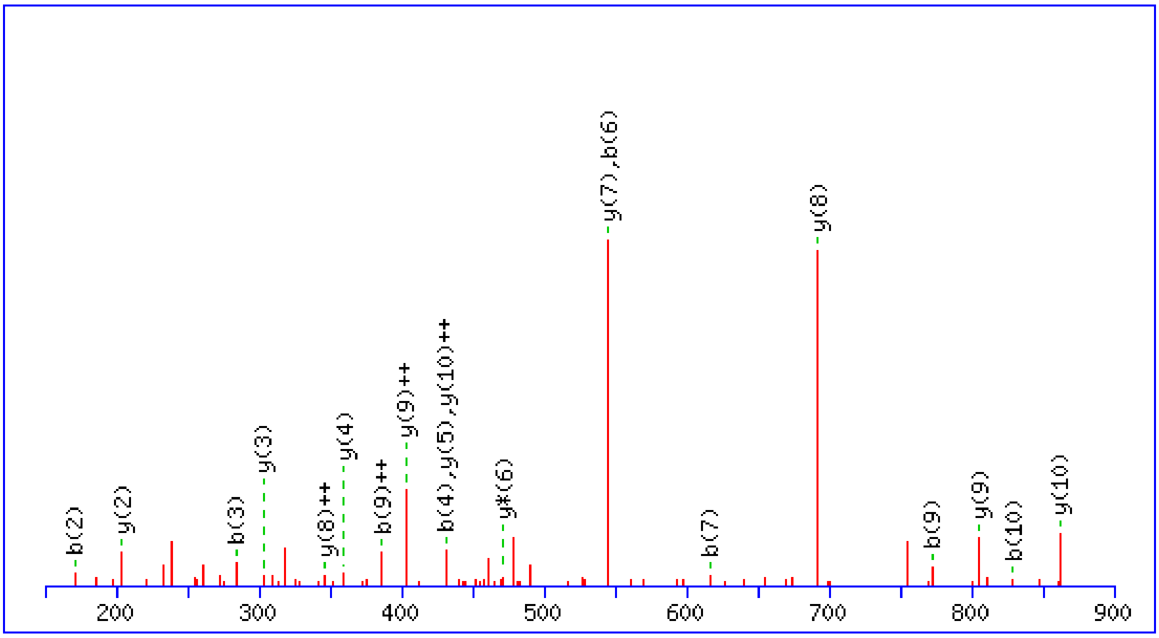

Spot no.799
ATP synthase subunit beta, mitochondrial precursor
TVLIMELINNVAK

#	b	b^{++}	b*	b^{*++}	b^{0}	b^{0++}	Seq.	y	y^{++}	y*	y^{*++}	y^{0}	y^{0++}	#
1	102.055	51.5311			84.0444	42.5258	T							13
2	201.1234	101.0653			183.1128	92.06	V	1356.7919	678.8996	1339.7654	670.3863	1338.7814	669.8943	12
3	***314.2074***	157.6074			296.1969	148.6021	L	1257.7235	629.3654	1240.697	620.8521	1239.713	620.3601	11
4	427.2915	214.1494			409.2809	205.1441	I	1144.6395	572.8234	1127.6129	564.3101	1126.6289	563.8181	10
5	558.332	279.6696			540.3214	270.6643	M	***1031.5554***	516.2813	1014.5288	507.7681	1013.5448	507.2761	9
6	687.3746	344.1909			669.364	335.1856	E	***900.5149***	450.7611	883.4884	442.2478	882.5043	441.7558	8
7	***800.4586***	400.733			782.4481	391.7277	L	***771.4723***	386.2398	754.4458	377.7265			7
8	***913.5427***	457.275			895.5321	448.2697	I	***658.3883***	329.6978	641.3617	321.1845			6
9	***1027.5856***	514.2965	1010.5591	505.7832	1009.5751	505.2912	N	***545.3042***	273.1557	528.2776	264.6425			5
10	***1141.6286***	571.3179	1124.602	562.8046	1123.618	562.3126	N	***431.2613***	216.1343	414.2347	207.621			4
11	***1240.697***	620.8521	1223.6704	612.3388	1222.6864	611.8468	V	***317.2183***	159.1128	300.1918	150.5995			3
12	***1311.7341***	656.3707	1294.7075	647.8574	1293.7235	647.3654	A	***218.1499***	109.5786	201.1234	101.0653			2
13							K	147.1128	74.06	130.0863	65.5468			1

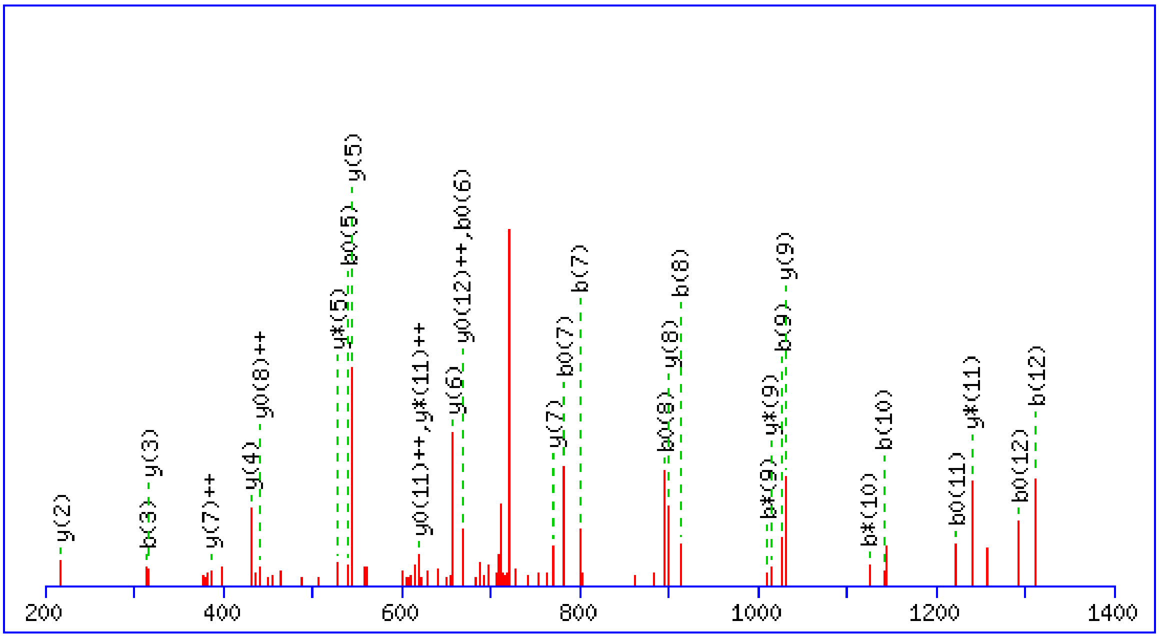

Spot no.799
ATP synthase subunit beta, mitochondrial precursor
VALVYGQMNEPPGAR

#	b	b++	b*	b*++	b0	b0++	Seq.	y	y++	y*	y*++	y0	y0++	#
1	100.0757	50.5415					V							15
2	171.1128	86.06					A	1502.742	751.8747	1485.7155	743.3614	1484.7315	742.8694	14
3	284.1969	142.6021					L	1431.7049	716.3561	1414.6784	707.8428	1413.6943	707.3508	13
4	383.2653	192.1363					V	1318.6208	659.8141	1301.5943	651.3008	1300.6103	650.8088	12
5	546.3286	273.6679					Y	***1219.5524***	610.2799	1202.5259	601.7666	1201.5419	601.2746	11
6	603.3501	302.1787					G	***1056.4891***	528.7482	1039.4626	520.2349	1038.4785	519.7429	10
7	731.4087	366.208	714.3821	357.6947			Q	999.4676	500.2375	982.4411	491.7242	981.4571	491.2322	9
8	862.4491	431.7282	845.4226	423.2149			M	***871.4091***	436.2082	854.3825	427.6949	853.3985	427.2029	8
9	976.4921	488.7497	959.4655	480.2364			N	***740.3686***	370.6879	723.342	362.1747	722.358	361.6826	7
10	**1105.5347**	553.271	1088.5081	544.7577	1087.5241	544.2657	E	***626.3257***	313.6665	609.2991	305.1532	608.3151	304.6612	6
11	1202.5874	601.7973	1185.5609	593.2841	1184.5769	592.7921	P	***497.2831***	249.1452	480.2565	240.6319			5
12	1299.6402	650.3237	1282.6136	641.8105	1281.6296	641.3184	P	400.2303	200.6188	383.2037	192.1055			4
13	**1356.6617**	678.8345	1339.6351	670.3212	1338.6511	669.8292	G	303.1775	152.0924	286.151	143.5791			3
14	1427.6988	714.353	1410.6722	705.8397	1409.6882	705.3477	A	246.1561	123.5817	229.1295	115.0684			2
15							R	175.119	88.0631	158.0924	79.5498			1

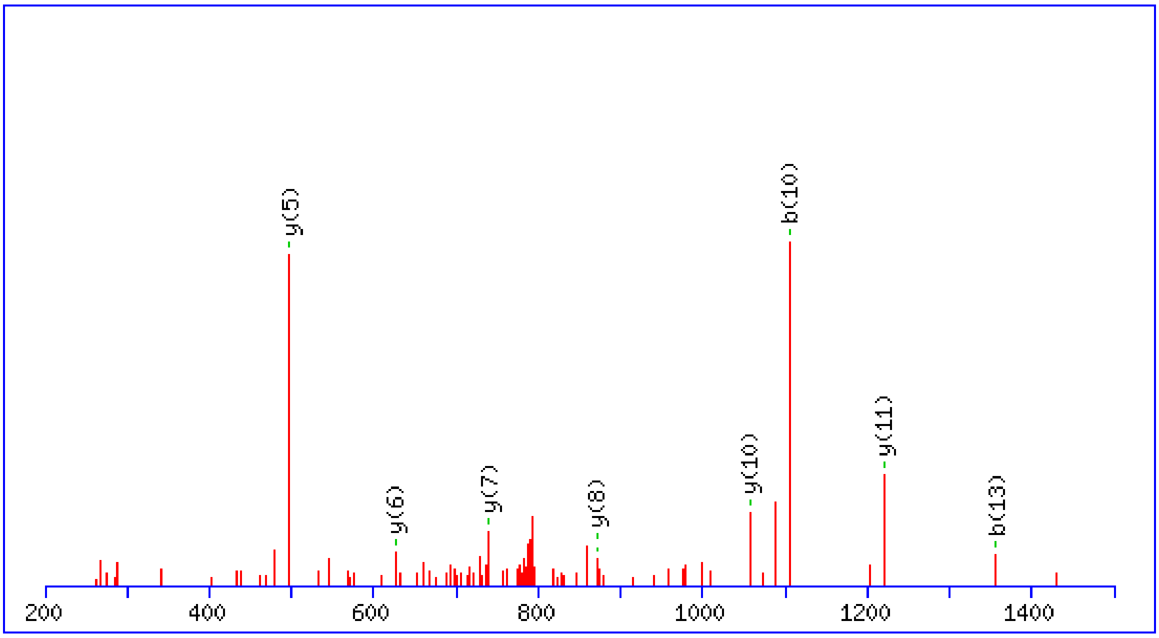

Spot no.799
ATP synthase subunit beta, mitochondrial precursor
VALTGLTVAEYFR

#	b	b^{++}	b^{0}	b^{0++}	Seq.	y	y^{++}	y*	y^{*++}	y^{0}	y^{0++}	#
1	100.0757	50.5415			V							13
2	171.1128	86.06			A	1340.7209	670.8641	1323.6943	662.3508	1322.7103	661.8588	12
3	284.1969	142.6021			L	1269.6838	635.3455	1252.6572	626.8322	1251.6732	626.3402	11
4	385.2445	193.1259	367.234	184.1206	T	***1156.5997***	578.8035	1139.5732	570.2902	1138.5891	569.7982	10
5	442.266	221.6366	424.2554	212.6314	G	***1055.552***	528.2796	1038.5255	519.7664	1037.5415	519.2744	9
6	**555.3501**	278.1787	537.3395	269.1734	L	***998.5306***	499.7689	981.504	491.2556	980.52	490.7636	8
7	656.3978	328.7025	638.3872	319.6972	T	***885.4465***	443.2269	868.4199	434.7136	867.4359	434.2216	7
8	755.4662	378.2367	737.4556	369.2314	V	***784.3988***	392.703	767.3723	384.1898	766.3883	383.6978	6
9	**826.5033**	413.7553	808.4927	404.75	A	***685.3304***	343.1688	668.3039	334.6556	667.3198	334.1636	5
10	**955.5459**	478.2766	937.5353	469.2713	E	***614.2933***	307.6503	597.2667	299.137	596.2827	298.645	4
11	**1118.6092**	559.8082	1100.5986	550.803	Y	***485.2507***	243.129	468.2241	234.6157			3
12	1265.6776	633.3424	1247.6671	624.3372	F	***322.1874***	161.5973	305.1608	153.084			2
13					R	175.119	88.0631	158.0924	79.5498			

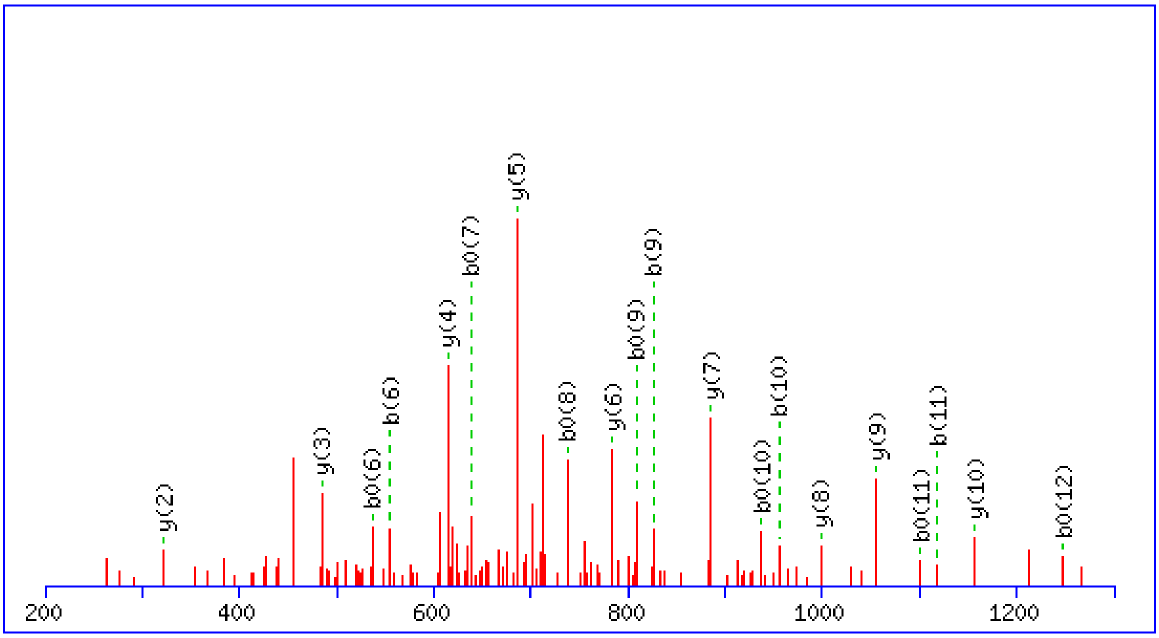

Spot no.799
ATP synthase subunit beta, mitochondrial precursor
FTQAGSEVSALLGR

#	b	b++	b*	b*++	b0	b0++	Seq.	y	y++	y*	y*++	y0	y0++	#
1	148.0757	74.5415					F							14
2	249.1234	125.0653			231.1128	116.06	T	1288.6856	644.8464	1271.659	636.3331	1270.675	635.8411	13
3	**377.1819**	189.0946	360.1554	180.5813	359.1714	180.0893	Q	1187.6379	594.3226	1170.6113	585.8093	1169.6273	585.3173	12
4	**448.2191**	224.6132	431.1925	216.0999	430.2085	215.6079	A	***1059.5793***	530.2933	1042.5527	521.78	1041.5687	521.288	11
5	505.2405	253.1239	488.214	244.6106	487.23	244.1186	G	***988.5422***	494.7747	971.5156	486.2615	970.5316	485.7694	10
6	592.2726	296.6399	575.246	288.1266	574.262	287.6346	S	***931.5207***	466.264	914.4942	457.7507	913.5102	457.2587	9
7	721.3151	361.1612	704.2886	352.6479	703.3046	352.1559	E	***844.4887***	422.748	827.4621	414.2347	826.4781	413.7427	8
8	**820.3836**	410.6954	803.357	402.1821	802.373	401.6901	V	***715.4461***	358.2267	698.4196	349.7134	697.4355	349.2214	7
9	907.4156	454.2114	890.389	445.6982	889.405	445.2061	S	***616.3777***	308.6925	599.3511	300.1792	598.3671	299.6872	6
10	978.4527	489.73	961.4262	481.2167	960.4421	480.7247	A	***529.3457***	265.1765	512.3191	256.6632			5
11	**1091.5368**	546.272	1074.5102	537.7587	1073.5262	537.2667	L	***458.3085***	229.6579	441.282	221.1446			4
12	**1204.6208**	602.8141	1187.5943	594.3008	1186.6103	593.8088	L	***345.2245***	173.1159	328.1979	164.6026			3
13	**1261.6423**	631.3248	1244.6157	622.8115	1243.6317	622.3195	G	***232.1404***	116.5738	215.1139	108.0606			2
14							R	175.119	88.0631	158.0924	79.5498			1

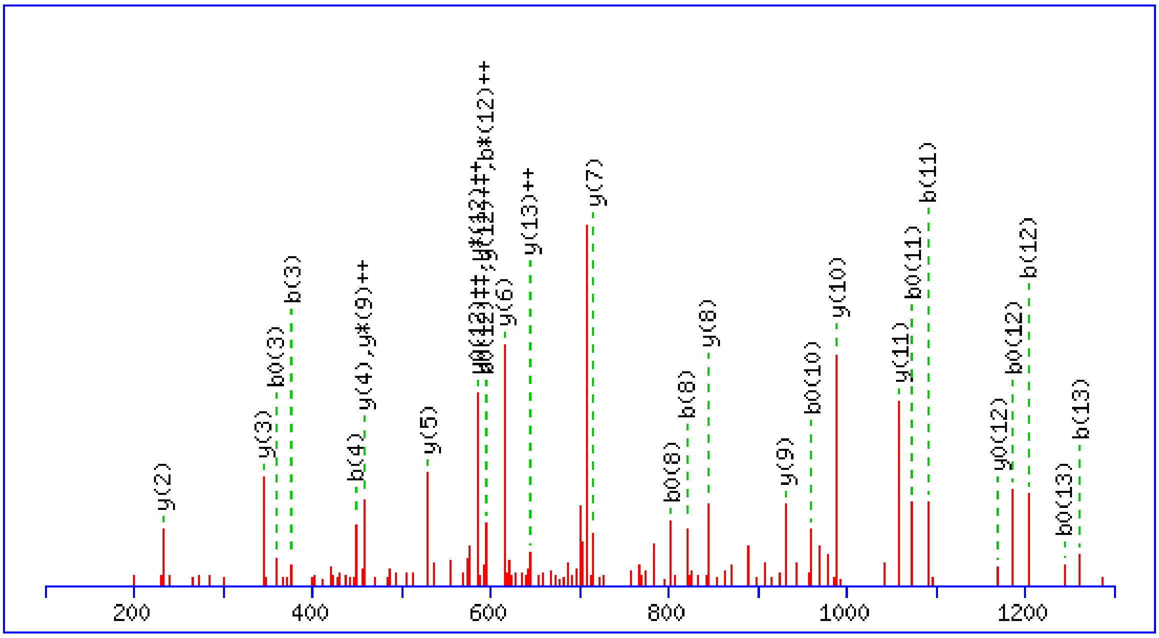

Spot no.799
ATP synthase subunit beta, mitochondrial precursor
AIAELGIYPAVDPLDSTSR

#	b	b^{++}	b^{0}	b^{0++}	Seq.	y	y^{++}	y*	y^{*++}	y^{0}	y^{0++}	#
1	72.0444	36.5258			A							19
2	185.1285	93.0679			I	1916.9964	959.0018	1899.9698	950.4886	1898.9858	949.9965	18
3	256.1656	128.5864			A	1803.9123	902.4598	1786.8858	893.9465	1785.9018	893.4545	17
4	385.2082	193.1077	367.1976	184.1024	E	1732.8752	866.9412	1715.8487	858.428	1714.8646	857.936	16
5	498.2922	249.6498	480.2817	240.6445	L	1603.8326	802.4199	1586.8061	793.9067	1585.822	793.4147	15
6	**555.3137**	278.1605	537.3031	269.1552	G	***1490.7486***	745.8779	1473.722	737.3646	1472.738	736.8726	14
7	**668.3978**	334.7025	650.3872	325.6972	I	***1433.7271***	717.3672	1416.7005	708.8539	1415.7165	708.3619	13
8	**831.4611**	416.2342	813.4505	407.2289	Y	***1320.643***	660.8251	1303.6165	652.3119	1302.6325	651.8199	12
9	**928.5138**	464.7606	910.5033	455.7553	P	***1157.5797***	579.2935	1140.5531	570.7802	1139.5691	570.2882	11
10	999.551	500.2791	981.5404	491.2738	A	***1060.5269***	530.7671	1043.5004	522.2538	1042.5164	521.7618	10
11	**1098.6194**	549.8133	1080.6088	540.808	V	989.4898	495.2485	972.4633	486.7353	971.4793	486.2433	9
12	**1213.6463**	607.3268	1195.6358	598.3215	D	***890.4214***	445.7143	873.3949	437.2011	872.4108	436.7091	8
13	1310.6991	655.8532	1292.6885	646.8479	P	***775.3945***	388.2009	758.3679	379.6876	757.3839	379.1956	7
14	1423.7831	712.3952	1405.7726	703.3899	L	678.3417	339.6745	661.3151	331.1612	660.3311	330.6692	6
15	1538.8101	769.9087	1520.7995	760.9034	D	***565.2576***	283.1325	548.2311	274.6192	547.2471	274.1272	5
16	1625.8421	813.4247	1607.8316	804.4194	S	***450.2307***	225.619	433.2041	217.1057	432.2201	216.6137	4
17	1726.8898	863.9485	1708.8792	854.9433	T	363.1987	182.103	346.1721	173.5897	345.1881	173.0977	3
18	1813.9218	907.4645	1795.9113	898.4593	S	262.151	131.5791	245.1244	123.0659	244.1404	122.5738	2
19					R	175.119	88.0631	158.0924	79.5498			1

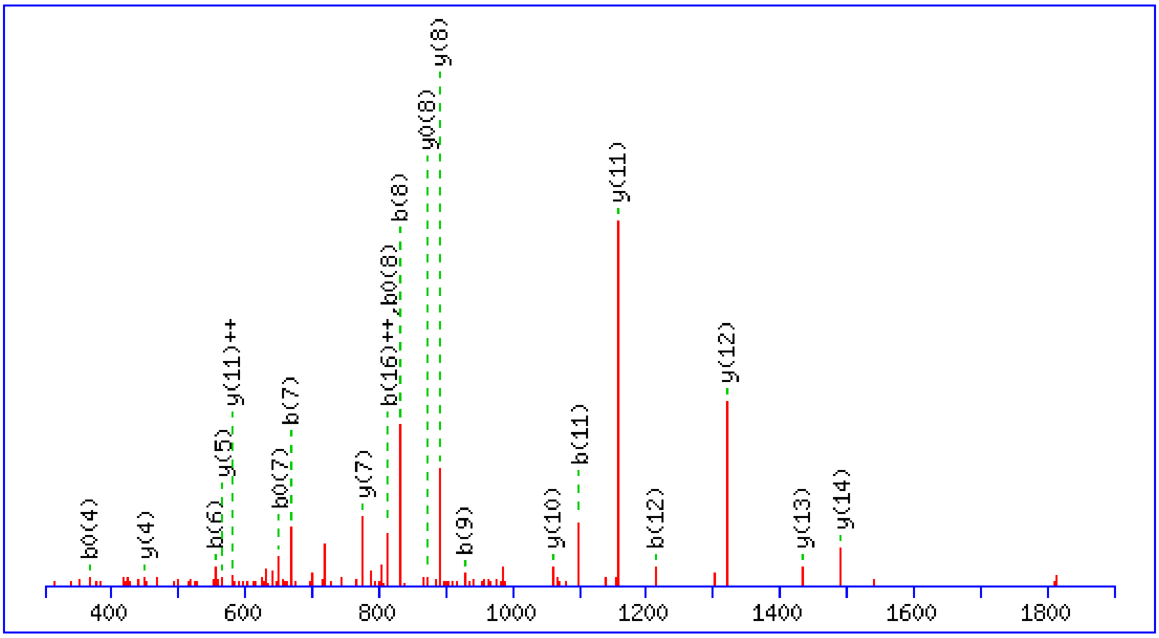

Figure S3

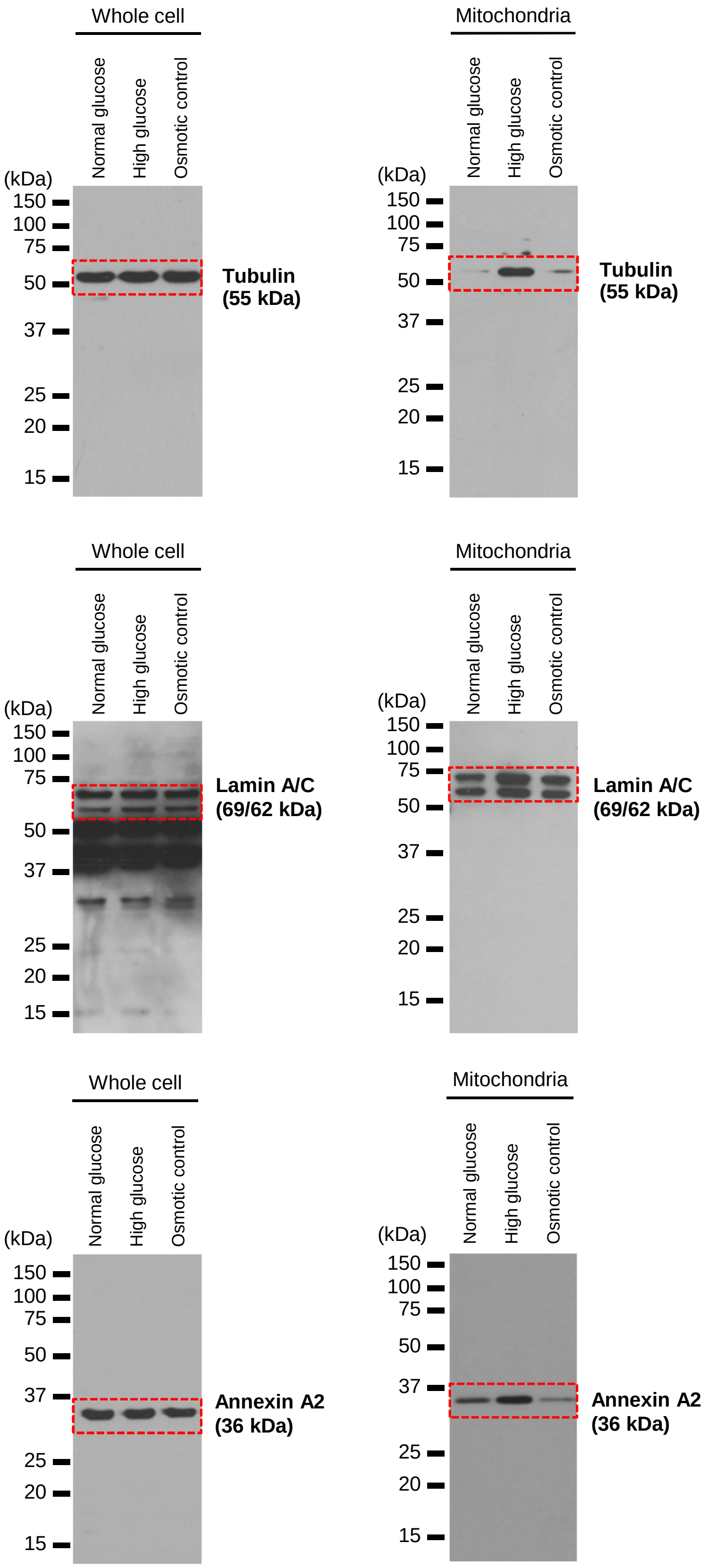

Supplementary Figure S3: Full-length blots of the cropped images shown in **Figure 4A**. The cropped areas are labeled with red-dotted boxes.

Figure S4

Pro-Q Diamond phosphoprotein gel staining

Normal glucose High glucose Osmotic control

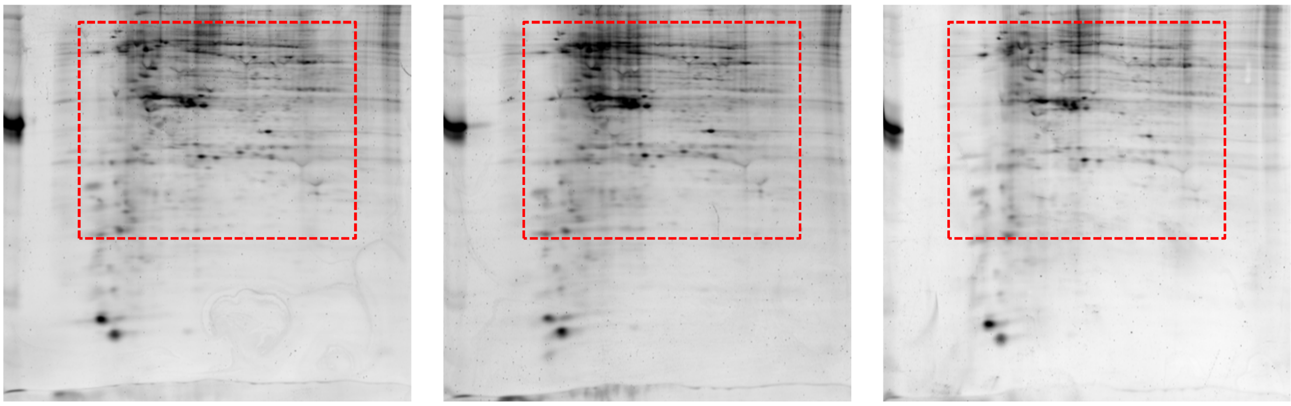

SYPRO Ruby total protein gel staining

Normal glucose High glucose Osmotic control

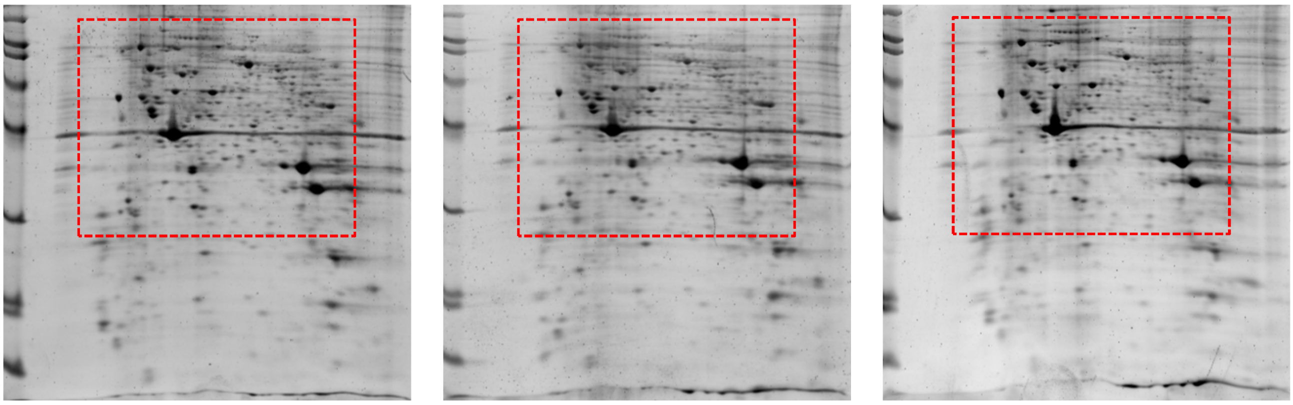

Supplementary Figure S4: Whole-gel images of the cropped (zoom-in) areas shown in **Figure 6A**. The cropped areas are labeled with red-dotted boxes.

Supplementary Figure S5: Illustrative MS/MS spectra of phosphorylated peptide identified from each protein spot.

Spot no.181
1 phosphorylation site at threonine

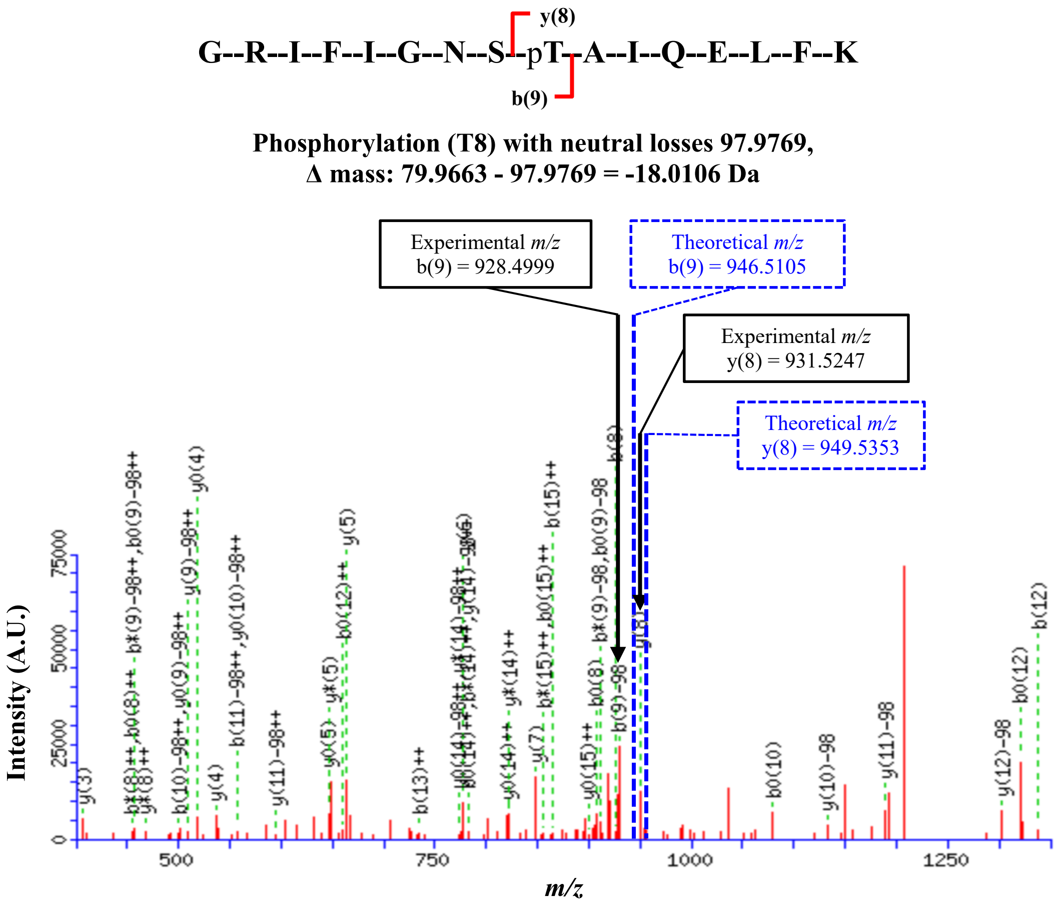

Spot no.610
2 phosphorylation sites at threonine

L--L--A--Q--K--A--A--G--pT--F--E--Q--D--P--pT--G--F--A--R

y(11) **b(9)** **y(5)** **b(15)**

Phosphorylation (T9) with neutral losses 97.9769,
Phosphorylation (T15) with neutral losses 97.9769,
Δ mass : 2 × (79.9663 - 97.9769) = -36.0212 Da

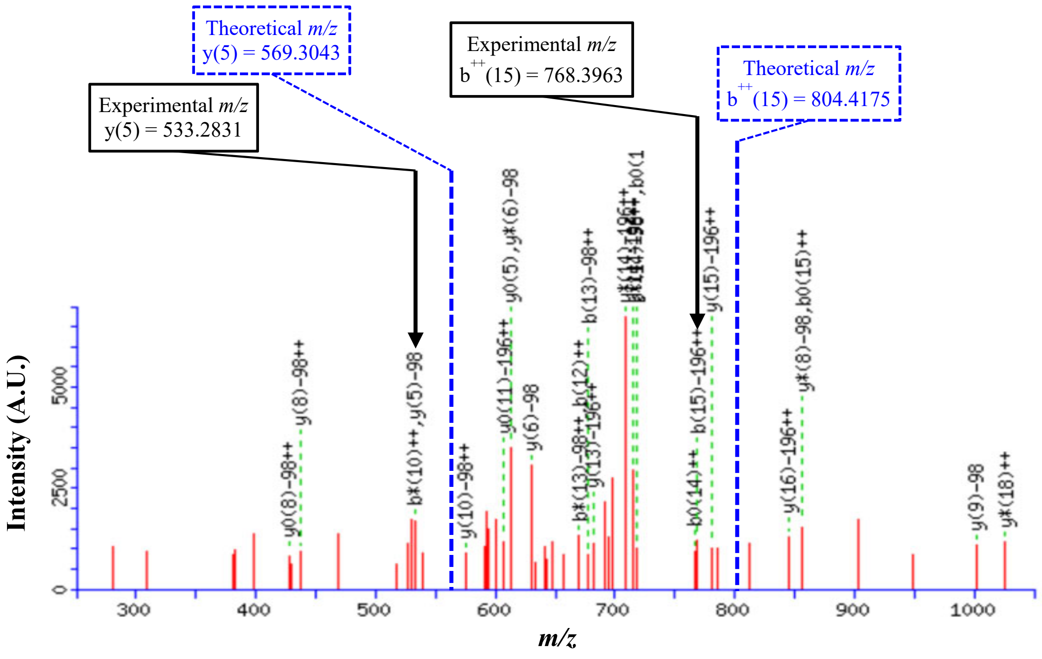

Spot no. 661
2 phosphorylation sites at serine

G--F--S--S--G--S--A--V--V--pS--G--G--pS--R

y(5), y(2), b(10), b(13)

Phosphorylation (S10) with neutral losses 97.9769,
Phosphorylation (S13) with neutral losses 97.9769,
Δ mass : 2 × (79.9663 - 97.9769) = -36.0212 Da

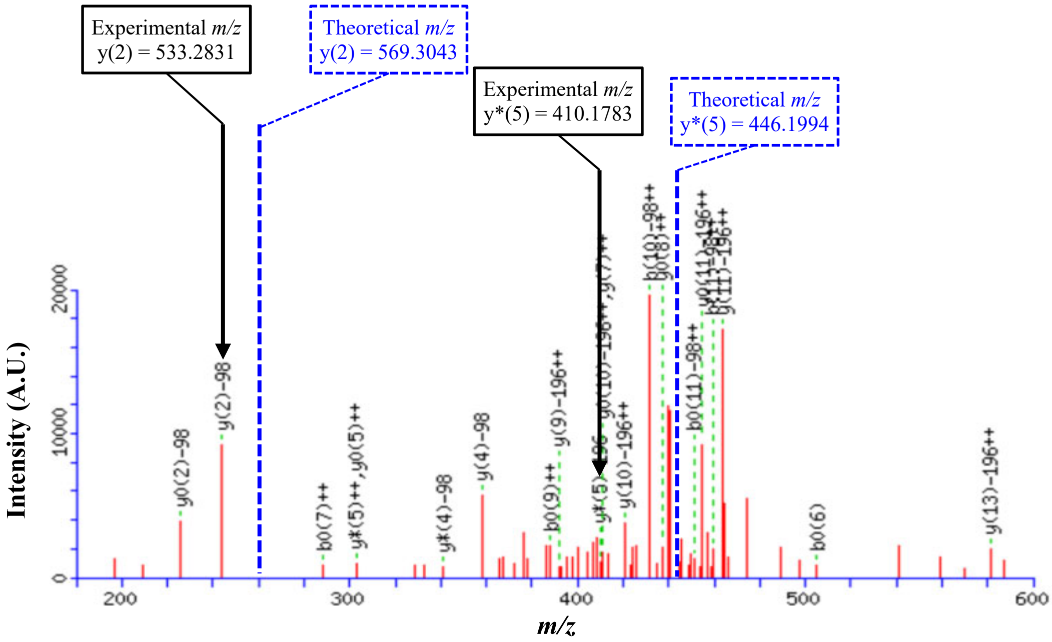

Spot no. 276
1 phosphorylation site at threonine

y(10)

A--E--H--pT--R--S--P--S--A--T--L--L--R

b(4)

Phosphorylation (T4) with neutral losses 97.9769,
Δ mass : 79.9663 - 97.9769 = -18.0106 Da

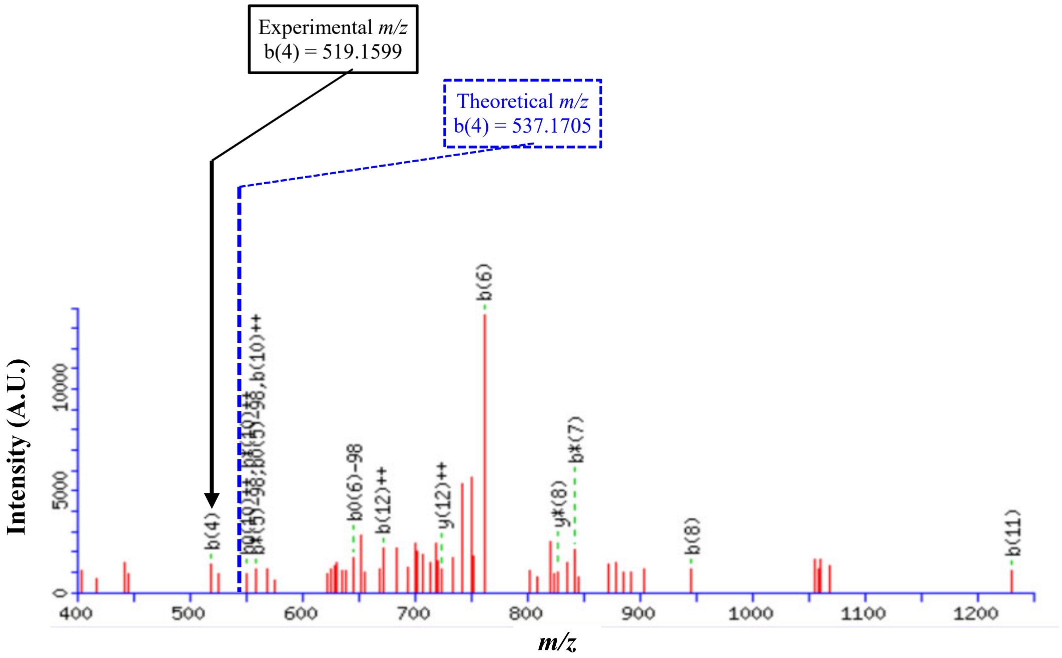

Spot no. 655
1 phosphorylation site at serine

y(3)
G--V--G--S--pS--S--R
b(5)

Phosphorylation (S5) with neutral losses 97.9769,
Δ mass : 79.9663 - 97.9769 = -18.0106 Da

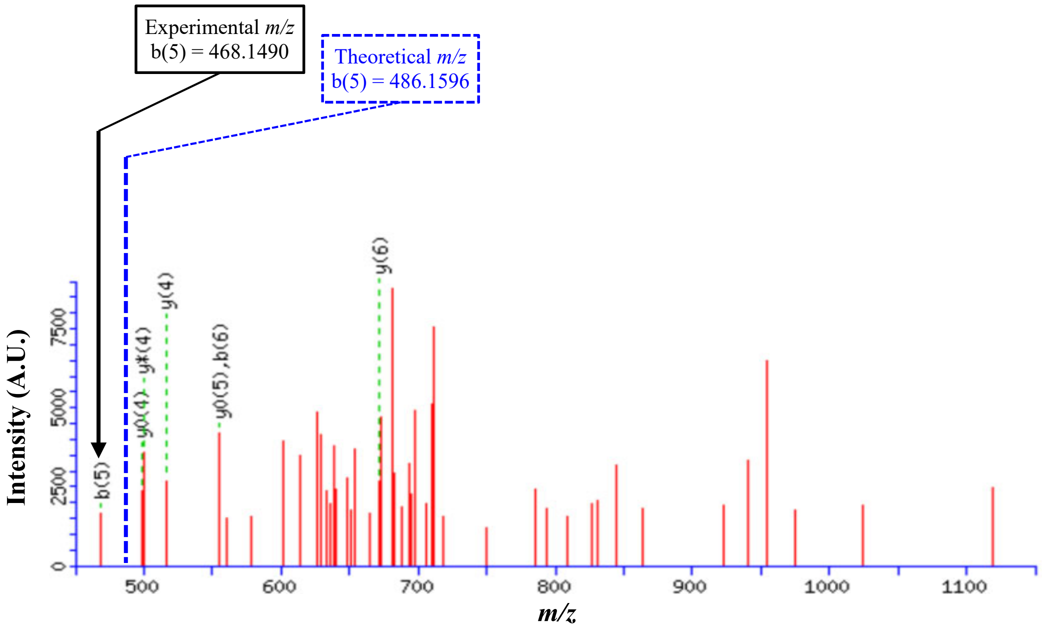

Spot no.799
1 phosphorylation site at serine

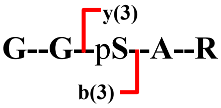

Phosphorylation (T8) with neutral losses 97.9769,
Δ mass : 79.9663 - 97.9769 = -18.0106 Da

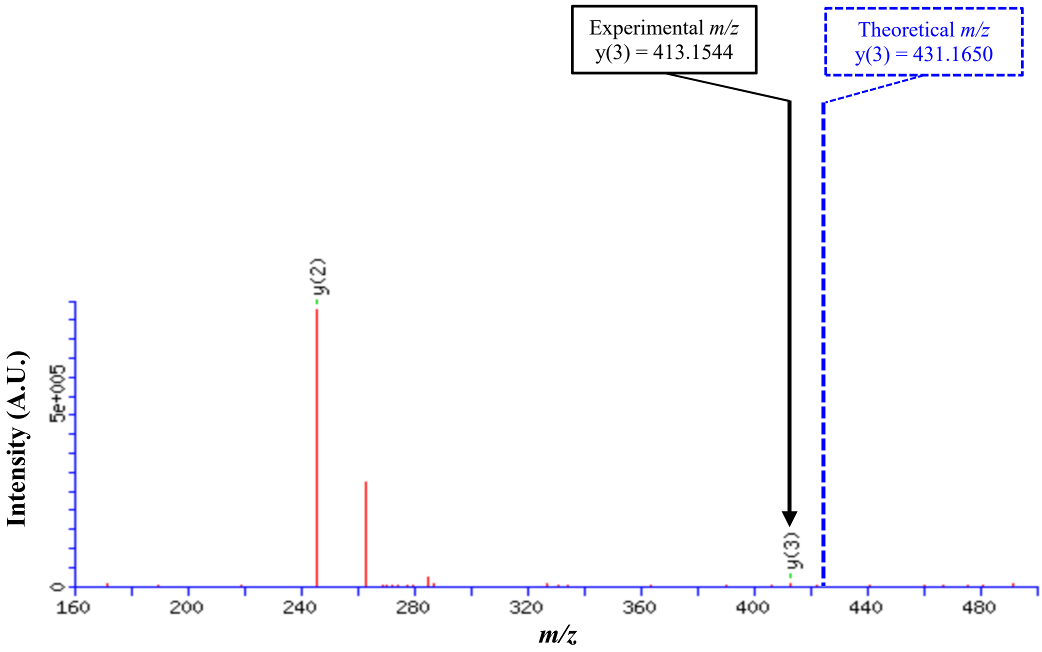

Supplementary Figure S6: Illustrative MS/MS spectra of oxidatively modified peptide identified from each protein spot.

Spot no.181
Dioxidation at methionine

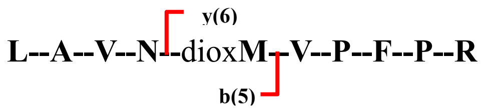

Dioxidation (M5) with neutral losses 0.0000,
Δ mass: 2 × 15.9949 = 31.9898 Da

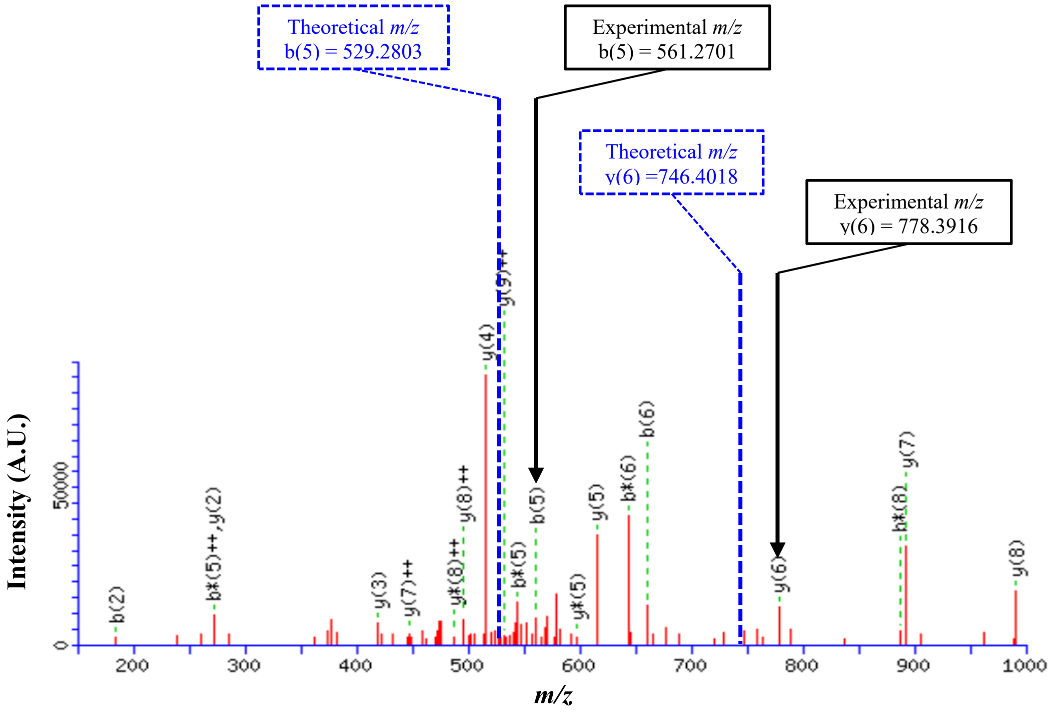

Spot no.610
Oxidation at methionine

y(6)

E--G--D--L--oxM--A--A--Q--A--R

b(5)

Oxidation (M5) with neutral losses 0.0000,
Δ mass : 15.9949 Da

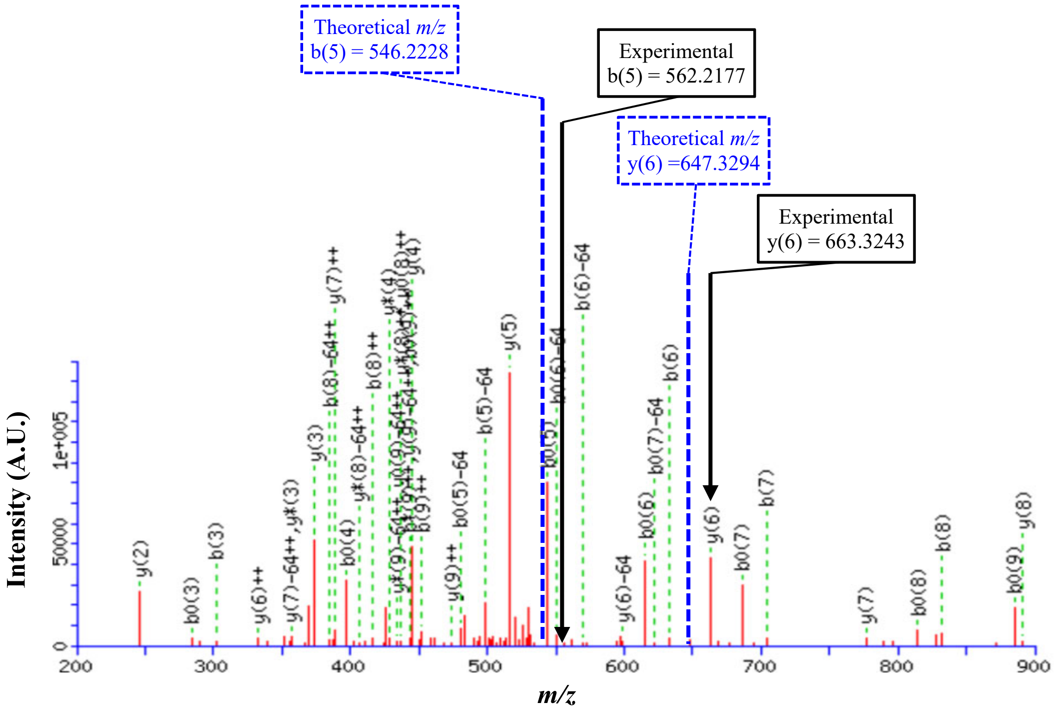

Spot no. 661
Oxidation at methionine

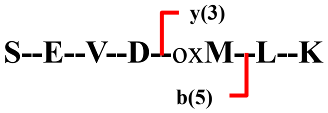

Oxidation (M5) with neutral losses 0.0000,
Δ mass : 15.9949 Da

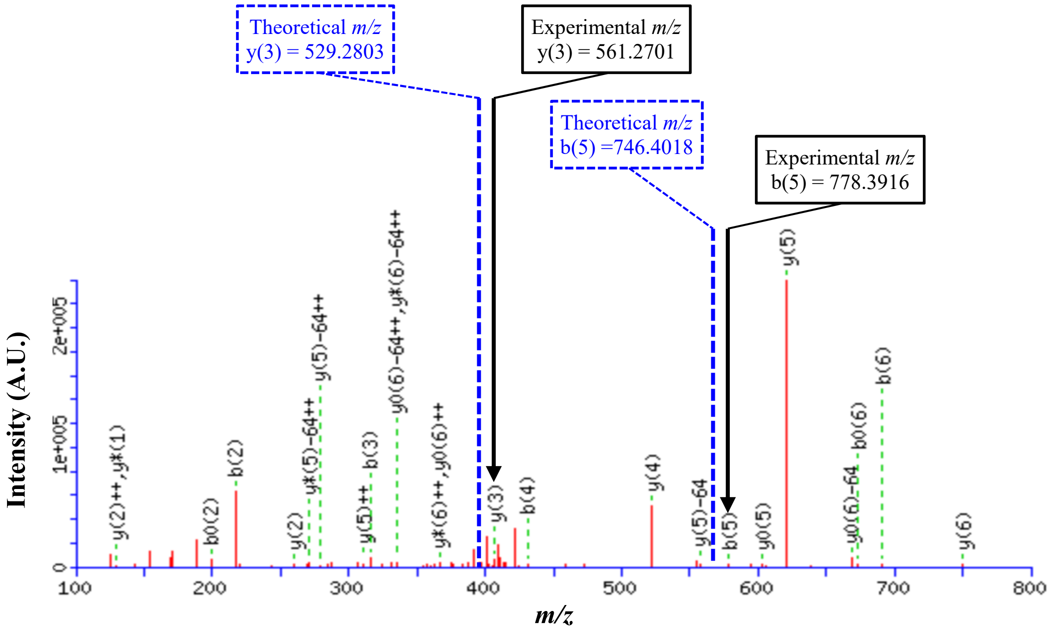

Spot no. 276
Oxidation at methionine

y(3)

Q--L--E--I--oxM--G--K

b(5)

Oxidation (M5) with neutral losses 0.0000,
Δ mass : 15.9949 Da

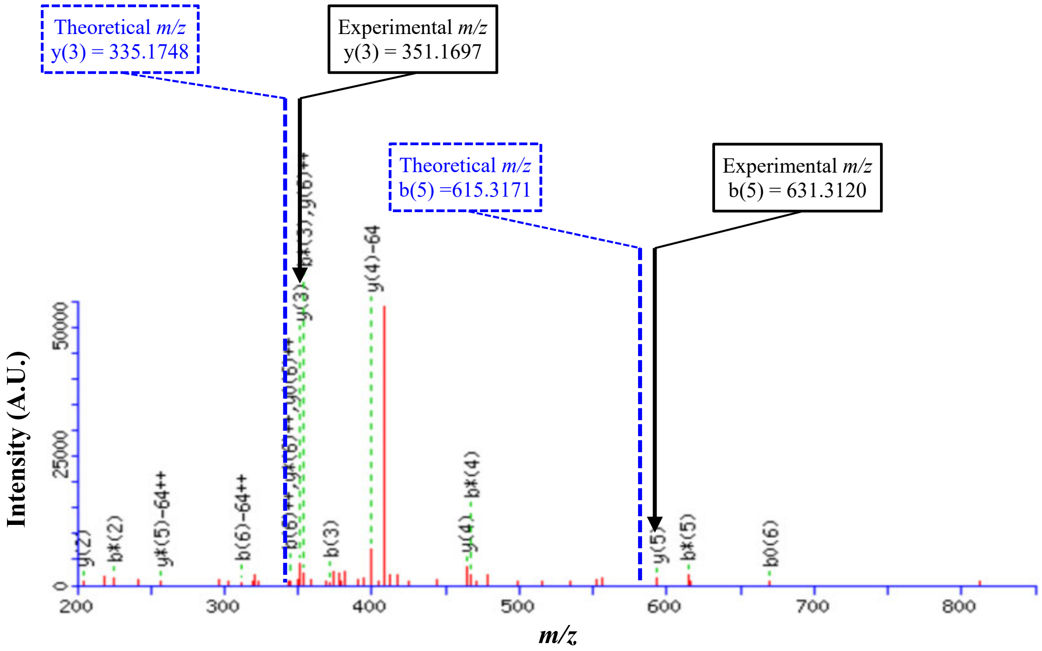

Spot no. 655
Oxidation at cysteine

y(3)
D--V--T--I--A--D--oxC**--G--K**
b(7)

Oxidation (M5) with neutral losses 0.0000,
Δ mass : 15.9949 Da

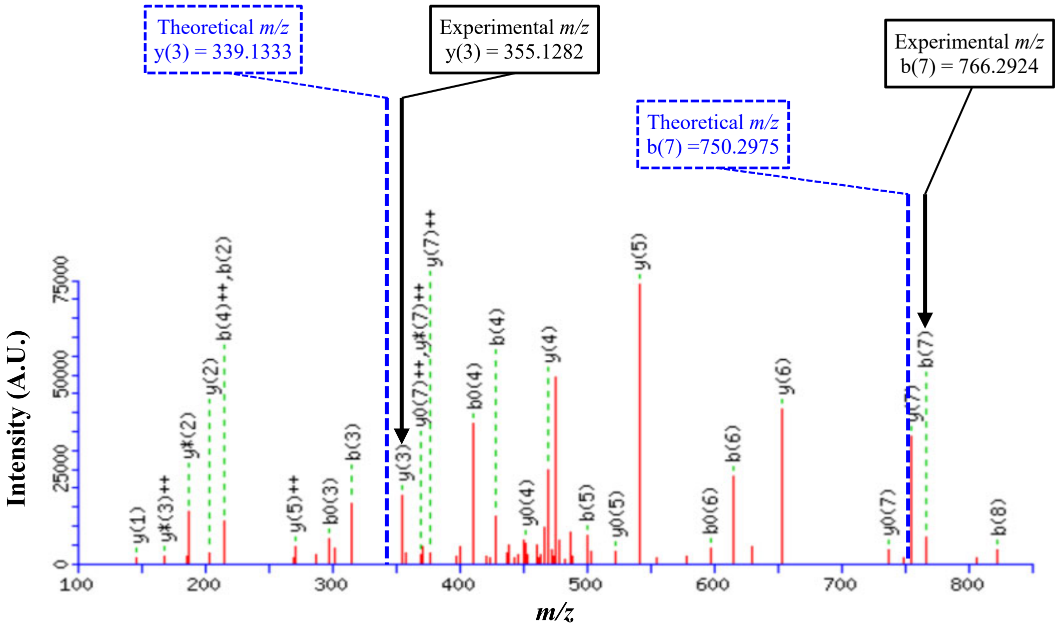

Spot no.799
Dioxidation at methionine

y(12)

A--I--A--E--L--G--I--dioxM**--P--A--V--D--P--L--D--S--T--S--R**

b(8)

Dioxidation (M5) with neutral losses 0.0000,
Δ mass : 2 × 15.9949 = 31.9898 Da

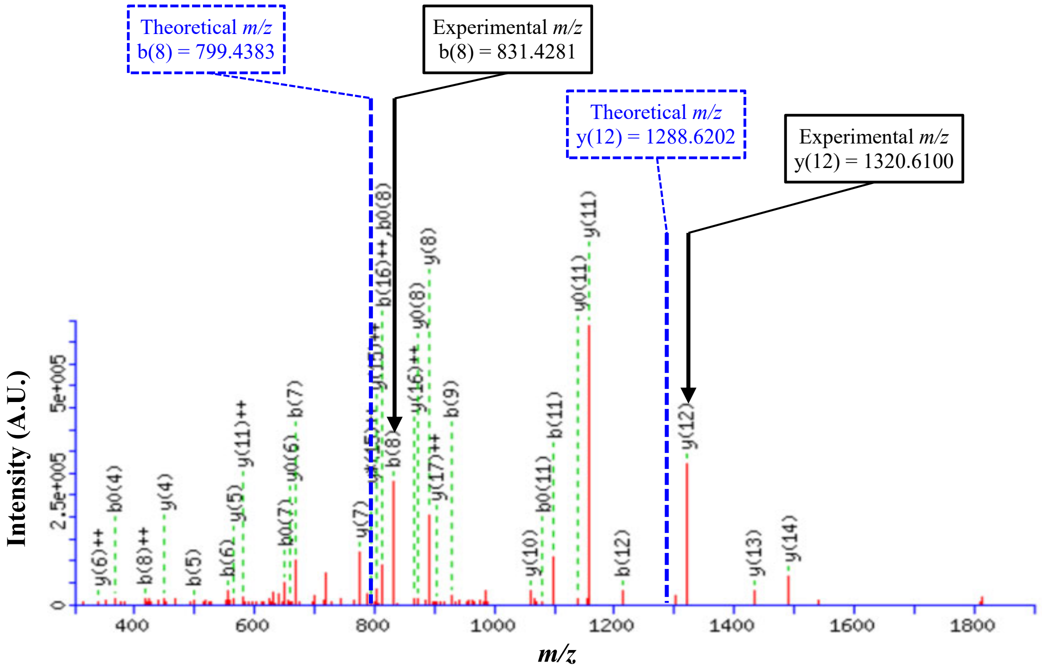

Supplementary Table S1: Summary of all distinct peptides matched in each identified protein.

Spot no. 181 (Tubulin beta-2A chain)

Peptide no.	Sequence	Residue	Ions score	Charge state	Observed m/z (Da)	Precursor m/Z (Da)	Mass error observed (Da)	Modification
1	AILVDLEPGTMDSVR	63-77	70	2+	816.4490	1630.8834	0.0598	Oxidation
2	IREEYPDR	155-162	45	2+	539.3850	1076.7554	0.2304	-
3	IMNTFSVMPSPK	163-174	67	2+	684.4970	1366.9794	0.3170	Oxidation
4	FPGQLNADLR	242-251	55	2+	565.9050	1129.7954	0.2075	-
5	LAVNMVPFPR	253-262	73	2+	580.4300	1158.8454	0.2235	Oxidation
6	LHFFMPGFAPLTSR	263-276	46	2+	810.9900	1619.9654	0.1372	-
7	ALTVPELTQQMFDSK	283-297	62	2+	862.4780	1722.9414	0.0916	Oxidation
8	YLTVAAIFR	310-318	46	2+	526.8380	1051.6614	-0.9404	-
9	MSMKEVDEQMLNVQNK	321-336	48	2+	658.0580	1971.1522	0.2774	Oxidation
10	EVDEQMLNVQNK	325-336	61	2+	731.8750	1461.7354	0.0585	Oxidation
11	NSSYFVEWIPNNVK	337-350	69	2+	848.9810	1695.9474	0.1218	-
12	TAVCDIPPR	351-359	60	2+	514.8050	1027.5954	0.0834	-
13	MSATFIGNSTAIQELFK	363-379	99	2+	937.9850	1873.9554	1.0263	Oxidation
14	ISEQFTAMFR	381-390	78	2+	623.3490	1244.6834	0.0975	Oxidation

Spot no. 610 (Prelamin A/C)

Peptide no.	Sequence	Residue	Ions score	Charge state	Observed m/z (Da)	Precursor m/Z (Da)	Mass error observed (Da)	Modification
1	SGAQASSTPLSPTR	12-25	43	2+	680.9260	1359.8374	1.1584	-
2	LQEKEDLQELNDR	29-41	47	2+	815.4070	1628.7994	-0.0011	-
3	EDLQELNDR	33-41	41	2+	566.3650	1130.7154	0.1951	-
4	LAVYIDR	42-48	44	2+	425.2870	848.5594	0.0839	-
5	SLETENAGLR	51-60	61	2+	545.3560	1088.6974	0.1513	-
6	ITESEEVVSR	63-72	89	2+	574.8760	1147.7374	0.1654	-
7	AAYEAELGDAR	79-89	76	2+	583.3190	1164.6234	0.0824	-
8	KTLDSVAK	90-97	29	2+	431.3500	860.6854	0.1887	-
9	EGDLMAAQAR	124-133	69	2+	539.3740	1076.7334	0.2414	Oxidation
10	EAALSTALSEK	145-155	33	2+	560.3280	1118.6414	0.0596	-
11	TLEGELHDLR	157-166	43	2+	591.9010	1181.7874	0.1834	-
12	LVEIDNGK	226-233	24	2+	444.3380	886.6614	0.1855	-
13	LADALQELR	241-249	88	2+	515.3700	1028.7254	1.1593	-
14	AQHEDQVEQYKK	250-261	37	2+	751.9270	1501.8394	0.1234	-
15	NSNLVGAAHEELQQSR	281-296	81	2+	877.5220	1753.0294	1.1744	-
16	IDSLSAQLSQLQK	299-311	40	2+	715.9760	1429.9374	0.1598	-
17	LRDLEDSLAR	320-329	49	2+	594.2190	1186.4234	-0.2071	-
18	DLEDSLAR	322-329	38	2+	459.7510	917.4874	0.0420	-
19	LLADKER	337-343	22	2+	422.8530	843.6914	0.2101	-
20	MQQQLDEYQELLDIK	352-366	42	2+	955.4290	1908.8434	-0.0704	Oxidation
21	LLEGEEER	379-386	37	2+	487.8390	973.6634	0.1918	-
22	ASSHSSQTQGTGSITK	402-417	18	2+	788.9090	1575.8034	0.0546	-
23	VAVEEVDEEGK	441-451	77	2+	602.3780	1202.7414	0.1748	-
24	TALINSTGEEVAMR	529-542	89	2+	754.3780	1506.7414	0.0067	Oxidation

Spot no. 661 (Annexin A2)

Peptide no.	Sequence	Residue	Ions score	Charge state	Observed m/z (Da)	Precursor m/Z (Da)	Mass error observed (Da)	Modification
1	LSLEGDHSTPPSAYGSVK	11-28	31	3+	615.9280	1844.7622	0.8670	-
2	AYTNFDAER	29-37	32	2+	543.3760	1084.7374	-0.7403	-
3	DALNIETAIK	38-47	66	2+	544.4010	1086.7870	0.1954	-
4	GVDEVTIVNILTNR	50-63	100	2+	772.0010	1541.9874	0.1461	-
5	QDIAFAYQR	69-77	60	2+	556.3840	1110.7534	0.2077	-
6	TPAQYDASELK	105-115	68	2+	611.9170	1221.8194	0.2317	-
7	GLGTDEDSLIEIICSR	120-135	12	2+	889.4610	1776.9074	0.0511	-
8	TNQELQEINR	136-145	65	2+	622.9340	1243.8534	0.2378	-
9	VYKEMYK	146-152	17	2+	488.7900	975.5654	0.0919	Oxidation
10	DIISDTSGDFR	158-168	69	2+	613.3910	1224.7674	0.2052	-
11	DIISDTSGDFRK	158-169	36	2+	677.4070	1352.7994	0.1422	-
12	RAEDGSVIDYELIDQDAR	179-196	79	3+	689.0650	2064.1732	0.1972	-
13	AEDGSVIDYELIDQDAR	180-196	72	2+	954.9780	1907.9414	0.0666	-
14	SVCHLQK	221-227	21	2+	436.3270	870.6394	0.2013	-
15	SYSPYDMLESIK	234-245	57	2+	724.9380	1447.8614	0.2074	Oxidation
16	SYSPYDMLESIKK	234-246	24	2+	788.4990	1574.9834	-0.7656	Oxidation
17	SEVDMLK	296-302	30	2+	419.2960	836.5774	0.1825	Oxidation
18	SLYYYIQQDTK	314-324	56	2+	711.4300	1420.8454	0.1580	-

Spot no. 296 (Elongation factor 1-delta)

**Peptide no.**	**Sequence**	**Residue**	**Ions score**	**Charge state**	**Observed m/z (Da)**	**Precursor m/Z (Da)**	**Mass error observed (Da)**	**Modification**
1	QENGASVILR	59-68	28	2+	544.2310	1086.4474	0.8645	-
2	IASLEVENQSLR	104-115	84	2+	679.9600	1357.9054	0.1853	-
3	AAAPQTQHVSPMR	144-156	46	2+	705.9430	1409.8714	1.1833	Oxidation
4	SIQLDGLTWGGSK	239-251	81	2+	681.4300	1360.8454	0.1468	-

Spot no. 655 (Peptidyl-prolyl cis-trans isomerase B isoform 2)

Peptide no.	Sequence	Residue	Ions score	Charge state	Observed m/z (Da)	Precursor m/Z (Da)	Mass error observed (Da)	Modification
1	VYFDLR	46-51	36	2+	406.7420	811.4694	0.0466	-
2	IGDEDIGR	52-59	68	2+	437.8010	873.5874	0.1682	-
3	VVIGLFGK	60-67	43	2+	416.8120	831.6094	0.0876	-
4	TVDNFVALATGEK	72-84	89	2+	682.9410	1363.8674	0.1691	-
5	GFGYKDSK	85-92	17	2+	451.3010	900.5874	0.1533	-
6	VIKDFMIQGGDFTR	96-109	95	2+	822.0400	1642.0654	0.2470	Oxidation
7	DFMIQGGDFTR	99-109	63	2+	643.8630	1285.7114	0.1353	-
8	FPDENFK	123-129	38	2+	448.7590	895.5034	0.0959	-
9	LKHYGPGWVSMANAGK	130-145	27	3+	578.0690	1731.1852	0.3289	Oxidation
10	HYGPGWVSMANAGK	132-145	72	2+	745.9590	1489.9034	0.2262	Oxidation
11	DTNGSQFFITTVK	146-158	54	2+	729.9500	1457.8854	1.1656	-
12	VLEGMEVVR	172-180	57	2+	516.3530	1030.6914	0.1433	-
13	DKPLKDVTIADCGK	191-204	63	2+	780.4570	1558.8994	0.0970	-
14	DVTIADCGK	196-204	52	2+	489.7590	977.5034	0.0546	-
15	IEVEKPFAIAK	205-215	50	2+	622.9680	1243.9214	0.2039	-
16	IEVEKPFAIAKE	205-216	48	2+	687.4780	1372.9414	0.1813	-

Spot no. 799 (ATP synthase subunit beta, mitochondrial precursor)

Peptide no.	Sequence	Residue	Ions score	Charge state	Observed m/z (Da)	Precursor m/Z (Da)	Mass error observed (Da)	Modification
1	LVLEVAQHLGESTVR	95-109	42	2+	826.0330	1650.0514	0.1414	-
2	TIAMDGTEGLVR	110-121	64	2+	631.9110	1261.8074	0.1738	-
3	VLDSGAPIK	125-133	44	2+	450.8450	899.6754	1.1631	-
4	VLDSGAPIKIPVGPETLGR	125-143	37	3+	640.7140	1919.1202	1.0314	-
5	IPVGPETLGR	134-143	33	2+	519.7280	1037.4414	-0.1455	-
6	VVDLLAPYAK	189-198	31	2+	544.3640	1086.7134	-0.9143	-
7	IGLFGGAGVGK	202-212	50	2+	488.3520	974.6894	0.1345	-
8	TVLIMELINNVAK	213-225	64	2+	729.4560	1456.8974	0.0651	-
9	VALVYGQMNEPPGAR	265-279	44	2+	801.4300	1600.8454	0.0423	-
10	VALTGLTVAEYFR	282-294	71	2+	720.4320	1438.8494	0.0674	-
11	FTQAGSEVSALLGR	311-324	93	2+	718.3890	1434.7634	0.0168	-
12	AIAELGIYPAVDPLDSTSR	388-406	56	2+	994.9770	1987.9394	0.9132	-

Supplementary Table S2: Summary of potential post-translational modifications (PTMs) of all the identified proteins.

Spot no.	Protein name	Potential PTMs	No. of modified residues	Sequence	Residues	Observed m/z (Da)	Theoretical m/z (Da)	Mass difference (Da)	Known PTMs
181	Tubulin beta-2A chain	Bromination	1	IMNT**F**SVMPSPK	163-174	1445.7730	1367.6698	78.1033	1xMethionine sulfoxide
						1461.7350	1383.6647	78.0703	2xMethionine sulfoxide
		Cysteine sulfinic acid (-SO_2H)	1	NMMAA**C**DPR	298-306	1129.7950	1097.4172	32.3778	1xCarboxyamidomethyl cysteine, 2xMethionine sulfoxide
						1129.8230	1097.4172	32.4058	1xCarboxyamidomethyl cysteine, 2xMethionine sulfoxide
		Cysteine persulfide	1	NMMAA**C**DPR	298-306	1129.7950	1097.4172	32.3778	1xCarboxyamidomethyl cysteine, 2xMethionine sulfoxide
						1129.8230	1097.4172	32.4058	1xCarboxyamidomethyl cysteine, 2xMethionine sulfoxide
		Deamidation followed by a methylation	7	ALTVPELT**QQ**MFDSK	283-297	1722.9410	1707.8622	15.0788	
				MSMKEVDE**Q**MLNV**Q**NK	321-336	1971.1520	1955.8871	15.2649	2xMethionine sulfoxide
				EVDE**Q**MLNV**Q**NK	325-336	1461.7350	1446.6893	15.0457	
				ISE**Q**FTAMFR	381-390	1244.6830	1229.5983	15.0847	
		2,3-didehydroalanine (Ser)	1	ALTVPELTQQMFD**S**K	283-297	1705.8950	1723.8571	-17.9621	Methionine sulfoxide: 293
		Dihydroxylation	2	NMMAACD**PR**	298-306	1129.7950	1097.4172	32.3778	1xCarboxyamidomethyl cysteine, 2xMethionine sulfoxide
						1129.8230	1097.4172	32.4058	1xCarboxyamidomethyl cysteine, 2xMethionine sulfoxide
		Dimethylation	4	FPG**Q**L**N**A**D**L**R**	242-251	1158.7730	1130.5952	28.1778	
						1158.8450	1130.5952	28.2498	
		3',4' Dihydroxyphenylalanine	2	MSAT**F**IGNSTAIQEL**F**K	363-379	1873.9550	1857.9415	16.0135	
		S-farnesyl cysteine	1	NMMAA**C**DPR	298-306	1244.6830	1040.3958	204.2872	2xMethionine sulfoxide
				NMMAA**C**DPRHGR	298-309	1619.9650	1415.6089	204.3561	1xCarboxyamidomethyl cysteine
						1635.9670	1431.6038	204.3632	1xCarboxyamidomethyl cysteine, 1xMethionine sulfoxide
						1636.0430	1431.6038	204.4392	1xCarboxyamidomethyl cysteine, 1xMethionine sulfoxide
		Geranyl-geranylation	1	NMMAA**C**DPRHGR	298-309	1630.8830	1358.5874	272.2956	
		Gamma-carboxyglutamic acid	1	RIS**E**QFTAMFR	380-390	1445.7730	1401.6943	44.0787	Methionine sulfoxide: 388
				IS**E**QFTAMFRR	381-391	1445.7730	1401.6943	44.0787	Methionine sulfoxide: 388
		O-GlcNAc	1	FPGQL**N**ADLRK	242-252	1461.7350	1258.6902	203.0448	
		Glutathionylation	1	NMMAA**C**DPRHGR	298-309	1695.9470	1390.5773	305.3697	2xMethionine sulfoxide
		Hydroxylation	5	LHFFM**P**GFA**P**LTSR	263-276	1707.8210	1620.8355	86.9855	
				**N**MMAAC**DP**RHGR	298-309	1445.7730	1358.5874	87.1856	
						1461.7350	1374.5823	87.1526	1xMethionine sulfoxide
		Methylation	5	NMMAA**CD**P**RH**G**R**	298-309	1445.7730	1431.6038	14.1692	1xCarboxyamidomethyl cysteine, 1xMethionine sulfoxide
						1461.7350	1447.5987	14.1363	1xCarboxyamidomethyl cysteine, 2xMethionine sulfoxide
		Methionine sulfone	2	N**MM**AACDPR	298-306	1129.7950	1097.4172	32.3778	1xCarboxyamidomethyl cysteine, 2xMethionine sulfoxide
						1129.8230	1097.4172	32.4058	1xCarboxyamidomethyl cysteine, 2xMethionine sulfoxide
		Phosphorylation	6	AILV**D**LEPG**T**M**DS**VR	63-77	1695.9470	1615.8359	80.1111	
				**T**AVC**D**IPPR	351-359	1051.6610	971.4978	80.1632	
		1-Thioglycine	1	MSATFI**G**NSTAIQELFK	363-379	1873.9550	1857.9415	16.0135	
		Triiodothyronine	1	HGR**Y**LTVAAIFR	307-318	1873.9550	1403.7906	470.1644	
		Thyroxine	1	**Y**VPR	59-62	1129.7950	534.3034	595.4916	
						1129.8230	534.3034	595.5196	
		Trimethylation	1	NMMAA**CD**P**R**	298-306	1051.6610	1008.4059	43.2550	
610	Lamin A/C	Acetylation	3	LLAD**K**ER	337-343	886.6610	844.4887	42.1724	
				**K**QLQDEMLR	181-189	1202.7410	1160.6092	42.1318	
				LALDMEIHAYR**K**	367-378	1501.8390	1459.7725	42.0665	
		ADP-ribosylation	5	Q**R**	234-235	843.6910	303.1775	540.5135	
				K**R**	418-419	843.6910	303.2139	540.4771	
				**R**K	419-420	843.6910	303.2139	540.4771	
				**E**L**E**K	262-265	1059.6610	518.2820	541.3790	
				A**R**NTK	118-122	1130.7150	589.3416	541.3734	
		Amidation	1	TQSPQNCSI**M**	656-665	1164.6230	1165.4976	-0.8746	1xCarboxyamidomethyl cysteine
		N6-1-carboxyethyl lysine	1	LEAALGEA**K**	172-180	973.6630	901.4989	72.1641	
		Cis-14-hydroxy-10,13-dioxo-7-heptadecenoic acid aspartate ester	2	LVEI**D**NGK	226-233	1181.7870	887.4832	294.3038	
				EEL**D**FQK	202-208	1202.7410	908.4360	294.3050	
		Citrullination	5	**R**VDAEN**R**	190-196	860.6850	859.4380	1.2470	
				LSPSPTSQ**R**	389-397	973.6630	972.5109	1.1521	
				SLETENAGL**R**L**R**	51-62	1359.8370	1358.7386	1.0984	
		Cysteine sulfenic acid	1	TQSPQN**C**SIM	656-665	1181.7870	1165.4976	16.2894	1xCarboxyamidomethyl

									cysteine
		Deamidation	3	LSPSPTS**Q**R	389-397	973.6630	972.5109	1.1521	
				NKSSED**Q**SMGNW**Q**IK	457-471	1753.0290	1751.8017	1.2273	
		Deamidation followed by a methylation	9	EGDLMAA**Q**AR	124-133	1076.7330	1061.5044	15.2286	
				LVEIDNGK**Q**R	226-235	1186.4230	1171.6429	14.7801	
				G**Q**VTKLEAALGEAK	167-180	1429.9370	1414.7900	15.1470	
				L**Q**TLKEELDF**Q**K	197-208	1506.7410	1491.8053	14.9357	
				M**QQQ**LDEY**Q**ELLDIK	352-366	1908.8430	1893.9262	14.9168	
		2,3-didehydroalanine (Ser)	8	KTLD**S**VAK	90-97	843.6910	861.5040	-17.8130	
				KLE**SS**E**S**R	420-427	917.4870	935.4792	-17.9922	
				ITE**S**EEVV**S**R	63-72	1130.7150	1148.5793	-17.8643	
				TQ**S**PQNC**S**IM	656-665	1147.7370	1165.4976	-17.7606	1xCarboxyamidomethyl cysteine
		Dimethylation	18	QLAA**KE**A**K**	312-319	886.6610	858.5043	28.1567	
				QL**QDE**ML**R**	182-189	1076.7330	1048.5092	28.2238	Methionine sulfoxide: 187
				KLL**E**G**EEER**	378-386	1130.7150	1102.5738	28.1412	
				EAALSTALS**EK**	145-155	1147.7370	1119.5892	28.1478	
				LAL**D**M**E**I**H**AY**R**	367-377	1359.8370	1331.6776	28.1594	
		Diphthamide	1	LALDMEI**H**AYR	367-377	1490.8970	1347.6725	143.2245	Methionine sulfoxide: 371
		Gamma-carboxyglutamic acid	1	QLQD**E**MLR	182-189	1076.7330	1032.5142	44.2188	
		Glutathionylation	1	TQSPQN**C**SIM	656-665	1429.9370	1124.4711	305.4659	1xMethionine sulfoxide
		Hydroxylation	9	LQLELS**K**	102-108	917.4870	830.4982	86.9888	
				LS**P**S**P**TSQR	389-397	1059.6610	972.5109	87.1501	
				EG**D**LMAAQAR	124-133	1164.6230	1077.4993	87.1237	Methionine sulfoxide: 128
				GQVT**K**LEAALGEA**K**	167-180	1501.8390	1414.7900	87.0490	
				SSE**D**QSMG**N**WQI**K**	459-471	1628.7990	1541.6536	87.1454	1xMethionine sulfoxide, 1xOxidized tryptophan
				SSE**D**QSMG**N**WQI**K**R	459-472	1753.0290	1665.7649	87.2641	
		Hypusine	4	LQLELS**K**	102-108	917.4870	830.4982	86.9888	
				GQVT**K**LEAALGEA**K**	167-180	1501.8390	1414.7900	87.0490	
				SSEDQSMGNWQI**K**	459-471	1628.7990	1541.6536	87.1454	1xMethionine sulfoxide, 1xOxidized tryptophan
				SSEDQSMGNWQI**K**R	459-472	1753.0290	1665.7649	87.2641	
		Methylation	8	LAVYI**DR**V**R**	42-50	1118.6410	1104.6524	13.9886	
				QL**QDE**ML**RR**	182-190	1202.7410	1188.6153	14.1257	
		Myristoylation	2	EELDFQ**K**	202-208	1118.6410	908.4360	210.2050	
				SSEDQSMGNWQI**K**	459-471	1752.0140	1541.6536	210.3604	1xMethionine sulfoxide, 1xOxidized tryptophan
		n-Octanoate	1	METP**S**QRR	1-8	1130.7150	1004.4942	126.2208	
		Omega-hydroxyceramide glutamate ester	3	IT**E**S**EE**VVSR	63-72	1908.8430	1148.5793	760.2637	
		Phosphorylation	9	ER**DTS**R	330-335	843.6910	763.3693	80.3217	
				**T**VLCG**T**CGQPA**D**K	586-598	1429.9370	1349.6188	80.3182	1xCarboxyamidomethyl cysteine
				**H**E**T**RLVEI**D**NGK	222-233	1490.8970	1410.7335	80.1635	
		1-Thioglycine	3	LEAAL**G**EAK	172-180	917.4870	901.4989	15.9881	
				KLLE**G**EEER	378-386	1118.6410	1102.5738	16.0672	
				AAYEAEL**G**DAR	79-89	1181.7870	1165.5483	16.2387	
		Thyroxine	1	T**Y**SAK	266-270	1164.6230	569.2929	595.3301	
		Trimethylation	18	LAVYI**DR**V**R**	42-50	1147.7370	1104.6524	43.0846	
				TYSA**K**L**DN**A**R**	266-275	1181.7870	1138.5851	43.2019	
				L**DN**A**RQ**SA**ER**	271-280	1202.7410	1159.5814	43.1596	
				R**QN**G**DD**PLLTY**R**	472-483	1490.8970	1447.7288	43.1682	
		N6,N6,N6-trimethyl-5-hydroxylysine	4	QLAA**K**EA**K**	312-319	917.4870	858.5043	58.9827	
				LEAALGEA**KK**	172-181	1088.6970	1029.5938	59.1032	
661	Annexin A2	ADP-ribosylation	1	**E**MYK	149-152	1110.7530	570.2592	540.4938	
						1110.7530	570.2592	540.4938	
		N6-1-carboxyethyl lysine	3	EMY**K**TDLE**K**	149-157	1244.7810	1172.5503	72.2307	Methionine sulfoxide: 150
				T**K**GVDEVTIVNILTNR	48-63	1843.9110	1771.9912	71.9198	
		Cis-14-hydroxy-10,13-dioxo-7-heptadecenoic acid aspartate ester	2	GTR**D**K	282-286	870.6390	576.3100	294.3290	
				YGKSLYYYIQQ**D**TK	311-324	2064.1730	1769.8745	294.2985	
		Citrullination	1	LMVALAKG**R**	170-178	975.5650	974.5815	0.9835	Methionine sulfoxide: 171
		2,3-didehydroalanine (Ser)	3	VLIRIMV**S**R	287-295	1084.7370	1102.6765	-17.9395	Methionine sulfoxide: 292
				WI**S**IMTER**S**VCHLQK	213-227	1844.7620	1862.9251	-18.1631	1xMethionine sulfoxide, 1xOxidized tryptophan
		Dihydroxylation	3	GVDEVTIVNILTN**R**	50-63	1574.9830	1542.8485	32.1345	
				TDLE**K**DIISDTSGDF**R**	153-168	1843.9110	1811.8657	32.0453	
		S-farnesyl cysteine	1	ALLYL**C**GGDD	330-339	1243.8530	1039.4764	204.3766	
		Geranyl-geranylation	1	SV**C**HLQK	221-227	1086.7870	814.4240	272.3630	
		O-GlcNAc	8	**SN**EQR	64-68	836.5770	633.2951	203.2819	
				LYD**S**MK	274-279	975.5650	772.3546	203.2105	Methionine sulfoxide: 278
				M**ST**VHEILCK	1-10	1420.8450	1217.6017	203.2433	1xCarboxyamidomethyl cysteine
				**TN**QELQEI**N**R	136-145	1447.8610	1244.6229	203.2381	
		Hydroxylation	3	EMY**K**T**D**LE**K**	149-157	1243.8530	1156.5554	87.2976	
		Hypusine	2	EMY**K**TDLE**K**	149-157	1243.8530	1156.5554	87.2976	
		Methylation	13	ALLYL**C**GG**DD**	330-339	1110.7530	1096.4979	14.2551	Carboxyamidomethyl cysteine: 335

				SYSPYDMLESIKK	234-246	1574.9830	1560.7614	14.2216	
				WISIMTERSVCHLQK	213-227	1844.7620	1830.9353	13.8267	
		Myristoylation	1	TNQELQEINRVYK	136-148	1844.7620	1634.8496	209.9124	
		n-Octanoate	5	SYSPYDMLESIK	234-245	1574.9830	1448.6614	126.3217	Methionine sulfoxide: 240
				SALSGHLETVILGLLK	89-104	1776.9070	1650.9788	125.9282	
						1777.4450	1650.9788	126.4662	
		Phosphorylation	3	LYDSMK	274-279	836.5770	756.3596	80.2174	
		Pyridoxal phosphate	1	SVCHLQKVFER	221-231	1574.9830	1345.7045	229.2785	
		Sulfation	1	LYDSMK	274-279	836.5770	756.3596	80.2174	
		Triiodothyronine	1	YGK	311-313	836.5770	367.1976	469.3794	
		Trimethylation	2	WISIMTER	213-220	1110.7530	1067.5190	43.2340	1xMethionine sulfoxide, 1xOxidized tryptophan
296	Elongation factor 1-delta	Dihydroxylation	4	LSALEKSSPTHR	132-143	1357.9050	1325.7171	32.1878	
				SIQLDGLTWGGSK	239-251	1409.8710	1377.7008	32.1702	Oxidized tryptophan: 247
		Hydroxylation	3	IWFDKFK	31-37	1086.4470	999.5298	86.9172	Oxidized tryptophan: 32
		Hypusine	2	IWFDKFK	31-37	1086.4470	999.5298	86.9172	Oxidized tryptophan: 32
		Methylation	12	LQIQCVVEDDK	262-272	1360.8450	1346.6620	14.1830	Carboxyamidomethyl cysteine: 266
				VGTDLLEEEITK	273-284	1360.8450	1346.7049	14.1401	
		Trimethylation	5	FKYDDAER	36-43	1086.4470	1043.4792	42.9678	
655	Peptidyl-prolyl cis-trans isomerase B isoform 2	Acetylation	1	DSKFHR	90-95	831.6090	789.4002	42.2088	
		Amidation	1	IEVEKPFAIAKE	205-216	1372.9410	1373.7674	-0.8264	
		Bromination	2	DFMIQGGDFTR	99-109	1363.8990	1286.5834	77.3156	
		Deamidation followed by a methylation	2	DFMIQGGDFTR	99-109	1301.5970	1286.5834	15.0136	
						1301.6150	1286.5834	15.0316	
						1301.7010	1286.5834	15.1176	
						1301.8130	1286.5834	15.2296	
						1301.9150	1286.5834	15.3316	
				VIKDFMIQGGDFTR	96-109	1642.0650	1626.8308	15.2342	
						1642.1060	1626.8308	15.2752	
		2,3-didehydroalanine (Ser)	1	HYGPGWVSMANAGK	132-145	1456.8270	1474.6896	-17.8625	
		Dihydroxylation	7	FHRVIK	93-98	831.6090	799.4937	32.1153	
				DTNGSQFFITTVK	146-158	1489.7240	1457.7270	31.9969	
						1489.9030	1457.7270	32.1760	
				TAWLDGKHVVFGK	159-171	1489.7240	1457.7899	31.9341	
						1489.9030	1457.7899	32.1131	
				HVVFGKVLEGMEVVR	166-180	1731.1850	1698.9359	32.2491	
		Dimethylation	2	VVIGLFGKTVPK	60-71	1285.7110	1257.7929	27.9181	
		3',4' Dihydroxyphenylalanine	4	DFMIQGGDFTR	99-109	1302.5810	1286.5834	15.9976	
				TAWLDGKHVVFGK	159-171	1489.7240	1473.7848	15.9392	Oxidized tryptophan: 161
						1489.9030	1473.7848	16.1182	Oxidized tryptophan: 161
				HVVFGKVLEGMEVVR	166-180	1731.1850	1714.9308	16.2542	Methionine sulfoxide: 176
		FMN conjugation (His)	1	DSKFHR	90-95	1243.9210	789.4002	454.5208	
		O-GlcNAc	1	DFMIQGGDFTR	99-109	1489.7240	1286.5834	203.1406	
						1489.9030	1286.5834	203.3196	
		Methionine sulfone	1	HVVFGKVLEGMEVVR	166-180	1731.1850	1698.9359	32.2491	
		n-Octanoate	1	MLRLSER	1-7	1030.6910	904.5033	126.1877	
						1030.7370	904.5033	126.2337	
						1046.7310	920.4982	126.2328	Methionine sulfoxide: 1
		1-Thioglycine	6	DFMIQGGDFTR	99-109	1302.5810	1286.5834	15.9976	
				TAWLDGKHVVFGK	159-171	1489.7240	1473.7848	15.9392	Oxidized tryptophan: 161
						1489.9030	1473.7848	16.1182	Oxidized tryptophan: 161
				HVVFGKVLEGMEVVR	166-180	1731.1850	1714.9308	16.2542	Methionine sulfoxide: 176
		Trimethylation	5	IGDEDIGRVVIGLFGK	52-67	1731.1850	1687.9377	43.2473	
799	ATP synthase subunit beta, mitochondrial precursor	Deamidation followed by a methylation	2	GLSPSAALPQAQLLLR	19-34	1650.0510	1634.9588	15.0922	
		Dihydroxylation	4	AHGGYSVFAGVGER	226-239	1438.8490	1406.6811	32.1679	
				VALVYGQMNEPPGAR	265-279	1650.0510	1617.8053	32.2457	Methionine sulfoxide: 272
		Dimethylation	5	AAPAGVHPAR	35-44	974.6890	946.5217	28.1673	
				AHGGYSVFAGVGER	226-239	1434.7630	1406.6811	28.0819	
		Hydroxylation	3	IMNVIGEPIDER	144-155	1472.9810	1385.7093	87.2717	
		Methylation	3	GLSPSAALPQAQLLLR	19-34	1648.9520	1634.9588	13.9932	
		Methionine sulfone	1	VALVYGQMNEPPGAR	265-279	1650.0510	1617.8053	32.2457	Methionine sulfoxide: 272
